# A two-sample Mendelian randomization analysis of modifiable risk factors and intracranial aneurysms

**DOI:** 10.1038/s41598-022-11720-9

**Published:** 2022-05-10

**Authors:** Danyang Tian, Linjing Zhang, Zhenhuang Zhuang, Tao Huang, Dongsheng Fan

**Affiliations:** 1 Department of Neurology, Peking University Third Hospital, No. 49, North Garden Rd., Haidian District, Beijing, 100191 China; 2 Beijing Municipal Key Laboratory of Biomarker and Translational Research in Neurodegenerative Diseases, No. 49, North Garden Rd., Haidian District, Beijing, 100191 China; 3 Department of Epidemiology and Biostatistics, School of Public Health, Peking University, No. 38, Xueyuan Rd., Haidian District, Beijing, 100191 China

**Keywords:** Neurology, Cerebrovascular disorders

## Abstract

We aimed to investigate the causality between potentially modifiable risk factors and the risk of intracranial aneurysm. Genetic instruments for 51 modifiable factors and intracranial aneurysm data were obtained from recently published genome-wide association studies. We applied two-sample Mendelian randomization methods to investigate their causal relationships. Genetically predicted cigarettes per day, smoking initiation, systolic blood pressure, hypertension and body fat percentage were significantly associated with an increased risk of intracranial aneurysm [odds ratios (OR) 2.67, 95% confidence interval (CI) 1.75–4.07, *p* = 5.36 × 10^–6^, OR 1.53, 95% CI 1.32–1.77, *p* = 9.58 × 10^–9^, OR 1.05, 95% CI 1.02–1.08, *p* = 1.18 × 10^–3^, OR 1.65, 95% CI 1.19–2.28, *p* = 2.56 × 10^–3^ and OR 1.29, 95% CI 1.11–1.52, *p* = 1.33 × 10^–3^, respectively]. Type 2 diabetes mellitus was significantly associated with a decreased risk of intracranial aneurysm (OR 0.89, 95% CI 0.83–0.95, *p* = 8.54 × 10^–4^). Body fat percentage was significantly associated with subarachnoid haemorrhage (*p* = 5.70 × 10^–5^). This study provided genetic evidence of causal effects of smoking, blood pressure, type 2 diabetes mellitus and obesity on the risk of intracranial aneurysm.

## Introduction

Intracranial aneurysms (IAs) are abnormal dilations of the intracranial vessels, in which all the layers of the vascular wall are affected by degenerative changes that lead to distension of the vessel. IA, which accounts for about 3% in the population, continues to contribute a large proportion of morbidity and mortality^1^. IA rupture is the main cause of subarachnoid haemorrhage (SAH), a stroke subtype that leads to severe morbidity and mortality^2^. Therefore, it may be worth identifying the causal factors in development of IAs for early targeted intervention.

Previous epidemiological studies have shown that traditional risk factors such as smoking and hypertension were related to IA risk^3–5^. A meta-analysis of observational studies with inconsistent results showed that diabetes mellitus is related to a decreased risk of aneurysmal subarachnoid haemorrhage^6^. Although no direct evidence of a relationship between obesity and IA, a systematic review showed that adiposity was associated with higher risk of abdominal aortic aneurysm^7^ and mortality due to subarachnoid haemorrhage^8^. Moreover, lipid-lowering agents and high levels of high-density lipoprotein were inversely associated with IA rupture^9^. However, available evidence from observational studies mainly rely on self-reported information and are subject to bias of reverse causation and measured or unmeasured confounding factors. Thus, the causality behind the observations is largely unknown.

Mendelian randomization (MR) is a method for investigating the causal relationship between risk factors and outcomes from a genetic perspective. Genetic variants were used as instrumental variables. Above-mentioned confounding and reverse causation are avoided in MR studies since genetic alleles are randomly assorted during conception^10^. Limited evidence from MR for ICAs has been reported^11,12^. A MR study using the data from UK-Biobank showed a causal link between smoking and the risk of SAH^12^. While, another MR study found that diabetes mellitus and obesity were not causally associated with IAs^11^.

Therefore, we leveraged the most updated genome-wide association study (GWAS) data for modifiable risk factors and applied a two sample MR approach to examine the causal relationship between modifiable risk factors and IAs.

## Methods

### Study design

**Figure 1:**
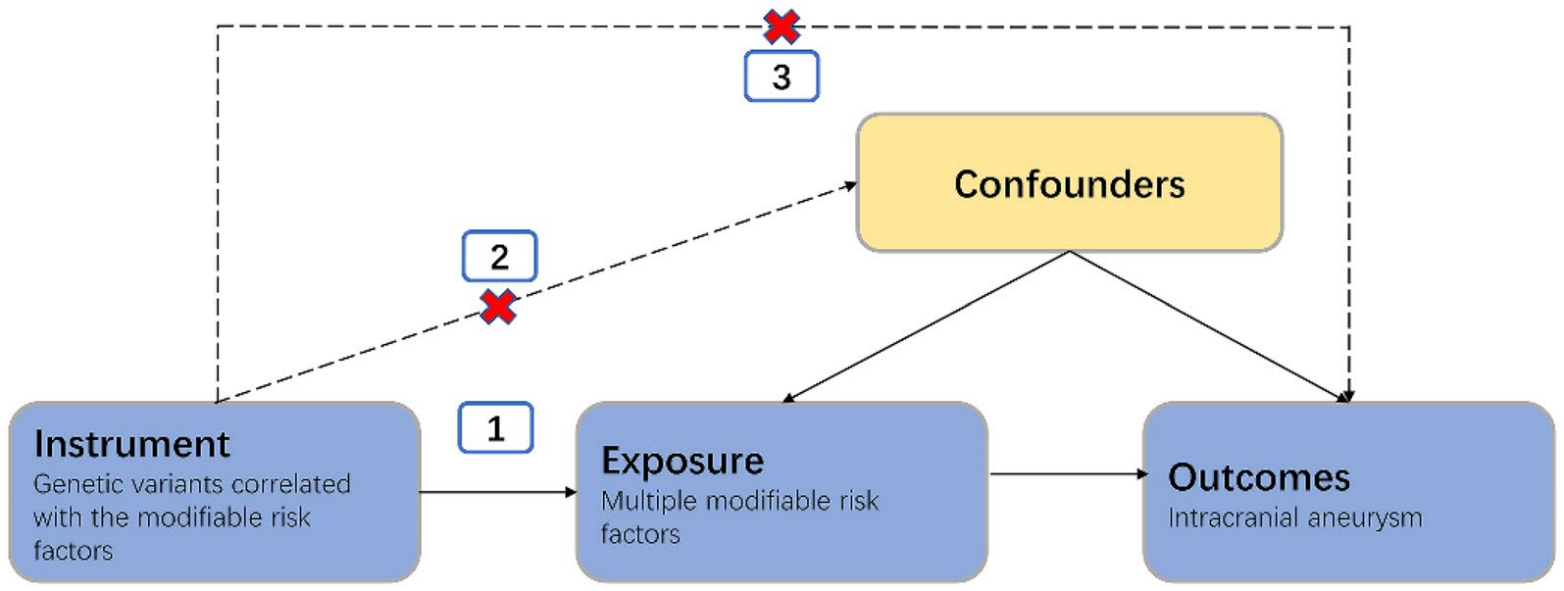
Schematic representation of Mendelian randomization analysis. Broken lines represent potential pleiotropic or direct causal effects between variables that would violate Mendelian randomization assumptions.

This study follows the three assumptions of MR studies^13^. First, the genetic variants selected as instrumental variables are associated with the investigated modifiable risk factors; second, the genetic variants are not associated with any unmeasured confounders; third, the genetic variants are associated with IA only through the investigated modifiable risk factors, not through other pathways (Fig. 1). We applied two-sample MR analyses in this research (Supplement Fig. 1)^14,15^.

### Modifiable risk factors

We included 51 modifiable risk factors that are reported to be associated with cerebral vascular diseases. The risk factors could be classified into the following categories: lifestyle (infant head circumference; the quality of being a morning person; sleep duration; smoking; alcohol intake; and physical activity), cardiometabolic (blood pressure; blood lipids; T2DM and blood glucose-related traits; obesity; and carotid atherosclerosis), nutrient and dietary intake (homocysteine; vitamins B12, A1, and D; coffee intake; and gut microbiota-dependent metabolites), impaired renal function (estimated glomerular filtration rate and albuminuria) and inflammation and immune abnormalities (systemic lupus erythematosus; Crohn’s disease, periodontitis; interleukin-18; interleukin-1Ra; and C-reactive protein).

### Data sources

Genetic variants for modifiable risk factors were collected from 45 published GWASs (Supplement Table 1). The genetic data of IA were acquired from the recently published GWAS, which included 10,754 cases and 306,882 controls of European and East Asian ancestry^16^.

### Ethics approval

No patients were involved in the design of the study, and no ethical approval from an institutional review board was required, since all analyses were based on publicly available summary statistics.

### Genetic variants

Single nucleotide polymorphisms (SNPs) associated with the previously mentioned risk factors at thresholds for genome-wide significance (*P* < 5 × 10^–8^) were selected (Supplement Table 2). We assessed linkage disequilibrium (defined as r2 < 0.01) with other genetic variants to ensure the independence of genetic variants through the website https://snipa.helmholtz-muenchen.de/snipa3/index.php. The variant with the lowest P value for association with the risk factor was selected when we encountered linkage disequilibrium. In instances where SNPs were not available in a dataset because of poor imputation quality, we replaced them with proxy SNPs if available (r^2^ > 0.9). We additionally removed the SNPs associated with more than one trait to conduct a sensitivity analysis.

### Statistical analysis

We performed two-sample MR analyses to evaluate the impact of modifiable risk factor-associated variants on IA in trans-ethnic populations. We further evaluated the impact of significant risk factors on IA between ruptured and unruptured IAs. Our primary analysis used an inverse variance-weighted (IVW) meta-analysis approach, which is considered conventional MR. We then performed secondary analyses using weighted median, simple median, and MR-Egger regression approaches. We assessed the potential role of directional pleiotropy by testing the intercept value from MR-Egger regression. We then used a leave-one-out analysis to investigate the influence of outlying and/or pleiotropic genetic variants. We used “r2 = beta2/(se2*(n − 2) + beta2)” to calculated r2 of single SNP, and we added each SNP of the exposure together to calculate r2. The strength of the genetic instruments was tested with the F-statistic (https://shiny.cnsgenomics.com/mRnd/). Univariable MR analysis and multivariable MR analysis using MR-base are conducted between exposures which are associated in two-sample MR analysis and IA.

We conducted the statistical analysis using R version 3.3.3 (R Foundation). A Bonferroni-corrected significance threshold of *p* = 9.80 × 10^–3^ (0.05/51 [51 exposures and 1 outcome (IA in trans-ethnic populations)]) was prespecified to adjust for multiple testing. Associations with p values between 0.05 and 9.80 × 10^–3^ were considered suggestive evidence of a possible association.

### Submit statements

All the authors declare the work is not under consideration by another journal, and has not been published previously. And if this manuscript is accepted, it will not be published elsewhere in the same form. All authors have read, validate the accuracy of the data and approved the final manuscript.

## Results

### Lifestyle

**Figure 2:**
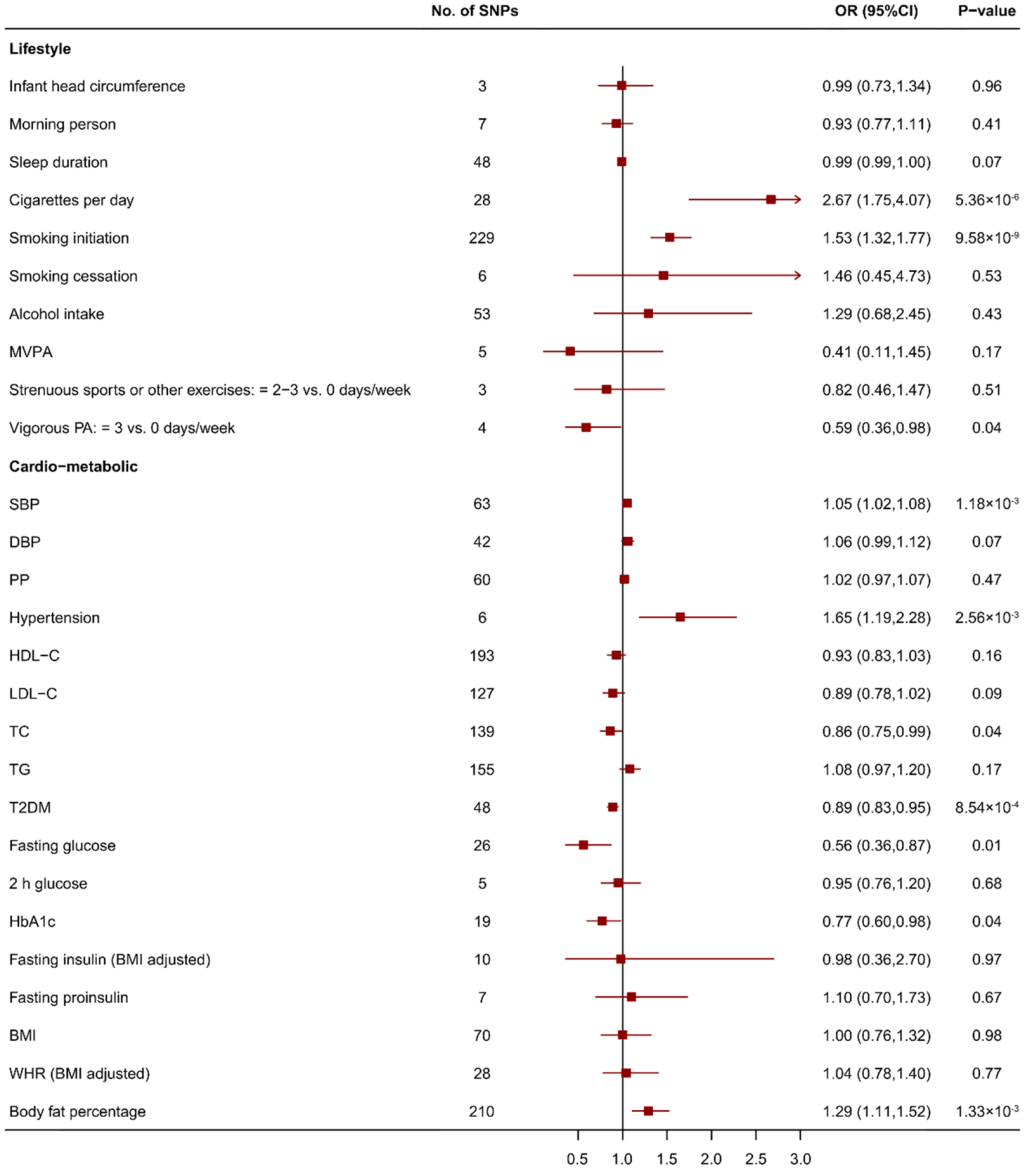
Mendelian randomization for associations between traits of lifestyle and cardio-metabolic risk factors and intracranial aneurysm. SNP: single nucleotide polymorphism; OR: odds ratios; CI: confidence interval; BMI: body mass index; MVPA: moderate-to-vigorous physical activity; SBP: systolic blood pressure; DBP: diastolic blood pressure; PP: pulse pressure; HDL-C: high-density lipoprotein cholesterol; LDL-C: low-density lipoprotein cholesterol; TC: total cholesterol; TG: triglyceride; T2DM: type 2 diabetes mellitus; HbA1c: hemoglobin A1c; WHR: waist hip ratio.

Genetically predicted cigarettes per day and smoking initiation were significantly associated with an increased risk of IA (odds ratio (OR) 2.67, 95% confidence interval (CI) 1.75–4.07, *p* = 5.36 × 10^–6^, and OR 1.53, 95% CI 1.32–1.77, *p* = 9.58 × 10^–9^, respectively), which means people whose cigarettes per day or smoking initiation was 1 SD above the population will have 2.67 or 1.53 times increase in risk to IA compared with the population prevalence Genetically predicted vigorous physical activity ≥ 3 vs. 0 days/week is suggestively associated with a decreased risk of IA (OR 0.59, 95% CI 0.36–0.96, *p* = 0.04). (Fig. 2) The significant association between cigarettes per day, smoking initiation and IA was consistent in sensitivity analyses that used simple median, weighted median analyses and MR-Egger analysis but not the association between physical activity and IA (Supplement Table 3). No SNP was detected to possibly drive the positive association in leave-one-out analyses (Supplement Data). No evidence of directional pleiotropy was found in MR-Egger analyses (*p* = 0.19, 0.26 and 0.60, respectively, Supplement Table 3).

### Cardiometabolic traits

**Figure 3:**
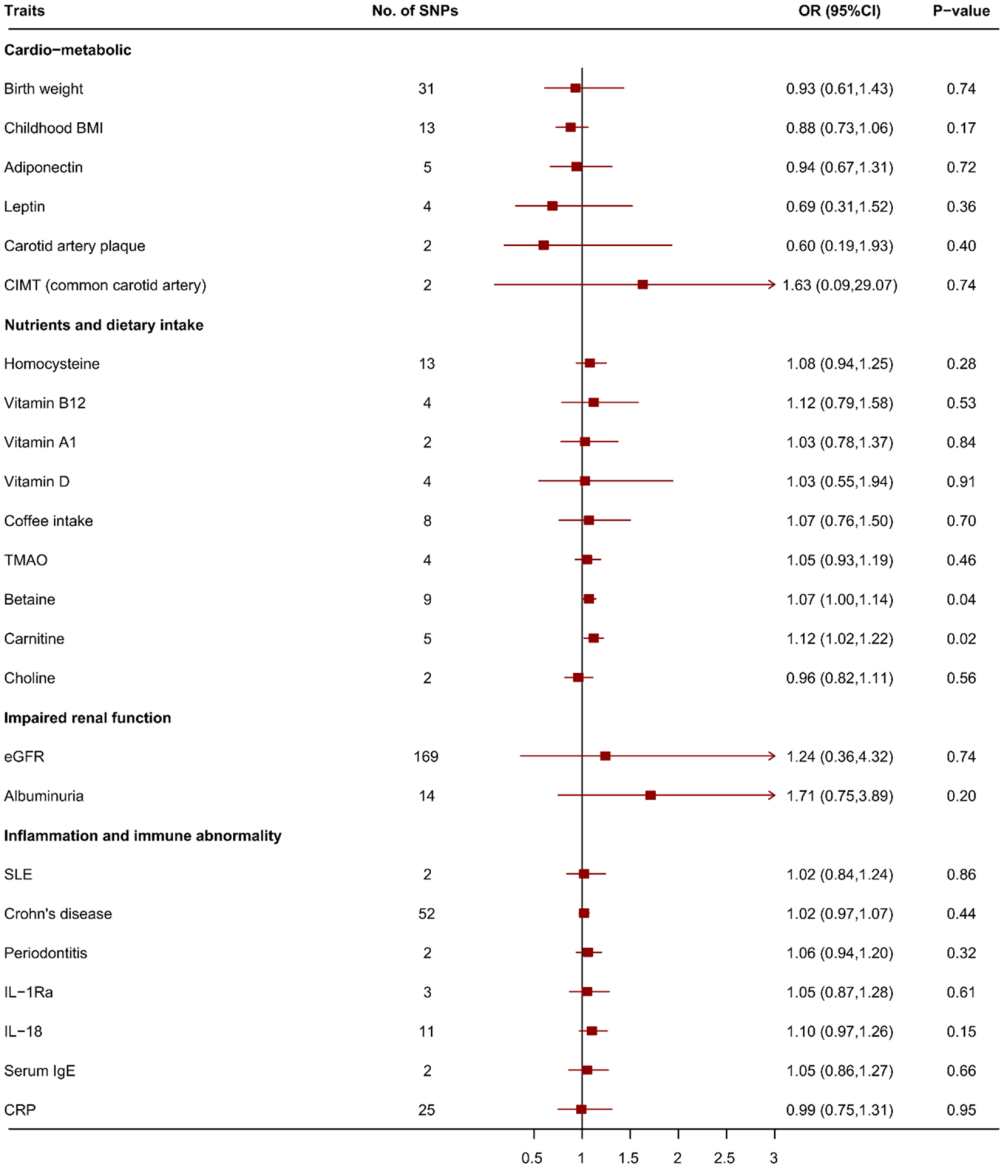
Mendelian randomization for associations between traits of cardio-metabolic, nutrients and dietary intake, impaired renal function and inflammation risk factors and intracranial aneurysm. SNP: single nucleotide polymorphism; OR: odds ratios; CI: confidence interval; BMI: body mass index; CIMT: carotid intima media thickness; CRP: C reactive protein; eGFR: estimated glomerular filtration rate; SLE: systemic lupus erythematosus; TMAO: trimethylamine-n-oxide.

Genetically predicted systolic blood pressure, hypertension and body fat percentage were significantly associated with an increased risk of IA (OR 1.05, 95% CI 1.02–1.08, *p* = 1.18 × 10^–3^, OR 1.65, 95% CI 1.19–2.28, *p* = 2.56 × 10^–3^ and OR 1.29, 95% CI 1.11–1.52, *p* = 1.33 × 10^–3^, respectively). Genetically predicted type 2 diabetes mellitus was significantly associated with a decreased risk of IA (OR 0.89, 95% CI 0.83–0.95, *p* = 8.54 × 10^–4^). Genetically predicted fasting glucose, haemoglobin A1c (HbA1c) and total cholesterol were suggestively associated with a decreased risk of IA (OR 0.56, 95% CI 0.36–0.87, *p* = 0.01, OR 0.77, 95% CI 0.60–0.98, *p* = 0.04 and OR 0.86, 95% CI 0.75–0.99, *p* = 0.04, respectively) (Figs. 2, 3). The significant association between systolic blood pressure, hypertension, type 2 diabetes and IA was consistent in sensitivity analyses that used simple median and weighted median analyses. The significant association between body fat percentage, fasting glucose, HbA1c and IA was consistent in sensitivity analyses that used simple median analyses (Supplement Table 3). The suggestive association between HbA1c and IA may have been driven by rs12219514, rs12221133, rs174584, rs7356034 and rs895636 in the leave-one-out analysis (*p* = 0.06, 0.05, 0.05, 0.07 and 0.05, respectively, Supplement Data). Directional pleiotropy might exist in the suggestive association between HbA1c and IA in MR-Egger analysis (*p* = 0.02, Supplement Table 3).

### Nutrients and dietary intake

Genetically predicted betaine and carnitine were suggestively associated with an increased risk of IA (OR 1.07, 95% CI 1.00–1.14, *p* = 0.04, OR 1.12, 95% CI 1.02–1.22, *p* = 0.02, respectively) (Fig. 3) The significant association between carnitine and IA was consistent in sensitivity analyses that used simple median and weighted median analyses. The significant association between betaine and IA was consistent in sensitivity analyses that used simple median analyses (Supplement Table 3). No SNP was detected to possibly drive the positive association in leave-one-out analysis (Supplement Data). No evidence of directional pleiotropy was found in MR-Egger analysis (*p* = 0.53 and 0.81, respectively, Supplement Table 3).

### Impaired renal function and inflammation and immune abnormalities

No significant association was found between the related risk factors and IA. (Fig. 3).

### The impact of significant risk factors on ruptured and unruptured IAs

**Figure 4:**
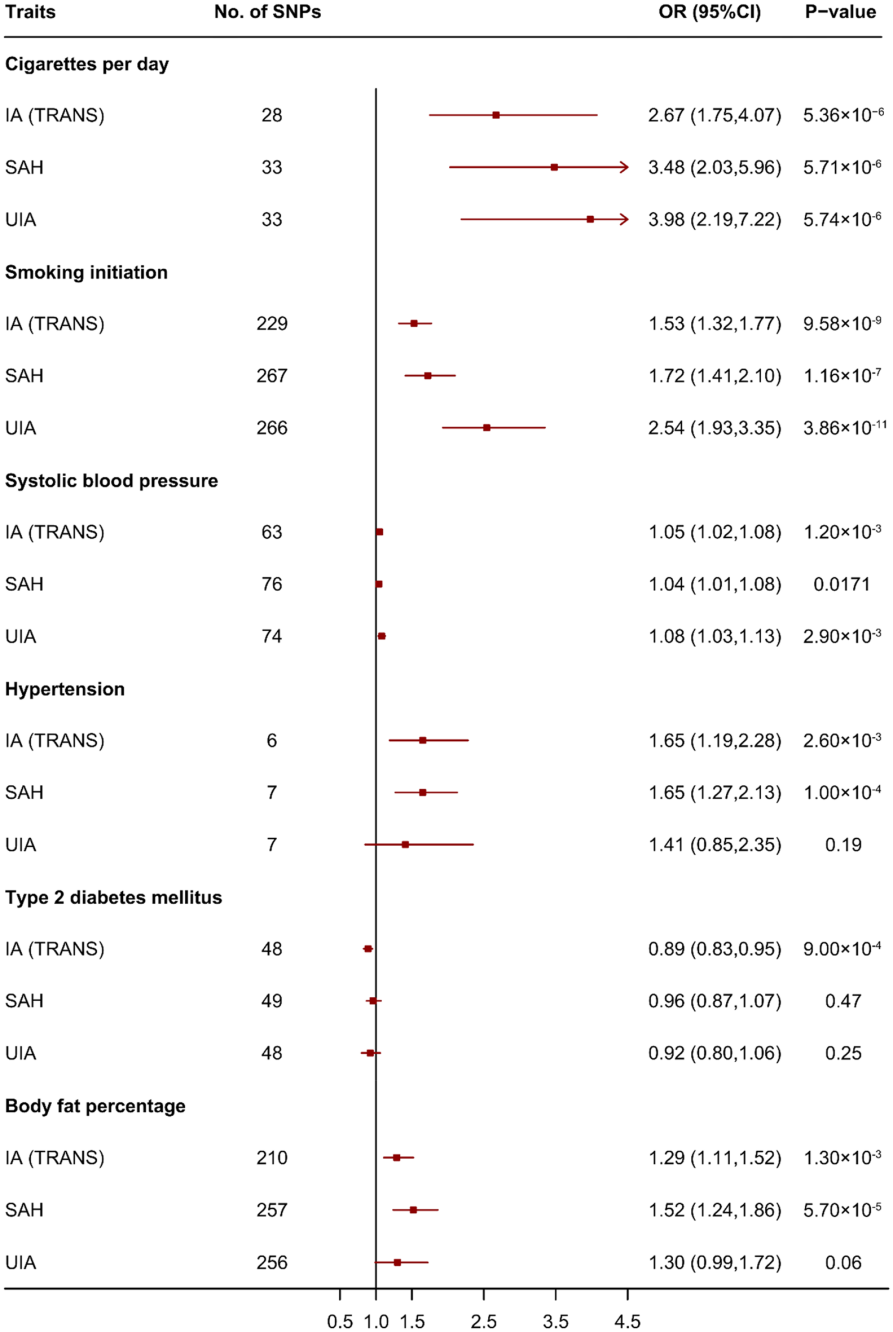
Mendelian randomization for associations between significant risk factors and intracranial aneurysm, subarachnoid haemorrhage and unruptured intracranial aneurysm. SNP: single nucleotide polymorphism; OR: odds ratios; CI: confidence interval; IA: intracranial aneurysm; SAH: subarachnoid haemorrhage; UIA: unruptured intracranial aneurysm; TRANS: trans-ethnics.

The impact of significant risk factors on ruptured and unruptured IAs is shown in Fig. 4. The impact of cigarettes per day, smoking initiation and systolic blood pressure on both ruptured and unruptured IA were significant (*p* = 5.71 × 10^–6^, 1.16 × 10^–7^ and 1.71 × 10^–2^ on ruptured IA; *p* = 5.74 × 10^–6^, 3.86 × 10^–11^ and 2.90 × 10^–3^ on unruptured IA, respectively). The impact of hypertension and body fat percentage on ruptured IA was significant (*p* = 1.00 × 10^–4^ and 5.70 × 10^–5^, respectively), but it was not significant on unruptured IA (*p* = 0.19 and 0.06, respectively). The impact of type 2 diabetes mellitus on both ruptured and unruptured IAs was not significant (*p* = 0.47 for ruptured IAs and 0.25 for unruptured IAs).

### Multivariable MR analysis

Since there a relatively clear conclusion between hypertension and IA in previous clinical research, hypertension is not included in the multivariable MR analysis. Cigarettes per day, smoking initiation, systolic blood pressure, body fat percentage are included. In the multivariable MR analysis, systolic blood pressure and cigarettes per day are significantly associated with IA. (p < 0,001, *p* = 0.001, respectively) (Supplement Table 7).

## Discussion

This MR study provides genetic evidence that smoking, hypertension and obesity are causally associated with a high risk of IA; type 2 diabetes mellitus is causally associated with a low risk of IA; gut microbiota-dependent metabolites are suggestively associated with a high risk of IA; and physical activity and blood lipids are suggestively associated with a low risk of IA.

Smoking is an independent risk factor for the formation, growth and rupture of IAs. In a case–control study including 4701 patients with 6411 IAs, the authors found that current cigarette smoking, smoking intensity, and smoking duration were significantly associated with ruptured IAs at presentation^4^. A recently published MR study using data from the UK Biobank also showed a causal relationship between smoking and SAH^12^. This MR study provided genetic evidence for the causal relationship between smoking and IA. The pathogenesis of aneurysm formation includes increased wall shear stress, endothelial dysfunction, atherosclerosis, and altered gene regulation^3^.

Previous population-based study and hospital-based retrospective study showed that hypertension was independently associated with the presence and rupture of IAs^17^.The joint risk of hypertension and smoking was higher than the risks of hypertension and smoking independently^5^. General statistics-based MR (GSMR) using summary statistics for related phenotypes available in the UK Biobank also showed that smoking and hypertension play important roles in the presence of IA^16^. Our MR study using the latest GWAS studies is consistent with the previous observational study and the GSMR study, which confirms the causal role of hypertension on risk of stroke. Hypertension is associated with hemodynamic stress, which might affect the mechanism of IAs formation and rupture.

Although several observational studies showed an inverse relationship between type 2 diabetes mellitus and SAH^6^, few studies showed a protective role of diabetes mellitus on IA presence. On the contrary, diabetes mellitus is a risk factor of IA according to some studies^18,19^. The previous MR study found no relationship between diabetes mellitus and IA presentation^11^. Our MR study showed that diabetes mellitus is causally associated with a decreased risk of IA presence with high consistence of sensitivity studies. Moreover, other glucose related traits, such as fasting glucose and HbA1c are suggestively associated with a decreased risk of IA. Our MR study improved statistic power after updating of latest IA GWAS, comparing with the previous MR study. The researches of protective role of diabetes mellitus on abdominal aortic aneurysm listed the influence of diabetes mellitus on vascular walls, such as remodelling of extracellular matrix, impact of advanced glycoxidation, inflammation and vascular smooth muscle cells homeostasis, which could be the possible reason for protective role on IAs^20^. The further study is still needed.

Although we did not find evidence to support that obesity is associated with IA, a systemic review showed a relationship between obesity and a high risk of abdominal aortic aneurysm^7^. However, a prospective study showed that a higher BMI is associated with a lower risk of mortality in SAH patients^8^. A previous MR study did not find a causal relationship between obesity and IA from a genetic perspective^11^. This MR study found a significant causal relationship between body fat percentage and IA. Additionally, body fat percentage was significantly associated with SAH (*p* = 5.70 × 10^–5^) but not unruptured IA (*p* = 0.06), suggesting that body fat percentage might be associated with IA rupture. Obesity induces vascular endothelial damage and dysfunction, decreases cerebral tight junction protein expression, which is related to IA formation and rupture^21^.

Previous observational studies and fundamental research have shown other risk factors that might be associated with IA, such as physical activity, blood lipids and gut microbiota^9,22,23^. Suggestive associations were found between vigorous physical activity ≥ 2–3 versus 0 days/week, total cholesterol and low risk of IA, and betaine and carnitine and high risk of IA.

The strengths of the study include the design of MR analysis, which avoids bias from reverse causation and confounding and enables us to investigate the causal relationship, the multiple risk factors, the latest GWAS database, which ensure high statistical power and the novel findings such as role of diabetes mellitus and obesity on IAs.

The limitations are as follows. First, pleiotropy exists in the analysis of the relationship between HbA1c and IA. Completely ruling out pleiotropy or an alternative direct causal pathway is a challenge for all MR analyses, particularly for risk factors determined by multiple genetic variants. Furthermore, the result of simple median analysis is consistent with the IVW analysis. Second, we could not exclude bias from population stratification. The ethnic of the exposures are not totally consistent with the ethnic of IA. However, this is a question many MR analysis encountered. Further study with consistent ethnics is still needed. Third, we could not absolutely avoid the possibility that the SNPs we used as instruments were not associated with unmeasured confounders, which existed in almost all MR analyses. However, we removed SNPs associated with more than one trait in this article, and the result is consistent with our main result (Supplement Tables 5, 6). We additionally checked the association of the SNPs we used as instruments with other risk factors that might be associated with IA on the website https://www.ebi.ac.uk/gwas/home. Although we could not confirm that the SNPs used as instruments were not associated with unmeasured confounders, this possibility was relatively small. Fourth, we cannot exclude that our findings might be affected by weak instrumental bias and statistic power^24^, especially in waist/hip ratio (WHR), coffee intake and Crohn’s disease analyses (Supplement Table 2). However, any bias from weak instruments is in the direction of the null, as we are considering the analyses in the two-sample setting^25^. Fifth, since selection and estimation of the coefficients for the exposures are done on the same data set, we could not avoid the potential of winner's curse bias.

## Summary

This study provided genetic evidence of causal effects of type 2 diabetes mellitus and obesity on the risk of IA. Obesity might be associated with IA rupture, and physical activity, blood lipids and gut microbiota are suggestively associated with IA. The causal effects of smoking and intracranial aneurysms are also shown in this study, which is consistent with the previous studies.

## Supplementary Information

Supplementary Information 1.

Supplementary Information 2.

## References

[1] Vlak MH, Algra A, Brandenburg R (2011). Prevalence of unruptured intracranial aneurysms, with emphasis on sex, age, comorbidity, country, and time period: A systematic review and meta-analysis. Lancet Neurol..

[2] Macdonald RL, Schweizer TA (2017). Spontaneous subarachnoid haemorrhage. Lancet (Lond. Engl.).

[3] Davis MC, Broadwater DR, Amburgy JW (2015). The clinical significance and reliability of self-reported smoking status in patients with intracranial aneurysms: A review. Clin. Neurol. Neurosurg..

[4] Can A, Castro VM, Ozdemir YH (2017). Association of intracranial aneurysm rupture with smoking duration, intensity, and cessation. Neurology.

[5] Kang H, Peng T, Qian Z (2015). Impact of hypertension and smoking on the rupture of intracranial aneurysms and their joint effect. Neurol. Neurochir. Pol..

[6] Yao XY, Jiang CQ, Jia GL (2016). Diabetes mellitus and the risk of aneurysmal subarachnoid haemorrhage: A systematic review and meta-analysis of current evidence. J. Int. Med. Res..

[7] Cronin O, Walker PJ, Golledge J (2013). The association of obesity with abdominal aortic aneurysm presence and growth. Atherosclerosis.

[8] Hughes JD, Samarage M, Burrows AM (2015). Body mass index and aneurysmal subarachnoid hemorrhage: Decreasing mortality with increasing body mass index. World neurosurgery.

[9] Can A, Castro VM, Dligach D (2018). Lipid-lowering agents and high HDL (high-density lipoprotein) are inversely associated with intracranial aneurysm rupture. Stroke.

[10] Bennett DA, Holmes MV (2017). Mendelian randomisation in cardiovascular research: An introduction for clinicians. Heart (Br. Cardiac Soc.).

[11] van’t Hof FN, Vaucher J, Holmes MV (2017). Genetic variants associated with type 2 diabetes and adiposity and risk of intracranial and abdominal aortic aneurysms. Eur. J. Hum. Genet. EJHG.

[12] Acosta JN, Szejko N, Both CP (2021). Genetically determined smoking behavior and risk of nontraumatic subarachnoid hemorrhage. Stroke.

[13] Bowden J, Davey Smith G, Burgess S (2015). Mendelian randomization with invalid instruments: Effect estimation and bias detection through Egger regression. Int. J. Epidemiol..

[14] Davey Smith G, Hemani G (2014). Mendelian randomization: Genetic anchors for causal inference in epidemiological studies. Hum. Mol. Genet..

[15] Taylor AE, Davies NM, Ware JJ (2014). Mendelian randomization in health research: Using appropriate genetic variants and avoiding biased estimates. Econ. Hum. Biol..

[16] Bakker MK, van der Spek RAA, van Rheenen W (2020). Genome-wide association study of intracranial aneurysms identifies 17 risk loci and genetic overlap with clinical risk factors. Nat. Genet..

[17] Kang HG, Kim BJ, Lee J (2015). Risk factors associated with the presence of unruptured intracranial aneurysms. Stroke.

[18] Yan T, Chopp M, Ning R (2013). Intracranial aneurysm formation in type-one diabetes rats. PLoS ONE.

[19] Gu YX, Chen XC, Song DL (2006). Risk factors for intracranial aneurysm in a Chinese ethnic population. Chin. Med. J..

[20] Raffort J, Lareyre F, Clément M (2018). Diabetes and aortic aneurysm: Current state of the art. Cardiovasc. Res..

[21] Tamura T, Jamous MA, Kitazato KT (2009). Endothelial damage due to impaired nitric oxide bioavailability triggers cerebral aneurysm formation in female rats. J. Hypertens..

[22] Shikata F, Shimada K, Sato H (2019). Potential influences of gut microbiota on the formation of intracranial aneurysm. Hypertension (Dallas, Tex : 1979).

[23] Lindbohm JV, Rautalin I, Jousilahti P (2019). Physical activity associates with subarachnoid hemorrhage risk—a population-based long-term cohort study. Sci. Rep..

[24] Burgess S, Thompson SG (2011). Avoiding bias from weak instruments in Mendelian randomization studies. Int. J. Epidemiol..

[25] Pierce BL, Burgess S (2013). Efficient design for Mendelian randomization studies: Subsample and 2-sample instrumental variable estimators. Am. J. Epidemiol..

