# Supplementary material for: A two-sample Mendelian randomization analysis of modifiable risk factors and intracranial aneurysms
Source: Sci Rep. 2022 May 10;12:7659. doi: 10.1038/s41598-022-11720-9 (PMC9091241; doi:10.1038/s41598-022-11720-9)
Supplement: Supplementary file 1 — Supplementary Information 1. [file 41598_2022_11720_MOESM1_ESM.pdf]

Supplement figure 1: Flowchart for this MR analysis

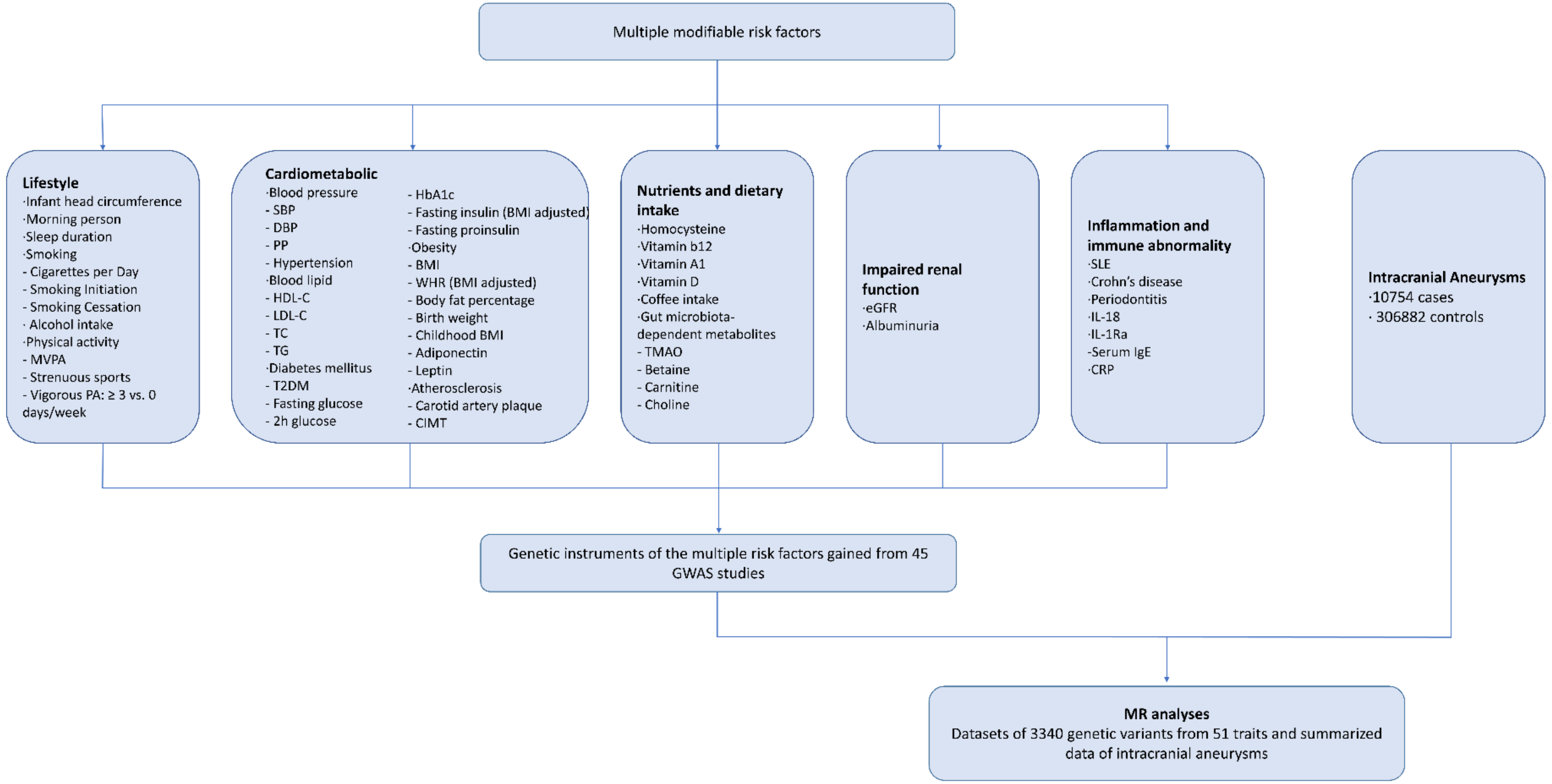

BMI: body mass index; CIMT: carotid intima media thickness; CRP: C reactive protein; MVPA: moderate-to-vigorous physical activity; Strenuous sports: Strenuous sports or other exercises: ≥ 2-3 vs. 0 days/week; SBP: systolic blood pressure; DBP: diastolic blood pressure; PP: pulse pressure; HDL-C: high-density lipoprotein cholesterol; LDL-C: low-density lipoprotein cholesterol; TC: total cholesterol; TG: triglyceride; T2DM: type 2 diabetes mellitus; HbA1c: hemoglobin A1c; WHR: waist hip ratio; TMAO: trimethylamine-n-oxide; SLE: systemic lupus erythematosus; eGFR: estimated glomerular filtration rate; IL: interleukin; GWAS: genome wide association study; MR: Mendelian randomization.

Supplement figure 2: Scatter plot of influence of smoking initiation on intracranial aneurysms.

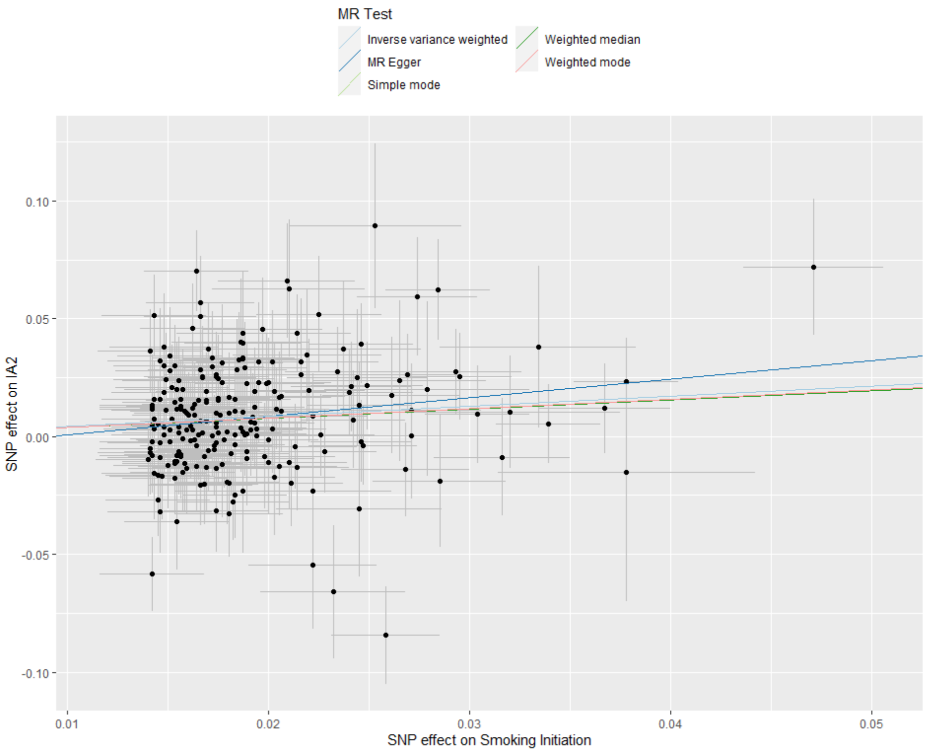

SNP: single nucleotide polymorphism; IA: intracranial aneurysm

Supplement figure 3: Scatter plot of influence of cigarettes intake on intracranial aneurysms.

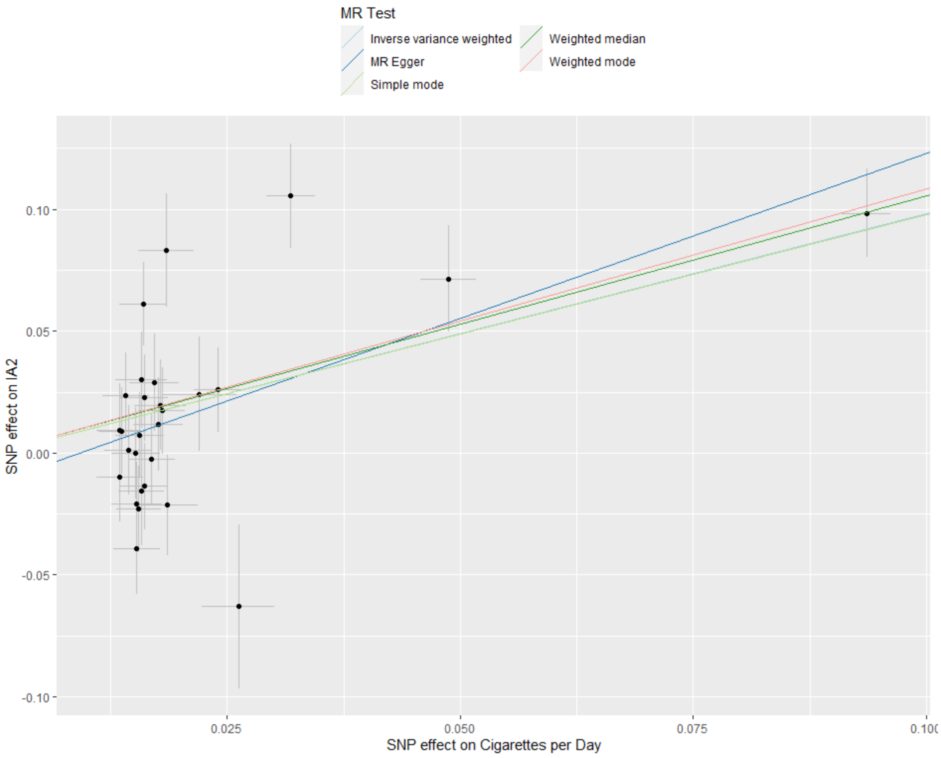

SNP: single nucleotide polymorphism; IA: intracranial aneurysm

Supplement figure 4: Scatter plot of influence of SBP on intracranial aneurysms.

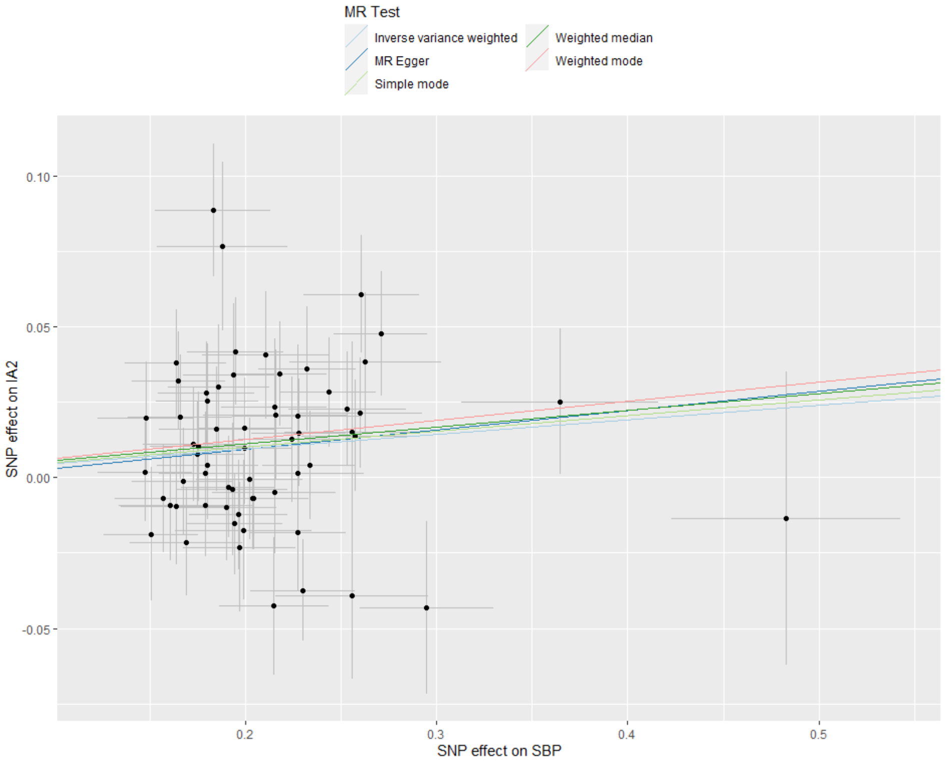

SNP: single nucleotide polymorphism; SBP: systolic blood pressure; IA: intracranial aneurysm

Supplement figure 5: Scatter plot of influence of hypertension on intracranial aneurysms.

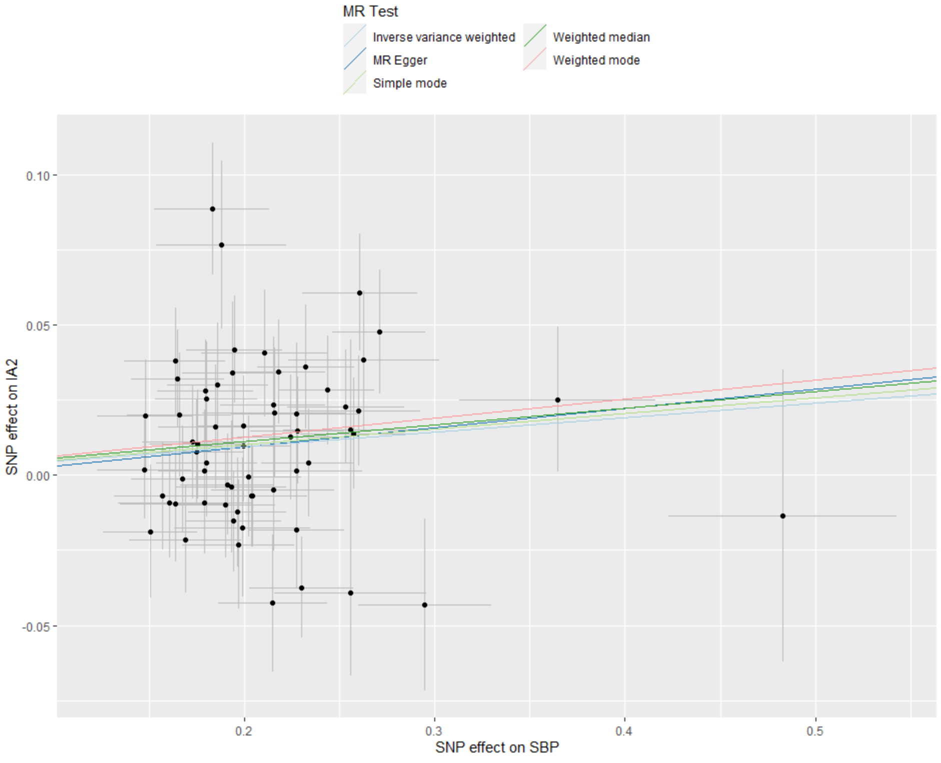

SNP: single nucleotide polymorphism; IA: intracranial aneurysm

Supplement figure 6: Scatter plot of influence of T2DM on intracranial aneurysms.

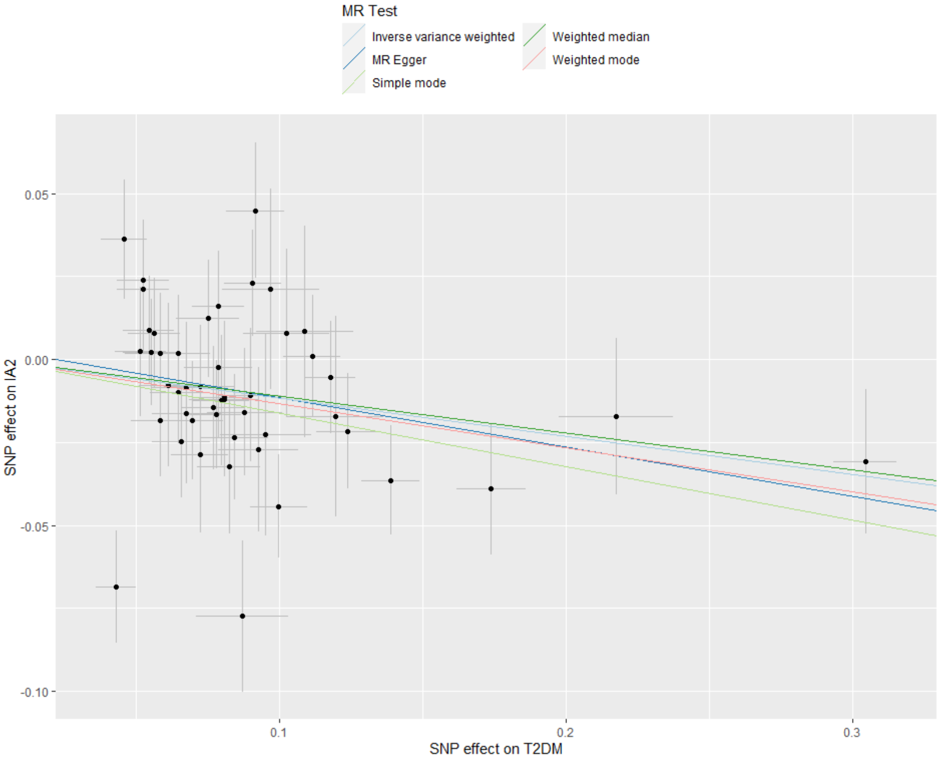

SNP: single nucleotide polymorphism; T2DM: type 2 diabetes mellitus; IA: intracranial aneurysm

Supplement figure 7: Scatter plot of influence of body fat percentage on intracranial aneurysms.

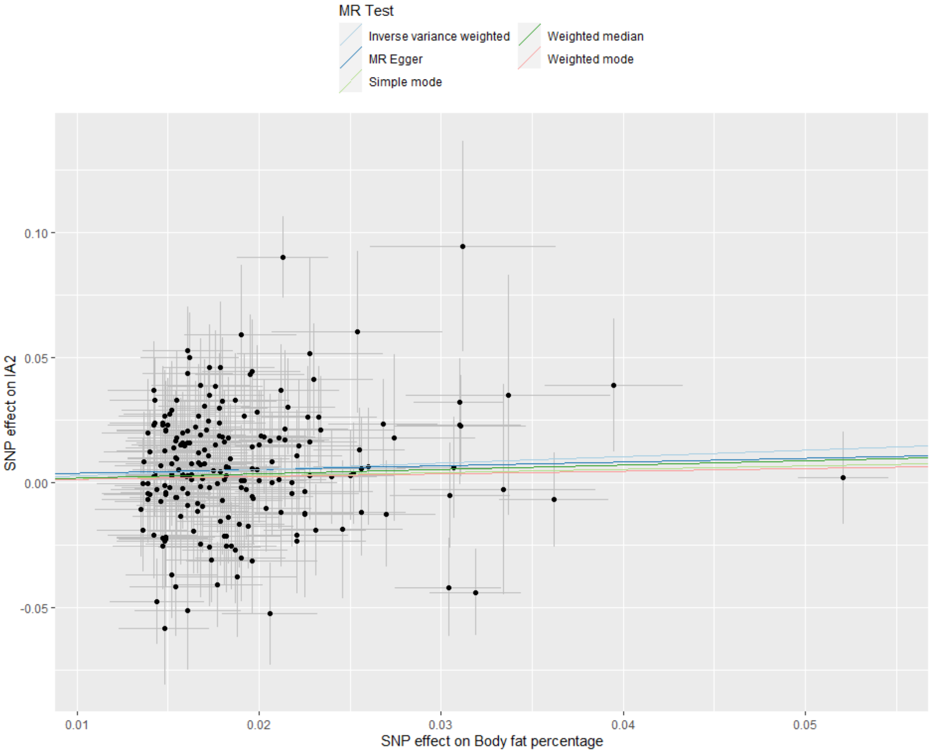

SNP: single nucleotide polymorphism; IA: intracranial aneurysm

Supplement figure 8: Scatter plot of influence of vigorous physical activity on intracranial aneurysms.

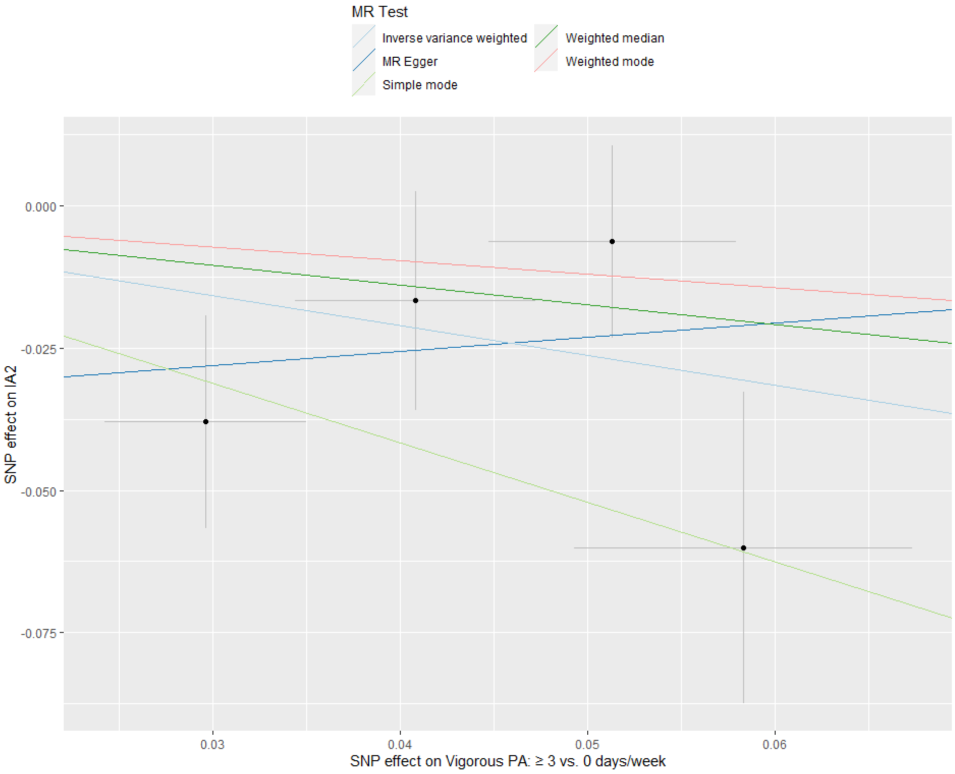

SNP: single nucleotide polymorphism; PA: physical activity; IA: intracranial aneurysm

Supplement figure 9: Scatter plot of influence of total cholesterol on intracranial aneurysms.

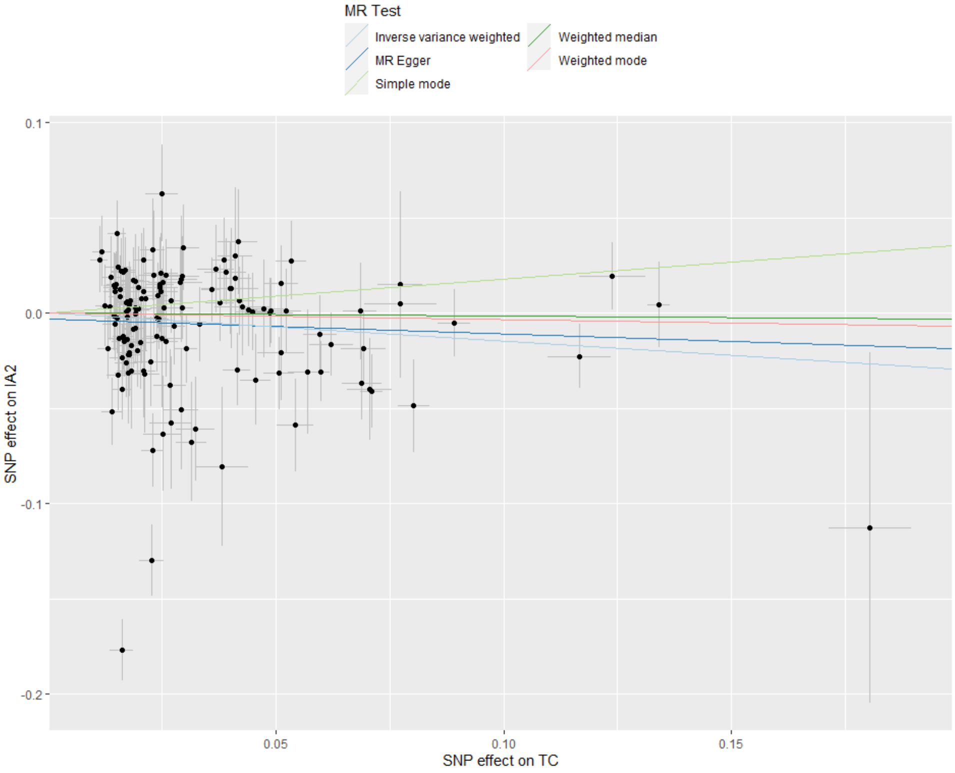

SNP: single nucleotide polymorphism; IA: intracranial aneurysm

Supplement figure 10: Scatter plot of influence of fasting glucose on intracranial aneurysms.

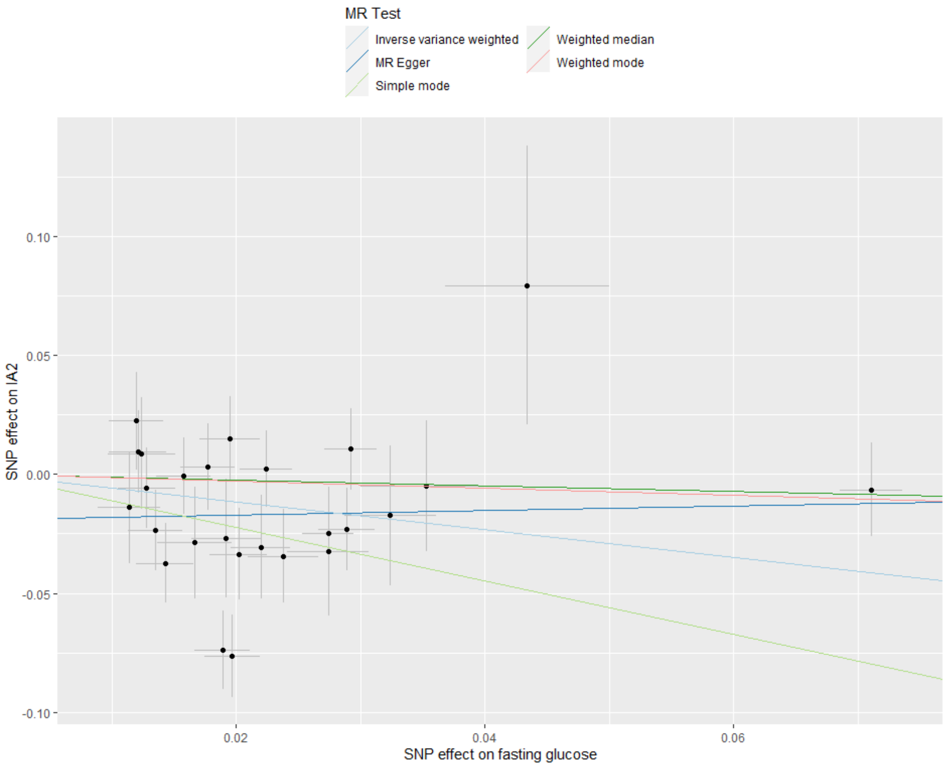

SNP: single nucleotide polymorphism; IA: intracranial aneurysm

Supplement figure 11: Scatter plot of influence of HbA1c on intracranial aneurysms.

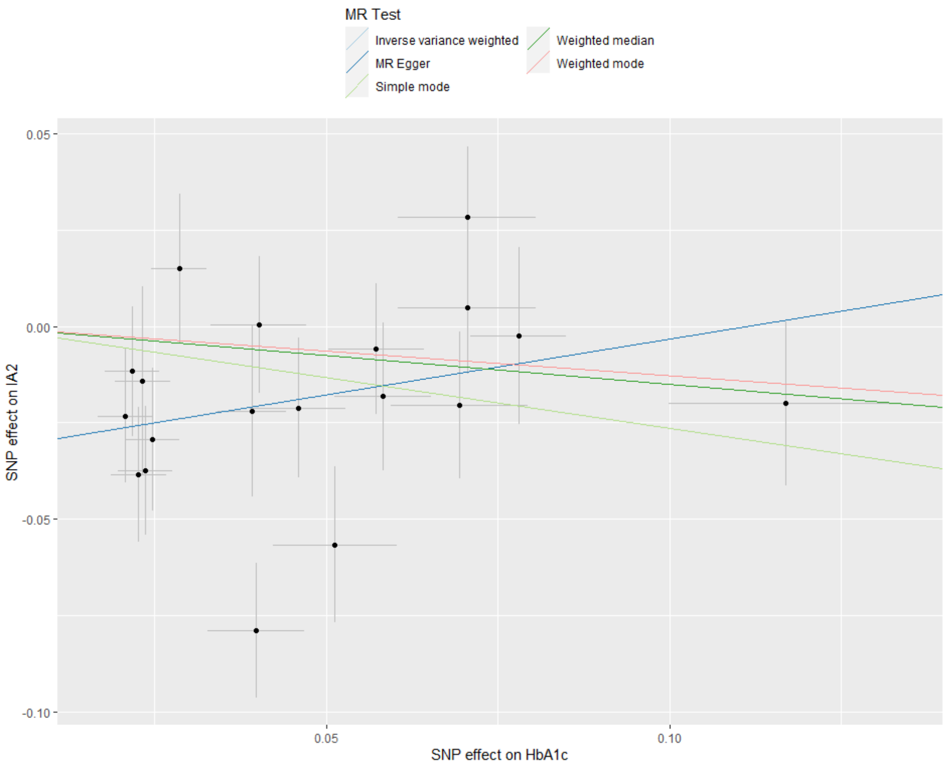

SNP: single nucleotide polymorphism; HbA1c: hemoglobin A1c; IA: intracranial aneurysm

Supplement figure 12: Scatter plot of influence of betaine on intracranial aneurysms.

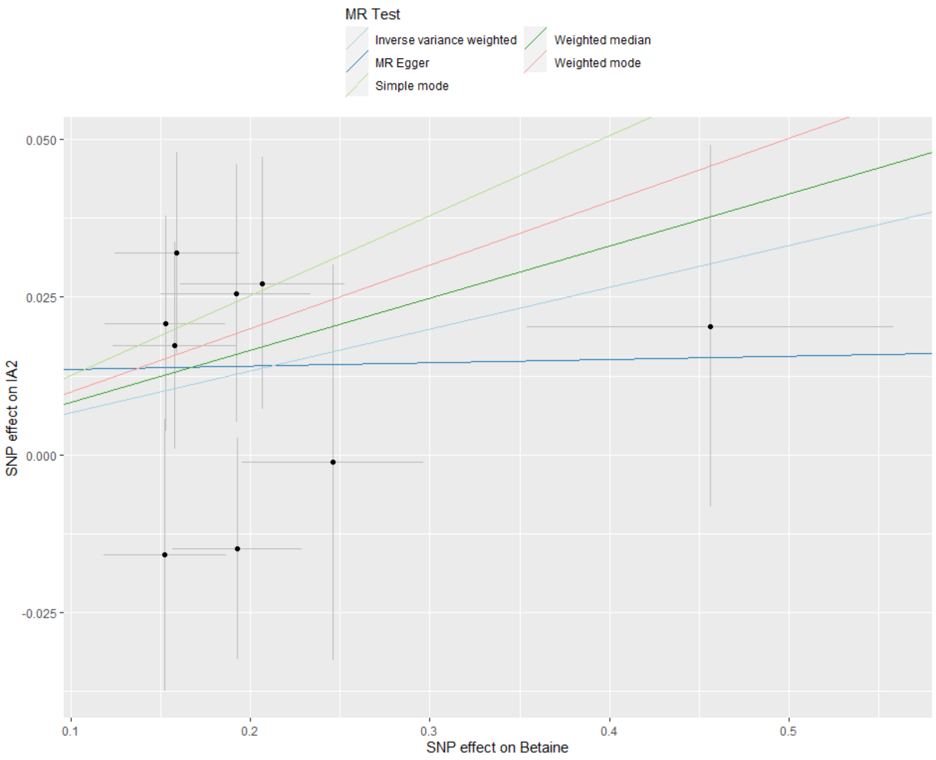

SNP: single nucleotide polymorphism; IA: intracranial aneurysm

Supplement figure 13: Scatter plot of influence of carnitine on intracranial aneurysms.

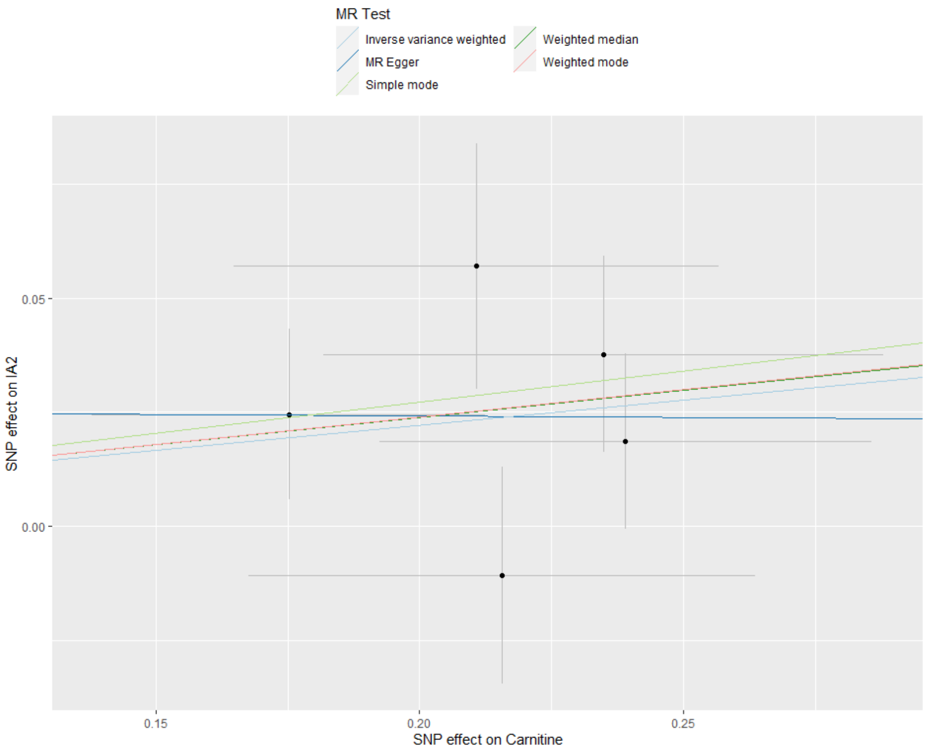

SNP: single nucleotide polymorphism; IA: intracranial aneurysm

Supplemental methods

Summarized data of GWAS study of intracranial aneurysms.

Study	Mark K Bakker, et. al.,2020
Study design	Meta-analysis of cohort studies
n	10754 cases, 306882 controls
Female	55%
Populations	European and East Asian ancestries
Cohorts included	@neurIST, ARIC, Busselton, Utrecht 1, Netherlands (EGA), Utrecht 2, Doetinchem Cohort Study, Project MinE, French Canadian, Finland (EGA), Finland, NFBC1966, ICAN, PREGO, GAIN, FIA, nonGAIN, Poland, NBS, UK Biobank, GOSH controls GOSH cases, NBS+1958BBC, the BioBank Japan (BBJ), the China Kadoorie Biobank (CKB).
Data collection	Both ruptured (thus with aSAH) and unruptured intracranial aneurysms confirmed using imaging are included.
Phenotype exclusions	Patients with conditions known to predispose to intracranial aneurysms, including autosomal dominant polycystic kidney disease, Ehlers–Danlos disease and Marfan's syndrome, were excluded.
Control selection	All controls were unselected controls. Controls were matched by genotyping platform and country at the cohort level.
Genotyping arrays	Illumina 550, Illumina 660, Affymetrix 6.0, Illumina CNV370-duo, Illumina GSA, Illlumina 2.5M, Illumina NeuroX, Affymetrix PMRA, Affymetrix Axiom, UK Biobank Axiom Array
SNP scaffold quality control	• Excluded SNPs with >10% missing data as an early outlier removal.<br>• Removed samples with >15% missing data as an early outlier removal.<br>• Included only autosomal SNPs.<br>• Excluded high LD regions LCT (chr2:129883539-140283530), MHC (chr6:24092021-38892022), chr8 inversion (chr8:6612592-13455629) and chr17 inversion (chr17:40546474-44644684).<br>• Excluded SNPs with >1% missing data.<br>• Removed samples with >2% missing data.<br>• Excluded SNPs with minor allele count < 10.<br>• Excluded heterozygosity outliers, by calculating F-statistic using plink --het and selecting upper and lower threshold by visual inspection.<br>• Removed duplicate samples as defined by pi(hat) > 0.80, calculated using plink --genome.<br>• Excluded SNPs with haplotype-biased missingness P-value < 1·10-5 , 1·10-10, or 1·10-15, depending on the samples size. This was calculated using plink –test-mishap, but only if both tested haplotypes had a frequency > 0.02.<br>• Excluded populations outliers by calculating principal components (PCs) projected on the HapMap3 dataset. Mean coordinate of all European ancestry HapMap3 samples was used to define European ancestry. Samples with a set number of SDs away from the European center in any of the first

	4 PCs were removed. The number of SDs was defined by visual inspection, and ranged from 4 to 12 per cohort.<br>• Excluded cohort outliers by calculating PCs within the cohort. Samples outside a set number of SD from the mean in any of the first 10 PCs were excluded. The number of SD was defined by visual inspection.<br>• Excluded SNPs with a large minor allele frequency (MAF) difference between any pair of cohorts. This was calculated as follows between each pair of cohorts. Define the error of a SNP as the square of the difference between allele frequencies of the two cohorts. Calculate a normalized error by dividing the error by the mean MAF for that SNP. Do this for every SNP in common between the two cohorts, and calculate the mean and SD of the normalized error terms for all SNPs. SNPs with a normalized error more than 10 SDs away from the mean are excluded, but only if the error is greater than 0.0025.<br>• Ran the HRC checking tool v4.2 (HRC-1000G-check-bim-NoReadKey.pl) [https://www.well.ox.ac.uk/~wrayner/tools/] to identify mismatched SNP alleles or position compared to the haplotype reference consortium (HRC) reference dataset.
