# Supplementary material for: A two-sample Mendelian randomization analysis of modifiable risk factors and intracranial aneurysms
Source: Sci Rep. 2022 May 10;12:7659. doi: 10.1038/s41598-022-11720-9 (PMC9091241; doi:10.1038/s41598-022-11720-9)
Supplement: Supplementary file 2 — Supplementary Information 2. [file 41598_2022_11720_MOESM2_ESM.docx]

| INDEX |
| --- |
| Supplement Table 1: Genome- wide association studies and number of single nucleotide  polymorphisms used as instrumental variable in the present Mendelian randomization. |
| Supplement Table 2: Characteristics of selected genetic variants. |
| Supplement Table 3: Results for four Mendelian randomization methods for influence of  mutiple risk factors on intracranial aneurysms. |
| Supplement Table 4: Effect allele, effect on exposure and strength of association with  intracranial aneurysms(TRANS) for SNPs used as instrumental variables. |
| Supplement Table 5: Genetic variants that are related with mutiple traits. |
| Supplement Table 6: MR estimates for the effect of each risk factor on IA using IVW for  sensitivity analyses.  Supplement Table 7: Multivariable MR analysis of exposures which are significantly associated with IA in two-sample MR and IA. |

| Supplement Table 1: Genome- wide association studies and number of single nucleotide polymorphisms used as instrumental  variable in the present Mendelian randomization. | | | | | | |
| --- | --- | --- | --- | --- | --- | --- |
| **Risk factors(ethnic)** | **Published GWAS of the modifiable risk factors** | | | | **Present MR analysis** | |
|  | **PMID** | **Sample size** | **No. of**  **independent significant** | **R2 (%)**  **explained by the SNPs** | **Power** | **F statistic**  **(instrumental strength)** |
| Lifestyle | | | | | | |
| Infant head circumference (EU) | 31681408 | 182814 | 5 | 0.003181 | 0.05 | 1014.62 |
| Morning person (EU) | 26835600 | 89283 | 15 | 0.006608 | 0.09 | 2113.90 |
| Sleep duration (EU) | 30846698 | 446118 | 78 | 0.006921 | 0.05 | 2214.68 |
| Cigarettes per day (Multi-Ethnic) | 30643251 | 335394 | 55 | 0.014009 | 1.00 | 4513.99 |
| Smoking initiation (Multi-Ethnic) | 30643251 | 1232091 | 378 | 0.013739 | 1.00 | 4425.79 |
| Smoking cessation (Multi-Ethnic) | 30643251 | 547219 | 24 | 0.00243 | 0.63 | 774.74 |
| Alcohol intake (Multi-Ethnic) | 30643251 | 939356 | 95 | 0.006326 | 0.65 | 2023.16 |
| MVPA (EU) | 29899525 | 377234 | 9 | 0.00085 | 0.44 | 271.22 |
| Strenuous sports (EU) | 29899525 | 350492 | 6 | 0.000777 | 0.08 | 248.00 |
| Vigorous PA (EU) | 29899525 | 261055 | 5 | 0.000798 | 0.22 | 254.68 |
| Cardiometabolic | | | | | | |
| SBP (EU) | 30224653 | 757601 | 130 | 0.009509 | 0.08 | 3050.40 |
| DBP (EU) | 30224653 | 757601 | 91 | 0.006745 | 0.08 | 2158.00 |
| PP (EU) | 30224653 | 757601 | 104 | 0.008024 | 0.08 | 2570.33 |
| Hypertension (EU) | 21909115 | 203056 | 11 | 0.010378 | 1.00 | 3332.00 |
| HDL-C (EU, AA, Hispanic) | 30275531 | 312571 | 312 | 0.12646 | 0.72 | 45984.30 |
| LDL-C (EU, AA, Hispanic) | 30275531 | 312571 | 219 | 0.097957 | 0.94 | 34494.55 |
| TC (EU, AA, Hispanic) | 30275531 | 312571 | 226 | 0.086272 | 1.00 | 29991.43 |
| TG (EU, AA, Hispanic) | 30275531 | 312571 | 253 | 0.085247 | 0.66 | 29601.90 |
| T2DM (EU, EA, SA, MA, MAA) | 24509480/  28869590 | 187590/265678 | 69 | 0.009222 | 0.19 | 2957.50 |
| Fasting glucose (EU) | 22885924 | 133010 | 37 | 0.031934 | 1.00 | 10478.99 |
| 2 h glucose (EU) | 22885924 | 131010 | 7 | 0.002326 | 0.06 | 741.54 |
| HbA1c (EU, AA, EA, SA) | 28898252/  29403010 | 20838 (east Asia)/  42790 (Japanese) | 27 | 0.019512 | 0.91 | 6322.05 |
| Fasting insulin (BMI adjusted) (EU) | 22885924 | 108557 | 14 | 0.005688 | 0.05 | 1818.05 |
| Fasting proinsulin (EU) | 21873549 | 27079 | 9 | 0.02566 | 0.37 | 8366.19 |
| BMI (Multi-Ethnic) | 25673413 | 339224 | 96 | 0.016041 | 0.05 | 5179.26 |
| WHR (BMI adjusted) (EU, EA, SA, AA) | 25673412 | 224459 | 49 | 0.010778 | 0.07 | 3461.78 |
| Body fat percentage (EU) | 31665421 | NA | 370 | 0.078243 | 1.00 | 26963.41 |
| Birth weight (EU, AA, Chinese, Filipino, Surinamese, Turkish and Moroccan) | 27680694 | 153781 | 57 | 0.017826 | 0.16 | 5765.95 |
| Childhood BMI (EU) | 26604143 | 47541 | 15 | 0.01714 | 0.36 | 5540.22 |
| Adiponectin (EU, AA, Asia) | 22479202 | 37638 | 7 | 0.014948 | 0.12 | 4821.07 |
| Leptin (white European, Caucasian) | 26833098 | 51450 | 5 | 0.00344 | 1.00 | 1097.44 |
| Carotid artery plaque (EU) | 21909108 | 29255 | 2 | 0.003209 | 0.65 | 1023.58 |
| CIMT (EU) | 21909108 | 41295 | 3 | 0.002524 | 0.89 | 804.74 |
| Nutrients and dietary intake | | | | | | |
| Homocysteine (EU) | 23824729 | 44147 | 18 | 0.03538 | 0.33 | 11651.14 |
| Vitamin B12 (EU) | 19744961 | 3340 | 7 | 0.060215 | 0.85 | 20352.94 |
| Vitamin A1 (Caucasian) | 21878437 | 5006 | 2 | 0.013742 | 0.06 | 4426.77 |
| Vitamin D (EU) | 29343764 | 79366 | 6 | 0.026727 | 0.08 | 8723.59 |
| Coffee intake (EU) | 25288136 | 91462 | 10 | 0.007545 | 0.09 | 2415.78 |
| TMAO (EU) | 23823483 | 2076 | 9 | 0.096496 | 0.35 | 33925.15 |
| Betaine (EU) | 23823483 | 2076 | 15 | 0.154172 | 0.80 | 57897.61 |
| Carnitine (EU) | 23823483 | 2076 | 12 | 0.170887 | 1.00 | 65468.39 |
| Choline (EU) | 23823483 | 2076 | 8 | 0.079826 | 0.21 | 27556.24 |
| Impaired renal function | | | | | | |
| eGFR (Multi-Ethnic) | 31152163 | 783978 | 308 | 0.028285 | 0.98 | 9246.85 |
| Albuminuria (EU) | 30220432 | 382500 | 33 | 0.006651 | 1.00 | 2127.74 |
| Inflammation and immune abnormality | | | | | | |
| SLE (EU) | 29848360 | 10995 | 5 | 0.020967 | 0.06 | 6803.50 |
| Crohn’s disease (Multi-Ethnic) | 21102463 | 51523 | 62 | 0.061498 | 0.08 | 20815.00 |
| Periodontitis (EU) | 29346566/  30218097 | 8000/15003 | 4 | 0.012849 | 0.11 | 4135.43 |
| Interleukin-18 (EU) | 24182552 | 5888 | 16 | 0.182155 | 0.99 | 70746.66 |
| Interleukin-1Ra (EU) | 24182552 | 5888 | 3 | 0.079702 | 0.30 | 27509.72 |
| CRP (EU) | 30388399 | 352566 | 40 | 0.004893 | 0.06 | 1562.84 |

GWAS: genome wide association study; MR: Mendelian randomization; MVPA: moderate-to-vigorous physical activity; Strenuous sports: Strenuous sports or other exercises: ≥ 2-3 vs. 0 days/week; vigorous PA: ≥3 vs. 0 days/week; SBP: systolic blood pressure; DBP: diastolic blood pressure; PP: pulse pressure; HDL-C: high-density lipoprotein cholesterol; LDL-C: low-density lipoprotein cholesterol; TC: total cholesterol; TG: triglyceride; T2DM: type 2 diabetes mellitus; HbA1c: hemoglobin A1c; BMI: body mass index; WHR: waist hip ratio; CIMT: carotid intima media thickness; TMAO: trimethylamine-n-oxide; SLE: systemic lupus erythematosus; CRP: C reactive protein; eGFR: estimated glomerular filtration rate; EU: European ancestry; AA: African ancestry; EA: East Asia; SA: South Asia; MA: Mexican ancestry; MAA: Mexican American ancestry

| Supplement Table 2: Characteristics of selected genetic variants. | | | | | | | |
| --- | --- | --- | --- | --- | --- | --- | --- |
| **SNP** | **Traits** | **Ethnics** | **RA** | **EAF** | **BETA** | **SE** | **P-value** |
| rs6975024 | 2h glucose | EUP | C | 0.15 | 0.1026 | 0.0156 | 5.25E-11 |
| rs11782386 | 2h glucose | EUP | C | 0.87 | 0.0985 | 0.0165 | 2.15E-09 |
| rs1019503 | 2h glucose | EUP | A | 0.48 | 0.0628 | 0.0109 | 8.87E-09 |
| rs11717195 | 2h glucose | EUP | T | 0.79 | 0.0904 | 0.0135 | 1.87E-11 |
| rs2877716 | 2h glucose | EUP | C | 0.77 | 0.0805 | 0.0143 | 1.70E-08 |
| rs12255372 | 2h glucose | EUP | T | 0.24 | 0.0916 | 0.0131 | 2.88E-12 |
| rs11672660 | 2h glucose | EUP | T | 0.22 | 0.1182 | 0.0144 | 2.40E-16 |
| rs1780813 | Systemic Lupus Erythematosus | EUP | C | 0.02 | -0.7035 | 0.2386 | 3.50E-08 |
| rs55849330 | Systemic Lupus Erythematosus | EUP | A | 0.29 | 0.1475 | 0.0220 | 4.90E-08 |
| rs150518861 | Systemic Lupus Erythematosus | EUP | A | 0.01 | 0.5043 | 0.0538 | 4.10E-08 |
| rs114038709 | Systemic Lupus Erythematosus | EUP | T | NA | 0.1516 | 0.0241 | 3.70E-08 |
| rs36023980 | Systemic Lupus Erythematosus | EUP | C | 0.75 | 0.1687 | 0.0237 | 4.70E-09 |
| rs7556815 | Sleep duration | EUP | A | 0.22 | 2.4430 | 0.1644 | 1.30E-49 |
| rs75539574 | Sleep duration | EUP | C | 0.09 | 2.1750 | 0.2439 | 6.90E-19 |
| rs12607679 | Sleep duration | EUP | T | 0.74 | 1.2080 | 0.1556 | 8.30E-15 |
| rs915416 | Sleep duration | EUP | C | 0.29 | 1.1560 | 0.1497 | 9.90E-15 |
| rs9940646 | Sleep duration | EUP | C | 0.58 | 1.0170 | 0.1374 | 1.20E-13 |
| rs13109404 | Sleep duration | EUP | T | 0.93 | 1.8720 | 0.2645 | 1.40E-12 |
| rs8050478 | Sleep duration | EUP | G | 0.50 | 0.9600 | 0.1359 | 1.70E-12 |
| rs56372231 | Sleep duration | EUP | T | 0.33 | 1.0170 | 0.1440 | 2.20E-12 |
| rs13088093 | Sleep duration | EUP | G | 0.34 | 0.9760 | 0.1441 | 7.00E-12 |
| rs2079070 | Sleep duration | EUP | C | 0.27 | 1.0530 | 0.1540 | 7.50E-12 |
| rs34556183 | Sleep duration | EUP | A | 0.72 | 1.0150 | 0.1514 | 2.30E-11 |
| rs3095508 | Sleep duration | EUP | C | 0.59 | 0.9210 | 0.1383 | 3.10E-11 |
| rs34731055 | Sleep duration | EUP | T | 0.18 | 1.1680 | 0.1769 | 3.70E-11 |
| rs73219758 | Sleep duration | EUP | G | 0.71 | 0.9840 | 0.1497 | 5.60E-11 |
| rs10973207 | Sleep duration | EUP | T | 0.16 | 1.2260 | 0.1874 | 6.00E-11 |
| rs2139261 | Sleep duration | EUP | G | 0.75 | 1.1220 | 0.1739 | 8.50E-11 |
| rs4592416 | Sleep duration | EUP | G | 0.46 | 0.8810 | 0.1362 | 9.30E-11 |
| rs365663 | Sleep duration | EUP | A | 0.55 | 0.8780 | 0.1367 | 1.00E-10 |
| rs1517572 | Sleep duration | EUP | C | 0.58 | 0.8790 | 0.1377 | 1.50E-10 |
| rs7915425 | Sleep duration | EUP | T | 0.18 | 1.1440 | 0.1794 | 2.00E-10 |
| rs330088 | Sleep duration | EUP | C | 0.55 | 0.8680 | 0.1366 | 2.70E-10 |
| rs8038326 | Sleep duration | EUP | A | 0.73 | 0.9550 | 0.1524 | 2.80E-10 |
| rs460692 | Sleep duration | EUP | C | 0.14 | 1.2630 | 0.1999 | 3.60E-10 |
| rs9382445 | Sleep duration | EUP | T | 0.62 | 0.8720 | 0.1400 | 4.80E-10 |
| rs4767550 | Sleep duration | EUP | G | 0.41 | 0.8580 | 0.1386 | 6.30E-10 |
| rs11885663 | Sleep duration | EUP | T | 0.25 | 0.9730 | 0.1571 | 8.60E-10 |
| rs1991556 | Sleep duration | EUP | G | 0.77 | 0.9940 | 0.1634 | 1.00E-09 |
| rs1057703 | Sleep duration | EUP | G | 0.15 | 1.1640 | 0.1924 | 1.10E-09 |
| rs4128364 | Sleep duration | EUP | C | 0.34 | 0.8760 | 0.1435 | 1.40E-09 |
| rs61796569 | Sleep duration | EUP | T | 0.27 | 0.9270 | 0.1539 | 1.50E-09 |
| rs10483350 | Sleep duration | EUP | G | 0.20 | 1.0420 | 0.1721 | 1.50E-09 |
| rs7115226 | Sleep duration | EUP | A | 0.07 | 1.5940 | 0.2615 | 1.70E-09 |
| rs269054 | Sleep duration | EUP | A | 0.42 | 0.8190 | 0.1376 | 2.10E-09 |
| rs112230981 | Sleep duration | EUP | A | 0.95 | 1.8920 | 0.3137 | 2.20E-09 |
| rs11602180 | Sleep duration | EUP | C | 0.84 | 1.0950 | 0.1839 | 2.30E-09 |
| rs2192528 | Sleep duration | EUP | A | 0.48 | 0.8020 | 0.1362 | 2.70E-09 |
| rs12246842 | Sleep duration | EUP | A | 0.46 | 0.8040 | 0.1365 | 3.90E-09 |
| rs205024 | Sleep duration | EUP | T | 0.38 | 0.8300 | 0.1396 | 3.90E-09 |
| rs12567114 | Sleep duration | EUP | A | 0.28 | 0.8900 | 0.1524 | 4.30E-09 |
| rs7616632 | Sleep duration | EUP | T | 0.52 | 0.7920 | 0.1362 | 4.30E-09 |
| rs6575005 | Sleep duration | EUP | T | 0.76 | 0.9340 | 0.1585 | 4.40E-09 |
| rs1776776 | Sleep duration | EUP | T | 0.87 | 1.1980 | 0.2046 | 4.90E-09 |
| rs11621908 | Sleep duration | EUP | C | 0.92 | 1.4460 | 0.2498 | 5.60E-09 |
| rs10421649 | Sleep duration | EUP | A | 0.56 | 0.7980 | 0.1377 | 6.90E-09 |

| rs2072727 | Sleep duration | EUP | T | 0.44 | 0.7950 | 0.1371 | 7.90E-09 |
| --- | --- | --- | --- | --- | --- | --- | --- |
| rs113113059 | Sleep duration | EUP | T | 0.78 | 0.9680 | 0.1642 | 8.40E-09 |
| rs374153 | Sleep duration | EUP | C | 0.16 | 1.0570 | 0.1862 | 9.10E-09 |
| rs151014368 | Sleep duration | EUP | A | 0.21 | 0.9660 | 0.1692 | 9.10E-09 |
| rs62120041 | Sleep duration | EUP | T | 0.93 | 1.5670 | 0.2745 | 9.60E-09 |
| rs7503199 | Sleep duration | EUP | C | 0.73 | 0.8850 | 0.1538 | 1.00E-08 |
| rs1939455 | Sleep duration | EUP | G | 0.88 | 1.2260 | 0.2137 | 1.20E-08 |
| rs7951019 | Sleep duration | EUP | G | 0.03 | 2.2130 | 0.3913 | 1.20E-08 |
| rs17732997 | Sleep duration | EUP | C | 0.57 | 0.7760 | 0.1373 | 1.20E-08 |
| rs61985058 | Sleep duration | EUP | T | 0.14 | 1.1160 | 0.1937 | 1.30E-08 |
| rs17427571 | Sleep duration | EUP | A | 0.68 | 0.8300 | 0.1461 | 1.30E-08 |
| rs7806045 | Sleep duration | EUP | T | 0.76 | 0.8870 | 0.1575 | 1.40E-08 |
| rs35531607 | Sleep duration | EUP | C | 0.47 | 0.7700 | 0.1364 | 1.50E-08 |
| rs7644809 | Sleep duration | EUP | T | 0.42 | 0.7840 | 0.1381 | 1.60E-08 |
| rs9345234 | Sleep duration | EUP | C | 0.58 | 0.7810 | 0.1379 | 1.80E-08 |
| rs12791153 | Sleep duration | EUP | T | 0.08 | 1.4130 | 0.2530 | 1.90E-08 |
| rs1263056 | Sleep duration | EUP | A | 0.52 | 0.7680 | 0.1366 | 2.00E-08 |
| rs55658675 | Sleep duration | EUP | C | 0.65 | 0.7880 | 0.1421 | 2.00E-08 |
| rs11567976 | Sleep duration | EUP | T | 0.57 | 0.7680 | 0.1371 | 2.10E-08 |
| rs180769 | Sleep duration | EUP | T | 0.43 | 0.7630 | 0.1376 | 2.30E-08 |
| rs1553132 | Sleep duration | EUP | G | 0.26 | 0.8700 | 0.1551 | 2.50E-08 |
| rs9903973 | Sleep duration | EUP | C | 0.47 | 0.7660 | 0.1363 | 2.60E-08 |
| rs11614986 | Sleep duration | EUP | A | 0.82 | 0.9830 | 0.1771 | 2.70E-08 |
| rs2231265 | Sleep duration | EUP | G | 0.77 | 0.8970 | 0.1620 | 2.70E-08 |
| rs174560 | Sleep duration | EUP | C | 0.31 | 0.8150 | 0.1462 | 2.80E-08 |
| rs10173260 | Sleep duration | EUP | C | 0.61 | 0.7700 | 0.1388 | 2.90E-08 |
| rs72804080 | Sleep duration | EUP | G | 0.15 | 1.0680 | 0.1918 | 2.90E-08 |
| rs12611523 | Sleep duration | EUP | A | 0.55 | 0.7580 | 0.1366 | 3.10E-08 |
| rs11643715 | Sleep duration | EUP | G | 0.29 | 0.8340 | 0.1498 | 3.20E-08 |
| rs4538155 | Sleep duration | EUP | T | 0.65 | 0.7790 | 0.1424 | 3.60E-08 |
| rs34354917 | Sleep duration | EUP | C | 0.71 | 0.8250 | 0.1500 | 3.90E-08 |
| rs80193650 | Sleep duration | EUP | G | 0.16 | 1.0100 | 0.1840 | 4.10E-08 |
| rs10761674 | Sleep duration | EUP | C | 0.48 | 0.7400 | 0.1360 | 4.20E-08 |
| rs11190970 | Sleep duration | EUP | G | 0.80 | 0.9230 | 0.1694 | 4.60E-08 |
| rs3755967 | Vitamin D level | EUP | T | 0.28 | -0.0890 | 0.0023 | 4.74E-343 |
| rs12785878 | Vitamin D level | EUP | T | 0.75 | 0.0360 | 0.0022 | 3.80E-62 |
| rs10741657 | Vitamin D level | EUP | A | 0.40 | 0.0310 | 0.0022 | 2.05E-46 |
| rs17216707 | Vitamin D level | EUP | T | 0.79 | 0.0260 | 0.0027 | 8.14E-23 |
| rs10745742 | Vitamin D level | EUP | T | 0.40 | 0.0170 | 0.0022 | 1.88E-14 |
| rs8018720 | Vitamin D level | EUP | C | 0.82 | -0.0170 | 0.0029 | 4.72E-09 |
| rs964184 | Vitamin D level | EUP | G | 0.15 | 0.0400 | 0.0100 | 7.80E-12 |
| rs2108622 | Vitamin D level | EUP | T | 0.21 | 0.0300 | 0.0100 | 1.40E-10 |
| rs11057830 | Vitamin D level | EUP | A | 0.15 | 0.0300 | 0.0100 | 8.20E-09 |
| rs11264100 | Cigarettes per Day | EUP | G | 0.88 | -0.0222 | 0.0037 | 2.22E-09 |
| rs2072659 | Cigarettes per Day | EUP | G | 0.10 | -0.0300 | 0.0041 | 2.51E-13 |
| rs34973462 | Cigarettes per Day | EUP | T | 0.33 | 0.0151 | 0.0026 | 5.85E-09 |
| rs7599488 | Cigarettes per Day | EUP | T | 0.44 | 0.0141 | 0.0025 | 8.95E-09 |
| rs78408772 | Cigarettes per Day | EUP | T | 0.10 | -0.0220 | 0.0040 | 4.51E-08 |
| rs10204824 | Cigarettes per Day | EUP | G | 0.64 | -0.0180 | 0.0025 | 1.35E-12 |
| rs2084533 | Cigarettes per Day | EUP | T | 0.32 | 0.0161 | 0.0026 | 6.53E-10 |
| rs7431710 | Cigarettes per Day | EUP | A | 0.65 | -0.0183 | 0.0026 | 1.04E-12 |
| rs2236951 | Cigarettes per Day | EUP | C | 0.20 | -0.0172 | 0.0030 | 1.59E-08 |
| rs699165 | Cigarettes per Day | EUP | G | 0.75 | 0.0161 | 0.0028 | 8.09E-09 |
| rs28813180 | Cigarettes per Day | EUP | A | 0.50 | -0.0155 | 0.0024 | 1.95E-10 |
| rs1024323 | Cigarettes per Day | EUP | T | 0.38 | -0.0144 | 0.0025 | 8.66E-09 |
| rs11940255 | Cigarettes per Day | EUP | A | 0.72 | -0.0172 | 0.0027 | 2.20E-10 |
| rs10454798 | Cigarettes per Day | EUP | T | 0.25 | 0.0158 | 0.0028 | 1.53E-08 |
| rs7766641 | Cigarettes per Day | EUP | A | 0.27 | -0.0173 | 0.0027 | 2.91E-10 |

| rs215600 | Cigarettes per Day | EUP | A | 0.65 | -0.0240 | 0.0025 | 4.02E-21 |
| --- | --- | --- | --- | --- | --- | --- | --- |
| rs62447179 | Cigarettes per Day | EUP | A | 0.30 | -0.0153 | 0.0027 | 9.68E-09 |
| rs2741351 | Cigarettes per Day | EUP | C | 0.83 | 0.0185 | 0.0032 | 8.80E-09 |
| rs73229090 | Cigarettes per Day | EUP | A | 0.11 | 0.0262 | 0.0039 | 1.14E-11 |
| rs13253502 | Cigarettes per Day | EUP | A | 0.41 | -0.0138 | 0.0025 | 2.31E-08 |
| rs4236926 | Cigarettes per Day | EUP | G | 0.77 | 0.0343 | 0.0029 | 7.66E-33 |
| rs790564 | Cigarettes per Day | EUP | C | 0.73 | -0.0176 | 0.0027 | 1.24E-10 |
| rs75596189 | Cigarettes per Day | EUP | T | 0.11 | 0.0358 | 0.0039 | 1.84E-20 |
| rs3025383 | Cigarettes per Day | EUP | C | 0.19 | -0.0314 | 0.0031 | 9.78E-24 |
| rs7951365 | Cigarettes per Day | EUP | C | 0.31 | 0.0178 | 0.0026 | 1.53E-11 |
| rs10742683 | Cigarettes per Day | EUP | A | 0.42 | -0.0135 | 0.0025 | 4.83E-08 |
| rs113001570 | Cigarettes per Day | EUP | T | 0.07 | 0.0298 | 0.0049 | 1.04E-09 |
| rs7125588 | Cigarettes per Day | EUP | G | 0.43 | -0.0169 | 0.0025 | 6.50E-12 |
| rs11846838 | Cigarettes per Day | EUP | A | 0.33 | 0.0152 | 0.0026 | 5.03E-09 |
| rs1115019 | Cigarettes per Day | EUP | C | 0.79 | -0.0179 | 0.0030 | 2.27E-09 |
| rs632811 | Cigarettes per Day | EUP | G | 0.33 | -0.0178 | 0.0028 | 1.67E-10 |
| rs4886550 | Cigarettes per Day | EUP | G | 0.29 | -0.0199 | 0.0034 | 4.58E-09 |
| rs12438181 | Cigarettes per Day | EUP | A | 0.22 | -0.0185 | 0.0030 | 4.97E-10 |
| rs10519203 | Cigarettes per Day | EUP | A | 0.66 | -0.0936 | 0.0026 | 3.12E-286 |
| rs28438420 | Cigarettes per Day | EUP | T | 0.55 | 0.0176 | 0.0025 | 1.25E-12 |
| rs72740955 | Cigarettes per Day | EUP | T | 0.34 | 0.0318 | 0.0026 | 2.42E-34 |
| rs146009840 | Cigarettes per Day | EUP | T | 0.34 | 0.0221 | 0.0026 | 2.00E-17 |
| rs28681284 | Cigarettes per Day | EUP | T | 0.21 | -0.0487 | 0.0030 | 2.10E-58 |
| rs8040868 | Cigarettes per Day | EUP | C | 0.40 | 0.0160 | 0.0025 | 1.79E-10 |
| rs3743063 | Cigarettes per Day | EUP | C | 0.56 | -0.0167 | 0.0025 | 1.53E-11 |
| rs182317 | Cigarettes per Day | EUP | T | 0.36 | -0.0156 | 0.0026 | 1.31E-09 |
| rs1592485 | Cigarettes per Day | EUP | A | 0.61 | -0.0162 | 0.0025 | 1.11E-10 |
| rs12924872 | Cigarettes per Day | EUP | T | 0.46 | -0.0134 | 0.0024 | 4.39E-08 |
| rs258321 | Cigarettes per Day | EUP | G | 0.43 | 0.0158 | 0.0025 | 1.53E-10 |
| rs4144686 | Cigarettes per Day | EUP | A | 0.17 | -0.0186 | 0.0033 | 1.35E-08 |
| rs4485470 | Cigarettes per Day | EUP | A | 0.59 | -0.0153 | 0.0025 | 7.05E-10 |
| rs59208569 | Cigarettes per Day | EUP | C | 0.83 | 0.0205 | 0.0032 | 2.45E-10 |
| rs143200968 | Cigarettes per Day | EUP | C | 0.03 | -0.0861 | 0.0079 | 6.97E-28 |
| rs56113850 | Cigarettes per Day | EUP | C | 0.56 | 0.0523 | 0.0025 | 4.01E-99 |
| rs8192726 | Cigarettes per Day | EUP | A | 0.07 | -0.0393 | 0.0049 | 8.35E-16 |
| rs117824460 | Cigarettes per Day | EUP | G | 0.03 | -0.0953 | 0.0077 | 7.66E-35 |
| rs6078373 | Cigarettes per Day | EUP | A | 0.40 | 0.0161 | 0.0025 | 9.40E-11 |
| rs1737894 | Cigarettes per Day | EUP | G | 0.41 | 0.0169 | 0.0025 | 9.90E-12 |
| rs2273500 | Cigarettes per Day | EUP | C | 0.15 | 0.0364 | 0.0034 | 3.49E-26 |
| rs7281463 | Cigarettes per Day | EUP | C | 0.41 | 0.0137 | 0.0025 | 3.15E-08 |
| rs112187834 | Smoking Cessation | EUP | A | 0.14 | 0.0334 | 0.0056 | 2.81E-09 |
| rs7617480 | Smoking Cessation | EUP | C | 0.77 | -0.0329 | 0.0047 | 1.68E-12 |
| rs12203592 | Smoking Cessation | EUP | T | 0.18 | -0.0292 | 0.0051 | 1.21E-08 |
| rs707968 | Smoking Cessation | EUP | G | 0.68 | 0.0233 | 0.0042 | 2.76E-08 |
| rs7778443 | Smoking Cessation | EUP | C | 0.62 | -0.0230 | 0.0040 | 1.04E-08 |
| rs1565735 | Smoking Cessation | EUP | A | 0.20 | -0.0346 | 0.0049 | 1.54E-12 |
| rs60749569 | Smoking Cessation | EUP | T | 0.08 | -0.0401 | 0.0072 | 2.68E-08 |
| rs12378015 | Smoking Cessation | EUP | A | 0.30 | -0.0277 | 0.0043 | 8.31E-11 |
| rs9409844 | Smoking Cessation | EUP | A | 0.05 | -0.0586 | 0.0094 | 4.37E-10 |
| rs3025327 | Smoking Cessation | EUP | C | 0.11 | 0.0786 | 0.0063 | 1.19E-35 |
| rs10821523 | Smoking Cessation | EUP | C | 0.54 | 0.0262 | 0.0039 | 2.28E-11 |
| rs1611124 | Smoking Cessation | EUP | T | 0.07 | -0.0453 | 0.0078 | 5.26E-09 |
| rs7109376 | Smoking Cessation | EUP | A | 0.28 | 0.0281 | 0.0044 | 1.14E-10 |
| rs591143 | Smoking Cessation | EUP | T | 0.59 | -0.0243 | 0.0040 | 1.14E-09 |
| rs3866543 | Smoking Cessation | EUP | G | 0.52 | 0.0222 | 0.0039 | 1.35E-08 |
| rs518425 | Smoking Cessation | EUP | G | 0.29 | -0.0305 | 0.0043 | 1.72E-12 |
| rs145580088 | Smoking Cessation | EUP | G | 0.02 | 0.0910 | 0.0127 | 9.48E-13 |
| rs56113850 | Smoking Cessation | EUP | C | 0.57 | -0.0576 | 0.0039 | 1.61E-48 |

| rs117824460 | Smoking Cessation | EUP | G | 0.03 | 0.0865 | 0.0121 | 1.09E-12 |
| --- | --- | --- | --- | --- | --- | --- | --- |
| rs59586387 | Smoking Cessation | EUP | G | 0.07 | 0.0514 | 0.0078 | 3.37E-11 |
| rs6011779 | Smoking Cessation | EUP | T | 0.81 | -0.0500 | 0.0050 | 9.89E-24 |
| rs4809543 | Smoking Cessation | EUP | A | 0.08 | 0.0444 | 0.0074 | 2.40E-09 |
| rs6089904 | Smoking Cessation | EUP | T | 0.05 | -0.0642 | 0.0093 | 4.01E-12 |
| rs9607805 | Smoking Cessation | EUP | T | 0.73 | 0.0295 | 0.0044 | 1.37E-11 |
| rs12130857 | Smoking Initiation | EUP | A | 0.32 | -0.0180 | 0.0027 | 3.65E-11 |
| rs301807 | Smoking Initiation | EUP | G | 0.57 | 0.0180 | 0.0026 | 2.50E-12 |
| rs3820277 | Smoking Initiation | EUP | T | 0.53 | -0.0188 | 0.0026 | 1.57E-13 |
| rs1889571 | Smoking Initiation | EUP | G | 0.13 | 0.0222 | 0.0038 | 4.19E-09 |
| rs10914684 | Smoking Initiation | EUP | A | 0.32 | -0.0158 | 0.0027 | 6.32E-09 |
| rs2637869 | Smoking Initiation | EUP | A | 0.30 | 0.0182 | 0.0028 | 6.54E-11 |
| rs12755632 | Smoking Initiation | EUP | G | 0.32 | -0.0154 | 0.0027 | 1.93E-08 |
| rs951740 | Smoking Initiation | EUP | A | 0.63 | 0.0295 | 0.0026 | 3.82E-29 |
| rs925524 | Smoking Initiation | EUP | G | 0.71 | 0.0156 | 0.0028 | 2.94E-08 |
| rs12022778 | Smoking Initiation | EUP | C | 0.20 | 0.0268 | 0.0032 | 3.18E-17 |
| rs11587399 | Smoking Initiation | EUP | T | 0.22 | -0.0178 | 0.0031 | 7.25E-09 |
| rs4912332 | Smoking Initiation | EUP | T | 0.49 | 0.0141 | 0.0025 | 2.94E-08 |
| rs1937443 | Smoking Initiation | EUP | G | 0.56 | 0.0204 | 0.0026 | 1.79E-15 |
| rs1022528 | Smoking Initiation | EUP | A | 0.34 | 0.0174 | 0.0027 | 8.48E-11 |
| rs12740789 | Smoking Initiation | EUP | A | 0.18 | -0.0285 | 0.0033 | 1.18E-17 |
| rs80054503 | Smoking Initiation | EUP | C | 0.12 | -0.0241 | 0.0041 | 3.10E-09 |
| rs10789369 | Smoking Initiation | EUP | G | 0.62 | -0.0234 | 0.0026 | 3.39E-19 |
| rs1514176 | Smoking Initiation | EUP | A | 0.58 | -0.0193 | 0.0026 | 7.67E-14 |
| rs10873871 | Smoking Initiation | EUP | G | 0.21 | 0.0175 | 0.0031 | 2.82E-08 |
| rs11162019 | Smoking Initiation | EUP | T | 0.36 | -0.0155 | 0.0026 | 5.06E-09 |
| rs1008078 | Smoking Initiation | EUP | T | 0.40 | 0.0228 | 0.0026 | 1.63E-18 |
| rs1935571 | Smoking Initiation | EUP | G | 0.48 | -0.0157 | 0.0026 | 6.99E-10 |
| rs12027999 | Smoking Initiation | EUP | C | 0.12 | -0.0244 | 0.0039 | 5.33E-10 |
| rs45444697 | Smoking Initiation | EUP | G | 0.21 | 0.0197 | 0.0031 | 2.72E-10 |
| rs2901785 | Smoking Initiation | EUP | A | 0.45 | -0.0173 | 0.0026 | 1.47E-11 |
| rs147052174 | Smoking Initiation | EUP | T | 0.02 | 0.0623 | 0.0098 | 2.30E-10 |
| rs35656245 | Smoking Initiation | EUP | A | 0.28 | 0.0159 | 0.0029 | 2.23E-08 |
| rs12739243 | Smoking Initiation | EUP | C | 0.22 | -0.0213 | 0.0031 | 4.45E-12 |
| rs12563365 | Smoking Initiation | EUP | A | 0.56 | 0.0166 | 0.0026 | 1.05E-10 |
| rs876793 | Smoking Initiation | EUP | C | 0.35 | -0.0179 | 0.0027 | 5.69E-11 |
| rs114976176 | Smoking Initiation | EUP | C | 0.35 | -0.0155 | 0.0027 | 6.04E-09 |
| rs62106258 | Smoking Initiation | EUP | C | 0.05 | -0.0455 | 0.0060 | 3.33E-14 |
| rs6731872 | Smoking Initiation | EUP | G | 0.83 | 0.0316 | 0.0034 | 5.35E-21 |
| rs1022376 | Smoking Initiation | EUP | C | 0.52 | -0.0147 | 0.0026 | 1.66E-08 |
| rs61533748 | Smoking Initiation | EUP | C | 0.38 | 0.0174 | 0.0026 | 2.82E-11 |
| rs72790288 | Smoking Initiation | EUP | A | 0.03 | -0.0455 | 0.0077 | 3.28E-09 |
| rs2710634 | Smoking Initiation | EUP | C | 0.52 | -0.0178 | 0.0026 | 3.36E-12 |
| rs62137126 | Smoking Initiation | EUP | G | 0.12 | -0.0237 | 0.0039 | 1.31E-09 |
| rs1004787 | Smoking Initiation | EUP | A | 0.55 | 0.0284 | 0.0026 | 1.11E-28 |
| rs7598402 | Smoking Initiation | EUP | G | 0.49 | -0.0147 | 0.0025 | 7.38E-09 |
| rs10490159 | Smoking Initiation | EUP | T | 0.39 | 0.0172 | 0.0026 | 3.86E-11 |
| rs1518393 | Smoking Initiation | EUP | C | 0.62 | 0.0169 | 0.0026 | 1.30E-10 |
| rs17616642 | Smoking Initiation | EUP | G | 0.25 | -0.0166 | 0.0030 | 2.10E-08 |
| rs6730325 | Smoking Initiation | EUP | A | 0.61 | -0.0146 | 0.0026 | 2.10E-08 |
| rs2539706 | Smoking Initiation | EUP | A | 0.53 | 0.0162 | 0.0026 | 1.95E-10 |
| rs7585579 | Smoking Initiation | EUP | G | 0.50 | 0.0204 | 0.0026 | 5.48E-15 |
| rs1863161 | Smoking Initiation | EUP | A | 0.56 | 0.0153 | 0.0026 | 2.34E-09 |
| rs359247 | Smoking Initiation | EUP | T | 0.64 | 0.0220 | 0.0027 | 9.89E-17 |
| rs62180324 | Smoking Initiation | EUP | A | 0.21 | -0.0195 | 0.0031 | 3.91E-10 |
| rs6750107 | Smoking Initiation | EUP | A | 0.39 | 0.0146 | 0.0026 | 2.60E-08 |
| rs12714017 | Smoking Initiation | EUP | C | 0.51 | 0.0154 | 0.0026 | 3.65E-09 |
| rs56208390 | Smoking Initiation | EUP | G | 0.12 | 0.0216 | 0.0039 | 2.68E-08 |

| rs11692435 | Smoking Initiation | EUP | A | 0.08 | 0.0251 | 0.0046 | 4.47E-08 |
| --- | --- | --- | --- | --- | --- | --- | --- |
| rs13392222 | Smoking Initiation | EUP | C | 0.14 | -0.0234 | 0.0037 | 1.93E-10 |
| rs1901477 | Smoking Initiation | EUP | G | 0.51 | 0.0304 | 0.0026 | 2.07E-31 |
| rs11889814 | Smoking Initiation | EUP | C | 0.13 | -0.0210 | 0.0038 | 3.44E-08 |
| rs3811038 | Smoking Initiation | EUP | C | 0.28 | 0.0191 | 0.0028 | 1.58E-11 |
| rs75210106 | Smoking Initiation | EUP | T | 0.18 | -0.0187 | 0.0033 | 2.33E-08 |
| rs34399632 | Smoking Initiation | EUP | G | 0.23 | 0.0194 | 0.0030 | 1.46E-10 |
| rs74697736 | Smoking Initiation | EUP | A | 0.29 | 0.0223 | 0.0028 | 2.43E-15 |
| rs6756212 | Smoking Initiation | EUP | T | 0.54 | -0.0339 | 0.0026 | 3.49E-40 |
| rs3076896 | Smoking Initiation | EUP | A | 0.39 | 0.0226 | 0.0028 | 1.99E-16 |
| rs16826827 | Smoking Initiation | EUP | C | 0.12 | -0.0222 | 0.0039 | 9.17E-09 |
| rs1445649 | Smoking Initiation | EUP | C | 0.54 | 0.0206 | 0.0026 | 8.48E-16 |
| rs1722666 | Smoking Initiation | EUP | T | 0.73 | 0.0161 | 0.0029 | 2.17E-08 |
| rs11678980 | Smoking Initiation | EUP | A | 0.45 | 0.0177 | 0.0026 | 5.19E-12 |
| rs12474587 | Smoking Initiation | EUP | T | 0.43 | 0.0242 | 0.0026 | 4.83E-21 |
| rs357304 | Smoking Initiation | EUP | C | 0.73 | 0.0167 | 0.0029 | 5.40E-09 |
| rs13007361 | Smoking Initiation | EUP | A | 0.21 | 0.0175 | 0.0031 | 2.29E-08 |
| rs7600835 | Smoking Initiation | EUP | A | 0.34 | -0.0151 | 0.0027 | 1.80E-08 |
| rs6750529 | Smoking Initiation | EUP | T | 0.74 | 0.0199 | 0.0029 | 9.26E-12 |
| rs17229285 | Smoking Initiation | EUP | T | 0.51 | -0.0155 | 0.0025 | 1.27E-09 |
| rs3115418 | Smoking Initiation | EUP | C | 0.45 | -0.0142 | 0.0026 | 2.79E-08 |
| rs62193862 | Smoking Initiation | EUP | A | 0.10 | 0.0238 | 0.0042 | 1.99E-08 |
| rs4674916 | Smoking Initiation | EUP | A | 0.33 | -0.0180 | 0.0027 | 3.06E-11 |
| rs4674993 | Smoking Initiation | EUP | G | 0.20 | -0.0240 | 0.0032 | 4.85E-14 |
| rs11713899 | Smoking Initiation | EUP | C | 0.17 | 0.0187 | 0.0034 | 3.15E-08 |
| rs748832 | Smoking Initiation | EUP | G | 0.37 | 0.0172 | 0.0026 | 6.60E-11 |
| rs10446419 | Smoking Initiation | EUP | G | 0.21 | -0.0196 | 0.0031 | 5.05E-10 |
| rs13319205 | Smoking Initiation | EUP | A | 0.29 | 0.0165 | 0.0028 | 3.77E-09 |
| rs3172494 | Smoking Initiation | EUP | T | 0.12 | -0.0291 | 0.0040 | 3.40E-13 |
| rs2526390 | Smoking Initiation | EUP | T | 0.33 | 0.0205 | 0.0027 | 3.62E-14 |
| rs2276825 | Smoking Initiation | EUP | C | 0.25 | 0.0189 | 0.0030 | 1.89E-10 |
| rs2306866 | Smoking Initiation | EUP | T | 0.61 | -0.0167 | 0.0026 | 1.89E-10 |
| rs73831818 | Smoking Initiation | EUP | G | 0.06 | 0.0320 | 0.0055 | 5.46E-09 |
| rs1910236 | Smoking Initiation | EUP | A | 0.47 | 0.0146 | 0.0026 | 9.91E-09 |
| rs7640107 | Smoking Initiation | EUP | T | 0.43 | -0.0142 | 0.0026 | 3.46E-08 |
| rs2734390 | Smoking Initiation | EUP | G | 0.37 | 0.0148 | 0.0026 | 2.09E-08 |
| rs221988 | Smoking Initiation | EUP | C | 0.38 | -0.0149 | 0.0026 | 1.43E-08 |
| rs2196356 | Smoking Initiation | EUP | C | 0.29 | -0.0188 | 0.0028 | 2.45E-11 |
| rs11128203 | Smoking Initiation | EUP | A | 0.53 | 0.0204 | 0.0026 | 1.29E-15 |
| rs62246017 | Smoking Initiation | EUP | A | 0.32 | -0.0162 | 0.0027 | 3.03E-09 |
| rs4543050 | Smoking Initiation | EUP | T | 0.82 | 0.0222 | 0.0033 | 1.45E-11 |
| rs6782116 | Smoking Initiation | EUP | T | 0.42 | -0.0147 | 0.0026 | 1.46E-08 |
| rs13066050 | Smoking Initiation | EUP | T | 0.21 | 0.0188 | 0.0031 | 1.93E-09 |
| rs12633090 | Smoking Initiation | EUP | C | 0.18 | -0.0230 | 0.0033 | 3.16E-12 |
| rs1549979 | Smoking Initiation | EUP | T | 0.62 | -0.0245 | 0.0026 | 8.80E-21 |
| rs74664784 | Smoking Initiation | EUP | C | 0.38 | -0.0199 | 0.0028 | 9.34E-13 |
| rs57153235 | Smoking Initiation | EUP | G | 0.32 | -0.0194 | 0.0027 | 1.56E-12 |
| rs6437769 | Smoking Initiation | EUP | T | 0.58 | 0.0142 | 0.0026 | 3.74E-08 |
| rs9288999 | Smoking Initiation | EUP | A | 0.74 | 0.0174 | 0.0029 | 1.50E-09 |
| rs6438436 | Smoking Initiation | EUP | T | 0.82 | 0.0247 | 0.0033 | 5.33E-14 |
| rs12053870 | Smoking Initiation | EUP | G | 0.54 | 0.0156 | 0.0026 | 1.02E-09 |
| rs9826984 | Smoking Initiation | EUP | A | 0.54 | -0.0141 | 0.0026 | 3.87E-08 |
| rs2279829 | Smoking Initiation | EUP | T | 0.22 | -0.0174 | 0.0031 | 2.05E-08 |
| rs2319545 | Smoking Initiation | EUP | A | 0.15 | 0.0232 | 0.0036 | 8.30E-11 |
| rs10935779 | Smoking Initiation | EUP | T | 0.42 | -0.0143 | 0.0026 | 2.95E-08 |
| rs963354 | Smoking Initiation | EUP | A | 0.69 | 0.0150 | 0.0027 | 4.21E-08 |
| rs1714521 | Smoking Initiation | EUP | C | 0.41 | -0.0163 | 0.0026 | 3.07E-10 |
| rs1449012 | Smoking Initiation | EUP | T | 0.46 | -0.0154 | 0.0026 | 1.77E-09 |

| rs9850597 | Smoking Initiation | EUP | A | 0.82 | -0.0186 | 0.0033 | 1.65E-08 |
| --- | --- | --- | --- | --- | --- | --- | --- |
| rs1187820 | Smoking Initiation | EUP | T | 0.44 | -0.0143 | 0.0026 | 2.69E-08 |
| rs16828799 | Smoking Initiation | EUP | T | 0.16 | 0.0198 | 0.0035 | 1.83E-08 |
| rs9841807 | Smoking Initiation | EUP | T | 0.27 | 0.0163 | 0.0029 | 1.35E-08 |
| rs7631379 | Smoking Initiation | EUP | C | 0.21 | 0.0208 | 0.0032 | 3.94E-11 |
| rs4140932 | Smoking Initiation | EUP | A | 0.43 | -0.0140 | 0.0026 | 4.89E-08 |
| rs12642744 | Smoking Initiation | EUP | T | 0.74 | -0.0166 | 0.0030 | 2.82E-08 |
| rs59537158 | Smoking Initiation | EUP | T | 0.21 | 0.0225 | 0.0031 | 4.62E-13 |
| rs1389171 | Smoking Initiation | EUP | A | 0.24 | -0.0175 | 0.0030 | 4.45E-09 |
| rs55944129 | Smoking Initiation | EUP | C | 0.27 | -0.0176 | 0.0029 | 1.06E-09 |
| rs58400863 | Smoking Initiation | EUP | A | 0.35 | -0.0202 | 0.0027 | 4.89E-14 |
| rs7657022 | Smoking Initiation | EUP | G | 0.49 | 0.0183 | 0.0025 | 7.34E-13 |
| rs55900829 | Smoking Initiation | EUP | T | 0.33 | 0.0191 | 0.0028 | 5.63E-12 |
| rs112725451 | Smoking Initiation | EUP | T | 0.17 | 0.0261 | 0.0034 | 1.65E-14 |
| rs1160685 | Smoking Initiation | EUP | G | 0.45 | 0.0153 | 0.0026 | 2.31E-09 |
| rs1435479 | Smoking Initiation | EUP | T | 0.29 | 0.0164 | 0.0028 | 5.68E-09 |
| rs3934797 | Smoking Initiation | EUP | A | 0.18 | -0.0213 | 0.0033 | 1.12E-10 |
| rs71602617 | Smoking Initiation | EUP | T | 0.22 | -0.0178 | 0.0032 | 2.10E-08 |
| rs7696257 | Smoking Initiation | EUP | A | 0.37 | 0.0153 | 0.0026 | 6.78E-09 |
| rs13109980 | Smoking Initiation | EUP | A | 0.33 | -0.0222 | 0.0027 | 3.37E-16 |
| rs1116690 | Smoking Initiation | EUP | G | 0.74 | 0.0163 | 0.0029 | 2.16E-08 |
| rs13110073 | Smoking Initiation | EUP | C | 0.40 | -0.0246 | 0.0026 | 3.24E-21 |
| rs28717373 | Smoking Initiation | EUP | T | 0.36 | -0.0165 | 0.0027 | 6.16E-10 |
| rs62340589 | Smoking Initiation | EUP | C | 0.20 | 0.0174 | 0.0032 | 4.31E-08 |
| rs12517438 | Smoking Initiation | EUP | G | 0.54 | 0.0154 | 0.0026 | 1.89E-09 |
| rs35375873 | Smoking Initiation | EUP | C | 0.11 | -0.0270 | 0.0041 | 3.29E-11 |
| rs986714 | Smoking Initiation | EUP | T | 0.45 | -0.0160 | 0.0026 | 4.13E-10 |
| rs71592686 | Smoking Initiation | EUP | C | 0.27 | 0.0207 | 0.0029 | 3.85E-13 |
| rs2028269 | Smoking Initiation | EUP | A | 0.40 | 0.0162 | 0.0026 | 5.19E-10 |
| rs6874731 | Smoking Initiation | EUP | G | 0.48 | 0.0153 | 0.0025 | 1.83E-09 |
| rs6452785 | Smoking Initiation | EUP | T | 0.47 | -0.0269 | 0.0026 | 4.69E-26 |
| rs10805858 | Smoking Initiation | EUP | T | 0.34 | 0.0181 | 0.0027 | 1.88E-11 |
| rs181508347 | Smoking Initiation | EUP | G | 0.01 | 0.0811 | 0.0130 | 4.95E-10 |
| rs42417 | Smoking Initiation | EUP | T | 0.69 | 0.0169 | 0.0028 | 8.27E-10 |
| rs72780746 | Smoking Initiation | EUP | C | 0.17 | -0.0258 | 0.0034 | 2.05E-14 |
| rs10060196 | Smoking Initiation | EUP | A | 0.58 | 0.0183 | 0.0026 | 1.29E-12 |
| rs72789626 | Smoking Initiation | EUP | A | 0.14 | -0.0256 | 0.0037 | 5.13E-12 |
| rs17165769 | Smoking Initiation | EUP | G | 0.39 | 0.0159 | 0.0026 | 9.56E-10 |
| rs329124 | Smoking Initiation | EUP | G | 0.43 | -0.0164 | 0.0026 | 1.96E-10 |
| rs1385108 | Smoking Initiation | EUP | T | 0.24 | 0.0187 | 0.0030 | 3.84E-10 |
| rs1173461 | Smoking Initiation | EUP | T | 0.33 | 0.0166 | 0.0027 | 9.51E-10 |
| rs11956866 | Smoking Initiation | EUP | G | 0.57 | -0.0148 | 0.0026 | 7.82E-09 |
| rs3909281 | Smoking Initiation | EUP | G | 0.54 | 0.0211 | 0.0026 | 1.62E-16 |
| rs3843905 | Smoking Initiation | EUP | T | 0.40 | -0.0151 | 0.0026 | 5.41E-09 |
| rs79476395 | Smoking Initiation | EUP | G | 0.07 | 0.0334 | 0.0049 | 1.04E-11 |
| rs6890961 | Smoking Initiation | EUP | T | 0.62 | -0.0193 | 0.0026 | 2.13E-13 |
| rs4044321 | Smoking Initiation | EUP | G | 0.64 | -0.0226 | 0.0027 | 1.75E-17 |
| rs2173019 | Smoking Initiation | EUP | A | 0.18 | 0.0282 | 0.0033 | 2.98E-17 |
| rs10042827 | Smoking Initiation | EUP | C | 0.68 | 0.0167 | 0.0027 | 9.41E-10 |
| rs359431 | Smoking Initiation | EUP | T | 0.56 | -0.0142 | 0.0026 | 3.16E-08 |
| rs1059490 | Smoking Initiation | EUP | C | 0.37 | -0.0186 | 0.0026 | 2.16E-12 |
| rs6932350 | Smoking Initiation | EUP | A | 0.45 | 0.0150 | 0.0026 | 5.13E-09 |
| rs1150668 | Smoking Initiation | EUP | G | 0.42 | -0.0185 | 0.0026 | 8.54E-13 |
| rs1632941 | Smoking Initiation | EUP | C | 0.46 | -0.0158 | 0.0026 | 6.67E-10 |
| rs3218116 | Smoking Initiation | EUP | T | 0.26 | -0.0198 | 0.0029 | 1.05E-11 |
| rs160631 | Smoking Initiation | EUP | G | 0.73 | -0.0173 | 0.0029 | 1.87E-09 |
| rs7743165 | Smoking Initiation | EUP | G | 0.50 | 0.0193 | 0.0025 | 4.15E-14 |
| rs79180767 | Smoking Initiation | EUP | T | 0.25 | 0.0201 | 0.0029 | 7.00E-12 |

| rs10945141 | Smoking Initiation | EUP | A | 0.26 | 0.0181 | 0.0029 | 3.59E-10 |
| --- | --- | --- | --- | --- | --- | --- | --- |
| rs17554906 | Smoking Initiation | EUP | C | 0.44 | 0.0142 | 0.0026 | 3.14E-08 |
| rs619087 | Smoking Initiation | EUP | G | 0.42 | 0.0143 | 0.0026 | 3.10E-08 |
| rs6568832 | Smoking Initiation | EUP | A | 0.75 | 0.0189 | 0.0030 | 1.74E-10 |
| rs12195240 | Smoking Initiation | EUP | A | 0.29 | 0.0249 | 0.0028 | 1.08E-18 |
| rs6936160 | Smoking Initiation | EUP | T | 0.70 | 0.0201 | 0.0028 | 4.20E-13 |
| rs12530388 | Smoking Initiation | EUP | C | 0.51 | -0.0184 | 0.0025 | 5.83E-13 |
| rs3800227 | Smoking Initiation | EUP | G | 0.74 | 0.0172 | 0.0029 | 3.64E-09 |
| rs118202 | Smoking Initiation | EUP | T | 0.81 | -0.0367 | 0.0033 | 1.90E-29 |
| rs73008357 | Smoking Initiation | EUP | C | 0.12 | -0.0223 | 0.0040 | 2.44E-08 |
| rs9331343 | Smoking Initiation | EUP | C | 0.57 | -0.0141 | 0.0026 | 3.90E-08 |
| rs10698713 | Smoking Initiation | EUP | A | 0.05 | -0.0335 | 0.0056 | 2.38E-09 |
| rs1737329 | Smoking Initiation | EUP | G | 0.74 | 0.0170 | 0.0029 | 5.08E-09 |
| rs10272990 | Smoking Initiation | EUP | C | 0.33 | -0.0209 | 0.0027 | 1.27E-14 |
| rs6948707 | Smoking Initiation | EUP | G | 0.42 | 0.0243 | 0.0026 | 4.24E-21 |
| rs10259715 | Smoking Initiation | EUP | A | 0.21 | -0.0187 | 0.0032 | 6.42E-09 |
| rs13237637 | Smoking Initiation | EUP | C | 0.49 | -0.0237 | 0.0025 | 1.54E-20 |
| rs79631993 | Smoking Initiation | EUP | C | 0.22 | -0.0170 | 0.0031 | 3.67E-08 |
| rs7809303 | Smoking Initiation | EUP | A | 0.33 | -0.0214 | 0.0027 | 3.48E-15 |
| rs7802996 | Smoking Initiation | EUP | T | 0.17 | -0.0209 | 0.0034 | 1.06E-09 |
| rs1030015 | Smoking Initiation | EUP | T | 0.52 | 0.0143 | 0.0026 | 2.15E-08 |
| rs4727189 | Smoking Initiation | EUP | C | 0.34 | 0.0149 | 0.0027 | 3.00E-08 |
| rs76841737 | Smoking Initiation | EUP | G | 0.10 | -0.0231 | 0.0042 | 3.26E-08 |
| rs11768481 | Smoking Initiation | EUP | A | 0.34 | -0.0186 | 0.0027 | 5.23E-12 |
| rs1799068 | Smoking Initiation | EUP | T | 0.38 | 0.0166 | 0.0026 | 2.59E-10 |
| rs13437771 | Smoking Initiation | EUP | G | 0.16 | -0.0271 | 0.0035 | 1.39E-14 |
| rs11766326 | Smoking Initiation | EUP | C | 0.51 | -0.0175 | 0.0026 | 1.79E-11 |
| rs6968380 | Smoking Initiation | EUP | A | 0.68 | -0.0234 | 0.0027 | 1.05E-17 |
| rs112913817 | Smoking Initiation | EUP | G | 0.01 | 0.0781 | 0.0120 | 9.28E-11 |
| rs10233018 | Smoking Initiation | EUP | G | 0.52 | 0.0246 | 0.0025 | 4.77E-22 |
| rs10953957 | Smoking Initiation | EUP | A | 0.39 | 0.0144 | 0.0026 | 3.66E-08 |
| rs77283305 | Smoking Initiation | EUP | A | 0.31 | -0.0152 | 0.0028 | 3.91E-08 |
| rs10279261 | Smoking Initiation | EUP | A | 0.62 | -0.0189 | 0.0026 | 6.05E-13 |
| rs1561112 | Smoking Initiation | EUP | C | 0.41 | -0.0152 | 0.0026 | 3.84E-09 |
| rs2952251 | Smoking Initiation | EUP | G | 0.74 | 0.0164 | 0.0030 | 4.24E-08 |
| rs4326350 | Smoking Initiation | EUP | G | 0.49 | -0.0176 | 0.0026 | 5.16E-12 |
| rs11780471 | Smoking Initiation | EUP | A | 0.06 | -0.0387 | 0.0052 | 1.57E-13 |
| rs11783093 | Smoking Initiation | EUP | T | 0.16 | -0.0471 | 0.0035 | 2.07E-41 |
| rs1565735 | Smoking Initiation | EUP | A | 0.20 | -0.0192 | 0.0032 | 1.33E-09 |
| rs7836565 | Smoking Initiation | EUP | T | 0.72 | -0.0155 | 0.0028 | 4.36E-08 |
| rs13261666 | Smoking Initiation | EUP | T | 0.52 | -0.0200 | 0.0025 | 4.36E-15 |
| rs3850736 | Smoking Initiation | EUP | G | 0.47 | 0.0191 | 0.0026 | 6.43E-14 |
| rs2063976 | Smoking Initiation | EUP | T | 0.66 | -0.0202 | 0.0027 | 7.45E-14 |
| rs6993429 | Smoking Initiation | EUP | A | 0.45 | -0.0191 | 0.0026 | 9.87E-14 |
| rs6986430 | Smoking Initiation | EUP | C | 0.22 | -0.0243 | 0.0031 | 1.99E-15 |
| rs9987376 | Smoking Initiation | EUP | G | 0.57 | -0.0205 | 0.0026 | 2.01E-15 |
| rs290601 | Smoking Initiation | EUP | T | 0.27 | 0.0163 | 0.0029 | 1.14E-08 |
| rs3847244 | Smoking Initiation | EUP | T | 0.47 | 0.0187 | 0.0026 | 2.60E-13 |
| rs11791671 | Smoking Initiation | EUP | T | 0.07 | 0.0279 | 0.0051 | 4.24E-08 |
| rs7024924 | Smoking Initiation | EUP | C | 0.17 | 0.0189 | 0.0034 | 1.90E-08 |
| rs6474609 | Smoking Initiation | EUP | A | 0.59 | -0.0156 | 0.0026 | 1.71E-09 |
| rs1931431 | Smoking Initiation | EUP | C | 0.48 | 0.0182 | 0.0026 | 8.56E-13 |
| rs7867822 | Smoking Initiation | EUP | G | 0.67 | -0.0151 | 0.0027 | 2.76E-08 |
| rs10966092 | Smoking Initiation | EUP | C | 0.27 | -0.0205 | 0.0029 | 1.12E-12 |
| rs10969352 | Smoking Initiation | EUP | A | 0.50 | 0.0143 | 0.0025 | 1.82E-08 |
| rs4877285 | Smoking Initiation | EUP | A | 0.67 | -0.0181 | 0.0027 | 2.10E-11 |
| rs1930371 | Smoking Initiation | EUP | T | 0.24 | -0.0172 | 0.0030 | 7.09E-09 |
| rs2378662 | Smoking Initiation | EUP | A | 0.54 | 0.0152 | 0.0026 | 2.67E-09 |

| rs1927901 | Smoking Initiation | EUP | C | 0.55 | -0.0142 | 0.0026 | 3.10E-08 |
| --- | --- | --- | --- | --- | --- | --- | --- |
| rs4837631 | Smoking Initiation | EUP | T | 0.45 | -0.0154 | 0.0026 | 2.03E-09 |
| rs1759433 | Smoking Initiation | EUP | A | 0.48 | 0.0154 | 0.0026 | 1.69E-09 |
| rs34553878 | Smoking Initiation | EUP | G | 0.11 | 0.0247 | 0.0041 | 1.17E-09 |
| rs7026534 | Smoking Initiation | EUP | G | 0.70 | -0.0166 | 0.0028 | 2.68E-09 |
| rs10858334 | Smoking Initiation | EUP | G | 0.14 | 0.0229 | 0.0038 | 1.18E-09 |
| rs10905461 | Smoking Initiation | EUP | C | 0.75 | -0.0164 | 0.0029 | 2.36E-08 |
| rs7920501 | Smoking Initiation | EUP | A | 0.47 | -0.0155 | 0.0026 | 1.25E-09 |
| rs1291821 | Smoking Initiation | EUP | G | 0.53 | 0.0145 | 0.0026 | 1.39E-08 |
| rs11258417 | Smoking Initiation | EUP | T | 0.39 | -0.0145 | 0.0026 | 2.71E-08 |
| rs7072776 | Smoking Initiation | EUP | G | 0.71 | -0.0220 | 0.0028 | 5.66E-15 |
| rs2796793 | Smoking Initiation | EUP | A | 0.45 | 0.0145 | 0.0026 | 1.55E-08 |
| rs1733760 | Smoking Initiation | EUP | C | 0.51 | 0.0148 | 0.0025 | 6.70E-09 |
| rs7921378 | Smoking Initiation | EUP | C | 0.48 | -0.0233 | 0.0025 | 6.10E-20 |
| rs7901883 | Smoking Initiation | EUP | A | 0.23 | -0.0193 | 0.0030 | 1.98E-10 |
| rs11594623 | Smoking Initiation | EUP | C | 0.23 | 0.0274 | 0.0030 | 7.45E-20 |
| rs11191269 | Smoking Initiation | EUP | G | 0.19 | 0.0176 | 0.0032 | 4.61E-08 |
| rs28408682 | Smoking Initiation | EUP | G | 0.60 | 0.0167 | 0.0026 | 1.41E-10 |
| rs12244388 | Smoking Initiation | EUP | A | 0.35 | 0.0258 | 0.0027 | 4.31E-22 |
| rs111842178 | Smoking Initiation | EUP | G | 0.23 | 0.0225 | 0.0032 | 2.24E-12 |
| rs34970111 | Smoking Initiation | EUP | T | 0.46 | -0.0146 | 0.0026 | 1.28E-08 |
| rs9787523 | Smoking Initiation | EUP | C | 0.42 | -0.0156 | 0.0026 | 1.42E-09 |
| rs11192347 | Smoking Initiation | EUP | A | 0.10 | -0.0265 | 0.0043 | 6.15E-10 |
| rs10885480 | Smoking Initiation | EUP | C | 0.28 | -0.0187 | 0.0028 | 3.83E-11 |
| rs4752018 | Smoking Initiation | EUP | A | 0.23 | 0.0189 | 0.0030 | 4.42E-10 |
| rs9423279 | Smoking Initiation | EUP | G | 0.65 | -0.0186 | 0.0027 | 3.06E-12 |
| rs6265 | Smoking Initiation | EUP | T | 0.19 | -0.0293 | 0.0033 | 2.81E-19 |
| rs4275621 | Smoking Initiation | EUP | G | 0.38 | -0.0214 | 0.0026 | 3.76E-16 |
| rs62618693 | Smoking Initiation | EUP | T | 0.04 | -0.0353 | 0.0063 | 2.09E-08 |
| rs2939756 | Smoking Initiation | EUP | A | 0.48 | -0.0157 | 0.0026 | 7.45E-10 |
| rs1381775 | Smoking Initiation | EUP | C | 0.71 | -0.0156 | 0.0028 | 2.79E-08 |
| rs2959084 | Smoking Initiation | EUP | A | 0.70 | 0.0171 | 0.0028 | 9.82E-10 |
| rs3740977 | Smoking Initiation | EUP | C | 0.17 | 0.0195 | 0.0034 | 1.17E-08 |
| rs61886926 | Smoking Initiation | EUP | T | 0.38 | -0.0179 | 0.0026 | 7.30E-12 |
| rs61884449 | Smoking Initiation | EUP | T | 0.15 | 0.0200 | 0.0036 | 2.32E-08 |
| rs644740 | Smoking Initiation | EUP | T | 0.46 | -0.0141 | 0.0026 | 3.67E-08 |
| rs7943721 | Smoking Initiation | EUP | A | 0.83 | -0.0212 | 0.0034 | 3.58E-10 |
| rs7929518 | Smoking Initiation | EUP | G | 0.77 | 0.0192 | 0.0030 | 2.55E-10 |
| rs586699 | Smoking Initiation | EUP | A | 0.54 | -0.0148 | 0.0026 | 7.29E-09 |
| rs76460663 | Smoking Initiation | EUP | G | 0.04 | -0.0423 | 0.0064 | 4.15E-11 |
| rs2155646 | Smoking Initiation | EUP | C | 0.40 | 0.0378 | 0.0026 | 9.44E-48 |
| rs78239456 | Smoking Initiation | EUP | T | 0.38 | -0.0185 | 0.0027 | 9.37E-12 |
| rs1713676 | Smoking Initiation | EUP | G | 0.52 | -0.0167 | 0.0026 | 5.38E-11 |
| rs238896 | Smoking Initiation | EUP | A | 0.49 | -0.0169 | 0.0025 | 3.65E-11 |
| rs540860 | Smoking Initiation | EUP | G | 0.54 | 0.0176 | 0.0026 | 5.75E-12 |
| rs1944689 | Smoking Initiation | EUP | T | 0.79 | 0.0177 | 0.0031 | 1.27E-08 |
| rs1834306 | Smoking Initiation | EUP | G | 0.58 | -0.0145 | 0.0026 | 1.96E-08 |
| rs1106363 | Smoking Initiation | EUP | T | 0.34 | 0.0174 | 0.0027 | 9.20E-11 |
| rs2010921 | Smoking Initiation | EUP | A | 0.31 | 0.0174 | 0.0028 | 2.47E-10 |
| rs11057005 | Smoking Initiation | EUP | G | 0.44 | -0.0157 | 0.0026 | 9.12E-10 |
| rs13906 | Smoking Initiation | EUP | T | 0.11 | -0.0245 | 0.0041 | 1.98E-09 |
| rs4759229 | Smoking Initiation | EUP | G | 0.66 | 0.0156 | 0.0027 | 6.53E-09 |
| rs7969559 | Smoking Initiation | EUP | G | 0.71 | -0.0170 | 0.0028 | 1.53E-09 |
| rs7134009 | Smoking Initiation | EUP | C | 0.29 | -0.0158 | 0.0029 | 4.30E-08 |
| rs77215829 | Smoking Initiation | EUP | C | 0.13 | -0.0240 | 0.0038 | 2.02E-10 |
| rs1109480 | Smoking Initiation | EUP | A | 0.38 | -0.0167 | 0.0026 | 1.84E-10 |
| rs11611651 | Smoking Initiation | EUP | A | 0.09 | 0.0271 | 0.0045 | 2.05E-09 |
| rs17197663 | Smoking Initiation | EUP | A | 0.13 | -0.0216 | 0.0039 | 2.06E-08 |

| rs4264267 | Smoking Initiation | EUP | T | 0.53 | 0.0148 | 0.0026 | 6.82E-09 |
| --- | --- | --- | --- | --- | --- | --- | --- |
| rs61959481 | Smoking Initiation | EUP | A | 0.21 | -0.0203 | 0.0031 | 7.95E-11 |
| rs3098272 | Smoking Initiation | EUP | C | 0.80 | -0.0178 | 0.0032 | 2.08E-08 |
| rs9538162 | Smoking Initiation | EUP | C | 0.42 | 0.0174 | 0.0026 | 1.76E-11 |
| rs1413119 | Smoking Initiation | EUP | T | 0.40 | -0.0153 | 0.0026 | 4.77E-09 |
| rs56367474 | Smoking Initiation | EUP | T | 0.30 | -0.0173 | 0.0028 | 4.20E-10 |
| rs55786907 | Smoking Initiation | EUP | G | 0.16 | 0.0194 | 0.0035 | 1.84E-08 |
| rs4886207 | Smoking Initiation | EUP | C | 0.64 | -0.0162 | 0.0026 | 8.78E-10 |
| rs9540731 | Smoking Initiation | EUP | T | 0.51 | -0.0177 | 0.0025 | 3.42E-12 |
| rs9545155 | Smoking Initiation | EUP | C | 0.48 | -0.0161 | 0.0026 | 3.04E-10 |
| rs1772572 | Smoking Initiation | EUP | A | 0.32 | -0.0169 | 0.0027 | 5.62E-10 |
| rs75674569 | Smoking Initiation | EUP | A | 0.10 | -0.0253 | 0.0043 | 2.58E-09 |
| rs7333559 | Smoking Initiation | EUP | A | 0.78 | -0.0232 | 0.0031 | 5.94E-14 |
| rs1108130 | Smoking Initiation | EUP | A | 0.21 | 0.0239 | 0.0031 | 1.57E-14 |
| rs12855717 | Smoking Initiation | EUP | T | 0.54 | 0.0155 | 0.0026 | 1.22E-09 |
| rs12878369 | Smoking Initiation | EUP | A | 0.41 | 0.0174 | 0.0026 | 1.60E-11 |
| rs2145451 | Smoking Initiation | EUP | C | 0.19 | -0.0200 | 0.0032 | 5.44E-10 |
| rs9323328 | Smoking Initiation | EUP | G | 0.54 | -0.0142 | 0.0026 | 2.55E-08 |
| rs1811739 | Smoking Initiation | EUP | A | 0.25 | 0.0183 | 0.0030 | 5.97E-10 |
| rs8005334 | Smoking Initiation | EUP | G | 0.36 | 0.0167 | 0.0027 | 3.44E-10 |
| rs34940743 | Smoking Initiation | EUP | G | 0.35 | 0.0159 | 0.0027 | 2.80E-09 |
| rs2925128 | Smoking Initiation | EUP | T | 0.39 | 0.0168 | 0.0027 | 3.67E-10 |
| rs1381287 | Smoking Initiation | EUP | T | 0.47 | 0.0180 | 0.0026 | 1.81E-12 |
| rs55913542 | Smoking Initiation | EUP | T | 0.18 | 0.0186 | 0.0034 | 3.25E-08 |
| rs1435672 | Smoking Initiation | EUP | C | 0.56 | 0.0141 | 0.0026 | 3.82E-08 |
| rs281296 | Smoking Initiation | EUP | A | 0.36 | 0.0247 | 0.0027 | 1.59E-20 |
| rs1435741 | Smoking Initiation | EUP | A | 0.43 | 0.0183 | 0.0026 | 1.09E-12 |
| rs56902655 | Smoking Initiation | EUP | G | 0.14 | -0.0219 | 0.0037 | 4.09E-09 |
| rs2289791 | Smoking Initiation | EUP | T | 0.25 | -0.0177 | 0.0030 | 2.01E-09 |
| rs60833441 | Smoking Initiation | EUP | G | 0.46 | -0.0143 | 0.0026 | 2.28E-08 |
| rs62007780 | Smoking Initiation | EUP | T | 0.42 | -0.0159 | 0.0026 | 7.48E-10 |
| rs12442563 | Smoking Initiation | EUP | T | 0.22 | -0.0232 | 0.0031 | 3.13E-14 |
| rs4310804 | Smoking Initiation | EUP | G | 0.25 | -0.0182 | 0.0030 | 7.55E-10 |
| rs8027457 | Smoking Initiation | EUP | C | 0.51 | 0.0153 | 0.0025 | 1.88E-09 |
| rs1139897 | Smoking Initiation | EUP | A | 0.23 | -0.0241 | 0.0030 | 1.77E-15 |
| rs11076962 | Smoking Initiation | EUP | C | 0.28 | 0.0183 | 0.0028 | 1.20E-10 |
| rs7192140 | Smoking Initiation | EUP | C | 0.50 | -0.0169 | 0.0025 | 3.40E-11 |
| rs9922607 | Smoking Initiation | EUP | T | 0.20 | -0.0222 | 0.0032 | 3.42E-12 |
| rs9941217 | Smoking Initiation | EUP | G | 0.35 | -0.0186 | 0.0027 | 3.50E-12 |
| rs7188873 | Smoking Initiation | EUP | G | 0.61 | 0.0203 | 0.0026 | 8.46E-15 |
| rs6497840 | Smoking Initiation | EUP | A | 0.71 | 0.0228 | 0.0029 | 2.01E-15 |
| rs4785187 | Smoking Initiation | EUP | A | 0.22 | 0.0200 | 0.0031 | 6.55E-11 |
| rs8050598 | Smoking Initiation | EUP | T | 0.25 | 0.0187 | 0.0029 | 1.76E-10 |
| rs12918191 | Smoking Initiation | EUP | G | 0.24 | -0.0197 | 0.0030 | 3.14E-11 |
| rs9302604 | Smoking Initiation | EUP | G | 0.44 | 0.0187 | 0.0026 | 3.29E-13 |
| rs9936784 | Smoking Initiation | EUP | G | 0.53 | 0.0140 | 0.0026 | 4.33E-08 |
| rs62052916 | Smoking Initiation | EUP | T | 0.07 | -0.0319 | 0.0050 | 1.62E-10 |
| rs4788676 | Smoking Initiation | EUP | C | 0.23 | -0.0177 | 0.0030 | 4.92E-09 |
| rs61537885 | Smoking Initiation | EUP | C | 0.04 | -0.0401 | 0.0069 | 8.06E-09 |
| rs117657830 | Smoking Initiation | EUP | G | 0.04 | -0.0378 | 0.0064 | 3.18E-09 |
| rs1050847 | Smoking Initiation | EUP | T | 0.56 | -0.0148 | 0.0026 | 7.37E-09 |
| rs11642231 | Smoking Initiation | EUP | A | 0.37 | -0.0156 | 0.0026 | 3.44E-09 |
| rs4790874 | Smoking Initiation | EUP | T | 0.53 | 0.0174 | 0.0026 | 8.43E-12 |
| rs11078713 | Smoking Initiation | EUP | G | 0.42 | -0.0146 | 0.0026 | 1.59E-08 |
| rs28441558 | Smoking Initiation | EUP | C | 0.06 | -0.0356 | 0.0055 | 1.24E-10 |
| rs11651955 | Smoking Initiation | EUP | A | 0.50 | -0.0140 | 0.0025 | 3.74E-08 |
| rs67777803 | Smoking Initiation | EUP | T | 0.17 | -0.0246 | 0.0034 | 3.18E-13 |
| rs2344976 | Smoking Initiation | EUP | C | 0.61 | -0.0151 | 0.0026 | 7.98E-09 |

| rs3764351 | Smoking Initiation | EUP | A | 0.66 | -0.0147 | 0.0027 | 3.89E-08 |
| --- | --- | --- | --- | --- | --- | --- | --- |
| rs72836318 | Smoking Initiation | EUP | C | 0.25 | -0.0171 | 0.0030 | 7.00E-09 |
| rs17692129 | Smoking Initiation | EUP | T | 0.33 | 0.0196 | 0.0027 | 4.57E-13 |
| rs75919030 | Smoking Initiation | EUP | C | 0.27 | -0.0210 | 0.0029 | 3.35E-13 |
| rs2938134 | Smoking Initiation | EUP | A | 0.67 | -0.0175 | 0.0028 | 3.14E-10 |
| rs2587507 | Smoking Initiation | EUP | C | 0.50 | -0.0147 | 0.0025 | 8.69E-09 |
| rs34342129 | Smoking Initiation | EUP | C | 0.51 | -0.0143 | 0.0025 | 2.13E-08 |
| rs4476253 | Smoking Initiation | EUP | A | 0.24 | -0.0185 | 0.0030 | 5.78E-10 |
| rs7505855 | Smoking Initiation | EUP | T | 0.59 | -0.0170 | 0.0026 | 5.31E-11 |
| rs8096225 | Smoking Initiation | EUP | C | 0.70 | 0.0155 | 0.0028 | 2.63E-08 |
| rs67050670 | Smoking Initiation | EUP | G | 0.23 | -0.0203 | 0.0030 | 2.34E-11 |
| rs2359180 | Smoking Initiation | EUP | G | 0.37 | -0.0144 | 0.0026 | 4.98E-08 |
| rs72898831 | Smoking Initiation | EUP | G | 0.16 | -0.0244 | 0.0035 | 4.14E-12 |
| rs8083764 | Smoking Initiation | EUP | T | 0.31 | -0.0160 | 0.0028 | 7.97E-09 |
| rs1373178 | Smoking Initiation | EUP | G | 0.59 | -0.0203 | 0.0026 | 4.16E-15 |
| rs62098013 | Smoking Initiation | EUP | A | 0.37 | 0.0177 | 0.0026 | 2.24E-11 |
| rs72938304 | Smoking Initiation | EUP | A | 0.11 | -0.0272 | 0.0040 | 1.36E-11 |
| rs11872397 | Smoking Initiation | EUP | A | 0.25 | -0.0171 | 0.0029 | 5.20E-09 |
| rs71367544 | Smoking Initiation | EUP | T | 0.20 | 0.0206 | 0.0032 | 8.54E-11 |
| rs76608582 | Smoking Initiation | EUP | A | 0.05 | -0.0345 | 0.0059 | 4.88E-09 |
| rs10853981 | Smoking Initiation | EUP | A | 0.33 | 0.0148 | 0.0027 | 4.88E-08 |
| rs113230003 | Smoking Initiation | EUP | A | 0.26 | -0.0189 | 0.0029 | 1.05E-10 |
| rs8103660 | Smoking Initiation | EUP | C | 0.35 | 0.0158 | 0.0027 | 3.03E-09 |
| rs117734003 | Smoking Initiation | EUP | C | 0.07 | 0.0303 | 0.0051 | 2.57E-09 |
| rs1126757 | Smoking Initiation | EUP | T | 0.47 | 0.0142 | 0.0026 | 2.92E-08 |
| rs6050446 | Smoking Initiation | EUP | G | 0.97 | 0.0544 | 0.0076 | 8.80E-13 |
| rs6058782 | Smoking Initiation | EUP | T | 0.91 | 0.0297 | 0.0044 | 1.78E-11 |
| rs1555445 | Smoking Initiation | EUP | T | 0.32 | 0.0188 | 0.0027 | 7.75E-12 |
| rs6073075 | Smoking Initiation | EUP | A | 0.82 | -0.0187 | 0.0034 | 2.44E-08 |
| rs910912 | Smoking Initiation | EUP | C | 0.74 | -0.0168 | 0.0029 | 7.82E-09 |
| rs6011779 | Smoking Initiation | EUP | T | 0.81 | -0.0192 | 0.0032 | 2.83E-09 |
| rs3810496 | Smoking Initiation | EUP | C | 0.62 | 0.0159 | 0.0026 | 1.54E-09 |
| rs4818005 | Smoking Initiation | EUP | A | 0.58 | -0.0204 | 0.0026 | 1.09E-14 |
| rs139896 | Smoking Initiation | EUP | C | 0.65 | 0.0154 | 0.0027 | 7.14E-09 |
| rs4822102 | Smoking Initiation | EUP | T | 0.62 | -0.0165 | 0.0026 | 2.78E-10 |
| rs9627272 | Smoking Initiation | EUP | C | 0.41 | -0.0155 | 0.0026 | 2.42E-09 |
| rs429358 | Moderate-to-Vigorous Physical Activ | EUP | T | 0.85 | -0.0190 | 0.0029 | 7.30E-11 |
| rs7804463 | Moderate-to-Vigorous Physical Activ | EUP | T | 0.53 | 0.0130 | 0.0021 | 4.10E-10 |
| rs2854277 | Moderate-to-Vigorous Physical Activ | EUP | C | 0.92 | 0.0270 | 0.0048 | 1.40E-08 |
| rs7791992 | Moderate-to-Vigorous Physical Activ | EUP | C | 0.41 | -0.0120 | 0.0021 | 1.70E-08 |
| rs3094622 | Moderate-to-Vigorous Physical Activ | EUP | A | 0.86 | 0.0180 | 0.0031 | 1.00E-08 |
| rs149943 | Moderate-to-Vigorous Physical Activ | EUP | G | 0.85 | 0.0160 | 0.0029 | 5.60E-08 |
| rs2035562 | Moderate-to-Vigorous Physical Activ | EUP | A | 0.33 | -0.0140 | 0.0023 | 1.00E-09 |
| rs2988004 | Moderate-to-Vigorous Physical Activ | EUP | T | 0.56 | -0.0140 | 0.0021 | 2.40E-11 |
| rs1043595 | Moderate-to-Vigorous Physical Activ | EUP | G | 0.72 | 0.0130 | 0.0024 | 4.20E-08 |
| rs1248860 | Vigorous PA: ≥ 3 vs. 0 days/week | EUP | G | 0.48 | -0.0513 | 0.0066 | 5.30E-15 |
| rs2764261 | Vigorous PA: ≥ 3 vs. 0 days/week | EUP | A | 0.37 | 0.0296 | 0.0054 | 5.30E-08 |
| rs13243553 | Vigorous PA: ≥ 3 vs. 0 days/week | EUP | G | 0.61 | 0.0392 | 0.0066 | 2.40E-09 |
| rs3781411 | Vigorous PA: ≥ 3 vs. 0 days/week | EUP | C | 0.88 | 0.0583 | 0.0090 | 1.00E-10 |
| rs328902 | Vigorous PA: ≥ 3 vs. 0 days/week | EUP | C | 0.69 | -0.0408 | 0.0064 | 1.30E-10 |
| rs62253088 | Strenuous sports or other exercises: ≥ | EUP | T | 0.33 | 0.0488 | 0.0050 | 8.90E-23 |
| rs166840 | Strenuous sports or other exercises: ≥ | EUP | G | 0.59 | 0.0296 | 0.0045 | 7.30E-11 |
| rs10946808 | Strenuous sports or other exercises: ≥ | EUP | A | 0.73 | -0.0305 | 0.0055 | 3.80E-08 |
| rs159544 | Strenuous sports or other exercises: ≥ | EUP | A | 0.61 | -0.0305 | 0.0051 | 2.70E-09 |
| rs75930676 | Strenuous sports or other exercises: ≥ | EUP | T | 0.95 | -0.0726 | 0.0118 | 8.50E-10 |
| rs111901094 | Strenuous sports or other exercises: ≥ | EUP | G | 0.82 | 0.0392 | 0.0072 | 5.10E-08 |
| rs9532243 | Systolic Blood Pressure | EUP | A | 0.48 | 0.2709 | 0.0243 | 6.43E-29 |
| rs72683923 | Systolic Blood Pressure | EUP | T | 0.98 | 0.9769 | 0.0934 | 1.40E-25 |

| rs7023828 | Systolic Blood Pressure | EUP | T | 0.42 | -0.2436 | 0.0249 | 1.22E-22 |
| --- | --- | --- | --- | --- | --- | --- | --- |
| rs2613765 | Systolic Blood Pressure | EUP | A | 0.47 | -0.2336 | 0.0244 | 9.01E-22 |
| rs34413141 | Systolic Blood Pressure | EUP | A | 0.18 | -0.3084 | 0.0325 | 2.10E-21 |
| rs1585453 | Systolic Blood Pressure | EUP | A | 0.89 | -0.3892 | 0.0412 | 3.53E-21 |
| rs10069690 | Systolic Blood Pressure | EUP | T | 0.26 | 0.2735 | 0.0290 | 3.78E-21 |
| rs8014182 | Systolic Blood Pressure | EUP | T | 0.14 | -0.3397 | 0.0361 | 4.99E-21 |
| rs7439567 | Systolic Blood Pressure | EUP | T | 0.42 | 0.2355 | 0.0251 | 7.35E-21 |
| rs1906672 | Systolic Blood Pressure | EUP | A | 0.23 | 0.2715 | 0.0291 | 1.03E-20 |
| rs4925159 | Systolic Blood Pressure | EUP | A | 0.43 | 0.2281 | 0.0247 | 2.27E-20 |
| rs606950 | Systolic Blood Pressure | EUP | A | 0.62 | 0.2324 | 0.0253 | 3.77E-20 |
| rs2610990 | Systolic Blood Pressure | EUP | A | 0.26 | -0.2573 | 0.0280 | 3.82E-20 |
| rs10858966 | Systolic Blood Pressure | EUP | C | 0.29 | 0.2540 | 0.0277 | 5.56E-20 |
| rs2024385 | Systolic Blood Pressure | EUP | A | 0.42 | -0.2326 | 0.0256 | 1.05E-19 |
| rs839755 | Systolic Blood Pressure | EUP | A | 0.62 | -0.2274 | 0.0251 | 1.36E-19 |
| rs34072724 | Systolic Blood Pressure | EUP | A | 0.49 | -0.2181 | 0.0244 | 4.09E-19 |
| rs1986971 | Systolic Blood Pressure | EUP | A | 0.70 | 0.2377 | 0.0274 | 3.93E-18 |
| rs231708 | Systolic Blood Pressure | EUP | C | 0.69 | -0.2281 | 0.0264 | 5.24E-18 |
| rs1133400 | Systolic Blood Pressure | EUP | A | 0.79 | -0.2607 | 0.0303 | 7.04E-18 |
| rs17035181 | Systolic Blood Pressure | EUP | T | 0.85 | 0.2949 | 0.0348 | 2.17E-17 |
| rs10048404 | Systolic Blood Pressure | EUP | T | 0.37 | -0.2277 | 0.0270 | 3.10E-17 |
| rs9368222 | Systolic Blood Pressure | EUP | A | 0.27 | 0.2301 | 0.0273 | 3.85E-17 |
| rs6021247 | Systolic Blood Pressure | EUP | A | 0.53 | 0.2041 | 0.0243 | 4.29E-17 |
| rs11632436 | Systolic Blood Pressure | EUP | C | 0.50 | 0.2056 | 0.0245 | 5.11E-17 |
| rs4980515 | Systolic Blood Pressure | EUP | T | 0.50 | 0.2086 | 0.0251 | 9.07E-17 |
| rs4385883 | Systolic Blood Pressure | EUP | T | 0.29 | -0.2331 | 0.0281 | 1.17E-16 |
| rs709668 | Systolic Blood Pressure | EUP | A | 0.20 | -0.2534 | 0.0306 | 1.24E-16 |
| rs11876341 | Systolic Blood Pressure | EUP | A | 0.69 | -0.2225 | 0.0270 | 1.93E-16 |
| rs10437954 | Systolic Blood Pressure | EUP | A | 0.90 | -0.3471 | 0.0424 | 2.64E-16 |
| rs10782230 | Systolic Blood Pressure | EUP | A | 0.48 | 0.1995 | 0.0246 | 5.27E-16 |
| rs246973 | Systolic Blood Pressure | EUP | T | 0.29 | 0.2244 | 0.0278 | 7.25E-16 |
| rs12638085 | Systolic Blood Pressure | EUP | A | 0.35 | 0.2132 | 0.0265 | 8.36E-16 |
| rs78474310 | Systolic Blood Pressure | EUP | A | 0.96 | -0.4828 | 0.0600 | 8.38E-16 |
| rs3743157 | Systolic Blood Pressure | EUP | A | 0.17 | 0.2602 | 0.0325 | 1.27E-15 |
| rs5742643 | Systolic Blood Pressure | EUP | A | 0.25 | -0.2236 | 0.0282 | 2.15E-15 |
| rs7963801 | Systolic Blood Pressure | EUP | T | 0.41 | -0.2027 | 0.0256 | 2.29E-15 |
| rs4926499 | Systolic Blood Pressure | EUP | C | 0.82 | 0.2954 | 0.0374 | 2.91E-15 |
| rs34430710 | Systolic Blood Pressure | EUP | A | 0.68 | -0.2069 | 0.0264 | 4.26E-15 |
| rs12454712 | Systolic Blood Pressure | EUP | T | 0.62 | 0.2038 | 0.0260 | 4.41E-15 |
| rs35590893 | Systolic Blood Pressure | EUP | A | 0.27 | -0.2152 | 0.0275 | 4.65E-15 |
| rs1043069 | Systolic Blood Pressure | EUP | T | 0.62 | 0.1995 | 0.0255 | 5.19E-15 |
| rs4651224 | Systolic Blood Pressure | EUP | T | 0.45 | 0.1944 | 0.0250 | 7.29E-15 |
| rs2300481 | Systolic Blood Pressure | EUP | T | 0.39 | 0.1949 | 0.0251 | 8.01E-15 |
| rs702395 | Systolic Blood Pressure | EUP | T | 0.44 | 0.1910 | 0.0246 | 8.25E-15 |
| rs4965529 | Systolic Blood Pressure | EUP | T | 0.17 | -0.2497 | 0.0325 | 1.42E-14 |
| rs1332813 | Systolic Blood Pressure | EUP | T | 0.35 | 0.1965 | 0.0259 | 3.58E-14 |
| rs2745599 | Systolic Blood Pressure | EUP | A | 0.55 | 0.1908 | 0.0253 | 4.17E-14 |
| rs28578714 | Systolic Blood Pressure | EUP | T | 0.61 | 0.1951 | 0.0258 | 4.17E-14 |
| rs9885632 | Systolic Blood Pressure | EUP | T | 0.73 | 0.2102 | 0.0279 | 5.07E-14 |
| rs912434 | Systolic Blood Pressure | EUP | T | 0.76 | 0.2151 | 0.0287 | 6.27E-14 |
| rs7912283 | Systolic Blood Pressure | EUP | A | 0.35 | 0.1943 | 0.0259 | 6.76E-14 |
| rs11112548 | Systolic Blood Pressure | EUP | A | 0.95 | 0.4608 | 0.0623 | 1.44E-13 |
| rs10233127 | Systolic Blood Pressure | EUP | A | 0.11 | 0.3025 | 0.0409 | 1.48E-13 |
| rs72688070 | Systolic Blood Pressure | EUP | T | 0.17 | -0.2410 | 0.0326 | 1.56E-13 |
| rs79598313 | Systolic Blood Pressure | EUP | T | 0.03 | 0.5926 | 0.0805 | 1.82E-13 |
| rs9526707 | Systolic Blood Pressure | EUP | A | 0.32 | -0.1939 | 0.0264 | 1.90E-13 |
| rs11623535 | Systolic Blood Pressure | EUP | A | 0.74 | 0.2022 | 0.0277 | 3.02E-13 |
| rs11159091 | Systolic Blood Pressure | EUP | A | 0.46 | 0.1793 | 0.0246 | 3.26E-13 |
| rs6788984 | Systolic Blood Pressure | EUP | A | 0.86 | 0.2558 | 0.0353 | 3.96E-13 |

| rs13179413 | Systolic Blood Pressure | EUP | T | 0.28 | 0.2045 | 0.0284 | 5.57E-13 |
| --- | --- | --- | --- | --- | --- | --- | --- |
| rs11031051 | Systolic Blood Pressure | EUP | A | 0.69 | -0.1899 | 0.0264 | 5.80E-13 |
| rs848445 | Systolic Blood Pressure | EUP | T | 0.28 | -0.2004 | 0.0279 | 6.57E-13 |
| rs62491354 | Systolic Blood Pressure | EUP | A | 0.13 | 0.2565 | 0.0358 | 7.82E-13 |
| rs7045409 | Systolic Blood Pressure | EUP | A | 0.37 | -0.1823 | 0.0255 | 9.15E-13 |
| rs11634028 | Systolic Blood Pressure | EUP | A | 0.21 | 0.2258 | 0.0316 | 9.46E-13 |
| rs139354822 | Systolic Blood Pressure | EUP | T | 0.97 | 0.5473 | 0.0768 | 1.06E-12 |
| rs4129585 | Systolic Blood Pressure | EUP | A | 0.44 | 0.1749 | 0.0246 | 1.06E-12 |
| rs1347345 | Systolic Blood Pressure | EUP | A | 0.62 | -0.1792 | 0.0253 | 1.45E-12 |
| rs4598218 | Systolic Blood Pressure | EUP | T | 0.61 | 0.1797 | 0.0254 | 1.50E-12 |
| rs12572586 | Systolic Blood Pressure | EUP | T | 0.94 | -0.3646 | 0.0517 | 1.83E-12 |
| rs67720684 | Systolic Blood Pressure | EUP | A | 0.24 | 0.2039 | 0.0291 | 2.26E-12 |
| rs1891730 | Systolic Blood Pressure | EUP | T | 0.62 | -0.1785 | 0.0256 | 2.95E-12 |
| rs28377357 | Systolic Blood Pressure | EUP | A | 0.29 | -0.1862 | 0.0267 | 3.37E-12 |
| rs11571376 | Systolic Blood Pressure | EUP | C | 0.70 | -0.1884 | 0.0271 | 3.49E-12 |
| rs2807337 | Systolic Blood Pressure | EUP | T | 0.37 | 0.1753 | 0.0252 | 3.60E-12 |
| rs62373688 | Systolic Blood Pressure | EUP | A | 0.13 | 0.2607 | 0.0378 | 5.49E-12 |
| rs34130368 | Systolic Blood Pressure | EUP | T | 0.12 | -0.2715 | 0.0394 | 5.64E-12 |
| rs72816333 | Systolic Blood Pressure | EUP | A | 0.83 | 0.2225 | 0.0323 | 5.88E-12 |
| rs7763294 | Systolic Blood Pressure | EUP | T | 0.32 | -0.1804 | 0.0263 | 6.85E-12 |
| rs189267552 | Systolic Blood Pressure | EUP | A | 0.01 | -0.7830 | 0.1154 | 1.14E-11 |
| rs6963105 | Systolic Blood Pressure | EUP | A | 0.43 | -0.1779 | 0.0263 | 1.39E-11 |
| rs28558491 | Systolic Blood Pressure | EUP | T | 0.74 | -0.1874 | 0.0278 | 1.47E-11 |
| rs9875380 | Systolic Blood Pressure | EUP | T | 0.46 | -0.1651 | 0.0245 | 1.70E-11 |
| rs7514579 | Systolic Blood Pressure | EUP | A | 0.77 | 0.1969 | 0.0293 | 1.73E-11 |
| rs11197813 | Systolic Blood Pressure | EUP | A | 0.70 | -0.1801 | 0.0268 | 1.84E-11 |
| rs1036902 | Systolic Blood Pressure | EUP | T | 0.84 | -0.2273 | 0.0338 | 1.85E-11 |
| rs1551355 | Systolic Blood Pressure | EUP | T | 0.23 | 0.1932 | 0.0288 | 1.96E-11 |
| rs67976715 | Systolic Blood Pressure | EUP | C | 0.23 | 0.1971 | 0.0295 | 2.30E-11 |
| rs6723509 | Systolic Blood Pressure | EUP | T | 0.86 | 0.2348 | 0.0351 | 2.32E-11 |
| rs12511987 | Systolic Blood Pressure | EUP | T | 0.82 | -0.2153 | 0.0323 | 2.71E-11 |
| rs863930 | Systolic Blood Pressure | EUP | A | 0.54 | 0.1629 | 0.0245 | 2.89E-11 |
| rs79523138 | Systolic Blood Pressure | EUP | A | 0.88 | -0.2629 | 0.0398 | 3.82E-11 |
| rs17396055 | Systolic Blood Pressure | EUP | A | 0.33 | -0.1727 | 0.0264 | 5.73E-11 |
| rs1044822 | Systolic Blood Pressure | EUP | T | 0.15 | -0.2275 | 0.0348 | 6.15E-11 |
| rs12042924 | Systolic Blood Pressure | EUP | T | 0.53 | -0.1615 | 0.0247 | 6.26E-11 |
| rs12694277 | Systolic Blood Pressure | EUP | T | 0.30 | -0.1765 | 0.0271 | 7.61E-11 |
| rs10274928 | Systolic Blood Pressure | EUP | A | 0.49 | 0.1592 | 0.0245 | 8.68E-11 |
| rs7187540 | Systolic Blood Pressure | EUP | A | 0.34 | -0.1766 | 0.0274 | 1.08E-10 |
| rs7555285 | Systolic Blood Pressure | EUP | C | 0.80 | 0.1961 | 0.0304 | 1.09E-10 |
| rs112280096 | Systolic Blood Pressure | EUP | A | 0.36 | -0.1706 | 0.0264 | 1.13E-10 |
| rs28558845 | Systolic Blood Pressure | EUP | C | 0.16 | -0.2180 | 0.0339 | 1.22E-10 |
| rs73187288 | Systolic Blood Pressure | EUP | A | 0.89 | -0.2558 | 0.0400 | 1.57E-10 |
| rs78151625 | Systolic Blood Pressure | EUP | T | 0.83 | -0.2108 | 0.0330 | 1.60E-10 |
| rs33996239 | Systolic Blood Pressure | EUP | T | 0.06 | -0.3538 | 0.0554 | 1.71E-10 |
| rs76719272 | Systolic Blood Pressure | EUP | T | 0.13 | -0.2394 | 0.0376 | 1.98E-10 |
| rs138877676 | Systolic Blood Pressure | EUP | T | 0.02 | -0.6556 | 0.1034 | 2.27E-10 |
| rs11771693 | Systolic Blood Pressure | EUP | A | 0.67 | 0.1673 | 0.0265 | 2.72E-10 |
| rs6774721 | Systolic Blood Pressure | EUP | C | 0.88 | 0.2457 | 0.0390 | 3.00E-10 |
| rs17115145 | Systolic Blood Pressure | EUP | T | 0.40 | 0.1569 | 0.0250 | 3.74E-10 |
| rs6739913 | Systolic Blood Pressure | EUP | A | 0.28 | 0.1675 | 0.0271 | 6.40E-10 |
| rs7976167 | Systolic Blood Pressure | EUP | T | 0.69 | 0.1638 | 0.0266 | 7.21E-10 |
| rs9857362 | Systolic Blood Pressure | EUP | A | 0.53 | 0.1510 | 0.0247 | 9.71E-10 |
| rs184457 | Systolic Blood Pressure | EUP | A | 0.30 | -0.1659 | 0.0272 | 1.09E-09 |
| rs2920899 | Systolic Blood Pressure | EUP | T | 0.79 | 0.1851 | 0.0304 | 1.17E-09 |
| rs12703989 | Systolic Blood Pressure | EUP | A | 0.49 | 0.1521 | 0.0250 | 1.26E-09 |
| rs10743086 | Systolic Blood Pressure | EUP | A | 0.21 | -0.1832 | 0.0303 | 1.57E-09 |
| rs3121685 | Systolic Blood Pressure | EUP | T | 0.48 | -0.1475 | 0.0246 | 1.92E-09 |

| rs74774746 | Systolic Blood Pressure | EUP | C | 0.26 | -0.1705 | 0.0285 | 2.08E-09 |
| --- | --- | --- | --- | --- | --- | --- | --- |
| rs35450617 | Systolic Blood Pressure | EUP | T | 0.70 | -0.1609 | 0.0269 | 2.33E-09 |
| rs7988232 | Systolic Blood Pressure | EUP | A | 0.42 | 0.1480 | 0.0249 | 2.99E-09 |
| rs190194639 | Systolic Blood Pressure | EUP | T | 0.08 | 0.2746 | 0.0468 | 4.55E-09 |
| rs72844590 | Systolic Blood Pressure | EUP | T | 0.15 | 0.2083 | 0.0356 | 4.77E-09 |
| rs260508 | Systolic Blood Pressure | EUP | T | 0.61 | 0.1485 | 0.0254 | 5.17E-09 |
| rs6593297 | Systolic Blood Pressure | EUP | A | 0.30 | 0.1581 | 0.0274 | 8.38E-09 |
| rs9401090 | Systolic Blood Pressure | EUP | T | 0.75 | 0.1637 | 0.0285 | 9.25E-09 |
| rs6545155 | Systolic Blood Pressure | EUP | T | 0.78 | 0.1691 | 0.0295 | 9.84E-09 |
| rs1882289 | Systolic Blood Pressure | EUP | A | 0.88 | -0.2159 | 0.0379 | 1.26E-08 |
| rs56352451 | Systolic Blood Pressure | EUP | T | 0.13 | 0.1992 | 0.0358 | 2.54E-08 |
| rs6996733 | Systolic Blood Pressure | EUP | T | 0.85 | 0.1882 | 0.0341 | 3.34E-08 |
| rs360153 | Diastolic Blood Pressure | EUP | T | 0.42 | -0.1917 | 0.0152 | 2.71E-36 |
| rs17248480 | Diastolic Blood Pressure | EUP | A | 0.03 | -0.5064 | 0.0479 | 4.07E-26 |
| rs73033340 | Diastolic Blood Pressure | EUP | A | 0.96 | 0.4615 | 0.0456 | 4.42E-24 |
| rs10062049 | Diastolic Blood Pressure | EUP | T | 0.14 | 0.2113 | 0.0221 | 1.43E-21 |
| rs34163044 | Diastolic Blood Pressure | EUP | A | 0.42 | 0.1488 | 0.0157 | 2.04E-21 |
| rs1947228 | Diastolic Blood Pressure | EUP | T | 0.42 | -0.1451 | 0.0153 | 2.39E-21 |
| rs45474499 | Diastolic Blood Pressure | EUP | T | 0.05 | 0.3433 | 0.0362 | 2.52E-21 |
| rs223361 | Diastolic Blood Pressure | EUP | T | 0.66 | 0.1522 | 0.0161 | 3.60E-21 |
| rs34517439 | Diastolic Blood Pressure | EUP | A | 0.12 | -0.2330 | 0.0248 | 4.84E-21 |
| rs35981664 | Diastolic Blood Pressure | EUP | A | 0.69 | -0.1542 | 0.0166 | 1.46E-20 |
| rs28667801 | Diastolic Blood Pressure | EUP | A | 0.59 | -0.1449 | 0.0157 | 2.28E-20 |
| rs7132012 | Diastolic Blood Pressure | EUP | A | 0.68 | 0.1480 | 0.0161 | 2.91E-20 |
| rs9472135 | Diastolic Blood Pressure | EUP | T | 0.70 | 0.1444 | 0.0165 | 2.09E-18 |
| rs11026586 | Diastolic Blood Pressure | EUP | A | 0.07 | 0.2606 | 0.0299 | 3.17E-18 |
| rs1996992 | Diastolic Blood Pressure | EUP | T | 0.05 | -0.2964 | 0.0344 | 6.54E-18 |
| rs1271309 | Diastolic Blood Pressure | EUP | A | 0.16 | -0.1786 | 0.0209 | 1.36E-17 |
| rs603424 | Diastolic Blood Pressure | EUP | A | 0.18 | 0.1717 | 0.0202 | 1.81E-17 |
| rs1706003 | Diastolic Blood Pressure | EUP | T | 0.47 | 0.1326 | 0.0157 | 2.76E-17 |
| rs7137749 | Diastolic Blood Pressure | EUP | T | 0.37 | 0.1320 | 0.0157 | 4.47E-17 |
| rs11665020 | Diastolic Blood Pressure | EUP | C | 0.32 | -0.1371 | 0.0163 | 4.55E-17 |
| rs1821295 | Diastolic Blood Pressure | EUP | T | 0.70 | -0.1347 | 0.0165 | 2.78E-16 |
| rs115245297 | Diastolic Blood Pressure | EUP | T | 0.96 | -0.3148 | 0.0385 | 3.05E-16 |
| rs954767 | Diastolic Blood Pressure | EUP | A | 0.74 | -0.1389 | 0.0171 | 4.71E-16 |
| rs1220128 | Diastolic Blood Pressure | EUP | C | 0.85 | 0.1733 | 0.0214 | 5.49E-16 |
| rs875106 | Diastolic Blood Pressure | EUP | A | 0.52 | -0.1217 | 0.0151 | 7.66E-16 |
| rs668459 | Diastolic Blood Pressure | EUP | T | 0.59 | -0.1219 | 0.0152 | 1.07E-15 |
| rs10087782 | Diastolic Blood Pressure | EUP | T | 0.45 | 0.1211 | 0.0151 | 1.21E-15 |
| rs11923667 | Diastolic Blood Pressure | EUP | A | 0.41 | 0.1222 | 0.0153 | 1.69E-15 |
| rs12142296 | Diastolic Blood Pressure | EUP | T | 0.86 | -0.1716 | 0.0222 | 1.10E-14 |
| rs1722886 | Diastolic Blood Pressure | EUP | A | 0.57 | 0.1166 | 0.0153 | 2.37E-14 |
| rs3898618 | Diastolic Blood Pressure | EUP | T | 0.95 | -0.2532 | 0.0336 | 4.76E-14 |
| rs1232482 | Diastolic Blood Pressure | EUP | T | 0.40 | -0.1155 | 0.0153 | 4.78E-14 |
| rs12938803 | Diastolic Blood Pressure | EUP | T | 0.80 | 0.1482 | 0.0197 | 5.57E-14 |
| rs55747751 | Diastolic Blood Pressure | EUP | A | 0.08 | -0.2159 | 0.0288 | 6.07E-14 |
| rs12583637 | Diastolic Blood Pressure | EUP | A | 0.13 | 0.1671 | 0.0223 | 6.46E-14 |
| rs6565174 | Diastolic Blood Pressure | EUP | A | 0.11 | -0.1821 | 0.0243 | 6.63E-14 |
| rs11628933 | Diastolic Blood Pressure | EUP | C | 0.23 | -0.1338 | 0.0179 | 7.40E-14 |
| rs4286632 | Diastolic Blood Pressure | EUP | A | 0.73 | 0.1270 | 0.0170 | 9.26E-14 |
| rs10906391 | Diastolic Blood Pressure | EUP | T | 0.32 | 0.1210 | 0.0163 | 1.32E-13 |
| rs13001283 | Diastolic Blood Pressure | EUP | A | 0.16 | 0.1513 | 0.0207 | 2.73E-13 |
| rs7606205 | Diastolic Blood Pressure | EUP | A | 0.70 | -0.1207 | 0.0166 | 3.69E-13 |
| rs55684003 | Diastolic Blood Pressure | EUP | A | 0.70 | 0.1193 | 0.0164 | 4.03E-13 |
| rs1886914 | Diastolic Blood Pressure | EUP | T | 0.41 | -0.1113 | 0.0154 | 4.18E-13 |
| rs34570306 | Diastolic Blood Pressure | EUP | T | 0.53 | -0.1115 | 0.0155 | 5.46E-13 |
| rs17880989 | Diastolic Blood Pressure | EUP | A | 0.03 | 0.3648 | 0.0506 | 5.58E-13 |
| rs55829085 | Diastolic Blood Pressure | EUP | A | 0.95 | -0.2672 | 0.0371 | 5.73E-13 |

| rs4851462 | Diastolic Blood Pressure | EUP | T | 0.63 | -0.1138 | 0.0158 | 6.43E-13 |
| --- | --- | --- | --- | --- | --- | --- | --- |
| rs7185555 | Diastolic Blood Pressure | EUP | C | 0.15 | -0.1537 | 0.0215 | 8.56E-13 |
| rs12515541 | Diastolic Blood Pressure | EUP | T | 0.61 | 0.1104 | 0.0154 | 8.66E-13 |
| rs9638084 | Diastolic Blood Pressure | EUP | A | 0.40 | 0.1107 | 0.0155 | 8.93E-13 |
| rs6681713 | Diastolic Blood Pressure | EUP | T | 0.98 | 0.4074 | 0.0575 | 1.39E-12 |
| rs9932866 | Diastolic Blood Pressure | EUP | A | 0.37 | 0.1098 | 0.0158 | 3.41E-12 |
| rs56233017 | Diastolic Blood Pressure | EUP | A | 0.04 | -0.2669 | 0.0387 | 5.49E-12 |
| rs6777317 | Diastolic Blood Pressure | EUP | A | 0.29 | 0.1147 | 0.0168 | 8.35E-12 |
| rs4634143 | Diastolic Blood Pressure | EUP | T | 0.30 | 0.1129 | 0.0166 | 9.05E-12 |
| rs1565716 | Diastolic Blood Pressure | EUP | A | 0.07 | 0.2012 | 0.0298 | 1.49E-11 |
| rs873122 | Diastolic Blood Pressure | EUP | C | 0.72 | 0.1147 | 0.0170 | 1.62E-11 |
| rs4800420 | Diastolic Blood Pressure | EUP | A | 0.29 | 0.1114 | 0.0166 | 1.90E-11 |
| rs8069739 | Diastolic Blood Pressure | EUP | T | 0.32 | -0.1082 | 0.0161 | 1.98E-11 |
| rs598682 | Diastolic Blood Pressure | EUP | A | 0.25 | -0.1153 | 0.0173 | 2.59E-11 |
| rs1607644 | Diastolic Blood Pressure | EUP | A | 0.36 | -0.1035 | 0.0155 | 2.75E-11 |
| rs7134060 | Diastolic Blood Pressure | EUP | A | 0.45 | -0.0999 | 0.0151 | 4.01E-11 |
| rs7020564 | Diastolic Blood Pressure | EUP | A | 0.70 | -0.1105 | 0.0169 | 6.79E-11 |
| rs7180952 | Diastolic Blood Pressure | EUP | T | 0.54 | -0.0984 | 0.0153 | 1.19E-10 |
| rs17224476 | Diastolic Blood Pressure | EUP | A | 0.11 | 0.1547 | 0.0241 | 1.30E-10 |
| rs2390258 | Diastolic Blood Pressure | EUP | A | 0.31 | -0.1031 | 0.0163 | 2.68E-10 |
| rs1047030 | Diastolic Blood Pressure | EUP | A | 0.81 | 0.1291 | 0.0205 | 3.01E-10 |
| rs113134141 | Diastolic Blood Pressure | EUP | A | 0.90 | -0.1569 | 0.0250 | 3.47E-10 |
| rs62004794 | Diastolic Blood Pressure | EUP | A | 0.44 | -0.0948 | 0.0151 | 3.77E-10 |
| rs7694000 | Diastolic Blood Pressure | EUP | A | 0.54 | -0.0954 | 0.0153 | 3.90E-10 |
| rs4424827 | Diastolic Blood Pressure | EUP | T | 0.57 | -0.0948 | 0.0152 | 4.17E-10 |
| rs72704264 | Diastolic Blood Pressure | EUP | C | 0.21 | 0.1173 | 0.0188 | 4.23E-10 |
| rs9563529 | Diastolic Blood Pressure | EUP | T | 0.21 | 0.1173 | 0.0188 | 4.27E-10 |
| rs2929184 | Diastolic Blood Pressure | EUP | A | 0.77 | 0.1164 | 0.0187 | 5.05E-10 |
| rs4411245 | Diastolic Blood Pressure | EUP | A | 0.29 | 0.1023 | 0.0165 | 5.92E-10 |
| rs4420291 | Diastolic Blood Pressure | EUP | A | 0.51 | 0.0937 | 0.0151 | 5.97E-10 |
| rs75507123 | Diastolic Blood Pressure | EUP | T | 0.13 | -0.1396 | 0.0227 | 8.47E-10 |
| rs11080134 | Diastolic Blood Pressure | EUP | A | 0.65 | -0.0974 | 0.0159 | 9.27E-10 |
| rs1098708 | Diastolic Blood Pressure | EUP | A | 0.54 | -0.0931 | 0.0152 | 9.30E-10 |
| rs2707238 | Diastolic Blood Pressure | EUP | C | 0.28 | 0.1019 | 0.0168 | 1.37E-09 |
| rs62229372 | Diastolic Blood Pressure | EUP | T | 0.13 | 0.1446 | 0.0240 | 1.69E-09 |
| rs34324971 | Diastolic Blood Pressure | EUP | A | 0.19 | 0.1199 | 0.0199 | 1.81E-09 |
| rs73049928 | Diastolic Blood Pressure | EUP | A | 0.81 | -0.1167 | 0.0195 | 2.13E-09 |
| rs59333122 | Diastolic Blood Pressure | EUP | A | 0.27 | 0.1069 | 0.0179 | 2.36E-09 |
| rs9865843 | Diastolic Blood Pressure | EUP | A | 0.52 | -0.0906 | 0.0153 | 3.42E-09 |
| rs56236159 | Diastolic Blood Pressure | EUP | T | 0.15 | -0.1285 | 0.0219 | 4.77E-09 |
| rs1718845 | Diastolic Blood Pressure | EUP | A | 0.30 | -0.0963 | 0.0165 | 5.14E-09 |
| rs12078697 | Diastolic Blood Pressure | EUP | C | 0.21 | -0.1083 | 0.0186 | 5.46E-09 |
| rs61892344 | Diastolic Blood Pressure | EUP | T | 0.17 | -0.1162 | 0.0199 | 5.64E-09 |
| rs2222544 | Diastolic Blood Pressure | EUP | T | 0.74 | -0.0987 | 0.0171 | 8.53E-09 |
| rs7313556 | Diastolic Blood Pressure | EUP | A | 0.35 | 0.0900 | 0.0158 | 1.24E-08 |
| rs680515 | Pulse Pressure | EUP | A | 0.39 | -0.2437 | 0.0180 | 6.98E-42 |
| rs62385385 | Pulse Pressure | EUP | A | 0.39 | 0.2177 | 0.0178 | 2.07E-34 |
| rs62053102 | Pulse Pressure | EUP | A | 0.95 | -0.5352 | 0.0440 | 4.17E-34 |
| rs62076103 | Pulse Pressure | EUP | A | 0.93 | -0.4132 | 0.0356 | 4.60E-31 |
| rs7412 | Pulse Pressure | EUP | T | 0.08 | -0.3753 | 0.0324 | 4.78E-31 |
| rs1800795 | Pulse Pressure | EUP | T | 0.58 | -0.1818 | 0.0174 | 1.53E-25 |
| rs10988442 | Pulse Pressure | EUP | A | 0.62 | 0.1783 | 0.0178 | 1.29E-23 |
| rs2820443 | Pulse Pressure | EUP | T | 0.71 | 0.1870 | 0.0189 | 3.96E-23 |
| rs7009170 | Pulse Pressure | EUP | T | 0.32 | -0.1790 | 0.0186 | 5.81E-22 |
| rs12195276 | Pulse Pressure | EUP | T | 0.72 | -0.1852 | 0.0195 | 1.80E-21 |
| rs2261608 | Pulse Pressure | EUP | A | 0.35 | 0.1693 | 0.0179 | 3.35E-21 |
| rs11770630 | Pulse Pressure | EUP | T | 0.54 | 0.1599 | 0.0172 | 1.57E-20 |
| rs704191 | Pulse Pressure | EUP | T | 0.46 | 0.1593 | 0.0175 | 7.33E-20 |

| rs11087740 | Pulse Pressure | EUP | T | 0.51 | -0.1560 | 0.0173 | 2.32E-19 |
| --- | --- | --- | --- | --- | --- | --- | --- |
| rs560887 | Pulse Pressure | EUP | T | 0.30 | -0.1676 | 0.0188 | 4.17E-19 |
| rs10842991 | Pulse Pressure | EUP | T | 0.20 | -0.1937 | 0.0218 | 5.65E-19 |
| rs3176336 | Pulse Pressure | EUP | A | 0.60 | 0.1553 | 0.0178 | 2.90E-18 |
| rs9904409 | Pulse Pressure | EUP | A | 0.10 | 0.2533 | 0.0292 | 4.15E-18 |
| rs143167197 | Pulse Pressure | EUP | A | 0.93 | -0.3016 | 0.0350 | 7.39E-18 |
| rs210381 | Pulse Pressure | EUP | A | 0.56 | -0.1501 | 0.0176 | 1.39E-17 |
| rs12807220 | Pulse Pressure | EUP | A | 0.36 | -0.1537 | 0.0182 | 2.72E-17 |
| rs13290326 | Pulse Pressure | EUP | T | 0.50 | -0.1443 | 0.0173 | 8.64E-17 |
| rs56228409 | Pulse Pressure | EUP | A | 0.85 | -0.2027 | 0.0247 | 2.39E-16 |
| rs11010905 | Pulse Pressure | EUP | A | 0.49 | 0.1417 | 0.0174 | 4.25E-16 |
| rs72659998 | Pulse Pressure | EUP | T | 0.15 | -0.1941 | 0.0241 | 8.57E-16 |
| rs34783010 | Pulse Pressure | EUP | T | 0.20 | 0.1718 | 0.0216 | 1.92E-15 |
| rs11248862 | Pulse Pressure | EUP | A | 0.12 | 0.2088 | 0.0267 | 5.44E-15 |
| rs4678915 | Pulse Pressure | EUP | A | 0.43 | -0.1386 | 0.0177 | 5.71E-15 |
| rs138285687 | Pulse Pressure | EUP | T | 0.04 | -0.3428 | 0.0441 | 7.67E-15 |
| rs267517 | Pulse Pressure | EUP | A | 0.59 | -0.1376 | 0.0177 | 8.38E-15 |
| rs10887914 | Pulse Pressure | EUP | T | 0.46 | 0.1327 | 0.0172 | 1.21E-14 |
| rs7250835 | Pulse Pressure | EUP | T | 0.17 | 0.1888 | 0.0246 | 1.67E-14 |
| rs12325702 | Pulse Pressure | EUP | A | 0.44 | 0.1374 | 0.0179 | 1.69E-14 |
| rs6731373 | Pulse Pressure | EUP | A | 0.34 | 0.1434 | 0.0188 | 2.14E-14 |
| rs9976596 | Pulse Pressure | EUP | T | 0.84 | 0.1868 | 0.0245 | 2.31E-14 |
| rs6823199 | Pulse Pressure | EUP | T | 0.74 | 0.1526 | 0.0201 | 3.66E-14 |
| rs20354 | Pulse Pressure | EUP | T | 0.13 | 0.1907 | 0.0253 | 4.69E-14 |
| rs6801957 | Pulse Pressure | EUP | T | 0.41 | 0.1315 | 0.0175 | 4.99E-14 |
| rs1630736 | Pulse Pressure | EUP | T | 0.46 | -0.1319 | 0.0176 | 6.71E-14 |
| rs512083 | Pulse Pressure | EUP | T | 0.53 | -0.1293 | 0.0173 | 7.44E-14 |
| rs7977311 | Pulse Pressure | EUP | T | 0.12 | -0.2026 | 0.0272 | 8.56E-14 |
| rs28470843 | Pulse Pressure | EUP | T | 0.60 | 0.1323 | 0.0178 | 9.83E-14 |
| rs4896104 | Pulse Pressure | EUP | T | 0.56 | -0.1316 | 0.0177 | 9.95E-14 |
| rs9860290 | Pulse Pressure | EUP | A | 0.21 | -0.1590 | 0.0214 | 1.11E-13 |
| rs37060 | Pulse Pressure | EUP | A | 0.25 | 0.1471 | 0.0198 | 1.13E-13 |
| rs1486236 | Pulse Pressure | EUP | A | 0.37 | -0.1365 | 0.0184 | 1.14E-13 |
| rs4664080 | Pulse Pressure | EUP | A | 0.39 | -0.1304 | 0.0177 | 1.94E-13 |
| rs10913934 | Pulse Pressure | EUP | T | 0.59 | 0.1302 | 0.0178 | 2.31E-13 |
| rs28499085 | Pulse Pressure | EUP | A | 0.73 | 0.1430 | 0.0196 | 2.74E-13 |
| rs151054210 | Pulse Pressure | EUP | A | 0.18 | 0.1625 | 0.0224 | 3.81E-13 |
| rs8013933 | Pulse Pressure | EUP | T | 0.70 | 0.1369 | 0.0189 | 4.08E-13 |
| rs10830963 | Pulse Pressure | EUP | C | 0.72 | -0.1411 | 0.0196 | 5.53E-13 |
| rs11222386 | Pulse Pressure | EUP | C | 0.20 | 0.1558 | 0.0217 | 7.69E-13 |
| rs832890 | Pulse Pressure | EUP | T | 0.46 | 0.1240 | 0.0173 | 8.40E-13 |
| rs36006409 | Pulse Pressure | EUP | T | 0.80 | -0.1557 | 0.0219 | 1.07E-12 |
| rs2656523 | Pulse Pressure | EUP | C | 0.77 | 0.1519 | 0.0214 | 1.25E-12 |
| rs2205260 | Pulse Pressure | EUP | A | 0.17 | 0.1627 | 0.0232 | 2.12E-12 |
| rs10982910 | Pulse Pressure | EUP | T | 0.90 | -0.2078 | 0.0298 | 2.96E-12 |
| rs71543920 | Pulse Pressure | EUP | T | 0.94 | -0.2572 | 0.0371 | 4.31E-12 |
| rs3796822 | Pulse Pressure | EUP | A | 0.70 | 0.1293 | 0.0188 | 5.58E-12 |
| rs7861040 | Pulse Pressure | EUP | C | 0.37 | -0.1253 | 0.0183 | 6.81E-12 |
| rs35504735 | Pulse Pressure | EUP | A | 0.56 | 0.1178 | 0.0172 | 8.36E-12 |
| rs141979279 | Pulse Pressure | EUP | T | 0.95 | 0.2702 | 0.0399 | 1.28E-11 |
| rs6141479 | Pulse Pressure | EUP | C | 0.20 | 0.1524 | 0.0226 | 1.59E-11 |
| rs737721 | Pulse Pressure | EUP | C | 0.94 | -0.2579 | 0.0384 | 1.92E-11 |
| rs11701512 | Pulse Pressure | EUP | A | 0.18 | 0.1510 | 0.0225 | 2.01E-11 |
| rs73080726 | Pulse Pressure | EUP | T | 0.10 | -0.1914 | 0.0286 | 2.06E-11 |
| rs6867399 | Pulse Pressure | EUP | A | 0.26 | 0.1399 | 0.0209 | 2.35E-11 |
| rs4304924 | Pulse Pressure | EUP | A | 0.57 | -0.1160 | 0.0174 | 2.74E-11 |
| rs4811601 | Pulse Pressure | EUP | T | 0.46 | 0.1164 | 0.0176 | 3.37E-11 |
| rs4977492 | Pulse Pressure | EUP | T | 0.66 | -0.1209 | 0.0183 | 3.60E-11 |

| rs963920 | Pulse Pressure | EUP | T | 0.68 | -0.1227 | 0.0185 | 3.60E-11 |
| --- | --- | --- | --- | --- | --- | --- | --- |
| rs7710854 | Pulse Pressure | EUP | A | 0.88 | 0.1806 | 0.0274 | 4.26E-11 |
| rs1334576 | Pulse Pressure | EUP | A | 0.42 | -0.1149 | 0.0174 | 4.43E-11 |
| rs3191402 | Pulse Pressure | EUP | A | 0.40 | -0.1191 | 0.0181 | 5.16E-11 |
| rs115172170 | Pulse Pressure | EUP | T | 0.94 | -0.2459 | 0.0375 | 5.39E-11 |
| rs5750482 | Pulse Pressure | EUP | T | 0.38 | 0.1166 | 0.0178 | 5.40E-11 |
| rs13122790 | Pulse Pressure | EUP | A | 0.73 | 0.1283 | 0.0197 | 7.29E-11 |
| rs3135967 | Pulse Pressure | EUP | A | 0.52 | 0.1128 | 0.0173 | 7.56E-11 |
| rs6772704 | Pulse Pressure | EUP | A | 0.68 | -0.1236 | 0.0191 | 9.33E-11 |
| rs11615689 | Pulse Pressure | EUP | T | 0.82 | -0.1476 | 0.0228 | 9.48E-11 |
| rs11901929 | Pulse Pressure | EUP | A | 0.35 | 0.1184 | 0.0183 | 1.01E-10 |
| rs11789875 | Pulse Pressure | EUP | A | 0.15 | 0.1563 | 0.0242 | 1.15E-10 |
| rs7869756 | Pulse Pressure | EUP | A | 0.81 | -0.1446 | 0.0225 | 1.38E-10 |
| rs12172847 | Pulse Pressure | EUP | A | 0.32 | -0.1184 | 0.0185 | 1.49E-10 |
| rs134041 | Pulse Pressure | EUP | T | 0.43 | -0.1122 | 0.0176 | 1.71E-10 |
| rs4443403 | Pulse Pressure | EUP | T | 0.90 | -0.1832 | 0.0290 | 2.74E-10 |
| rs385437 | Pulse Pressure | EUP | A | 0.86 | 0.1552 | 0.0247 | 3.35E-10 |
| rs1432457 | Pulse Pressure | EUP | A | 0.72 | -0.1199 | 0.0191 | 3.59E-10 |
| rs77692990 | Pulse Pressure | EUP | T | 0.08 | -0.2014 | 0.0321 | 3.69E-10 |
| rs4652875 | Pulse Pressure | EUP | C | 0.42 | 0.1098 | 0.0177 | 5.04E-10 |
| rs6925750 | Pulse Pressure | EUP | T | 0.88 | 0.1657 | 0.0269 | 7.27E-10 |
| rs2277788 | Pulse Pressure | EUP | C | 0.10 | 0.1721 | 0.0282 | 9.87E-10 |
| rs12034319 | Pulse Pressure | EUP | A | 0.22 | 0.1278 | 0.0210 | 1.18E-09 |
| rs286809 | Pulse Pressure | EUP | A | 0.17 | -0.1391 | 0.0230 | 1.38E-09 |
| rs4342401 | Pulse Pressure | EUP | A | 0.54 | 0.1041 | 0.0174 | 2.02E-09 |
| rs7144602 | Pulse Pressure | EUP | T | 0.65 | -0.1094 | 0.0184 | 2.62E-09 |
| rs11642631 | Pulse Pressure | EUP | T | 0.56 | -0.1020 | 0.0174 | 4.26E-09 |
| rs66990951 | Pulse Pressure | EUP | T | 0.32 | 0.1075 | 0.0185 | 6.62E-09 |
| rs11760407 | Pulse Pressure | EUP | T | 0.25 | 0.1146 | 0.0200 | 1.04E-08 |
| rs2631669 | Pulse Pressure | EUP | T | 0.47 | 0.0974 | 0.0173 | 1.67E-08 |
| rs9833313 | Pulse Pressure | EUP | A | 0.24 | -0.1154 | 0.0205 | 1.80E-08 |
| rs4582532 | Pulse Pressure | EUP | A | 0.51 | -0.0966 | 0.0173 | 2.16E-08 |
| rs17119370 | Pulse Pressure | EUP | A | 0.69 | 0.1021 | 0.0187 | 4.99E-08 |
| rs780093 | Leptin | TRANS | C | 0.61 | 0.0240 | 0.0040 | 3.80E-10 |
| rs6738627 | Leptin | TRANS | A | 0.37 | 0.0200 | 0.0040 | 1.90E-06 |
| rs900400 | Leptin | TRANS | T | 0.60 | 0.0210 | 0.0040 | 1.20E-07 |
| rs10487505 | Leptin | TRANS | G | 0.50 | 0.0290 | 0.0040 | 2.00E-12 |
| rs6071166 | Leptin | TRANS | C | 0.37 | 0.0240 | 0.0040 | 1.80E-08 |
| rs12736689 | Morning Person | EUP | T | 0.97 | -0.3011 | 0.0345 | 7.00E-18 |
| rs9479402 | Morning Person | EUP | T | 0.99 | -0.3711 | 0.0553 | 3.90E-11 |
| rs55694368 | Morning Person | EUP | T | 0.07 | -0.1508 | 0.0269 | 2.60E-09 |
| rs35833281 | Morning Person | EUP | G | 0.79 | -0.0834 | 0.0138 | 9.20E-01 |
| rs11545787 | Morning Person | EUP | G | 0.76 | 0.0770 | 0.0142 | 1.40E-08 |
| rs11121022 | Morning Person | EUP | C | 0.42 | 0.0677 | 0.0120 | 2.00E-08 |
| rs9565309 | Morning Person | EUP | T | 0.97 | 0.1740 | 0.0300 | 3.50E-08 |
| rs1595824 | Morning Person | EUP | T | 0.49 | 0.0770 | 0.0119 | 1.20E-10 |
| rs34714364 | Morning Person | EUP | T | 0.17 | 0.1133 | 0.0182 | 2.00E-10 |
| rs3972456 | Morning Person | EUP | G | 0.71 | -0.0834 | 0.0139 | 6.00E-09 |
| rs12965577 | Morning Person | EUP | G | 0.34 | -0.0619 | 0.0109 | 2.10E-08 |
| rs12927162 | Morning Person | EUP | G | 0.26 | -0.0943 | 0.0139 | 1.60E-12 |
| rs10493596 | Morning Person | EUP | T | 0.24 | 0.0862 | 0.0117 | 8.00E-12 |
| rs2948276 | Morning Person | EUP | G | 0.18 | -0.0834 | 0.0166 | 1.10E-08 |
| rs6582618 | Morning Person | EUP | G | 0.52 | 0.0677 | 0.0120 | 1.50E-08 |
| rs12134663 | Homocysteine | EUP | A | 0.80 | -0.1010 | 0.0110 | 2.54E-21 |
| rs12780845 | Homocysteine | EUP | A | 0.65 | 0.0529 | 0.0090 | 7.80E-10 |
| rs12921383 | Homocysteine | EUP | T | 0.87 | -0.0900 | 0.0140 | 8.22E-11 |
| rs154657 | Homocysteine | EUP | A | 0.47 | 0.0963 | 0.0070 | 1.74E-43 |
| rs1801133 | Homocysteine | EUP | A | 0.34 | 0.1583 | 0.0070 | 4.34E-104 |

| rs1801222 | Homocysteine | EUP | A | 0.34 | 0.0453 | 0.0070 | 8.43E-10 |
| --- | --- | --- | --- | --- | --- | --- | --- |
| rs2251468 | Homocysteine | EUP | A | 0.65 | -0.0512 | 0.0070 | 1.28E-12 |
| rs2275565 | Homocysteine | EUP | T | 0.21 | -0.0542 | 0.0090 | 1.96E-10 |
| rs234709 | Homocysteine | EUP | T | 0.45 | -0.0718 | 0.0070 | 3.90E-24 |
| rs2851391 | Homocysteine | EUP | T | 0.47 | 0.0560 | 0.0080 | 1.70E-12 |
| rs42648 | Homocysteine | EUP | A | 0.40 | -0.0395 | 0.0070 | 1.97E-08 |
| rs4660306 | Homocysteine | EUP | T | 0.33 | 0.0435 | 0.0070 | 2.33E-09 |
| rs548987 | Homocysteine | EUP | C | 0.13 | 0.0597 | 0.0100 | 1.12E-08 |
| rs7130284 | Homocysteine | EUP | T | 0.07 | -0.1242 | 0.0130 | 1.88E-20 |
| rs7422339 | Homocysteine | EUP | A | 0.33 | 0.0864 | 0.0080 | 4.58E-10 |
| rs838133 | Homocysteine | EUP | A | 0.45 | 0.0422 | 0.0070 | 7.48E-09 |
| rs9369898 | Homocysteine | EUP | A | 0.62 | 0.0449 | 0.0070 | 2.17E-10 |
| rs957140 | Homocysteine | EUP | A | 0.45 | -0.0450 | 0.0080 | 2.43E-08 |
| rs1173771 | Hypertension | EUP | G | 0.60 | 0.0620 | 0.0099 | 3.23E-10 |
| rs1799945 | Hypertension | EUP | G | 0.14 | 0.0947 | 0.0148 | 1.76E-10 |
| rs805303 | Hypertension | EUP | G | 0.61 | 0.0537 | 0.0083 | 1.12E-10 |
| rs932764 | Hypertension | EUP | G | 0.44 | 0.0551 | 0.0096 | 9.35E-09 |
| rs633185 | Hypertension | EUP | G | 0.28 | -0.0702 | 0.0107 | 5.41E-11 |
| rs6015450 | Hypertension | EUP | G | 0.12 | 0.1104 | 0.0146 | 4.18E-14 |
| rs17367504 | Hypertension | EUP | G | 0.15 | -0.1031 | 0.0163 | 2.34E-10 |
| rs1813353 | Hypertension | EUP | T | 0.68 | 0.0778 | 0.0126 | 6.24E-10 |
| rs4590817 | Hypertension | EUP | G | 0.84 | 0.0961 | 0.0168 | 9.82E-09 |
| rs17249754 | Hypertension | EUP | G | 0.84 | 0.1261 | 0.0163 | 1.13E-14 |
| rs1378942 | Hypertension | EUP | C | 0.35 | 0.0726 | 0.0127 | 1.04E-08 |
| rs6743376 | Interleukin-1Ra | TRANS | A | 0.43 | 0.1300 | 0.0100 | 2.27E-26 |
| rs6761276 | Interleukin-1Ra | TRANS | C | 0.48 | 0.1200 | 0.0100 | 4.56E-25 |
| rs13386602 | Interleukin-1Ra | TRANS | A | 0.44 | 0.1300 | 0.0100 | 6.12E-25 |
| rs7980687 | Infant head circumference | EUP | A | 0.22 | 0.0740 | 0.0130 | 8.10E-09 |
| rs1042725 | Infant head circumference | EUP | T | 0.54 | -0.0650 | 0.0100 | 2.80E-10 |
| rs12221306 | Trimethylamine-N-Oxide | American | A | 0.39 | 0.1576 | 0.0345 | 5.04E-06 |
| rs13236413 | Trimethylamine-N-Oxide | American | C | 0.09 | 0.4082 | 0.0863 | 2.23E-06 |
| rs16917161 | Trimethylamine-N-Oxide | American | A | 0.09 | 0.2617 | 0.0590 | 9.23E-06 |
| rs17418675 | Trimethylamine-N-Oxide | American | T | 0.34 | 0.1558 | 0.0351 | 9.11E-06 |
| rs2703228 | Trimethylamine-N-Oxide | American | C | 0.32 | 0.2165 | 0.0410 | 1.28E-07 |
| rs3935570 | Trimethylamine-N-Oxide | American | G | 0.26 | 0.2271 | 0.0433 | 1.56E-07 |
| rs4846884 | Trimethylamine-N-Oxide | American | A | 0.16 | 0.2108 | 0.0466 | 6.19E-06 |
| rs692588 | Trimethylamine-N-Oxide | American | A | 0.03 | 2.7940 | 0.5767 | 1.27E-06 |
| rs7302178 | Trimethylamine-N-Oxide | American | A | 0.16 | 0.2371 | 0.0525 | 6.40E-06 |
| rs925735 | Adiponectin | TRANS | G | 0.64 | -0.0200 | 0.0040 | 2.12E-08 |
| rs2590838 | Adiponectin | TRANS | G | 0.51 | -0.0300 | 0.0040 | 1.88E-13 |
| rs6810075 | Adiponectin | TRANS | T | 0.93 | 0.0600 | 0.0040 | 2.41E-42 |
| rs2980879 | Adiponectin | TRANS | T | 0.69 | 0.0300 | 0.0040 | 9.91E-10 |
| rs2925979 | Adiponectin | TRANS | T | 0.30 | -0.0400 | 0.0040 | 3.12E-21 |
| rs12051272 | Adiponectin | TRANS | T | 0.03 | -0.2600 | 0.0320 | 1.10E-14 |
| rs4805885 | Adiponectin | TRANS | T | 0.39 | -0.0300 | 0.0050 | 2.05E-08 |
| rs12032996 | Albuminuria | EUP | G | 0.84 | 0.0146 | 0.0023 | 9.33E-11 |
| rs10157710 | Albuminuria | EUP | T | 0.80 | 0.0190 | 0.0021 | 9.69E-20 |
| rs11264327 | Albuminuria | EUP | A | 0.40 | 0.0099 | 0.0017 | 7.03E-09 |
| rs4665972 | Albuminuria | EUP | T | 0.39 | 0.0118 | 0.0017 | 6.96E-12 |
| rs13394343 | Albuminuria | EUP | C | 0.57 | 0.0105 | 0.0017 | 3.86E-10 |
| rs10207567 | Albuminuria | EUP | C | 0.81 | 0.0146 | 0.0021 | 1.00E-11 |
| rs1047891 | Albuminuria | EUP | C | 0.68 | 0.0121 | 0.0018 | 1.71E-11 |
| rs183131780 | Albuminuria | EUP | T | 0.00 | 0.1906 | 0.0196 | 2.33E-22 |
| rs35483183 | Albuminuria | EUP | A | 0.12 | 0.0149 | 0.0026 | 5.19E-09 |
| rs35924503 | Albuminuria | EUP | C | 0.00 | 0.2474 | 0.0252 | 8.68E-23 |
| rs112607182 | Albuminuria | EUP | T | 0.08 | 0.0228 | 0.0033 | 3.39E-12 |
| rs7654754 | Albuminuria | EUP | G | 0.46 | 0.0102 | 0.0017 | 9.96E-10 |
| rs6535594 | Albuminuria | EUP | A | 0.50 | 0.0115 | 0.0017 | 7.12E-12 |

| rs189107782 | Albuminuria | EUP | T | 0.00 | 0.2450 | 0.0203 | 1.12E-33 |
| --- | --- | --- | --- | --- | --- | --- | --- |
| rs702634 | Albuminuria | EUP | A | 0.69 | 0.0104 | 0.0018 | 8.03E-09 |
| rs7731168 | Albuminuria | EUP | C | 0.23 | 0.0125 | 0.0020 | 2.19E-10 |
| rs4410790 | Albuminuria | EUP | C | 0.63 | 0.0180 | 0.0017 | 2.63E-25 |
| rs2023844 | Albuminuria | EUP | A | 0.93 | 0.0193 | 0.0032 | 1.18E-09 |
| rs17158386 | Albuminuria | EUP | A | 0.26 | 0.0133 | 0.0019 | 3.65E-12 |
| rs28601761 | Albuminuria | EUP | C | 0.58 | 0.0114 | 0.0017 | 2.81E-11 |
| rs45551835 | Albuminuria | EUP | A | 0.01 | 0.1424 | 0.0070 | 2.28E-92 |
| rs144360241 | Albuminuria | EUP | C | 0.01 | 0.0819 | 0.0123 | 3.31E-11 |
| rs1276720 | Albuminuria | EUP | T | 0.75 | 0.0111 | 0.0019 | 8.98E-09 |
| rs141640975 | Albuminuria | EUP | A | 0.00 | 0.3588 | 0.0163 | 1.75E-107 |
| rs67339103 | Albuminuria | EUP | A | 0.21 | 0.0152 | 0.0021 | 1.07E-13 |
| rs17368443 | Albuminuria | EUP | C | 0.06 | 0.0207 | 0.0035 | 2.58E-09 |
| rs2601006 | Albuminuria | EUP | C | 0.66 | 0.0118 | 0.0018 | 2.13E-11 |
| rs4288924 | Albuminuria | EUP | G | 0.48 | 0.0098 | 0.0017 | 5.66E-09 |
| rs8035855 | Albuminuria | EUP | A | 0.64 | 0.0123 | 0.0017 | 1.91E-12 |
| rs1145074 | Albuminuria | EUP | T | 0.75 | 0.0114 | 0.0019 | 2.41E-09 |
| rs2472297 | Albuminuria | EUP | T | 0.27 | 0.0181 | 0.0019 | 5.31E-22 |
| rs35572189 | Albuminuria | EUP | G | 0.64 | 0.0105 | 0.0017 | 1.44E-09 |
| rs838142 | Albuminuria | EUP | A | 0.72 | 0.0117 | 0.0019 | 3.13E-10 |
| rs705687 | Alcohol intake | TRANS | G | 0.22 | -0.0109 | 0.0018 | 8.15E-10 |
| rs58107686 | Alcohol intake | TRANS | A | 0.33 | -0.0097 | 0.0016 | 7.79E-10 |
| rs12088813 | Alcohol intake | TRANS | C | 0.27 | -0.0093 | 0.0016 | 1.58E-08 |
| rs5024204 | Alcohol intake | TRANS | T | 0.28 | 0.0097 | 0.0016 | 2.55E-09 |
| rs10753661 | Alcohol intake | TRANS | A | 0.32 | -0.0086 | 0.0016 | 3.76E-08 |
| rs28680958 | Alcohol intake | TRANS | A | 0.22 | -0.0110 | 0.0018 | 5.13E-10 |
| rs823114 | Alcohol intake | TRANS | A | 0.45 | 0.0088 | 0.0015 | 2.31E-09 |
| rs77165542 | Alcohol intake | TRANS | T | 0.03 | -0.0260 | 0.0040 | 5.63E-11 |
| rs1260326 | Alcohol intake | TRANS | C | 0.40 | 0.0209 | 0.0015 | 8.05E-45 |
| rs2178197 | Alcohol intake | TRANS | G | 0.43 | -0.0088 | 0.0015 | 2.45E-09 |
| rs13383034 | Alcohol intake | TRANS | T | 0.33 | 0.0149 | 0.0016 | 6.31E-22 |
| rs1004787 | Alcohol intake | TRANS | A | 0.45 | 0.0084 | 0.0015 | 8.40E-09 |
| rs13032049 | Alcohol intake | TRANS | G | 0.28 | 0.0102 | 0.0016 | 3.00E-10 |
| rs828867 | Alcohol intake | TRANS | A | 0.46 | 0.0088 | 0.0015 | 2.15E-09 |
| rs11692435 | Alcohol intake | TRANS | A | 0.09 | 0.0174 | 0.0026 | 2.53E-11 |
| rs13024996 | Alcohol intake | TRANS | A | 0.36 | -0.0109 | 0.0015 | 5.72E-13 |
| rs72859280 | Alcohol intake | TRANS | T | 0.04 | 0.0229 | 0.0039 | 4.44E-09 |
| rs56337305 | Alcohol intake | TRANS | C | 0.38 | -0.0096 | 0.0015 | 1.63E-10 |
| rs13094887 | Alcohol intake | TRANS | T | 0.30 | -0.0103 | 0.0016 | 8.57E-11 |
| rs62250685 | Alcohol intake | TRANS | G | 0.39 | -0.0144 | 0.0015 | 1.05E-21 |
| rs13066454 | Alcohol intake | TRANS | T | 0.40 | -0.0088 | 0.0015 | 4.13E-09 |
| rs9838144 | Alcohol intake | TRANS | C | 0.21 | -0.0100 | 0.0018 | 2.65E-08 |
| rs2011092 | Alcohol intake | TRANS | C | 0.34 | -0.0089 | 0.0015 | 7.35E-09 |
| rs60654199 | Alcohol intake | TRANS | A | 0.06 | -0.0167 | 0.0030 | 2.85E-08 |
| rs6787172 | Alcohol intake | TRANS | G | 0.45 | -0.0080 | 0.0015 | 4.27E-08 |
| rs3748034 | Alcohol intake | TRANS | T | 0.14 | -0.0117 | 0.0021 | 1.67E-08 |
| rs7682824 | Alcohol intake | TRANS | T | 0.46 | 0.0084 | 0.0015 | 2.77E-08 |
| rs11940694 | Alcohol intake | TRANS | G | 0.40 | 0.0259 | 0.0015 | 3.03E-68 |
| rs4501255 | Alcohol intake | TRANS | G | 0.24 | 0.0107 | 0.0017 | 4.83E-10 |
| rs12499107 | Alcohol intake | TRANS | G | 0.13 | 0.0127 | 0.0022 | 4.45E-09 |
| rs144198753 | Alcohol intake | TRANS | T | 0.02 | -0.0418 | 0.0059 | 1.35E-12 |
| rs1154414 | Alcohol intake | TRANS | C | 0.14 | 0.0176 | 0.0021 | 3.74E-17 |
| rs1229984 | Alcohol intake | TRANS | C | 0.04 | 0.1505 | 0.0039 | 2.20E-208 |
| rs10028756 | Alcohol intake | TRANS | A | 0.13 | -0.0186 | 0.0022 | 1.16E-17 |
| rs561222871 | Alcohol intake | TRANS | T | 0.05 | -0.0388 | 0.0036 | 6.56E-27 |
| rs36052336 | Alcohol intake | TRANS | G | 0.06 | -0.0184 | 0.0030 | 1.23E-09 |
| rs2165670 | Alcohol intake | TRANS | A | 0.11 | 0.0231 | 0.0024 | 1.67E-22 |
| rs79139602 | Alcohol intake | TRANS | T | 0.02 | 0.0603 | 0.0051 | 1.80E-32 |

| rs4699791 | Alcohol intake | TRANS | A | 0.10 | 0.0186 | 0.0025 | 6.58E-14 |
| --- | --- | --- | --- | --- | --- | --- | --- |
| rs13107325 | Alcohol intake | TRANS | T | 0.07 | -0.0275 | 0.0028 | 1.53E-22 |
| rs4690727 | Alcohol intake | TRANS | G | 0.28 | 0.0108 | 0.0016 | 2.43E-11 |
| rs10004020 | Alcohol intake | TRANS | A | 0.28 | 0.0091 | 0.0016 | 2.43E-08 |
| rs12651313 | Alcohol intake | TRANS | G | 0.44 | -0.0086 | 0.0015 | 3.79E-09 |
| rs4916723 | Alcohol intake | TRANS | C | 0.42 | -0.0100 | 0.0015 | 1.72E-11 |
| rs12655091 | Alcohol intake | TRANS | A | 0.47 | -0.0083 | 0.0015 | 1.25E-08 |
| rs55872084 | Alcohol intake | TRANS | T | 0.24 | 0.0100 | 0.0017 | 6.32E-09 |
| rs11739827 | Alcohol intake | TRANS | T | 0.45 | -0.0084 | 0.0015 | 1.18E-08 |
| rs10085696 | Alcohol intake | TRANS | G | 0.19 | -0.0114 | 0.0019 | 1.12E-09 |
| rs6460047 | Alcohol intake | TRANS | C | 0.21 | 0.0116 | 0.0018 | 9.69E-11 |
| rs10236149 | Alcohol intake | TRANS | G | 0.12 | -0.0135 | 0.0022 | 1.18E-09 |
| rs35034355 | Alcohol intake | TRANS | A | 0.48 | -0.0081 | 0.0015 | 2.87E-08 |
| rs6951574 | Alcohol intake | TRANS | C | 0.46 | 0.0132 | 0.0015 | 1.58E-19 |
| rs13250583 | Alcohol intake | TRANS | T | 0.21 | -0.0097 | 0.0018 | 4.70E-08 |
| rs1217091 | Alcohol intake | TRANS | C | 0.19 | 0.0122 | 0.0019 | 7.05E-11 |
| rs28601761 | Alcohol intake | TRANS | G | 0.42 | 0.0091 | 0.0015 | 7.17E-10 |
| rs55932213 | Alcohol intake | TRANS | G | 0.26 | 0.0095 | 0.0017 | 9.55E-09 |
| rs10978550 | Alcohol intake | TRANS | C | 0.21 | -0.0117 | 0.0018 | 7.15E-11 |
| rs7074871 | Alcohol intake | TRANS | A | 0.26 | -0.0094 | 0.0017 | 1.86E-08 |
| rs17665139 | Alcohol intake | TRANS | T | 0.15 | -0.0116 | 0.0020 | 1.59E-08 |
| rs7950166 | Alcohol intake | TRANS | T | 0.36 | -0.0098 | 0.0015 | 9.89E-11 |
| rs11030084 | Alcohol intake | TRANS | T | 0.18 | -0.0106 | 0.0019 | 1.72E-08 |
| rs56030824 | Alcohol intake | TRANS | A | 0.32 | -0.0116 | 0.0016 | 1.15E-13 |
| rs10750025 | Alcohol intake | TRANS | T | 0.31 | 0.0103 | 0.0016 | 4.89E-11 |
| rs1713676 | Alcohol intake | TRANS | G | 0.48 | -0.0080 | 0.0015 | 4.29E-08 |
| rs4938230 | Alcohol intake | TRANS | A | 0.16 | 0.0128 | 0.0020 | 1.48E-10 |
| rs682011 | Alcohol intake | TRANS | C | 0.44 | 0.0082 | 0.0015 | 2.22E-08 |
| rs12795042 | Alcohol intake | TRANS | C | 0.38 | -0.0083 | 0.0015 | 3.25E-08 |
| rs10876188 | Alcohol intake | TRANS | T | 0.46 | -0.0080 | 0.0015 | 4.84E-08 |
| rs3809162 | Alcohol intake | TRANS | G | 0.40 | 0.0091 | 0.0015 | 1.19E-09 |
| rs10506274 | Alcohol intake | TRANS | T | 0.48 | -0.0090 | 0.0015 | 5.78E-10 |
| rs4842786 | Alcohol intake | TRANS | A | 0.42 | -0.0088 | 0.0015 | 2.73E-09 |
| rs500321 | Alcohol intake | TRANS | T | 0.26 | -0.0097 | 0.0017 | 4.92E-09 |
| rs1123285 | Alcohol intake | TRANS | G | 0.34 | -0.0089 | 0.0015 | 8.14E-09 |
| rs2180870 | Alcohol intake | TRANS | C | 0.14 | -0.0122 | 0.0021 | 1.12E-08 |
| rs28929474 | Alcohol intake | TRANS | T | 0.02 | -0.0368 | 0.0054 | 1.34E-11 |
| rs11625650 | Alcohol intake | TRANS | A | 0.23 | -0.0096 | 0.0017 | 2.89E-08 |
| rs2472297 | Alcohol intake | TRANS | T | 0.25 | 0.0106 | 0.0017 | 3.10E-10 |
| rs12907323 | Alcohol intake | TRANS | G | 0.41 | 0.0085 | 0.0015 | 9.93E-09 |
| rs2764771 | Alcohol intake | TRANS | A | 0.31 | 0.0099 | 0.0016 | 4.02E-10 |
| rs17177078 | Alcohol intake | TRANS | T | 0.06 | -0.0223 | 0.0030 | 1.27E-13 |
| rs378421 | Alcohol intake | TRANS | A | 0.40 | -0.0112 | 0.0015 | 4.83E-14 |
| rs113443718 | Alcohol intake | TRANS | A | 0.31 | -0.0102 | 0.0016 | 1.19E-10 |
| rs62044525 | Alcohol intake | TRANS | G | 0.18 | -0.0122 | 0.0019 | 1.03E-10 |
| rs7185555 | Alcohol intake | TRANS | C | 0.15 | -0.0111 | 0.0020 | 4.24E-08 |
| rs79616692 | Alcohol intake | TRANS | C | 0.11 | 0.0163 | 0.0024 | 4.11E-12 |
| rs1104608 | Alcohol intake | TRANS | C | 0.43 | -0.0110 | 0.0015 | 1.05E-13 |
| rs4548913 | Alcohol intake | TRANS | A | 0.37 | -0.0084 | 0.0015 | 3.11E-08 |
| rs3803800 | Alcohol intake | TRANS | G | 0.21 | 0.0114 | 0.0018 | 1.50E-10 |
| rs2854334 | Alcohol intake | TRANS | G | 0.39 | 0.0092 | 0.0015 | 7.51E-10 |
| rs10438820 | Alcohol intake | TRANS | T | 0.30 | 0.0090 | 0.0016 | 1.76E-08 |
| rs9950000 | Alcohol intake | TRANS | T | 0.40 | -0.0091 | 0.0015 | 9.38E-10 |
| rs4092465 | Alcohol intake | TRANS | G | 0.37 | -0.0083 | 0.0015 | 4.39E-08 |
| rs281379 | Alcohol intake | TRANS | A | 0.49 | 0.0137 | 0.0015 | 4.91E-21 |
| rs4815364 | Alcohol intake | TRANS | A | 0.38 | 0.0086 | 0.0015 | 1.02E-08 |
| rs9607814 | Alcohol intake | TRANS | A | 0.20 | -0.0102 | 0.0019 | 4.31E-08 |
| rs10178297 | Betaine | American | C | 0.16 | 0.2068 | 0.0459 | 6.74E-06 |

| rs10786317 | Betaine | American | A | 0.44 | 0.1580 | 0.0345 | 4.72E-06 |
| --- | --- | --- | --- | --- | --- | --- | --- |
| rs10817686 | Betaine | American | C | 0.47 | 0.4561 | 0.0952 | 1.68E-06 |
| rs10883712 | Betaine | American | T | 0.40 | 0.1592 | 0.0345 | 4.02E-06 |
| rs11030909 | Betaine | American | A | 0.09 | 0.2885 | 0.0643 | 7.30E-06 |
| rs11224038 | Betaine | American | T | 0.01 | 1.2107 | 0.2509 | 1.40E-06 |
| rs11742447 | Betaine | American | T | 0.34 | 0.2460 | 0.0506 | 1.16E-06 |
| rs1321958 | Betaine | American | A | 0.39 | 0.1525 | 0.0342 | 8.12E-06 |
| rs17815398 | Betaine | American | G | 0.02 | 0.8380 | 0.1867 | 7.19E-06 |
| rs1868264 | Betaine | American | G | 0.20 | 0.1920 | 0.0420 | 4.92E-06 |
| rs2087307 | Betaine | American | T | 0.26 | 0.1720 | 0.0387 | 8.63E-06 |
| rs2879414 | Betaine | American | G | 0.46 | 0.1526 | 0.0334 | 5.03E-06 |
| rs358538 | Betaine | American | G | 0.05 | 0.4559 | 0.1020 | 7.86E-06 |
| rs6862283 | Betaine | American | C | 0.31 | 0.1926 | 0.0361 | 9.57E-08 |
| rs9587723 | Betaine | American | C | 0.00 | 3.2835 | 0.7245 | 5.84E-06 |
| rs10821590 | Carnitine | American | A | 0.07 | 0.5984 | 0.1119 | 8.99E-08 |
| rs1171610 | Carnitine | American | C | 0.38 | 0.1951 | 0.0361 | 6.63E-08 |
| rs1171617 | Carnitine | American | T | 0.23 | 0.4175 | 0.0396 | 5.87E-26 |
| rs11733138 | Carnitine | American | A | 0.35 | 0.2251 | 0.0498 | 6.10E-06 |
| rs16913790 | Carnitine | American | A | 0.14 | 0.2156 | 0.0480 | 7.18E-06 |
| rs274554 | Carnitine | American | T | 0.16 | 0.2108 | 0.0460 | 4.68E-06 |
| rs4656852 | Carnitine | American | C | 0.05 | 0.5076 | 0.1139 | 8.30E-06 |
| rs562672 | Carnitine | American | C | 0.37 | 0.2337 | 0.0521 | 7.39E-06 |
| rs6108228 | Carnitine | American | T | 0.41 | 0.1752 | 0.0373 | 2.56E-06 |
| rs6959875 | Carnitine | American | G | 0.30 | 0.2702 | 0.0546 | 7.58E-07 |
| rs7606454 | Carnitine | American | T | 0.14 | 0.2349 | 0.0531 | 9.62E-06 |
| rs9879988 | Carnitine | American | G | 0.16 | 0.2391 | 0.0467 | 3.00E-07 |
| rs17398575 | Carotid Artery Plaque | TRANS | A | 0.25 | 0.1655 | 0.0239 | 2.30E-12 |
| rs1878406 | Carotid Artery Plaque | TRANS | T | 0.13 | 0.1989 | 0.0293 | 6.90E-12 |
| rs10098425 | Choline | American | A | 0.05 | 0.4586 | 0.1033 | 8.93E-06 |
| rs10819950 | Choline | American | A | 0.32 | 0.2078 | 0.0462 | 6.95E-06 |
| rs10826197 | Choline | American | C | 0.49 | 0.1515 | 0.0339 | 8.15E-06 |
| rs12444044 | Choline | American | G | 0.11 | 0.3641 | 0.0803 | 5.77E-06 |
| rs2408564 | Choline | American | A | 0.17 | 0.2491 | 0.0516 | 1.41E-06 |
| rs4008155 | Choline | American | C | 0.16 | 0.2154 | 0.0467 | 4.02E-06 |
| rs4312185 | Choline | American | A | 0.43 | 0.1559 | 0.0347 | 7.13E-06 |
| rs9599459 | Choline | American | T | 0.01 | 1.0154 | 0.2165 | 2.73E-06 |
| rs1260326 | Coffee Intake | TRANS | T | 0.41 | -0.0400 | 0.0100 | 7.14E-08 |
| rs1481012 | Coffee Intake | TRANS | A | 0.89 | 0.0600 | 0.0100 | 8.93E-08 |
| rs4410790 | Coffee Intake | TRANS | T | 0.37 | -0.1000 | 0.0100 | 3.08E-17 |
| rs6968554 | Coffee Intake | TRANS | A | 0.39 | -0.1000 | 0.0100 | 5.23E-17 |
| rs7800944 | Coffee Intake | TRANS | T | 0.72 | -0.0500 | 0.0100 | 2.29E-11 |
| rs17685 | Coffee Intake | TRANS | A | 0.29 | 0.0700 | 0.0100 | 4.26E-11 |
| rs6265 | Coffee Intake | TRANS | T | 0.19 | -0.0400 | 0.0100 | 2.69E-06 |
| rs2470893 | Coffee Intake | TRANS | T | 0.31 | 0.1200 | 0.0100 | 2.72E-19 |
| rs2472297 | Coffee Intake | TRANS | T | 0.24 | 0.1400 | 0.0100 | 2.47E-24 |
| rs9902453 | Coffee Intake | TRANS | A | 0.54 | -0.0300 | 0.0100 | 2.44E-08 |
| rs11781551 | Carotid Intima Media Thickness | TRANS | A | 0.48 | -0.0078 | 0.0012 | 2.40E-11 |
| rs445925 | Carotid Intima Media Thickness | TRANS | A | 0.11 | -0.0156 | 0.0028 | 1.70E-08 |
| rs6601530 | Carotid Intima Media Thickness | TRANS | G | 0.45 | 0.0078 | 0.0014 | 1.70E-08 |
| rs1998598 | Crohn's Disease | EUP | G | 0.30 | 0.0392 | 0.0220 | 8.70E-09 |
| rs13428812 | Crohn's Disease | EUP | G | 0.33 | 0.0583 | 0.0168 | 8.50E-10 |
| rs780093 | Crohn's Disease | EUP | T | 0.42 | 0.1398 | 0.0243 | 4.70E-11 |
| rs10495903 | Crohn's Disease | EUP | T | 0.13 | 0.1310 | 0.0245 | 1.60E-14 |
| rs10181042 | Crohn's Disease | EUP | T | 0.42 | 0.1310 | 0.0224 | 6.61E-09 |
| rs2058660 | Crohn's Disease | EUP | G | 0.23 | 0.1740 | 0.0255 | 1.58E-12 |
| rs6738825 | Crohn's Disease | EUP | A | 0.47 | 0.0583 | 0.0216 | 3.50E-09 |
| rs7423615 | Crohn's Disease | EUP | T | 0.19 | 0.1133 | 0.0250 | 3.10E-13 |
| rs13073817 | Crohn's Disease | EUP | A | 0.32 | 0.0770 | 0.0236 | 6.70E-09 |

| rs7702331 | Crohn's Disease | EUP | A | 0.60 | 0.1133 | 0.0228 | 5.90E-12 |
| --- | --- | --- | --- | --- | --- | --- | --- |
| rs2549794 | Crohn's Disease | EUP | C | 0.41 | 0.0488 | 0.0169 | 1.10E-10 |
| rs11167764 | Crohn's Disease | EUP | C | 0.80 | 0.0583 | 0.0216 | 2.00E-09 |
| rs359457 | Crohn's Disease | EUP | T | 0.57 | 0.0770 | 0.0189 | 2.50E-12 |
| rs17309827 | Crohn's Disease | EUP | T | 0.64 | 0.0953 | 0.0254 | 6.70E-09 |
| rs6651252 | Crohn's Disease | EUP | T | 0.87 | 0.2070 | 0.0269 | 3.90E-18 |
| rs4077515 | Crohn's Disease | EUP | T | 0.41 | 0.1655 | 0.0195 | 1.30E-36 |
| rs12722489 | Crohn's Disease | EUP | C | 0.85 | 0.1044 | 0.0254 | 2.90E-09 |
| rs1819658 | Crohn's Disease | EUP | C | 0.77 | 0.1740 | 0.0257 | 9.10E-17 |
| rs102275 | Crohn's Disease | EUP | C | 0.34 | 0.0770 | 0.0189 | 2.30E-11 |
| rs694739 | Crohn's Disease | EUP | A | 0.63 | 0.0953 | 0.0254 | 6.00E-10 |
| rs2062305 | Crohn's Disease | EUP | G | 0.35 | 0.0953 | 0.0232 | 4.90E-10 |
| rs4902642 | Crohn's Disease | EUP | G | 0.58 | 0.0677 | 0.0166 | 1.60E-10 |
| rs8005161 | Crohn's Disease | EUP | T | 0.12 | 0.2070 | 0.0310 | 4.20E-18 |
| rs17293632 | Crohn's Disease | EUP | T | 0.23 | 0.1133 | 0.0206 | 2.70E-19 |
| rs3091315 | Crohn's Disease | EUP | A | 0.72 | 0.1823 | 0.0255 | 1.70E-13 |
| rs736289 | Crohn's Disease | EUP | T | 0.61 | 0.0583 | 0.0216 | 8.70E-09 |
| rs281379 | Crohn's Disease | EUP | A | 0.49 | 0.0677 | 0.0166 | 7.40E-12 |
| rs4809330 | Crohn's Disease | EUP | G | 0.71 | 0.1133 | 0.0274 | 2.70E-15 |
| rs713875 | Crohn's Disease | EUP | C | 0.47 | 0.0770 | 0.0212 | 7.30E-12 |
| rs2413583 | Crohn's Disease | EUP | C | 0.83 | 0.2070 | 0.0249 | 1.10E-26 |
| rs11209026 | Crohn's Disease | EUP | G | 0.93 | 0.9783 | 0.0612 | 1.00E-64 |
| rs2476601 | Crohn's Disease | EUP | G | 0.91 | 0.2311 | 0.0403 | 4.47E-09 |
| rs4656940 | Crohn's Disease | EUP | A | 0.80 | 0.1398 | 0.0266 | 6.17E-07 |
| rs7517810 | Crohn's Disease | EUP | T | 0.25 | 0.1989 | 0.0251 | 1.51E-15 |
| rs7554511 | Crohn's Disease | EUP | C | 0.73 | 0.1310 | 0.0247 | 1.58E-07 |
| rs3792109 | Crohn's Disease | EUP | A | 0.53 | 0.2927 | 0.0209 | 6.76E-41 |
| rs3197999 | Crohn's Disease | EUP | A | 0.30 | 0.1989 | 0.0231 | 6.17E-17 |
| rs11742570 | Crohn's Disease | EUP | C | 0.61 | 0.2852 | 0.0230 | 7.08E-36 |
| rs12521868 | Crohn's Disease | EUP | T | 0.42 | 0.2070 | 0.0208 | 1.41E-20 |
| rs7714584 | Crohn's Disease | EUP | G | 0.09 | 0.3148 | 0.0353 | 7.76E-19 |
| rs6556412 | Crohn's Disease | EUP | A | 0.33 | 0.1655 | 0.0237 | 5.37E-14 |
| rs6908425 | Crohn's Disease | EUP | C | 0.78 | 0.1570 | 0.0262 | 1.41E-08 |
| rs1799964 | Crohn's Disease | EUP | C | 0.21 | 0.1740 | 0.0257 | 3.98E-11 |
| rs6568421 | Crohn's Disease | EUP | G | 0.30 | 0.1222 | 0.0250 | 4.37E-08 |
| rs415890 | Crohn's Disease | EUP | C | 0.52 | 0.1570 | 0.0218 | 2.51E-12 |
| rs1456896 | Crohn's Disease | EUP | T | 0.69 | 0.1310 | 0.0245 | 1.20E-08 |
| rs4871611 | Crohn's Disease | EUP | A | 0.61 | 0.1570 | 0.0239 | 1.51E-12 |
| rs10758669 | Crohn's Disease | EUP | C | 0.35 | 0.1655 | 0.0216 | 1.00E-13 |
| rs3810936 | Crohn's Disease | EUP | C | 0.68 | 0.1906 | 0.0253 | 1.00E-15 |
| rs12242110 | Crohn's Disease | EUP | G | 0.32 | 0.1398 | 0.0222 | 1.10E-09 |
| rs10761659 | Crohn's Disease | EUP | G | 0.54 | 0.2070 | 0.0227 | 4.37E-22 |
| rs4409764 | Crohn's Disease | EUP | T | 0.49 | 0.1989 | 0.0209 | 2.29E-20 |
| rs7927997 | Crohn's Disease | EUP | T | 0.39 | 0.1570 | 0.0218 | 5.62E-13 |
| rs11564258 | Crohn's Disease | EUP | A | 0.03 | 0.5539 | 0.0586 | 6.17E-21 |
| rs3764147 | Crohn's Disease | EUP | G | 0.25 | 0.1570 | 0.0239 | 1.41E-10 |
| rs2076756 | Crohn's Disease | EUP | G | 0.26 | 0.4253 | 0.0234 | 3.98E-69 |
| rs2872507 | Crohn's Disease | EUP | A | 0.46 | 0.1310 | 0.0224 | 1.51E-09 |
| rs11871801 | Crohn's Disease | EUP | A | 0.76 | 0.1398 | 0.0243 | 2.51E-08 |
| rs1893217 | Crohn's Disease | EUP | G | 0.15 | 0.2231 | 0.0286 | 1.29E-14 |
| rs740495 | Crohn's Disease | EUP | G | 0.25 | 0.1484 | 0.0243 | 8.13E-12 |
| rs1736020 | Crohn's Disease | EUP | C | 0.58 | 0.1484 | 0.0220 | 9.33E-12 |
| rs2838519 | Crohn's Disease | EUP | G | 0.39 | 0.1655 | 0.0216 | 2.09E-14 |
| rs469772 | C-Reactive Protein | EUP | T | 0.19 | -0.0310 | 0.0050 | 5.54E-12 |
| rs12995480 | C-Reactive Protein | EUP | T | 0.17 | -0.0310 | 0.0050 | 1.24E-10 |
| rs4246598 | C-Reactive Protein | EUP | A | 0.46 | 0.0220 | 0.0040 | 5.11E-10 |
| rs9284725 | C-Reactive Protein | EUP | C | 0.24 | 0.0270 | 0.0040 | 7.34E-11 |
| rs1441169 | C-Reactive Protein | EUP | G | 0.53 | -0.0250 | 0.0040 | 2.27E-11 |

| rs2352975 | C-Reactive Protein | EUP | C | 0.30 | 0.0250 | 0.0040 | 6.43E-10 |
| --- | --- | --- | --- | --- | --- | --- | --- |
| rs17658229 | C-Reactive Protein | EUP | C | 0.05 | 0.0560 | 0.0100 | 5.50E-09 |
| rs9271608 | C-Reactive Protein | EUP | G | 0.22 | 0.0420 | 0.0050 | 2.33E-17 |
| rs12202641 | C-Reactive Protein | EUP | T | 0.39 | -0.0230 | 0.0040 | 3.00E-10 |
| rs1490384 | C-Reactive Protein | EUP | T | 0.51 | -0.0250 | 0.0040 | 2.65E-12 |
| rs9385532 | C-Reactive Protein | EUP | T | 0.33 | -0.0260 | 0.0040 | 1.90E-11 |
| rs1880241 | C-Reactive Protein | EUP | G | 0.48 | -0.0280 | 0.0040 | 8.41E-14 |
| rs2710804 | C-Reactive Protein | EUP | C | 0.37 | 0.0210 | 0.0040 | 1.30E-08 |
| rs2064009 | C-Reactive Protein | EUP | C | 0.42 | -0.0270 | 0.0040 | 2.28E-14 |
| rs2891677 | C-Reactive Protein | EUP | C | 0.46 | -0.0200 | 0.0040 | 1.59E-08 |
| rs643434 | C-Reactive Protein | EUP | A | 0.37 | 0.0230 | 0.0040 | 1.02E-09 |
| rs1051338 | C-Reactive Protein | EUP | G | 0.31 | 0.0240 | 0.0040 | 2.27E-09 |
| rs10832027 | C-Reactive Protein | EUP | G | 0.33 | -0.0260 | 0.0040 | 4.43E-12 |
| rs10838687 | C-Reactive Protein | EUP | G | 0.22 | -0.0310 | 0.0040 | 9.12E-13 |
| rs1582763 | C-Reactive Protein | EUP | A | 0.37 | -0.0220 | 0.0040 | 2.37E-09 |
| rs7121935 | C-Reactive Protein | EUP | A | 0.38 | -0.0220 | 0.0040 | 5.28E-09 |
| rs11108056 | C-Reactive Protein | EUP | G | 0.42 | -0.0280 | 0.0040 | 5.42E-14 |
| rs2239222 | C-Reactive Protein | EUP | G | 0.36 | 0.0350 | 0.0040 | 9.87E-20 |
| rs4774590 | C-Reactive Protein | EUP | A | 0.35 | -0.0220 | 0.0040 | 2.71E-08 |
| rs1558902 | C-Reactive Protein | EUP | A | 0.41 | 0.0340 | 0.0040 | 5.20E-20 |
| rs178810 | C-Reactive Protein | EUP | T | 0.56 | 0.0200 | 0.0040 | 2.95E-08 |
| rs10512597 | C-Reactive Protein | EUP | T | 0.18 | -0.0370 | 0.0050 | 4.44E-14 |
| rs4092465 | C-Reactive Protein | EUP | A | 0.35 | -0.0270 | 0.0040 | 3.11E-10 |
| rs12960928 | C-Reactive Protein | EUP | C | 0.27 | 0.0240 | 0.0040 | 1.91E-09 |
| rs2315008 | C-Reactive Protein | EUP | T | 0.31 | -0.0230 | 0.0040 | 5.36E-10 |
| rs2836878 | C-Reactive Protein | EUP | G | 0.27 | 0.0430 | 0.0040 | 7.71E-26 |
| rs6001193 | C-Reactive Protein | EUP | G | 0.35 | -0.0280 | 0.0040 | 6.53E-14 |
| rs75460349 | C-Reactive Protein | EUP | A | 0.97 | 0.0860 | 0.0140 | 4.50E-10 |
| rs1514895 | C-Reactive Protein | EUP | A | 0.71 | -0.0270 | 0.0040 | 2.70E-09 |
| rs112635299 | C-Reactive Protein | EUP | T | 0.02 | -0.1070 | 0.0170 | 2.10E-10 |
| rs1189402 | C-Reactive Protein | EUP | A | 0.62 | 0.0250 | 0.0040 | 3.90E-09 |
| rs687339 | C-Reactive Protein | EUP | T | 0.78 | -0.0300 | 0.0050 | 2.80E-10 |
| rs7795281 | C-Reactive Protein | EUP | A | 0.76 | 0.0280 | 0.0050 | 3.10E-08 |
| rs1736060 | C-Reactive Protein | EUP | T | 0.60 | 0.0290 | 0.0040 | 2.60E-13 |
| rs9611441 | C-Reactive Protein | EUP | C | 0.49 | -0.0220 | 0.0040 | 1.40E-08 |
| rs74748843 | estimated Glomerular Filtration Rate | TRANS | T | 0.07 | -0.0048 | 0.0008 | 3.69E-09 |
| rs10159261 | estimated Glomerular Filtration Rate | TRANS | T | 0.36 | -0.0034 | 0.0003 | 4.84E-25 |
| rs34966560 | estimated Glomerular Filtration Rate | TRANS | T | 0.29 | -0.0023 | 0.0003 | 2.49E-11 |
| rs12061708 | estimated Glomerular Filtration Rate | TRANS | A | 0.29 | -0.0026 | 0.0003 | 9.63E-14 |
| rs2749153 | estimated Glomerular Filtration Rate | TRANS | A | 0.69 | -0.0033 | 0.0003 | 7.84E-23 |
| rs659437 | estimated Glomerular Filtration Rate | TRANS | T | 0.22 | -0.0027 | 0.0004 | 3.32E-12 |
| rs11211257 | estimated Glomerular Filtration Rate | TRANS | A | 0.82 | 0.0027 | 0.0005 | 2.34E-09 |
| rs688540 | estimated Glomerular Filtration Rate | TRANS | A | 0.87 | -0.0030 | 0.0005 | 2.95E-08 |
| rs17413465 | estimated Glomerular Filtration Rate | TRANS | A | 0.18 | 0.0025 | 0.0004 | 8.93E-09 |
| rs1757915 | estimated Glomerular Filtration Rate | TRANS | A | 0.33 | 0.0021 | 0.0003 | 2.95E-10 |
| rs7536433 | estimated Glomerular Filtration Rate | TRANS | T | 0.26 | 0.0021 | 0.0004 | 6.66E-09 |
| rs679843 | estimated Glomerular Filtration Rate | TRANS | T | 0.33 | 0.0021 | 0.0003 | 5.21E-10 |
| rs1887252 | estimated Glomerular Filtration Rate | TRANS | C | 0.62 | -0.0019 | 0.0003 | 2.92E-09 |
| rs7543734 | estimated Glomerular Filtration Rate | TRANS | C | 0.21 | 0.0031 | 0.0005 | 9.90E-11 |
| rs11166440 | estimated Glomerular Filtration Rate | TRANS | A | 0.60 | 0.0020 | 0.0003 | 1.84E-10 |
| rs10857788 | estimated Glomerular Filtration Rate | TRANS | A | 0.70 | 0.0030 | 0.0004 | 1.96E-16 |
| rs12736457 | estimated Glomerular Filtration Rate | TRANS | C | 0.87 | 0.0054 | 0.0005 | 1.04E-25 |
| rs3118119 | estimated Glomerular Filtration Rate | TRANS | T | 0.18 | 0.0030 | 0.0005 | 6.67E-11 |
| rs267738 | estimated Glomerular Filtration Rate | TRANS | T | 0.80 | -0.0048 | 0.0004 | 1.20E-32 |
| rs4971100 | estimated Glomerular Filtration Rate | TRANS | A | 0.48 | 0.0020 | 0.0003 | 8.25E-10 |
| rs3845534 | estimated Glomerular Filtration Rate | TRANS | A | 0.53 | -0.0019 | 0.0003 | 1.20E-09 |
| rs4656220 | estimated Glomerular Filtration Rate | TRANS | T | 0.42 | 0.0020 | 0.0003 | 3.29E-10 |
| rs34720381 | estimated Glomerular Filtration Rate | TRANS | T | 0.09 | -0.0032 | 0.0005 | 2.52E-09 |

| rs1011731 | estimated Glomerular Filtration Rate | TRANS | A | 0.60 | -0.0019 | 0.0003 | 5.29E-09 |
| --- | --- | --- | --- | --- | --- | --- | --- |
| rs3795503 | estimated Glomerular Filtration Rate | TRANS | T | 0.36 | 0.0020 | 0.0003 | 9.77E-10 |
| rs78444298 | estimated Glomerular Filtration Rate | TRANS | A | 0.02 | -0.0105 | 0.0014 | 4.89E-14 |
| rs78329830 | estimated Glomerular Filtration Rate | TRANS | A | 0.96 | -0.0054 | 0.0009 | 6.69E-09 |
| rs3850625 | estimated Glomerular Filtration Rate | TRANS | A | 0.12 | 0.0046 | 0.0005 | 1.14E-18 |
| rs12024377 | estimated Glomerular Filtration Rate | TRANS | A | 0.43 | 0.0020 | 0.0003 | 3.14E-09 |
| rs2808454 | estimated Glomerular Filtration Rate | TRANS | A | 0.50 | 0.0019 | 0.0003 | 2.64E-09 |
| rs75625374 | estimated Glomerular Filtration Rate | TRANS | C | 0.06 | 0.0045 | 0.0007 | 4.48E-10 |
| rs7535253 | estimated Glomerular Filtration Rate | TRANS | T | 0.27 | 0.0021 | 0.0004 | 1.29E-09 |
| rs2577134 | estimated Glomerular Filtration Rate | TRANS | T | 0.68 | 0.0020 | 0.0003 | 1.52E-08 |
| rs61830291 | estimated Glomerular Filtration Rate | TRANS | A | 0.90 | -0.0036 | 0.0006 | 1.20E-09 |
| rs417237 | estimated Glomerular Filtration Rate | TRANS | T | 0.57 | 0.0018 | 0.0003 | 7.50E-09 |
| rs2490391 | estimated Glomerular Filtration Rate | TRANS | A | 0.43 | -0.0024 | 0.0003 | 1.33E-14 |
| rs3791221 | estimated Glomerular Filtration Rate | TRANS | A | 0.67 | 0.0022 | 0.0003 | 1.17E-11 |
| rs807624 | estimated Glomerular Filtration Rate | TRANS | T | 0.42 | 0.0032 | 0.0003 | 7.07E-23 |
| rs4491726 | estimated Glomerular Filtration Rate | TRANS | A | 0.69 | 0.0032 | 0.0004 | 5.48E-19 |
| rs780093 | estimated Glomerular Filtration Rate | TRANS | T | 0.42 | 0.0044 | 0.0003 | 1.63E-46 |
| rs2301343 | estimated Glomerular Filtration Rate | TRANS | T | 0.76 | -0.0023 | 0.0004 | 4.12E-10 |
| rs10865189 | estimated Glomerular Filtration Rate | TRANS | C | 0.51 | 0.0024 | 0.0003 | 3.29E-14 |
| rs6544743 | estimated Glomerular Filtration Rate | TRANS | T | 0.21 | -0.0021 | 0.0004 | 2.96E-08 |
| rs2971880 | estimated Glomerular Filtration Rate | TRANS | A | 0.37 | -0.0024 | 0.0003 | 7.63E-15 |
| rs10197255 | estimated Glomerular Filtration Rate | TRANS | A | 0.40 | 0.0018 | 0.0003 | 1.15E-08 |
| rs6546869 | estimated Glomerular Filtration Rate | TRANS | A | 0.23 | 0.0059 | 0.0004 | 5.06E-48 |
| rs72995641 | estimated Glomerular Filtration Rate | TRANS | A | 0.20 | -0.0021 | 0.0004 | 1.42E-08 |
| rs11123169 | estimated Glomerular Filtration Rate | TRANS | T | 0.68 | 0.0025 | 0.0003 | 9.24E-15 |
| rs17050272 | estimated Glomerular Filtration Rate | TRANS | A | 0.44 | -0.0022 | 0.0003 | 1.11E-12 |
| rs11694902 | estimated Glomerular Filtration Rate | TRANS | A | 0.14 | 0.0041 | 0.0005 | 1.06E-16 |
| rs6708702 | estimated Glomerular Filtration Rate | TRANS | A | 0.40 | -0.0019 | 0.0003 | 2.08E-08 |
| rs7425436 | estimated Glomerular Filtration Rate | TRANS | A | 0.65 | 0.0024 | 0.0003 | 5.16E-13 |
| rs4664475 | estimated Glomerular Filtration Rate | TRANS | T | 0.38 | -0.0020 | 0.0003 | 4.63E-11 |
| rs7565830 | estimated Glomerular Filtration Rate | TRANS | A | 0.63 | -0.0019 | 0.0003 | 7.87E-09 |
| rs35472707 | estimated Glomerular Filtration Rate | TRANS | T | 0.05 | -0.0073 | 0.0008 | 6.16E-19 |
| rs187355703 | estimated Glomerular Filtration Rate | TRANS | C | 0.97 | 0.0100 | 0.0011 | 1.01E-18 |
| rs35284526 | estimated Glomerular Filtration Rate | TRANS | A | 0.32 | 0.0029 | 0.0003 | 6.24E-17 |
| rs4666821 | estimated Glomerular Filtration Rate | TRANS | T | 0.53 | 0.0020 | 0.0003 | 2.50E-11 |
| rs60980181 | estimated Glomerular Filtration Rate | TRANS | A | 0.17 | -0.0027 | 0.0004 | 1.75E-10 |
| rs17818393 | estimated Glomerular Filtration Rate | TRANS | A | 0.48 | 0.0017 | 0.0003 | 1.25E-08 |
| rs1047891 | estimated Glomerular Filtration Rate | TRANS | A | 0.29 | -0.0065 | 0.0004 | 1.18E-75 |
| rs3791699 | estimated Glomerular Filtration Rate | TRANS | A | 0.31 | -0.0019 | 0.0003 | 2.57E-08 |
| rs1548945 | estimated Glomerular Filtration Rate | TRANS | T | 0.44 | 0.0036 | 0.0003 | 8.40E-31 |
| rs17462630 | estimated Glomerular Filtration Rate | TRANS | C | 0.31 | 0.0021 | 0.0004 | 4.02E-08 |
| rs1050816 | estimated Glomerular Filtration Rate | TRANS | T | 0.33 | 0.0026 | 0.0003 | 1.12E-15 |
| rs35669853 | estimated Glomerular Filtration Rate | TRANS | A | 0.18 | 0.0024 | 0.0004 | 9.19E-09 |
| rs7592697 | estimated Glomerular Filtration Rate | TRANS | T | 0.67 | -0.0019 | 0.0003 | 1.59E-08 |
| rs13003198 | estimated Glomerular Filtration Rate | TRANS | T | 0.37 | 0.0018 | 0.0003 | 3.14E-08 |
| rs795009 | estimated Glomerular Filtration Rate | TRANS | T | 0.73 | 0.0020 | 0.0003 | 7.01E-09 |
| rs6778731 | estimated Glomerular Filtration Rate | TRANS | T | 0.59 | -0.0017 | 0.0003 | 3.32E-08 |
| rs6779998 | estimated Glomerular Filtration Rate | TRANS | A | 0.52 | -0.0017 | 0.0003 | 1.65E-08 |
| rs11914389 | estimated Glomerular Filtration Rate | TRANS | T | 0.48 | 0.0030 | 0.0003 | 3.11E-22 |
| rs7651407 | estimated Glomerular Filtration Rate | TRANS | T | 0.44 | 0.0025 | 0.0004 | 2.41E-11 |
| rs4625 | estimated Glomerular Filtration Rate | TRANS | A | 0.70 | -0.0023 | 0.0004 | 5.47E-11 |
| rs77495508 | estimated Glomerular Filtration Rate | TRANS | A | 0.94 | 0.0044 | 0.0008 | 2.17E-08 |
| rs62257555 | estimated Glomerular Filtration Rate | TRANS | A | 0.94 | 0.0048 | 0.0009 | 2.18E-08 |
| rs2581820 | estimated Glomerular Filtration Rate | TRANS | A | 0.29 | 0.0021 | 0.0003 | 7.92E-10 |
| rs3774726 | estimated Glomerular Filtration Rate | TRANS | T | 0.36 | -0.0021 | 0.0003 | 3.65E-11 |
| rs2289746 | estimated Glomerular Filtration Rate | TRANS | T | 0.41 | -0.0019 | 0.0003 | 2.54E-09 |
| rs9868185 | estimated Glomerular Filtration Rate | TRANS | A | 0.50 | 0.0026 | 0.0003 | 5.03E-17 |
| rs10934754 | estimated Glomerular Filtration Rate | TRANS | T | 0.60 | 0.0020 | 0.0003 | 1.33E-10 |

| rs35320690 | estimated Glomerular Filtration Rate | TRANS | T | 0.73 | -0.0025 | 0.0004 | 3.03E-11 |
| --- | --- | --- | --- | --- | --- | --- | --- |
| rs9828976 | estimated Glomerular Filtration Rate | TRANS | C | 0.76 | -0.0024 | 0.0004 | 1.92E-09 |
| rs7624084 | estimated Glomerular Filtration Rate | TRANS | T | 0.58 | 0.0017 | 0.0003 | 1.97E-08 |
| rs1397764 | estimated Glomerular Filtration Rate | TRANS | A | 0.27 | 0.0043 | 0.0003 | 2.51E-37 |
| rs76272256 | estimated Glomerular Filtration Rate | TRANS | T | 0.24 | 0.0024 | 0.0004 | 4.88E-10 |
| rs1525362 | estimated Glomerular Filtration Rate | TRANS | T | 0.87 | -0.0028 | 0.0005 | 2.55E-09 |
| rs56065557 | estimated Glomerular Filtration Rate | TRANS | C | 0.32 | -0.0029 | 0.0003 | 4.32E-18 |
| rs11919484 | estimated Glomerular Filtration Rate | TRANS | T | 0.32 | -0.0026 | 0.0003 | 5.76E-16 |
| rs9823161 | estimated Glomerular Filtration Rate | TRANS | A | 0.58 | 0.0022 | 0.0004 | 3.25E-09 |
| rs75501914 | estimated Glomerular Filtration Rate | TRANS | A | 0.09 | 0.0039 | 0.0006 | 8.98E-11 |
| rs3775932 | estimated Glomerular Filtration Rate | TRANS | A | 0.51 | -0.0018 | 0.0003 | 1.96E-09 |
| rs16874073 | estimated Glomerular Filtration Rate | TRANS | T | 0.95 | -0.0045 | 0.0007 | 6.56E-11 |
| rs4864890 | estimated Glomerular Filtration Rate | TRANS | T | 0.31 | -0.0023 | 0.0004 | 2.50E-09 |
| rs28817415 | estimated Glomerular Filtration Rate | TRANS | T | 0.40 | -0.0073 | 0.0003 | 3.49E-120 |
| rs12509595 | estimated Glomerular Filtration Rate | TRANS | T | 0.70 | -0.0035 | 0.0003 | 6.42E-25 |
| rs223471 | estimated Glomerular Filtration Rate | TRANS | C | 0.34 | 0.0028 | 0.0003 | 5.86E-19 |
| rs55929207 | estimated Glomerular Filtration Rate | TRANS | C | 0.48 | 0.0019 | 0.0003 | 3.43E-10 |
| rs71606723 | estimated Glomerular Filtration Rate | TRANS | A | 0.77 | 0.0025 | 0.0004 | 3.38E-12 |
| rs13159523 | estimated Glomerular Filtration Rate | TRANS | A | 0.49 | -0.0024 | 0.0003 | 3.16E-13 |
| rs13157326 | estimated Glomerular Filtration Rate | TRANS | A | 0.45 | -0.0027 | 0.0003 | 1.67E-15 |
| rs1362800 | estimated Glomerular Filtration Rate | TRANS | T | 0.38 | -0.0049 | 0.0003 | 5.77E-51 |
| rs495237 | estimated Glomerular Filtration Rate | TRANS | T | 0.25 | 0.0027 | 0.0003 | 1.95E-14 |
| rs73754158 | estimated Glomerular Filtration Rate | TRANS | T | 0.15 | -0.0028 | 0.0005 | 4.40E-09 |
| rs11746506 | estimated Glomerular Filtration Rate | TRANS | T | 0.41 | 0.0017 | 0.0003 | 3.00E-08 |
| rs12520984 | estimated Glomerular Filtration Rate | TRANS | C | 0.32 | 0.0019 | 0.0003 | 5.26E-09 |
| rs79760705 | estimated Glomerular Filtration Rate | TRANS | T | 0.11 | 0.0056 | 0.0005 | 6.47E-25 |
| rs72759880 | estimated Glomerular Filtration Rate | TRANS | T | 0.11 | -0.0056 | 0.0005 | 1.11E-26 |
| rs2010352 | estimated Glomerular Filtration Rate | TRANS | A | 0.45 | -0.0018 | 0.0003 | 1.74E-09 |
| rs6453319 | estimated Glomerular Filtration Rate | TRANS | A | 0.40 | 0.0019 | 0.0003 | 1.77E-08 |
| rs3797537 | estimated Glomerular Filtration Rate | TRANS | A | 0.73 | 0.0019 | 0.0003 | 2.93E-08 |
| rs12777 | estimated Glomerular Filtration Rate | TRANS | C | 0.96 | 0.0050 | 0.0009 | 1.07E-08 |
| rs12163971 | estimated Glomerular Filtration Rate | TRANS | A | 0.16 | -0.0029 | 0.0004 | 1.74E-12 |
| rs11743174 | estimated Glomerular Filtration Rate | TRANS | T | 0.67 | 0.0019 | 0.0003 | 1.34E-08 |
| rs3812036 | estimated Glomerular Filtration Rate | TRANS | T | 0.26 | -0.0065 | 0.0004 | 2.38E-74 |
| rs11755724 | estimated Glomerular Filtration Rate | TRANS | A | 0.37 | 0.0027 | 0.0004 | 1.13E-13 |
| rs3765502 | estimated Glomerular Filtration Rate | TRANS | T | 0.79 | 0.0024 | 0.0004 | 3.98E-08 |
| rs622076 | estimated Glomerular Filtration Rate | TRANS | A | 0.14 | -0.0035 | 0.0005 | 2.61E-14 |
| rs144100226 | estimated Glomerular Filtration Rate | TRANS | T | 0.04 | 0.0059 | 0.0010 | 5.98E-09 |
| rs13200335 | estimated Glomerular Filtration Rate | TRANS | A | 0.46 | 0.0024 | 0.0003 | 8.06E-15 |
| rs77915916 | estimated Glomerular Filtration Rate | TRANS | A | 0.92 | 0.0046 | 0.0006 | 7.25E-14 |
| rs881858 | estimated Glomerular Filtration Rate | TRANS | A | 0.71 | -0.0054 | 0.0003 | 2.92E-55 |
| rs720989 | estimated Glomerular Filtration Rate | TRANS | T | 0.79 | 0.0021 | 0.0004 | 1.84E-08 |
| rs12212034 | estimated Glomerular Filtration Rate | TRANS | T | 0.37 | -0.0018 | 0.0003 | 1.03E-08 |
| rs6458868 | estimated Glomerular Filtration Rate | TRANS | T | 0.67 | -0.0020 | 0.0003 | 1.18E-09 |
| rs3925003 | estimated Glomerular Filtration Rate | TRANS | T | 0.58 | -0.0018 | 0.0003 | 1.97E-09 |
| rs72912510 | estimated Glomerular Filtration Rate | TRANS | A | 0.20 | -0.0024 | 0.0004 | 6.44E-09 |
| rs1857859 | estimated Glomerular Filtration Rate | TRANS | A | 0.31 | 0.0019 | 0.0003 | 2.58E-08 |
| rs7766720 | estimated Glomerular Filtration Rate | TRANS | T | 0.88 | 0.0029 | 0.0005 | 3.62E-08 |
| rs1268168 | estimated Glomerular Filtration Rate | TRANS | A | 0.34 | 0.0024 | 0.0003 | 5.36E-14 |
| rs7740107 | estimated Glomerular Filtration Rate | TRANS | A | 0.74 | 0.0027 | 0.0004 | 8.93E-13 |
| rs9375818 | estimated Glomerular Filtration Rate | TRANS | A | 0.25 | -0.0031 | 0.0004 | 6.09E-18 |
| rs3822939 | estimated Glomerular Filtration Rate | TRANS | A | 0.42 | -0.0025 | 0.0003 | 2.16E-16 |
| rs9397738 | estimated Glomerular Filtration Rate | TRANS | A | 0.84 | 0.0027 | 0.0004 | 3.50E-10 |
| rs12207180 | estimated Glomerular Filtration Rate | TRANS | A | 0.11 | -0.0085 | 0.0005 | 2.64E-63 |
| rs62435145 | estimated Glomerular Filtration Rate | TRANS | T | 0.59 | -0.0060 | 0.0004 | 2.25E-59 |
| rs6968554 | estimated Glomerular Filtration Rate | TRANS | A | 0.43 | -0.0019 | 0.0003 | 1.07E-09 |
| rs2058024 | estimated Glomerular Filtration Rate | TRANS | A | 0.65 | 0.0020 | 0.0003 | 4.65E-09 |
| rs3750081 | estimated Glomerular Filtration Rate | TRANS | T | 0.59 | -0.0022 | 0.0003 | 1.87E-12 |

| rs78503047 | estimated Glomerular Filtration Rate | TRANS | T | 0.09 | -0.0045 | 0.0007 | 7.52E-10 |
| --- | --- | --- | --- | --- | --- | --- | --- |
| rs1294861 | estimated Glomerular Filtration Rate | TRANS | T | 0.69 | 0.0020 | 0.0004 | 2.46E-08 |
| rs700753 | estimated Glomerular Filtration Rate | TRANS | C | 0.32 | 0.0031 | 0.0003 | 2.12E-20 |
| rs17152083 | estimated Glomerular Filtration Rate | TRANS | C | 0.11 | -0.0039 | 0.0005 | 1.62E-13 |
| rs55773927 | estimated Glomerular Filtration Rate | TRANS | T | 0.41 | 0.0019 | 0.0003 | 1.20E-08 |
| rs801193 | estimated Glomerular Filtration Rate | TRANS | T | 0.58 | -0.0020 | 0.0003 | 1.88E-09 |
| rs41301394 | estimated Glomerular Filtration Rate | TRANS | T | 0.32 | 0.0023 | 0.0003 | 3.65E-12 |
| rs6973656 | estimated Glomerular Filtration Rate | TRANS | A | 0.64 | 0.0035 | 0.0003 | 5.66E-28 |
| rs35154268 | estimated Glomerular Filtration Rate | TRANS | A | 0.72 | -0.0022 | 0.0004 | 9.85E-09 |
| rs3757387 | estimated Glomerular Filtration Rate | TRANS | T | 0.59 | 0.0030 | 0.0003 | 6.96E-20 |
| rs62491533 | estimated Glomerular Filtration Rate | TRANS | T | 0.81 | -0.0027 | 0.0004 | 1.08E-11 |
| rs10254101 | estimated Glomerular Filtration Rate | TRANS | T | 0.28 | -0.0068 | 0.0004 | 1.85E-67 |
| rs12671694 | estimated Glomerular Filtration Rate | TRANS | T | 0.54 | 0.0025 | 0.0003 | 2.16E-16 |
| rs868822 | estimated Glomerular Filtration Rate | TRANS | T | 0.33 | 0.0029 | 0.0003 | 2.20E-18 |
| rs2980423 | estimated Glomerular Filtration Rate | TRANS | T | 0.49 | -0.0023 | 0.0003 | 3.20E-12 |
| rs1533059 | estimated Glomerular Filtration Rate | TRANS | A | 0.51 | 0.0025 | 0.0003 | 1.19E-14 |
| rs35353426 | estimated Glomerular Filtration Rate | TRANS | T | 0.29 | -0.0026 | 0.0004 | 2.21E-11 |
| rs7832708 | estimated Glomerular Filtration Rate | TRANS | T | 0.49 | 0.0022 | 0.0004 | 9.79E-10 |
| rs11783418 | estimated Glomerular Filtration Rate | TRANS | A | 0.50 | -0.0020 | 0.0004 | 2.00E-08 |
| rs10098664 | estimated Glomerular Filtration Rate | TRANS | T | 0.49 | -0.0021 | 0.0003 | 6.13E-10 |
| rs4872526 | estimated Glomerular Filtration Rate | TRANS | T | 0.59 | 0.0019 | 0.0003 | 2.61E-09 |
| rs34861762 | estimated Glomerular Filtration Rate | TRANS | T | 0.39 | -0.0043 | 0.0003 | 4.06E-41 |
| rs10102889 | estimated Glomerular Filtration Rate | TRANS | C | 0.51 | -0.0036 | 0.0006 | 6.65E-09 |
| rs60991551 | estimated Glomerular Filtration Rate | TRANS | A | 0.19 | -0.0024 | 0.0004 | 5.77E-09 |
| rs1913641 | estimated Glomerular Filtration Rate | TRANS | T | 0.50 | -0.0018 | 0.0003 | 2.84E-09 |
| rs2976178 | estimated Glomerular Filtration Rate | TRANS | C | 0.67 | -0.0025 | 0.0003 | 7.81E-14 |
| rs79346194 | estimated Glomerular Filtration Rate | TRANS | A | 0.69 | -0.0022 | 0.0004 | 4.40E-10 |
| rs2954017 | estimated Glomerular Filtration Rate | TRANS | T | 0.46 | 0.0024 | 0.0003 | 1.66E-12 |
| rs12377027 | estimated Glomerular Filtration Rate | TRANS | A | 0.82 | -0.0026 | 0.0005 | 2.89E-08 |
| rs13287724 | estimated Glomerular Filtration Rate | TRANS | A | 0.89 | -0.0030 | 0.0006 | 4.67E-08 |
| rs544169 | estimated Glomerular Filtration Rate | TRANS | A | 0.73 | 0.0022 | 0.0003 | 3.89E-11 |
| rs2039424 | estimated Glomerular Filtration Rate | TRANS | A | 0.64 | 0.0044 | 0.0003 | 2.06E-44 |
| rs1321917 | estimated Glomerular Filtration Rate | TRANS | C | 0.44 | -0.0023 | 0.0003 | 1.38E-13 |
| rs13287061 | estimated Glomerular Filtration Rate | TRANS | T | 0.32 | -0.0021 | 0.0004 | 6.56E-09 |
| rs7024579 | estimated Glomerular Filtration Rate | TRANS | T | 0.29 | 0.0023 | 0.0004 | 8.17E-11 |
| rs28404308 | estimated Glomerular Filtration Rate | TRANS | A | 0.64 | 0.0024 | 0.0004 | 3.58E-09 |
| rs80282103 | estimated Glomerular Filtration Rate | TRANS | A | 0.91 | 0.0078 | 0.0006 | 1.23E-44 |
| rs6481598 | estimated Glomerular Filtration Rate | TRANS | C | 0.79 | 0.0024 | 0.0004 | 1.51E-09 |
| rs7072591 | estimated Glomerular Filtration Rate | TRANS | A | 0.56 | 0.0019 | 0.0003 | 2.67E-09 |
| rs8474 | estimated Glomerular Filtration Rate | TRANS | C | 0.45 | 0.0020 | 0.0003 | 1.77E-09 |
| rs10821905 | estimated Glomerular Filtration Rate | TRANS | A | 0.18 | 0.0037 | 0.0004 | 9.39E-19 |
| rs10821944 | estimated Glomerular Filtration Rate | TRANS | T | 0.70 | 0.0020 | 0.0003 | 3.89E-09 |
| rs7475348 | estimated Glomerular Filtration Rate | TRANS | T | 0.46 | 0.0031 | 0.0003 | 1.24E-22 |
| rs12240572 | estimated Glomerular Filtration Rate | TRANS | A | 0.17 | -0.0032 | 0.0006 | 5.25E-09 |
| rs816850 | estimated Glomerular Filtration Rate | TRANS | C | 0.25 | -0.0020 | 0.0004 | 7.40E-09 |
| rs7095954 | estimated Glomerular Filtration Rate | TRANS | A | 0.45 | -0.0018 | 0.0003 | 3.74E-08 |
| rs9420446 | estimated Glomerular Filtration Rate | TRANS | T | 0.27 | 0.0023 | 0.0004 | 4.97E-08 |
| rs2068888 | estimated Glomerular Filtration Rate | TRANS | A | 0.49 | -0.0024 | 0.0003 | 4.43E-15 |
| rs4918943 | estimated Glomerular Filtration Rate | TRANS | A | 0.22 | -0.0022 | 0.0004 | 9.19E-09 |
| rs284859 | estimated Glomerular Filtration Rate | TRANS | T | 0.21 | 0.0026 | 0.0004 | 4.99E-12 |
| rs1536225 | estimated Glomerular Filtration Rate | TRANS | T | 0.63 | -0.0021 | 0.0003 | 1.91E-11 |
| rs1055256 | estimated Glomerular Filtration Rate | TRANS | A | 0.42 | 0.0025 | 0.0003 | 3.56E-16 |
| rs11564722 | estimated Glomerular Filtration Rate | TRANS | T | 0.31 | 0.0033 | 0.0004 | 2.12E-20 |
| rs63934 | estimated Glomerular Filtration Rate | TRANS | A | 0.83 | 0.0041 | 0.0004 | 3.87E-23 |
| rs1541937 | estimated Glomerular Filtration Rate | TRANS | A | 0.64 | -0.0029 | 0.0004 | 5.18E-17 |
| rs75248620 | estimated Glomerular Filtration Rate | TRANS | A | 0.08 | 0.0038 | 0.0006 | 3.91E-09 |
| rs963837 | estimated Glomerular Filtration Rate | TRANS | T | 0.57 | -0.0057 | 0.0003 | 4.32E-73 |
| rs6484504 | estimated Glomerular Filtration Rate | TRANS | T | 0.31 | -0.0026 | 0.0003 | 6.45E-15 |

| rs61897431 | estimated Glomerular Filtration Rate | TRANS | T | 0.65 | 0.0029 | 0.0004 | 6.33E-16 |
| --- | --- | --- | --- | --- | --- | --- | --- |
| rs7127946 | estimated Glomerular Filtration Rate | TRANS | T | 0.69 | 0.0023 | 0.0003 | 2.39E-12 |
| rs2727040 | estimated Glomerular Filtration Rate | TRANS | T | 0.24 | -0.0026 | 0.0004 | 2.16E-09 |
| rs1813937 | estimated Glomerular Filtration Rate | TRANS | T | 0.72 | 0.0022 | 0.0004 | 6.89E-10 |
| rs11532186 | estimated Glomerular Filtration Rate | TRANS | T | 0.79 | 0.0029 | 0.0005 | 2.56E-10 |
| rs7118132 | estimated Glomerular Filtration Rate | TRANS | T | 0.23 | -0.0023 | 0.0004 | 5.18E-10 |
| rs1783827 | estimated Glomerular Filtration Rate | TRANS | A | 0.60 | -0.0020 | 0.0003 | 3.77E-09 |
| rs948493 | estimated Glomerular Filtration Rate | TRANS | T | 0.33 | -0.0033 | 0.0003 | 1.98E-24 |
| rs3892895 | estimated Glomerular Filtration Rate | TRANS | A | 0.40 | -0.0023 | 0.0003 | 6.17E-13 |
| rs11237450 | estimated Glomerular Filtration Rate | TRANS | A | 0.25 | 0.0032 | 0.0004 | 1.49E-14 |
| rs6589750 | estimated Glomerular Filtration Rate | TRANS | A | 0.63 | 0.0020 | 0.0003 | 1.54E-09 |
| rs10790452 | estimated Glomerular Filtration Rate | TRANS | T | 0.74 | 0.0020 | 0.0003 | 7.14E-09 |
| rs11062167 | estimated Glomerular Filtration Rate | TRANS | A | 0.48 | -0.0039 | 0.0003 | 2.48E-34 |
| rs632887 | estimated Glomerular Filtration Rate | TRANS | A | 0.62 | 0.0032 | 0.0003 | 2.19E-24 |
| rs4238020 | estimated Glomerular Filtration Rate | TRANS | T | 0.87 | 0.0029 | 0.0005 | 8.41E-09 |
| rs117113238 | estimated Glomerular Filtration Rate | TRANS | A | 0.09 | 0.0039 | 0.0006 | 8.60E-11 |
| rs10846157 | estimated Glomerular Filtration Rate | TRANS | A | 0.76 | -0.0034 | 0.0004 | 8.34E-21 |
| rs2634675 | estimated Glomerular Filtration Rate | TRANS | A | 0.52 | 0.0025 | 0.0003 | 2.66E-13 |
| rs12829524 | estimated Glomerular Filtration Rate | TRANS | T | 0.31 | -0.0022 | 0.0004 | 9.49E-10 |
| rs12313306 | estimated Glomerular Filtration Rate | TRANS | T | 0.23 | 0.0029 | 0.0004 | 1.46E-14 |
| rs1275609 | estimated Glomerular Filtration Rate | TRANS | A | 0.39 | 0.0024 | 0.0003 | 4.61E-13 |
| rs2339717 | estimated Glomerular Filtration Rate | TRANS | T | 0.63 | -0.0022 | 0.0003 | 2.62E-10 |
| rs10850001 | estimated Glomerular Filtration Rate | TRANS | A | 0.47 | -0.0021 | 0.0003 | 8.25E-11 |
| rs9590675 | estimated Glomerular Filtration Rate | TRANS | T | 0.43 | 0.0021 | 0.0003 | 1.17E-11 |
| rs41284816 | estimated Glomerular Filtration Rate | TRANS | T | 0.03 | -0.0078 | 0.0012 | 1.72E-10 |
| rs500830 | estimated Glomerular Filtration Rate | TRANS | T | 0.46 | 0.0029 | 0.0003 | 1.96E-19 |
| rs1956438 | estimated Glomerular Filtration Rate | TRANS | A | 0.62 | 0.0018 | 0.0003 | 2.28E-08 |
| rs72683923 | estimated Glomerular Filtration Rate | TRANS | T | 0.98 | -0.0074 | 0.0013 | 3.37E-08 |
| rs6574652 | estimated Glomerular Filtration Rate | TRANS | T | 0.50 | -0.0017 | 0.0003 | 2.41E-08 |
| rs1028455 | estimated Glomerular Filtration Rate | TRANS | A | 0.33 | 0.0020 | 0.0003 | 4.78E-10 |
| rs17184313 | estimated Glomerular Filtration Rate | TRANS | T | 0.17 | -0.0029 | 0.0005 | 1.98E-10 |
| rs61993680 | estimated Glomerular Filtration Rate | TRANS | A | 0.61 | -0.0019 | 0.0003 | 1.48E-08 |
| rs12913015 | estimated Glomerular Filtration Rate | TRANS | T | 0.41 | 0.0027 | 0.0003 | 2.28E-17 |
| rs6492982 | estimated Glomerular Filtration Rate | TRANS | T | 0.56 | -0.0033 | 0.0004 | 3.11E-20 |
| rs1145077 | estimated Glomerular Filtration Rate | TRANS | T | 0.42 | -0.0085 | 0.0003 | 6.85E-142 |
| rs690428 | estimated Glomerular Filtration Rate | TRANS | A | 0.67 | -0.0039 | 0.0003 | 8.23E-32 |
| rs1994887 | estimated Glomerular Filtration Rate | TRANS | A | 0.26 | -0.0020 | 0.0004 | 1.61E-08 |
| rs956006 | estimated Glomerular Filtration Rate | TRANS | T | 0.33 | 0.0019 | 0.0003 | 4.45E-09 |
| rs11071738 | estimated Glomerular Filtration Rate | TRANS | T | 0.57 | -0.0025 | 0.0003 | 2.01E-15 |
| rs28522606 | estimated Glomerular Filtration Rate | TRANS | T | 0.31 | 0.0022 | 0.0004 | 3.59E-08 |
| rs11071939 | estimated Glomerular Filtration Rate | TRANS | T | 0.93 | -0.0039 | 0.0006 | 4.92E-10 |
| rs351237 | estimated Glomerular Filtration Rate | TRANS | A | 0.62 | -0.0018 | 0.0003 | 4.42E-08 |
| rs2472297 | estimated Glomerular Filtration Rate | TRANS | T | 0.26 | 0.0039 | 0.0004 | 4.47E-20 |
| rs4886696 | estimated Glomerular Filtration Rate | TRANS | A | 0.33 | -0.0032 | 0.0004 | 2.03E-19 |
| rs4886755 | estimated Glomerular Filtration Rate | TRANS | A | 0.50 | 0.0041 | 0.0003 | 2.04E-39 |
| rs166906 | estimated Glomerular Filtration Rate | TRANS | T | 0.09 | 0.0033 | 0.0005 | 8.69E-10 |
| rs17507300 | estimated Glomerular Filtration Rate | TRANS | A | 0.83 | 0.0024 | 0.0004 | 1.05E-08 |
| rs7169629 | estimated Glomerular Filtration Rate | TRANS | C | 0.48 | 0.0018 | 0.0003 | 1.62E-08 |
| rs59646751 | estimated Glomerular Filtration Rate | TRANS | T | 0.30 | -0.0023 | 0.0003 | 3.06E-12 |
| rs438339 | estimated Glomerular Filtration Rate | TRANS | T | 0.88 | 0.0035 | 0.0006 | 4.97E-08 |
| rs1635404 | estimated Glomerular Filtration Rate | TRANS | T | 0.71 | -0.0025 | 0.0004 | 5.68E-11 |
| rs193538 | estimated Glomerular Filtration Rate | TRANS | T | 0.70 | -0.0020 | 0.0003 | 1.82E-09 |
| rs77924615 | estimated Glomerular Filtration Rate | TRANS | A | 0.20 | 0.0098 | 0.0004 | 1.45E-138 |
| rs7187776 | estimated Glomerular Filtration Rate | TRANS | A | 0.62 | -0.0022 | 0.0003 | 1.25E-12 |
| rs9932625 | estimated Glomerular Filtration Rate | TRANS | A | 0.26 | -0.0030 | 0.0003 | 2.24E-17 |
| rs7203398 | estimated Glomerular Filtration Rate | TRANS | A | 0.74 | 0.0025 | 0.0003 | 4.67E-13 |
| rs7185391 | estimated Glomerular Filtration Rate | TRANS | T | 0.27 | -0.0027 | 0.0004 | 8.56E-14 |
| rs62050038 | estimated Glomerular Filtration Rate | TRANS | A | 0.83 | 0.0028 | 0.0004 | 1.26E-11 |

| rs62053077 | estimated Glomerular Filtration Rate | TRANS | T | 0.43 | -0.0021 | 0.0004 | 3.72E-09 |
| --- | --- | --- | --- | --- | --- | --- | --- |
| rs1858800 | estimated Glomerular Filtration Rate | TRANS | T | 0.32 | 0.0020 | 0.0003 | 2.10E-09 |
| rs28581385 | estimated Glomerular Filtration Rate | TRANS | A | 0.84 | -0.0028 | 0.0004 | 1.35E-11 |
| rs72817412 | estimated Glomerular Filtration Rate | TRANS | T | 0.06 | 0.0046 | 0.0008 | 2.82E-08 |
| rs154656 | estimated Glomerular Filtration Rate | TRANS | A | 0.43 | -0.0030 | 0.0003 | 4.11E-18 |
| rs9900967 | estimated Glomerular Filtration Rate | TRANS | A | 0.55 | -0.0021 | 0.0003 | 1.43E-11 |
| rs28735420 | estimated Glomerular Filtration Rate | TRANS | T | 0.92 | 0.0039 | 0.0006 | 8.75E-10 |
| rs2349648 | estimated Glomerular Filtration Rate | TRANS | T | 0.34 | -0.0017 | 0.0003 | 4.93E-08 |
| rs9891340 | estimated Glomerular Filtration Rate | TRANS | T | 0.55 | 0.0024 | 0.0004 | 2.74E-11 |
| rs2440165 | estimated Glomerular Filtration Rate | TRANS | T | 0.64 | 0.0040 | 0.0003 | 1.72E-31 |
| rs2411192 | estimated Glomerular Filtration Rate | TRANS | A | 0.60 | -0.0024 | 0.0003 | 2.14E-15 |
| rs4794813 | estimated Glomerular Filtration Rate | TRANS | A | 0.24 | 0.0055 | 0.0004 | 3.58E-53 |
| rs143199581 | estimated Glomerular Filtration Rate | TRANS | A | 0.09 | 0.0040 | 0.0006 | 1.66E-10 |
| rs227731 | estimated Glomerular Filtration Rate | TRANS | T | 0.57 | 0.0018 | 0.0003 | 1.44E-08 |
| rs35662455 | estimated Glomerular Filtration Rate | TRANS | C | 0.89 | 0.0030 | 0.0005 | 3.86E-08 |
| rs9903801 | estimated Glomerular Filtration Rate | TRANS | C | 0.16 | 0.0047 | 0.0004 | 1.01E-27 |
| rs9895661 | estimated Glomerular Filtration Rate | TRANS | T | 0.72 | 0.0069 | 0.0004 | 2.49E-71 |
| rs8866 | estimated Glomerular Filtration Rate | TRANS | C | 0.62 | -0.0018 | 0.0003 | 2.17E-08 |
| rs883541 | estimated Glomerular Filtration Rate | TRANS | A | 0.72 | -0.0022 | 0.0003 | 2.66E-10 |
| rs1719934 | estimated Glomerular Filtration Rate | TRANS | A | 0.58 | 0.0026 | 0.0003 | 2.62E-17 |
| rs16942751 | estimated Glomerular Filtration Rate | TRANS | A | 0.18 | -0.0029 | 0.0005 | 2.21E-09 |
| rs9807656 | estimated Glomerular Filtration Rate | TRANS | T | 0.87 | -0.0028 | 0.0005 | 3.52E-09 |
| rs2878889 | estimated Glomerular Filtration Rate | TRANS | A | 0.53 | -0.0022 | 0.0003 | 2.42E-12 |
| rs4940525 | estimated Glomerular Filtration Rate | TRANS | T | 0.26 | 0.0025 | 0.0003 | 4.07E-13 |
| rs8096658 | estimated Glomerular Filtration Rate | TRANS | C | 0.55 | 0.0050 | 0.0004 | 3.15E-44 |
| rs2974751 | estimated Glomerular Filtration Rate | TRANS | A | 0.38 | 0.0018 | 0.0003 | 4.39E-08 |
| rs4808154 | estimated Glomerular Filtration Rate | TRANS | T | 0.72 | 0.0024 | 0.0004 | 4.41E-10 |
| rs8101667 | estimated Glomerular Filtration Rate | TRANS | T | 0.39 | 0.0044 | 0.0003 | 9.35E-44 |
| rs7251730 | estimated Glomerular Filtration Rate | TRANS | T | 0.35 | 0.0024 | 0.0003 | 2.20E-12 |
| rs78241494 | estimated Glomerular Filtration Rate | TRANS | T | 0.70 | -0.0030 | 0.0004 | 4.39E-17 |
| rs113445505 | estimated Glomerular Filtration Rate | TRANS | T | 0.36 | 0.0037 | 0.0003 | 6.96E-27 |
| rs281380 | estimated Glomerular Filtration Rate | TRANS | T | 0.56 | -0.0021 | 0.0003 | 2.86E-10 |
| rs34647824 | estimated Glomerular Filtration Rate | TRANS | A | 0.74 | -0.0021 | 0.0004 | 3.95E-08 |
| rs62187537 | estimated Glomerular Filtration Rate | TRANS | T | 0.07 | 0.0039 | 0.0007 | 9.21E-09 |
| rs1509117 | estimated Glomerular Filtration Rate | TRANS | A | 0.30 | 0.0024 | 0.0004 | 7.07E-10 |
| rs1041606 | estimated Glomerular Filtration Rate | TRANS | T | 0.23 | -0.0021 | 0.0004 | 2.52E-08 |
| rs6087579 | estimated Glomerular Filtration Rate | TRANS | A | 0.48 | -0.0028 | 0.0003 | 1.39E-19 |
| rs2273684 | estimated Glomerular Filtration Rate | TRANS | T | 0.58 | 0.0032 | 0.0003 | 7.79E-25 |
| rs6029640 | estimated Glomerular Filtration Rate | TRANS | A | 0.57 | -0.0021 | 0.0003 | 1.97E-09 |
| rs17216707 | estimated Glomerular Filtration Rate | TRANS | T | 0.81 | -0.0051 | 0.0004 | 1.07E-33 |
| rs2235826 | estimated Glomerular Filtration Rate | TRANS | A | 0.79 | -0.0030 | 0.0004 | 6.83E-15 |
| rs1407040 | estimated Glomerular Filtration Rate | TRANS | T | 0.68 | 0.0018 | 0.0003 | 1.44E-08 |
| rs35636653 | estimated Glomerular Filtration Rate | TRANS | T | 0.34 | 0.0022 | 0.0003 | 2.28E-11 |
| rs72629024 | estimated Glomerular Filtration Rate | TRANS | C | 0.66 | 0.0035 | 0.0005 | 2.02E-13 |
| rs4408777 | estimated Glomerular Filtration Rate | TRANS | A | 0.51 | -0.0021 | 0.0003 | 5.40E-11 |
| rs2823139 | estimated Glomerular Filtration Rate | TRANS | A | 0.33 | -0.0026 | 0.0003 | 5.24E-16 |
| rs2834317 | estimated Glomerular Filtration Rate | TRANS | A | 0.14 | -0.0035 | 0.0005 | 4.26E-14 |
| rs2244237 | estimated Glomerular Filtration Rate | TRANS | T | 0.22 | 0.0027 | 0.0004 | 6.28E-11 |
| rs131263 | estimated Glomerular Filtration Rate | TRANS | T | 0.46 | 0.0024 | 0.0004 | 2.20E-11 |
| rs80576 | estimated Glomerular Filtration Rate | TRANS | A | 0.16 | -0.0028 | 0.0005 | 1.27E-09 |
| rs4820324 | estimated Glomerular Filtration Rate | TRANS | C | 0.59 | -0.0023 | 0.0003 | 5.07E-14 |
| rs112880707 | estimated Glomerular Filtration Rate | TRANS | T | 0.17 | 0.0052 | 0.0005 | 4.88E-31 |
| rs738527 | estimated Glomerular Filtration Rate | TRANS | T | 0.29 | 0.0032 | 0.0003 | 4.18E-21 |
| rs11603334 | Fasting Proinsulin | EUP | A | 0.15 | 0.0938 | 0.0052 | 3.20E-102 |
| rs10501320 | Fasting Proinsulin | EUP | G | 0.72 | 0.0775 | 0.0064 | 1.10E-88 |
| rs6235 | Fasting Proinsulin | EUP | G | 0.28 | 0.0438 | 0.0049 | 9.80E-27 |
| rs7903146 | Fasting Proinsulin | EUP | T | 0.30 | 0.0303 | 0.0063 | 2.30E-20 |
| rs4502156 | Fasting Proinsulin | EUP | T | 0.58 | 0.0260 | 0.0043 | 3.50E-20 |

| rs11558471 | Fasting Proinsulin | EUP | A | 0.69 | 0.0273 | 0.0048 | 3.10E-18 |
| --- | --- | --- | --- | --- | --- | --- | --- |
| rs10838687 | Fasting Proinsulin | EUP | T | 0.80 | 0.0246 | 0.0049 | 6.90E-12 |
| rs1549318 | Fasting Proinsulin | EUP | T | 0.61 | 0.0180 | 0.0050 | 2.40E-10 |
| rs4790333 | Fasting Proinsulin | EUP | T | 0.45 | 0.0179 | 0.0042 | 3.00E-09 |
| rs1011939 | Birth Weight | TRANS | G | 0.31 | 0.0240 | 0.0040 | 2.70E-09 |
| rs10402712 | Birth Weight | TRANS | A | 0.27 | 0.0230 | 0.0040 | 2.30E-08 |
| rs10830963 | Birth Weight | TRANS | G | 0.27 | 0.0220 | 0.0040 | 1.00E-07 |
| rs10935733 | Birth Weight | TRANS | T | 0.42 | 0.0230 | 0.0040 | 6.20E-10 |
| rs1101081 | Birth Weight | TRANS | C | 0.73 | 0.0370 | 0.0040 | 6.10E-20 |
| rs11055034 | Birth Weight | TRANS | C | 0.73 | 0.0230 | 0.0040 | 2.30E-08 |
| rs113086489 | Birth Weight | TRANS | T | 0.55 | 0.0300 | 0.0040 | 1.30E-15 |
| rs11719201 | Birth Weight | TRANS | T | 0.23 | 0.0460 | 0.0040 | 6.40E-27 |
| rs11765649 | Birth Weight | TRANS | T | 0.76 | 0.0260 | 0.0040 | 1.00E-09 |
| rs12543725 | Birth Weight | TRANS | G | 0.60 | 0.0220 | 0.0040 | 1.90E-09 |
| rs12823128 | Birth Weight | TRANS | T | 0.56 | 0.0200 | 0.0040 | 3.20E-08 |
| rs12906125 | Birth Weight | TRANS | G | 0.69 | 0.0230 | 0.0040 | 1.00E-08 |
| rs12942207 | Birth Weight | TRANS | C | 0.30 | 0.0240 | 0.0040 | 3.00E-09 |
| rs13266210 | Birth Weight | TRANS | A | 0.79 | 0.0300 | 0.0040 | 1.60E-11 |
| rs13322435 | Birth Weight | TRANS | A | 0.59 | 0.0520 | 0.0040 | 1.30E-42 |
| rs134594 | Birth Weight | TRANS | C | 0.35 | 0.0220 | 0.0040 | 2.20E-08 |
| rs1351394 | Birth Weight | TRANS | T | 0.48 | 0.0430 | 0.0040 | 2.00E-33 |
| rs1374204 | Birth Weight | TRANS | T | 0.70 | 0.0460 | 0.0040 | 1.50E-29 |
| rs1415701 | Birth Weight | TRANS | G | 0.73 | 0.0270 | 0.0040 | 4.00E-11 |
| rs144843919 | Birth Weight | TRANS | G | 0.96 | 0.0680 | 0.0110 | 1.50E-09 |
| rs1819436 | Birth Weight | TRANS | C | 0.87 | 0.0330 | 0.0050 | 1.80E-09 |
| rs2150052 | Birth Weight | TRANS | T | 0.50 | 0.0200 | 0.0040 | 2.80E-08 |
| rs2229742 | Birth Weight | TRANS | G | 0.87 | 0.0340 | 0.0060 | 1.50E-08 |
| rs2242116 | Birth Weight | TRANS | A | 0.39 | 0.0210 | 0.0040 | 1.20E-08 |
| rs2324499 | Birth Weight | TRANS | G | 0.67 | 0.0230 | 0.0040 | 8.30E-09 |
| rs2421016 | Birth Weight | TRANS | T | 0.48 | 0.0210 | 0.0040 | 6.10E-09 |
| rs2473248 | Birth Weight | TRANS | C | 0.87 | 0.0330 | 0.0050 | 1.10E-09 |
| rs28510415 | Birth Weight | TRANS | G | 0.09 | 0.0530 | 0.0060 | 4.00E-16 |
| rs28530618 | Birth Weight | TRANS | A | 0.50 | 0.0240 | 0.0040 | 8.40E-11 |
| rs2854355 | Birth Weight | TRANS | G | 0.26 | 0.0240 | 0.0040 | 2.20E-08 |
| rs35261542 | Birth Weight | TRANS | C | 0.73 | 0.0440 | 0.0040 | 9.70E-29 |
| rs3753639 | Birth Weight | TRANS | C | 0.23 | 0.0310 | 0.0040 | 1.30E-12 |
| rs6016377 | Birth Weight | TRANS | T | 0.45 | 0.0240 | 0.0040 | 3.70E-10 |
| rs6040076 | Birth Weight | TRANS | C | 0.51 | 0.0220 | 0.0040 | 7.20E-09 |
| rs61154119 | Birth Weight | TRANS | T | 0.84 | 0.0280 | 0.0050 | 2.30E-08 |
| rs61830764 | Birth Weight | TRANS | A | 0.36 | 0.0220 | 0.0040 | 4.50E-08 |
| rs61862780 | Birth Weight | TRANS | T | 0.52 | 0.0280 | 0.0040 | 9.50E-15 |
| rs62240962 | Birth Weight | TRANS | C | 0.92 | 0.0470 | 0.0070 | 3.70E-02 |
| rs62466330 | Birth Weight | TRANS | C | 0.07 | 0.0510 | 0.0070 | 5.90E-12 |
| rs6537307 | Birth Weight | TRANS | G | 0.48 | 0.0260 | 0.0040 | 1.30E-12 |
| rs6959887 | Birth Weight | TRANS | A | 0.61 | 0.0210 | 0.0040 | 1.00E-08 |
| rs6989280 | Birth Weight | TRANS | G | 0.70 | 0.0220 | 0.0040 | 5.00E-08 |
| rs700059 | Birth Weight | TRANS | G | 0.16 | 0.0360 | 0.0050 | 1.20E-12 |
| rs7076938 | Birth Weight | TRANS | T | 0.73 | 0.0350 | 0.0040 | 4.70E-18 |
| rs72480273 | Birth Weight | TRANS | C | 0.17 | 0.0300 | 0.0050 | 1.50E-09 |
| rs72851023 | Birth Weight | TRANS | T | 0.07 | 0.0460 | 0.0070 | 6.80E-10 |
| rs7402982 | Birth Weight | TRANS | A | 0.42 | 0.0230 | 0.0040 | 1.10E-09 |
| rs74233809 | Birth Weight | TRANS | C | 0.08 | 0.0390 | 0.0060 | 1.80E-09 |
| rs7575873 | Birth Weight | TRANS | A | 0.88 | 0.0360 | 0.0060 | 6.20E-11 |
| rs7729301 | Birth Weight | TRANS | A | 0.72 | 0.0250 | 0.0040 | 1.30E-09 |
| rs7742369 | Birth Weight | TRANS | G | 0.19 | 0.0270 | 0.0050 | 1.10E-08 |
| rs7847628 | Birth Weight | TRANS | G | 0.67 | 0.0230 | 0.0040 | 5.40E-09 |
| rs7964361 | Birth Weight | TRANS | A | 0.08 | 0.0380 | 0.0070 | 9.70E-09 |
| rs798489 | Birth Weight | TRANS | C | 0.74 | 0.0240 | 0.0040 | 5.00E-09 |

| rs854037 | Birth Weight | TRANS | A | 0.80 | 0.0250 | 0.0050 | 3.50E-08 |
| --- | --- | --- | --- | --- | --- | --- | --- |
| rs925098 | Birth Weight | TRANS | G | 0.28 | 0.0320 | 0.0040 | 1.30E-15 |
| rs9379832 | Birth Weight | TRANS | A | 0.71 | 0.0240 | 0.0040 | 1.20E-08 |
| rs11676272 | Childhood BMI | EUP | G | 0.46 | 0.0680 | 0.0070 | 8.55E-23 |
| rs12041852 | Childhood BMI | EUP | G | 0.46 | 0.0460 | 0.0070 | 1.77E-10 |
| rs12429545 | Childhood BMI | EUP | A | 0.13 | 0.0760 | 0.0100 | 3.66E-11 |
| rs13107325 | Childhood BMI | EUP | C | 0.07 | 0.0810 | 0.0160 | 1.19E-08 |
| rs13130484 | Childhood BMI | EUP | T | 0.44 | 0.0670 | 0.0070 | 8.94E-11 |
| rs1421085 | Childhood BMI | EUP | C | 0.41 | 0.0590 | 0.0070 | 3.20E-19 |
| rs17309930 | Childhood BMI | EUP | A | 0.21 | 0.0450 | 0.0090 | 2.47E-08 |
| rs2590942 | Childhood BMI | EUP | T | 0.82 | 0.0470 | 0.0090 | 3.88E-09 |
| rs4854349 | Childhood BMI | EUP | C | 0.83 | 0.0900 | 0.0090 | 6.00E-21 |
| rs543874 | Childhood BMI | EUP | G | 0.20 | 0.0770 | 0.0090 | 2.38E-17 |
| rs6567160 | Childhood BMI | EUP | C | 0.23 | 0.0500 | 0.0080 | 4.06E-12 |
| rs7132908 | Childhood BMI | EUP | A | 0.39 | 0.0660 | 0.0080 | 4.99E-19 |
| rs7550711 | Childhood BMI | EUP | T | 0.04 | 0.1050 | 0.0190 | 1.50E-08 |
| rs8046312 | Childhood BMI | EUP | A | 0.81 | 0.0420 | 0.0090 | 4.06E-10 |
| rs987237 | Childhood BMI | EUP | G | 0.19 | 0.0620 | 0.0090 | 3.81E-13 |
| rs1000940 | Body Mass Index (BMI) | TRANS | G | 0.32 | 0.0192 | 0.0034 | 1.28E-08 |
| rs10132280 | Body Mass Index (BMI) | TRANS | C | 0.68 | 0.0230 | 0.0034 | 1.14E-11 |
| rs1016287 | Body Mass Index (BMI) | TRANS | T | 0.29 | 0.0229 | 0.0034 | 2.25E-11 |
| rs10182181 | Body Mass Index (BMI) | TRANS | G | 0.46 | 0.0307 | 0.0031 | 8.78E-24 |
| rs10733682 | Body Mass Index (BMI) | TRANS | A | 0.48 | 0.0174 | 0.0031 | 1.83E-08 |
| rs10938397 | Body Mass Index (BMI) | TRANS | G | 0.43 | 0.0402 | 0.0031 | 3.21E-38 |
| rs10968576 | Body Mass Index (BMI) | TRANS | G | 0.32 | 0.0249 | 0.0033 | 6.61E-14 |
| rs11030104 | Body Mass Index (BMI) | TRANS | A | 0.79 | 0.0414 | 0.0038 | 5.56E-28 |
| rs11057405 | Body Mass Index (BMI) | TRANS | G | 0.90 | 0.0307 | 0.0055 | 2.02E-08 |
| rs11126666 | Body Mass Index (BMI) | TRANS | A | 0.28 | 0.0207 | 0.0034 | 1.33E-09 |
| rs11165643 | Body Mass Index (BMI) | TRANS | T | 0.58 | 0.0218 | 0.0031 | 2.07E-12 |
| rs11191560 | Body Mass Index (BMI) | TRANS | C | 0.09 | 0.0308 | 0.0053 | 8.45E-09 |
| rs11583200 | Body Mass Index (BMI) | TRANS | C | 0.40 | 0.0177 | 0.0031 | 1.48E-08 |
| rs1167827 | Body Mass Index (BMI) | TRANS | G | 0.55 | 0.0202 | 0.0033 | 6.33E-10 |
| rs11688816 | Body Mass Index (BMI) | TRANS | G | 0.52 | 0.0172 | 0.0031 | 1.89E-08 |
| rs11727676 | Body Mass Index (BMI) | TRANS | T | 0.91 | 0.0358 | 0.0064 | 2.55E-08 |
| rs11847697 | Body Mass Index (BMI) | TRANS | T | 0.04 | 0.0492 | 0.0084 | 3.99E-09 |
| rs12286929 | Body Mass Index (BMI) | TRANS | G | 0.52 | 0.0217 | 0.0031 | 1.31E-12 |
| rs12401738 | Body Mass Index (BMI) | TRANS | A | 0.35 | 0.0211 | 0.0033 | 1.15E-10 |
| rs12429545 | Body Mass Index (BMI) | TRANS | A | 0.13 | 0.0334 | 0.0047 | 1.09E-12 |
| rs12446632 | Body Mass Index (BMI) | TRANS | G | 0.87 | 0.0403 | 0.0046 | 1.48E-18 |
| rs12566985 | Body Mass Index (BMI) | TRANS | G | 0.45 | 0.0242 | 0.0031 | 3.28E-15 |
| rs12885454 | Body Mass Index (BMI) | TRANS | C | 0.64 | 0.0207 | 0.0033 | 1.94E-10 |
| rs12940622 | Body Mass Index (BMI) | TRANS | G | 0.57 | 0.0182 | 0.0031 | 2.49E-09 |
| rs13021737 | Body Mass Index (BMI) | TRANS | G | 0.83 | 0.0601 | 0.0040 | 1.11E-50 |
| rs13078960 | Body Mass Index (BMI) | TRANS | G | 0.20 | 0.0297 | 0.0039 | 1.74E-14 |
| rs13107325 | Body Mass Index (BMI) | TRANS | T | 0.07 | 0.0477 | 0.0068 | 1.83E-12 |
| rs13191362 | Body Mass Index (BMI) | TRANS | A | 0.88 | 0.0277 | 0.0048 | 7.34E-09 |
| rs13201877 | Body Mass Index (BMI) | TRANS | G | 0.14 | 0.0233 | 0.0045 | 2.35E-07 |
| rs1441264 | Body Mass Index (BMI) | TRANS | A | 0.61 | 0.0175 | 0.0032 | 6.04E-08 |
| rs1460676 | Body Mass Index (BMI) | TRANS | C | 0.17 | 0.0197 | 0.0040 | 8.98E-07 |
| rs1516725 | Body Mass Index (BMI) | TRANS | C | 0.87 | 0.0451 | 0.0046 | 1.89E-22 |
| rs1528435 | Body Mass Index (BMI) | TRANS | T | 0.63 | 0.0178 | 0.0031 | 1.20E-08 |
| rs1558902 | Body Mass Index (BMI) | TRANS | A | 0.42 | 0.0818 | 0.0031 | 7.51E-153 |
| rs16851483 | Body Mass Index (BMI) | TRANS | T | 0.07 | 0.0483 | 0.0077 | 3.55E-10 |
| rs16907751 | Body Mass Index (BMI) | TRANS | C | 0.92 | 0.0350 | 0.0066 | 1.26E-07 |
| rs16951275 | Body Mass Index (BMI) | TRANS | T | 0.78 | 0.0311 | 0.0037 | 1.91E-17 |
| rs17001654 | Body Mass Index (BMI) | TRANS | G | 0.15 | 0.0306 | 0.0053 | 7.76E-09 |
| rs17024393 | Body Mass Index (BMI) | TRANS | C | 0.04 | 0.0658 | 0.0088 | 7.03E-14 |
| rs17094222 | Body Mass Index (BMI) | TRANS | C | 0.21 | 0.0249 | 0.0038 | 5.94E-11 |

| rs17203016 | Body Mass Index (BMI) | TRANS | G | 0.20 | 0.0210 | 0.0039 | 8.15E-08 |
| --- | --- | --- | --- | --- | --- | --- | --- |
| rs17405819 | Body Mass Index (BMI) | TRANS | T | 0.70 | 0.0224 | 0.0033 | 2.07E-11 |
| rs17724992 | Body Mass Index (BMI) | TRANS | A | 0.75 | 0.0194 | 0.0035 | 3.42E-08 |
| rs1808579 | Body Mass Index (BMI) | TRANS | C | 0.53 | 0.0167 | 0.0031 | 4.17E-08 |
| rs1928295 | Body Mass Index (BMI) | TRANS | T | 0.55 | 0.0188 | 0.0031 | 7.91E-10 |
| rs2033529 | Body Mass Index (BMI) | TRANS | G | 0.29 | 0.0190 | 0.0033 | 1.39E-08 |
| rs2033732 | Body Mass Index (BMI) | TRANS | C | 0.75 | 0.0192 | 0.0035 | 4.89E-08 |
| rs205262 | Body Mass Index (BMI) | TRANS | G | 0.27 | 0.0221 | 0.0035 | 1.75E-10 |
| rs2075650 | Body Mass Index (BMI) | TRANS | A | 0.85 | 0.0258 | 0.0045 | 1.25E-08 |
| rs2080454 | Body Mass Index (BMI) | TRANS | C | 0.41 | 0.0168 | 0.0031 | 6.55E-08 |
| rs2112347 | Body Mass Index (BMI) | TRANS | T | 0.63 | 0.0261 | 0.0031 | 6.19E-17 |
| rs2121279 | Body Mass Index (BMI) | TRANS | T | 0.15 | 0.0245 | 0.0044 | 2.31E-08 |
| rs2176040 | Body Mass Index (BMI) | TRANS | A | 0.37 | 0.0141 | 0.0031 | 6.06E-06 |
| rs2176598 | Body Mass Index (BMI) | TRANS | T | 0.25 | 0.0198 | 0.0036 | 2.97E-08 |
| rs2207139 | Body Mass Index (BMI) | TRANS | G | 0.18 | 0.0447 | 0.0040 | 4.13E-29 |
| rs2245368 | Body Mass Index (BMI) | TRANS | C | 0.18 | 0.0317 | 0.0057 | 3.19E-08 |
| rs2287019 | Body Mass Index (BMI) | TRANS | C | 0.80 | 0.0360 | 0.0042 | 4.59E-18 |
| rs2365389 | Body Mass Index (BMI) | TRANS | C | 0.58 | 0.0200 | 0.0031 | 1.63E-10 |
| rs2650492 | Body Mass Index (BMI) | TRANS | A | 0.30 | 0.0207 | 0.0035 | 1.92E-09 |
| rs2820292 | Body Mass Index (BMI) | TRANS | C | 0.56 | 0.0195 | 0.0031 | 1.83E-10 |
| rs2836754 | Body Mass Index (BMI) | TRANS | C | 0.61 | 0.0164 | 0.0032 | 4.16E-07 |
| rs29941 | Body Mass Index (BMI) | TRANS | G | 0.67 | 0.0182 | 0.0033 | 2.41E-08 |
| rs3101336 | Body Mass Index (BMI) | TRANS | C | 0.61 | 0.0334 | 0.0031 | 2.66E-26 |
| rs3736485 | Body Mass Index (BMI) | TRANS | A | 0.45 | 0.0176 | 0.0031 | 7.41E-09 |
| rs3810291 | Body Mass Index (BMI) | TRANS | A | 0.67 | 0.0283 | 0.0036 | 4.81E-15 |
| rs3817334 | Body Mass Index (BMI) | TRANS | T | 0.41 | 0.0262 | 0.0031 | 5.15E-17 |
| rs3849570 | Body Mass Index (BMI) | TRANS | A | 0.36 | 0.0188 | 0.0034 | 2.60E-08 |
| rs3888190 | Body Mass Index (BMI) | TRANS | A | 0.40 | 0.0309 | 0.0031 | 3.14E-23 |
| rs4256980 | Body Mass Index (BMI) | TRANS | G | 0.65 | 0.0209 | 0.0031 | 2.90E-11 |
| rs4740619 | Body Mass Index (BMI) | TRANS | T | 0.54 | 0.0179 | 0.0031 | 4.56E-09 |
| rs4787491 | Body Mass Index (BMI) | TRANS | G | 0.51 | 0.0159 | 0.0034 | 2.24E-06 |
| rs492400 | Body Mass Index (BMI) | TRANS | C | 0.42 | 0.0158 | 0.0031 | 4.17E-07 |
| rs543874 | Body Mass Index (BMI) | TRANS | G | 0.19 | 0.0482 | 0.0039 | 2.62E-35 |
| rs6091540 | Body Mass Index (BMI) | TRANS | C | 0.72 | 0.0188 | 0.0035 | 8.02E-08 |
| rs6465468 | Body Mass Index (BMI) | TRANS | T | 0.30 | 0.0166 | 0.0035 | 2.32E-06 |
| rs6477694 | Body Mass Index (BMI) | TRANS | C | 0.37 | 0.0174 | 0.0031 | 2.67E-08 |
| rs6567160 | Body Mass Index (BMI) | TRANS | C | 0.24 | 0.0556 | 0.0036 | 3.93E-53 |
| rs657452 | Body Mass Index (BMI) | TRANS | A | 0.39 | 0.0227 | 0.0031 | 5.48E-13 |
| rs6804842 | Body Mass Index (BMI) | TRANS | G | 0.57 | 0.0185 | 0.0031 | 2.48E-09 |
| rs7138803 | Body Mass Index (BMI) | TRANS | A | 0.38 | 0.0315 | 0.0031 | 8.15E-24 |
| rs7141420 | Body Mass Index (BMI) | TRANS | T | 0.53 | 0.0235 | 0.0031 | 1.23E-14 |
| rs7164727 | Body Mass Index (BMI) | TRANS | T | 0.69 | 0.0180 | 0.0033 | 6.83E-08 |
| rs7239883 | Body Mass Index (BMI) | TRANS | G | 0.39 | 0.0164 | 0.0031 | 1.63E-07 |
| rs7243357 | Body Mass Index (BMI) | TRANS | T | 0.81 | 0.0217 | 0.0040 | 3.86E-08 |
| rs758747 | Body Mass Index (BMI) | TRANS | T | 0.27 | 0.0225 | 0.0037 | 7.47E-10 |
| rs7599312 | Body Mass Index (BMI) | TRANS | G | 0.72 | 0.0220 | 0.0034 | 1.17E-10 |
| rs7715256 | Body Mass Index (BMI) | TRANS | G | 0.42 | 0.0163 | 0.0031 | 1.70E-07 |
| rs7899106 | Body Mass Index (BMI) | TRANS | G | 0.05 | 0.0395 | 0.0071 | 2.96E-08 |
| rs7903146 | Body Mass Index (BMI) | TRANS | C | 0.71 | 0.0234 | 0.0034 | 1.11E-11 |
| rs9374842 | Body Mass Index (BMI) | TRANS | T | 0.75 | 0.0187 | 0.0035 | 9.67E-08 |
| rs9400239 | Body Mass Index (BMI) | TRANS | C | 0.69 | 0.0188 | 0.0033 | 1.61E-08 |
| rs9540493 | Body Mass Index (BMI) | TRANS | A | 0.46 | 0.0172 | 0.0033 | 1.42E-07 |
| rs9641123 | Body Mass Index (BMI) | TRANS | C | 0.43 | 0.0191 | 0.0038 | 5.00E-07 |
| rs977747 | Body Mass Index (BMI) | TRANS | T | 0.39 | 0.0167 | 0.0031 | 8.65E-08 |
| rs9914578 | Body Mass Index (BMI) | TRANS | G | 0.21 | 0.0201 | 0.0038 | 8.99E-08 |
| rs9925964 | Body Mass Index (BMI) | TRANS | A | 0.62 | 0.0192 | 0.0031 | 8.11E-10 |
| rs10195252 | WHR (BMI adjusted) | EUP | T | 0.59 | 0.0270 | 0.0036 | 5.90E-15 |
| rs10245353 | WHR (BMI adjusted) | EUP | A | 0.20 | 0.0350 | 0.0042 | 8.40E-16 |

| rs1045241 | WHR (BMI adjusted) | EUP | C | 0.71 | 0.0190 | 0.0042 | 4.40E-07 |
| --- | --- | --- | --- | --- | --- | --- | --- |
| rs10804591 | WHR (BMI adjusted) | EUP | A | 0.79 | 0.0250 | 0.0040 | 6.60E-09 |
| rs10842707 | WHR (BMI adjusted) | EUP | T | 0.23 | 0.0320 | 0.0034 | 4.40E-16 |
| rs10919388 | WHR (BMI adjusted) | EUP | C | 0.72 | 0.0240 | 0.0035 | 3.20E-09 |
| rs10991437 | WHR (BMI adjusted) | EUP | A | 0.11 | 0.0310 | 0.0036 | 1.00E-08 |
| rs11231693 | WHR (BMI adjusted) | EUP | A | 0.06 | 0.0410 | 0.0036 | 4.50E-08 |
| rs12454712 | WHR (BMI adjusted) | EUP | T | 0.61 | 0.0160 | 0.0039 | 1.00E-04 |
| rs12608504 | WHR (BMI adjusted) | EUP | A | 0.36 | 0.0220 | 0.0036 | 8.80E-10 |
| rs12679556 | WHR (BMI adjusted) | EUP | G | 0.25 | 0.0270 | 0.0040 | 2.10E-11 |
| rs1294410 | WHR (BMI adjusted) | EUP | C | 0.63 | 0.0310 | 0.0035 | 2.00E-18 |
| rs1358980 | WHR (BMI adjusted) | EUP | T | 0.47 | 0.0390 | 0.0036 | 3.10E-27 |
| rs1385167 | WHR (BMI adjusted) | EUP | G | 0.15 | 0.0290 | 0.0037 | 1.90E-09 |
| rs1440372 | WHR (BMI adjusted) | EUP | C | 0.71 | 0.0240 | 0.0040 | 1.10E-10 |
| rs1443512 | WHR (BMI adjusted) | EUP | A | 0.24 | 0.0280 | 0.0040 | 6.90E-13 |
| rs1534696 | WHR (BMI adjusted) | EUP | C | 0.43 | 0.0110 | 0.0036 | 1.30E-03 |
| rs1569135 | WHR (BMI adjusted) | EUP | A | 0.53 | 0.0210 | 0.0034 | 5.60E-10 |
| rs17451107 | WHR (BMI adjusted) | EUP | T | 0.61 | 0.0260 | 0.0035 | 1.10E-12 |
| rs1776897 | WHR (BMI adjusted) | EUP | G | 0.08 | 0.0300 | 0.0039 | 1.10E-05 |
| rs17819328 | WHR (BMI adjusted) | EUP | G | 0.43 | 0.0210 | 0.0035 | 2.40E-09 |
| rs1936805 | WHR (BMI adjusted) | EUP | T | 0.51 | 0.0430 | 0.0039 | 3.60E-35 |
| rs224333 | WHR (BMI adjusted) | EUP | G | 0.62 | 0.0200 | 0.0043 | 2.60E-08 |
| rs2276824 | WHR (BMI adjusted) | EUP | C | 0.43 | 0.0240 | 0.0049 | 3.20E-11 |
| rs2294239 | WHR (BMI adjusted) | EUP | A | 0.59 | 0.0250 | 0.0036 | 7.20E-13 |
| rs2371767 | WHR (BMI adjusted) | EUP | G | 0.72 | 0.0360 | 0.0034 | 1.60E-20 |
| rs2645294 | WHR (BMI adjusted) | EUP | T | 0.58 | 0.0310 | 0.0051 | 1.70E-19 |
| rs2820443 | WHR (BMI adjusted) | EUP | T | 0.72 | 0.0350 | 0.0034 | 5.30E-21 |
| rs2925979 | WHR (BMI adjusted) | EUP | T | 0.31 | 0.0180 | 0.0054 | 1.20E-06 |
| rs303084 | WHR (BMI adjusted) | EUP | A | 0.80 | 0.0230 | 0.0042 | 3.90E-08 |
| rs3805389 | WHR (BMI adjusted) | EUP | A | 0.28 | 0.0120 | 0.0049 | 1.50E-03 |
| rs4081724 | WHR (BMI adjusted) | EUP | G | 0.85 | 0.0350 | 0.0038 | 7.40E-12 |
| rs4646404 | WHR (BMI adjusted) | EUP | G | 0.67 | 0.0270 | 0.0075 | 1.40E-11 |
| rs4765219 | WHR (BMI adjusted) | EUP | C | 0.67 | 0.0280 | 0.0034 | 1.60E-15 |
| rs6090583 | WHR (BMI adjusted) | EUP | A | 0.48 | 0.0220 | 0.0039 | 6.20E-11 |
| rs6556301 | WHR (BMI adjusted) | EUP | T | 0.36 | 0.0220 | 0.0035 | 2.60E-08 |
| rs714515 | WHR (BMI adjusted) | EUP | G | 0.43 | 0.0270 | 0.0036 | 4.40E-15 |
| rs7705502 | WHR (BMI adjusted) | EUP | A | 0.33 | 0.0270 | 0.0036 | 4.70E-14 |
| rs7759742 | WHR (BMI adjusted) | EUP | A | 0.51 | 0.0230 | 0.0036 | 4.40E-11 |
| rs7801581 | WHR (BMI adjusted) | EUP | T | 0.24 | 0.0270 | 0.0042 | 3.70E-10 |
| rs7830933 | WHR (BMI adjusted) | EUP | A | 0.77 | 0.0220 | 0.0034 | 7.40E-08 |
| rs7917772 | WHR (BMI adjusted) | EUP | A | 0.62 | 0.0140 | 0.0035 | 5.60E-05 |
| rs8030605 | WHR (BMI adjusted) | EUP | A | 0.14 | 0.0300 | 0.0042 | 8.80E-09 |
| rs8042543 | WHR (BMI adjusted) | EUP | C | 0.78 | 0.0260 | 0.0043 | 1.20E-09 |
| rs8066985 | WHR (BMI adjusted) | EUP | A | 0.50 | 0.0180 | 0.0040 | 1.40E-07 |
| rs905938 | WHR (BMI adjusted) | EUP | T | 0.74 | 0.0250 | 0.0040 | 7.30E-10 |
| rs9687846 | WHR (BMI adjusted) | EUP | A | 0.19 | 0.0240 | 0.0036 | 7.10E-08 |
| rs979012 | WHR (BMI adjusted) | EUP | T | 0.34 | 0.0270 | 0.0053 | 3.30E-14 |
| rs9991328 | WHR (BMI adjusted) | EUP | T | 0.49 | 0.0190 | 0.0034 | 4.50E-08 |
| rs9846396 | Infant head circumference | EUP | T | 0.46 | 0.0600 | 0.0100 | 3.35E-08 |
| rs12534093 | Infant head circumference | EUP | A | 0.23 | -0.0800 | 0.0100 | 2.00E-08 |
| rs7048271 | Infant head circumference | EUP | A | 0.15 | 0.1000 | 0.0200 | 9.23E-10 |
| rs1834481 | Interleukin-18 | TRANS | C | 0.25 | 0.1200 | 0.0100 | 5.30E-28 |
| rs5744222 | Interleukin-18 | TRANS | T | 0.25 | -0.1100 | 0.0100 | 8.60E-28 |
| rs10891343 | Interleukin-18 | TRANS | T | 0.48 | 0.1000 | 0.0100 | 4.80E-32 |
| rs2250417 | Interleukin-18 | TRANS | T | 0.48 | 0.1000 | 0.0100 | 1.90E-32 |
| rs2300702 | Interleukin-18 | TRANS | G | 0.42 | 0.0700 | 0.0100 | 1.60E-17 |
| rs2268797 | Interleukin-18 | TRANS | T | 0.42 | 0.0700 | 0.0100 | 2.80E-17 |
| rs6748621 | Interleukin-18 | TRANS | T | 0.40 | 0.0800 | 0.0100 | 1.10E-16 |
| rs6737500 | Interleukin-18 | TRANS | T | 0.41 | 0.0800 | 0.0100 | 1.10E-16 |

| rs12989936 | Interleukin-18 | TRANS | C | 0.41 | 0.0800 | 0.0100 | 2.30E-17 |
| --- | --- | --- | --- | --- | --- | --- | --- |
| rs7577696 | Interleukin-18 | TRANS | G | 0.41 | 0.0800 | 0.0100 | 2.70E-19 |
| rs2280967 | Interleukin-18 | TRANS | T | 0.40 | 0.0700 | 0.0100 | 3.20E-16 |
| rs6760105 | Interleukin-18 | TRANS | G | 0.40 | 0.0600 | 0.0100 | 3.60E-16 |
| rs212745 | Interleukin-18 | TRANS | C | 0.40 | 0.0700 | 0.0100 | 2.10E-15 |
| rs212679 | Interleukin-18 | TRANS | C | 0.40 | 0.0700 | 0.0100 | 1.90E-15 |
| rs212713 | Interleukin-18 | TRANS | C | 0.50 | 0.0600 | 0.0100 | 1.50E-10 |
| rs479333 | Interleukin-18 | TRANS | C | 0.38 | 0.0700 | 0.0100 | 1.30E-15 |
| rs2878349 | High-Density Lipoprotein Cholestero | TRANS | A | 0.33 | -0.0150 | 0.0025 | 2.05E-09 |
| rs12740374 | High-Density Lipoprotein Cholestero | TRANS | T | 0.22 | 0.0454 | 0.0024 | 9.58E-79 |
| rs7550711 | High-Density Lipoprotein Cholestero | TRANS | T | 0.03 | -0.0490 | 0.0059 | 1.63E-16 |
| rs28362581 | High-Density Lipoprotein Cholestero | TRANS | A | 0.08 | -0.0276 | 0.0038 | 4.05E-13 |
| rs333947 | High-Density Lipoprotein Cholestero | TRANS | A | 0.15 | -0.0267 | 0.0034 | 4.32E-15 |
| rs267738 | High-Density Lipoprotein Cholestero | TRANS | T | 0.81 | -0.0239 | 0.0025 | 9.87E-22 |
| rs12145743 | High-Density Lipoprotein Cholestero | TRANS | T | 0.68 | -0.0140 | 0.0022 | 2.00E-10 |
| rs1011731 | High-Density Lipoprotein Cholestero | TRANS | A | 0.57 | 0.0134 | 0.0020 | 1.88E-11 |
| rs4650994 | High-Density Lipoprotein Cholestero | TRANS | A | 0.50 | -0.0193 | 0.0020 | 4.69E-22 |
| rs2243976 | High-Density Lipoprotein Cholestero | TRANS | A | 0.65 | 0.0289 | 0.0024 | 2.00E-33 |
| rs16856110 | High-Density Lipoprotein Cholestero | TRANS | A | 0.78 | 0.0199 | 0.0032 | 5.02E-10 |
| rs6694509 | High-Density Lipoprotein Cholestero | TRANS | T | 0.61 | -0.0195 | 0.0023 | 2.56E-17 |
| rs2807834 | High-Density Lipoprotein Cholestero | TRANS | T | 0.29 | -0.0238 | 0.0025 | 1.77E-21 |
| rs4846914 | High-Density Lipoprotein Cholestero | TRANS | A | 0.57 | 0.0468 | 0.0021 | 9.24E-110 |
| rs1043900 | High-Density Lipoprotein Cholestero | TRANS | A | 0.20 | 0.0234 | 0.0029 | 8.30E-16 |
| rs558971 | High-Density Lipoprotein Cholestero | TRANS | A | 0.47 | -0.0138 | 0.0024 | 8.10E-09 |
| rs1767141 | High-Density Lipoprotein Cholestero | TRANS | A | 0.08 | -0.0263 | 0.0041 | 1.43E-10 |
| rs6668958 | High-Density Lipoprotein Cholestero | TRANS | T | 0.80 | 0.0204 | 0.0029 | 2.35E-12 |
| rs17162330 | High-Density Lipoprotein Cholestero | TRANS | T | 0.86 | 0.0413 | 0.0033 | 1.50E-35 |
| rs12144891 | High-Density Lipoprotein Cholestero | TRANS | A | 0.72 | 0.0168 | 0.0026 | 1.31E-10 |
| rs4660293 | High-Density Lipoprotein Cholestero | TRANS | A | 0.78 | 0.0400 | 0.0024 | 2.46E-62 |
| rs1168089 | High-Density Lipoprotein Cholestero | TRANS | T | 0.33 | -0.0153 | 0.0024 | 1.83E-10 |
| rs4847399 | High-Density Lipoprotein Cholestero | TRANS | A | 0.62 | -0.0216 | 0.0021 | 8.26E-25 |
| rs2862954 | High-Density Lipoprotein Cholestero | TRANS | T | 0.56 | -0.0173 | 0.0020 | 5.98E-18 |
| rs2792751 | High-Density Lipoprotein Cholestero | TRANS | T | 0.28 | 0.0277 | 0.0022 | 2.49E-36 |
| rs2148489 | High-Density Lipoprotein Cholestero | TRANS | T | 0.77 | 0.0223 | 0.0028 | 1.82E-15 |
| rs7076938 | High-Density Lipoprotein Cholestero | TRANS | T | 0.72 | 0.0172 | 0.0022 | 5.22E-15 |
| rs10904908 | High-Density Lipoprotein Cholestero | TRANS | A | 0.56 | -0.0118 | 0.0020 | 4.11E-09 |
| rs970548 | High-Density Lipoprotein Cholestero | TRANS | A | 0.76 | -0.0245 | 0.0023 | 1.72E-26 |
| rs11255744 | High-Density Lipoprotein Cholestero | TRANS | T | 0.73 | -0.0182 | 0.0030 | 1.32E-09 |
| rs2068888 | High-Density Lipoprotein Cholestero | TRANS | A | 0.46 | 0.0205 | 0.0020 | 1.08E-24 |
| rs746463 | High-Density Lipoprotein Cholestero | TRANS | T | 0.69 | -0.0165 | 0.0022 | 6.32E-14 |
| rs180349 | High-Density Lipoprotein Cholestero | TRANS | A | 0.29 | 0.0171 | 0.0027 | 1.83E-10 |
| rs10488698 | High-Density Lipoprotein Cholestero | TRANS | A | 0.06 | 0.0447 | 0.0043 | 1.25E-25 |
| rs964184 | High-Density Lipoprotein Cholestero | TRANS | C | 0.85 | 0.1321 | 0.0031 | 0.00E+00 |
| rs138326449 | High-Density Lipoprotein Cholestero | TRANS | A | 0.00 | 0.7155 | 0.0257 | 2.11E-170 |
| rs138407155 | High-Density Lipoprotein Cholestero | TRANS | A | 1.00 | 0.3555 | 0.0420 | 2.45E-17 |
| rs12281729 | High-Density Lipoprotein Cholestero | TRANS | A | 0.94 | 0.0769 | 0.0052 | 3.49E-49 |
| rs10892063 | High-Density Lipoprotein Cholestero | TRANS | A | 0.39 | 0.0418 | 0.0030 | 9.37E-45 |
| rs12269901 | High-Density Lipoprotein Cholestero | TRANS | C | 0.32 | 0.0268 | 0.0027 | 3.27E-23 |
| rs593245 | High-Density Lipoprotein Cholestero | TRANS | T | 0.44 | 0.0146 | 0.0024 | 1.99E-09 |
| rs7941030 | High-Density Lipoprotein Cholestero | TRANS | T | 0.62 | -0.0229 | 0.0020 | 3.26E-30 |
| rs4937122 | High-Density Lipoprotein Cholestero | TRANS | T | 0.92 | 0.0317 | 0.0044 | 6.02E-13 |
| rs6486121 | High-Density Lipoprotein Cholestero | TRANS | T | 0.64 | -0.0152 | 0.0024 | 2.48E-10 |
| rs2303975 | High-Density Lipoprotein Cholestero | TRANS | A | 0.12 | 0.0192 | 0.0030 | 1.43E-10 |
| rs925946 | High-Density Lipoprotein Cholestero | TRANS | T | 0.30 | -0.0122 | 0.0021 | 6.58E-09 |
| rs16928809 | High-Density Lipoprotein Cholestero | TRANS | A | 0.09 | -0.0246 | 0.0036 | 7.72E-12 |
| rs7927401 | High-Density Lipoprotein Cholestero | TRANS | T | 0.24 | -0.0210 | 0.0031 | 1.24E-11 |
| rs3824866 | High-Density Lipoprotein Cholestero | TRANS | T | 0.16 | 0.0331 | 0.0037 | 4.45E-19 |
| rs326214 | High-Density Lipoprotein Cholestero | TRANS | A | 0.66 | -0.0248 | 0.0027 | 1.02E-20 |

| rs10838738 | High-Density Lipoprotein Cholestero | TRANS | A | 0.66 | 0.0193 | 0.0023 | 1.60E-17 |
| --- | --- | --- | --- | --- | --- | --- | --- |
| rs61897793 | High-Density Lipoprotein Cholestero | TRANS | A | 0.17 | 0.0238 | 0.0040 | 3.68E-09 |
| rs174583 | High-Density Lipoprotein Cholestero | TRANS | T | 0.34 | -0.0424 | 0.0024 | 1.08E-68 |
| rs35169799 | High-Density Lipoprotein Cholestero | TRANS | T | 0.06 | -0.0396 | 0.0041 | 5.00E-22 |
| rs644740 | High-Density Lipoprotein Cholestero | TRANS | T | 0.45 | 0.0160 | 0.0023 | 3.87E-12 |
| rs622082 | High-Density Lipoprotein Cholestero | TRANS | A | 0.69 | 0.0155 | 0.0021 | 1.63E-13 |
| rs499974 | High-Density Lipoprotein Cholestero | TRANS | A | 0.18 | -0.0254 | 0.0026 | 1.47E-22 |
| rs140201358 | High-Density Lipoprotein Cholestero | TRANS | C | 0.99 | 0.0523 | 0.0088 | 2.83E-09 |
| rs2373459 | High-Density Lipoprotein Cholestero | TRANS | T | 0.66 | 0.0160 | 0.0024 | 2.88E-11 |
| rs7298565 | High-Density Lipoprotein Cholestero | TRANS | A | 0.53 | 0.0280 | 0.0020 | 1.81E-44 |
| rs3184504 | High-Density Lipoprotein Cholestero | TRANS | T | 0.46 | -0.0243 | 0.0021 | 4.98E-31 |
| rs72650673 | High-Density Lipoprotein Cholestero | TRANS | A | 0.00 | -0.1716 | 0.0283 | 1.36E-09 |
| rs1183910 | High-Density Lipoprotein Cholestero | TRANS | A | 0.32 | 0.0127 | 0.0021 | 1.72E-09 |
| rs12369179 | High-Density Lipoprotein Cholestero | TRANS | T | 0.09 | -0.0310 | 0.0049 | 3.77E-10 |
| rs1798192 | High-Density Lipoprotein Cholestero | TRANS | T | 0.44 | -0.0193 | 0.0020 | 3.60E-21 |
| rs940904 | High-Density Lipoprotein Cholestero | TRANS | A | 0.74 | -0.0226 | 0.0027 | 1.81E-16 |
| rs4759375 | High-Density Lipoprotein Cholestero | TRANS | T | 0.10 | 0.0487 | 0.0039 | 5.98E-35 |
| rs12317176 | High-Density Lipoprotein Cholestero | TRANS | T | 0.65 | -0.0224 | 0.0024 | 5.21E-20 |
| rs863750 | High-Density Lipoprotein Cholestero | TRANS | T | 0.59 | -0.0172 | 0.0024 | 3.10E-13 |
| rs12230272 | High-Density Lipoprotein Cholestero | TRANS | A | 0.79 | 0.0223 | 0.0034 | 5.72E-11 |
| rs838880 | High-Density Lipoprotein Cholestero | TRANS | T | 0.65 | -0.0249 | 0.0022 | 6.30E-31 |
| rs10773105 | High-Density Lipoprotein Cholestero | TRANS | T | 0.51 | -0.0255 | 0.0024 | 3.61E-27 |
| rs150728540 | High-Density Lipoprotein Cholestero | TRANS | A | 0.00 | 0.4756 | 0.0576 | 1.58E-16 |
| rs5891 | High-Density Lipoprotein Cholestero | TRANS | T | 0.01 | 0.0776 | 0.0089 | 2.21E-18 |
| rs7306660 | High-Density Lipoprotein Cholestero | TRANS | A | 0.36 | -0.0286 | 0.0024 | 2.44E-32 |
| rs7298751 | High-Density Lipoprotein Cholestero | TRANS | A | 0.88 | -0.0426 | 0.0036 | 5.11E-32 |
| rs7134375 | High-Density Lipoprotein Cholestero | TRANS | A | 0.42 | 0.0215 | 0.0020 | 6.24E-27 |
| rs7134150 | High-Density Lipoprotein Cholestero | TRANS | A | 0.07 | -0.0314 | 0.0048 | 6.33E-11 |
| rs4963975 | High-Density Lipoprotein Cholestero | TRANS | A | 0.24 | -0.0198 | 0.0027 | 2.32E-13 |
| rs1126930 | High-Density Lipoprotein Cholestero | TRANS | C | 0.03 | -0.0373 | 0.0056 | 2.89E-11 |
| rs784563 | High-Density Lipoprotein Cholestero | TRANS | T | 0.54 | 0.0153 | 0.0023 | 3.09E-11 |
| rs11613352 | High-Density Lipoprotein Cholestero | TRANS | T | 0.21 | 0.0226 | 0.0027 | 5.38E-17 |
| rs17532301 | High-Density Lipoprotein Cholestero | TRANS | A | 0.07 | -0.0266 | 0.0045 | 3.40E-09 |
| rs4983559 | High-Density Lipoprotein Cholestero | TRANS | A | 0.59 | -0.0252 | 0.0020 | 2.16E-36 |
| rs10483776 | High-Density Lipoprotein Cholestero | TRANS | A | 0.83 | 0.0153 | 0.0026 | 3.89E-09 |
| rs8021180 | High-Density Lipoprotein Cholestero | TRANS | A | 0.54 | 0.0142 | 0.0024 | 3.32E-09 |
| rs13379043 | High-Density Lipoprotein Cholestero | TRANS | T | 0.70 | -0.0176 | 0.0022 | 1.40E-15 |
| rs9944249 | High-Density Lipoprotein Cholestero | TRANS | T | 0.43 | -0.0132 | 0.0023 | 9.05E-09 |
| rs55707100 | High-Density Lipoprotein Cholestero | TRANS | T | 0.03 | -0.0934 | 0.0062 | 3.08E-51 |
| rs4622454 | High-Density Lipoprotein Cholestero | TRANS | T | 0.36 | 0.0205 | 0.0027 | 1.03E-14 |
| rs4775041 | High-Density Lipoprotein Cholestero | TRANS | C | 0.29 | 0.0388 | 0.0039 | 9.11E-24 |
| rs16940147 | High-Density Lipoprotein Cholestero | TRANS | A | 0.05 | 0.0499 | 0.0082 | 1.07E-09 |
| rs117901517 | High-Density Lipoprotein Cholestero | TRANS | T | 0.93 | 0.0378 | 0.0060 | 3.18E-10 |
| rs34718390 | High-Density Lipoprotein Cholestero | TRANS | A | 0.06 | 0.0528 | 0.0060 | 1.07E-18 |
| rs1532085 | High-Density Lipoprotein Cholestero | TRANS | A | 0.40 | 0.0620 | 0.0036 | 2.93E-68 |
| rs7165077 | High-Density Lipoprotein Cholestero | TRANS | T | 0.13 | -0.0273 | 0.0038 | 8.47E-13 |
| rs6494003 | High-Density Lipoprotein Cholestero | TRANS | A | 0.98 | 0.0927 | 0.0107 | 5.04E-18 |
| rs16940233 | High-Density Lipoprotein Cholestero | TRANS | T | 0.92 | -0.0478 | 0.0056 | 7.00E-18 |
| rs12912415 | High-Density Lipoprotein Cholestero | TRANS | A | 0.85 | 0.0400 | 0.0038 | 2.59E-25 |
| rs6494006 | High-Density Lipoprotein Cholestero | TRANS | T | 0.93 | 0.0436 | 0.0065 | 1.66E-11 |
| rs17301746 | High-Density Lipoprotein Cholestero | TRANS | T | 0.02 | 0.0879 | 0.0097 | 8.64E-20 |
| rs936960 | High-Density Lipoprotein Cholestero | TRANS | T | 0.09 | 0.0524 | 0.0045 | 7.92E-32 |
| rs1869138 | High-Density Lipoprotein Cholestero | TRANS | T | 0.90 | -0.0281 | 0.0042 | 3.13E-11 |
| rs6083 | High-Density Lipoprotein Cholestero | TRANS | A | 0.61 | -0.0162 | 0.0028 | 5.00E-09 |
| rs17269397 | High-Density Lipoprotein Cholestero | TRANS | A | 0.45 | -0.0261 | 0.0030 | 6.37E-18 |
| rs424346 | High-Density Lipoprotein Cholestero | TRANS | T | 0.04 | 0.0545 | 0.0091 | 2.20E-09 |
| rs181181625 | High-Density Lipoprotein Cholestero | TRANS | T | 0.00 | 0.3478 | 0.0326 | 1.27E-26 |
| rs12148597 | High-Density Lipoprotein Cholestero | TRANS | A | 0.23 | -0.0158 | 0.0027 | 5.27E-09 |

| rs34317102 | High-Density Lipoprotein Cholestero | TRANS | A | 0.23 | -0.0190 | 0.0023 | 1.49E-16 |
| --- | --- | --- | --- | --- | --- | --- | --- |
| rs2228510 | High-Density Lipoprotein Cholestero | TRANS | T | 0.48 | -0.0117 | 0.0020 | 4.62E-09 |
| rs139271800 | High-Density Lipoprotein Cholestero | TRANS | A | 1.00 | -0.2482 | 0.0374 | 3.22E-11 |
| rs12928822 | High-Density Lipoprotein Cholestero | TRANS | T | 0.16 | 0.0179 | 0.0028 | 1.63E-10 |
| rs1421085 | High-Density Lipoprotein Cholestero | TRANS | T | 0.60 | 0.0282 | 0.0020 | 3.35E-45 |
| rs12929759 | High-Density Lipoprotein Cholestero | TRANS | A | 0.68 | 0.0183 | 0.0025 | 2.97E-13 |
| rs4238772 | High-Density Lipoprotein Cholestero | TRANS | A | 0.09 | 0.0286 | 0.0047 | 1.23E-09 |
| rs8044753 | High-Density Lipoprotein Cholestero | TRANS | A | 0.52 | 0.0248 | 0.0028 | 3.92E-19 |
| rs36049418 | High-Density Lipoprotein Cholestero | TRANS | A | 0.02 | 0.0656 | 0.0085 | 1.36E-14 |
| rs11648751 | High-Density Lipoprotein Cholestero | TRANS | T | 0.12 | -0.0304 | 0.0045 | 1.01E-11 |
| rs37029 | High-Density Lipoprotein Cholestero | TRANS | A | 0.44 | -0.0242 | 0.0025 | 2.01E-21 |
| rs9989419 | High-Density Lipoprotein Cholestero | TRANS | A | 0.40 | -0.0336 | 0.0025 | 1.45E-41 |
| rs72786786 | High-Density Lipoprotein Cholestero | TRANS | A | 0.30 | 0.0577 | 0.0043 | 3.00E-40 |
| rs76315536 | High-Density Lipoprotein Cholestero | TRANS | T | 0.01 | 0.1614 | 0.0157 | 1.14E-24 |
| rs4783961 | High-Density Lipoprotein Cholestero | TRANS | A | 0.49 | 0.0260 | 0.0037 | 2.07E-12 |
| rs1800775 | High-Density Lipoprotein Cholestero | TRANS | A | 0.50 | 0.0317 | 0.0048 | 3.69E-11 |
| rs34065661 | High-Density Lipoprotein Cholestero | TRANS | C | 1.00 | -0.4847 | 0.0193 | 1.45E-138 |
| rs7203984 | High-Density Lipoprotein Cholestero | TRANS | A | 0.79 | 0.0741 | 0.0047 | 2.19E-56 |
| rs1532624 | High-Density Lipoprotein Cholestero | TRANS | A | 0.42 | 0.1128 | 0.0048 | 1.68E-121 |
| rs12708974 | High-Density Lipoprotein Cholestero | TRANS | T | 0.12 | -0.0857 | 0.0047 | 4.48E-73 |
| rs5883 | High-Density Lipoprotein Cholestero | TRANS | T | 0.06 | 0.1674 | 0.0058 | 4.70E-186 |
| rs289714 | High-Density Lipoprotein Cholestero | TRANS | A | 0.80 | 0.0822 | 0.0047 | 8.38E-70 |
| rs5880 | High-Density Lipoprotein Cholestero | TRANS | C | 0.05 | -0.1378 | 0.0053 | 1.46E-147 |
| rs506829 | High-Density Lipoprotein Cholestero | TRANS | T | 0.78 | 0.0192 | 0.0032 | 2.21E-09 |
| rs16962034 | High-Density Lipoprotein Cholestero | TRANS | T | 0.39 | -0.0157 | 0.0027 | 6.24E-09 |
| rs7202185 | High-Density Lipoprotein Cholestero | TRANS | A | 0.07 | -0.0373 | 0.0049 | 3.36E-14 |
| rs16942887 | High-Density Lipoprotein Cholestero | TRANS | A | 0.13 | 0.0742 | 0.0030 | 3.15E-134 |
| rs1345868 | High-Density Lipoprotein Cholestero | TRANS | A | 0.31 | 0.0144 | 0.0025 | 7.81E-09 |
| rs12443634 | High-Density Lipoprotein Cholestero | TRANS | A | 0.28 | -0.0313 | 0.0030 | 2.03E-25 |
| rs7202647 | High-Density Lipoprotein Cholestero | TRANS | T | 0.27 | 0.0170 | 0.0029 | 4.58E-09 |
| rs2071379 | High-Density Lipoprotein Cholestero | TRANS | A | 0.42 | 0.0143 | 0.0023 | 5.06E-10 |
| rs11078915 | High-Density Lipoprotein Cholestero | TRANS | T | 0.23 | -0.0267 | 0.0037 | 6.37E-13 |
| rs11869286 | High-Density Lipoprotein Cholestero | TRANS | C | 0.64 | 0.0239 | 0.0027 | 1.81E-18 |
| rs11556624 | High-Density Lipoprotein Cholestero | TRANS | C | 0.02 | 0.0867 | 0.0068 | 8.89E-37 |
| rs4794822 | High-Density Lipoprotein Cholestero | TRANS | T | 0.38 | -0.0177 | 0.0021 | 1.92E-16 |
| rs72836561 | High-Density Lipoprotein Cholestero | TRANS | T | 0.03 | -0.1875 | 0.0058 | 7.63E-228 |
| rs231539 | High-Density Lipoprotein Cholestero | TRANS | T | 0.17 | -0.0357 | 0.0036 | 4.17E-23 |
| rs17679445 | High-Density Lipoprotein Cholestero | TRANS | A | 0.07 | 0.0295 | 0.0041 | 6.13E-13 |
| rs11652146 | High-Density Lipoprotein Cholestero | TRANS | A | 0.69 | -0.0172 | 0.0025 | 6.65E-12 |
| rs12602912 | High-Density Lipoprotein Cholestero | TRANS | T | 0.21 | -0.0176 | 0.0024 | 2.17E-13 |
| rs10852765 | High-Density Lipoprotein Cholestero | TRANS | A | 0.43 | 0.0156 | 0.0023 | 1.10E-11 |
| rs3803800 | High-Density Lipoprotein Cholestero | TRANS | A | 0.24 | -0.0134 | 0.0023 | 5.68E-09 |
| rs2292642 | High-Density Lipoprotein Cholestero | TRANS | T | 0.61 | 0.0319 | 0.0020 | 1.57E-55 |
| rs2289750 | High-Density Lipoprotein Cholestero | TRANS | A | 0.09 | -0.0283 | 0.0049 | 6.55E-09 |
| rs1788783 | High-Density Lipoprotein Cholestero | TRANS | T | 0.48 | 0.0150 | 0.0023 | 6.97E-11 |
| rs8093249 | High-Density Lipoprotein Cholestero | TRANS | A | 0.84 | 0.0337 | 0.0035 | 9.51E-22 |
| rs77960347 | High-Density Lipoprotein Cholestero | TRANS | A | 0.99 | -0.2505 | 0.0091 | 7.67E-166 |
| rs117623631 | High-Density Lipoprotein Cholestero | TRANS | T | 0.00 | 0.3432 | 0.0254 | 1.05E-41 |
| rs3786248 | High-Density Lipoprotein Cholestero | TRANS | T | 0.95 | -0.0590 | 0.0056 | 2.81E-26 |
| rs7241918 | High-Density Lipoprotein Cholestero | TRANS | T | 0.84 | 0.0741 | 0.0032 | 4.32E-120 |
| rs4939886 | High-Density Lipoprotein Cholestero | TRANS | A | 0.36 | 0.0198 | 0.0032 | 8.81E-10 |
| rs11660468 | High-Density Lipoprotein Cholestero | TRANS | T | 0.40 | 0.0253 | 0.0024 | 1.21E-25 |
| rs9956279 | High-Density Lipoprotein Cholestero | TRANS | T | 0.29 | -0.0168 | 0.0026 | 1.04E-10 |
| rs6511720 | High-Density Lipoprotein Cholestero | TRANS | T | 0.11 | 0.0201 | 0.0031 | 1.13E-10 |
| rs17616661 | High-Density Lipoprotein Cholestero | TRANS | A | 0.92 | 0.0258 | 0.0036 | 1.17E-12 |
| rs737337 | High-Density Lipoprotein Cholestero | TRANS | T | 0.90 | 0.0570 | 0.0034 | 1.25E-62 |
| rs2111504 | High-Density Lipoprotein Cholestero | TRANS | A | 0.17 | 0.0167 | 0.0026 | 1.39E-10 |
| rs731839 | High-Density Lipoprotein Cholestero | TRANS | A | 0.65 | 0.0178 | 0.0021 | 2.42E-17 |

| rs12975319 | High-Density Lipoprotein Cholestero | TRANS | A | 0.31 | -0.0153 | 0.0026 | 3.66E-09 |
| --- | --- | --- | --- | --- | --- | --- | --- |
| rs2075650 | High-Density Lipoprotein Cholestero | TRANS | A | 0.86 | 0.0379 | 0.0035 | 7.69E-28 |
| rs77301115 | High-Density Lipoprotein Cholestero | TRANS | A | 0.03 | -0.0660 | 0.0090 | 1.96E-13 |
| rs7412 | High-Density Lipoprotein Cholestero | TRANS | T | 0.08 | 0.0856 | 0.0044 | 3.01E-85 |
| rs439401 | High-Density Lipoprotein Cholestero | TRANS | T | 0.37 | 0.0141 | 0.0022 | 7.87E-11 |
| rs4420638 | High-Density Lipoprotein Cholestero | TRANS | A | 0.82 | 0.0420 | 0.0037 | 2.94E-29 |
| rs5167 | High-Density Lipoprotein Cholestero | TRANS | T | 0.63 | -0.0376 | 0.0020 | 2.37E-78 |
| rs8111071 | High-Density Lipoprotein Cholestero | TRANS | A | 0.92 | 0.0278 | 0.0036 | 1.14E-14 |
| rs2303108 | High-Density Lipoprotein Cholestero | TRANS | T | 0.32 | 0.0137 | 0.0021 | 7.92E-11 |
| rs3752125 | High-Density Lipoprotein Cholestero | TRANS | T | 0.29 | -0.0204 | 0.0025 | 3.36E-16 |
| rs12975366 | High-Density Lipoprotein Cholestero | TRANS | T | 0.60 | 0.0212 | 0.0023 | 1.36E-19 |
| rs386000 | High-Density Lipoprotein Cholestero | TRANS | C | 0.21 | 0.0437 | 0.0030 | 5.74E-48 |
| rs12979085 | High-Density Lipoprotein Cholestero | TRANS | A | 0.29 | 0.0204 | 0.0029 | 1.06E-12 |
| rs10408844 | High-Density Lipoprotein Cholestero | TRANS | T | 0.80 | 0.0180 | 0.0029 | 5.19E-10 |
| rs4804833 | High-Density Lipoprotein Cholestero | TRANS | A | 0.40 | -0.0150 | 0.0024 | 4.89E-10 |
| rs116843064 | High-Density Lipoprotein Cholestero | TRANS | A | 0.02 | 0.2456 | 0.0072 | 1.25E-252 |
| rs2913968 | High-Density Lipoprotein Cholestero | TRANS | T | 0.72 | 0.0166 | 0.0026 | 1.95E-10 |
| rs13389219 | High-Density Lipoprotein Cholestero | TRANS | T | 0.40 | 0.0327 | 0.0021 | 1.21E-54 |
| rs6435161 | High-Density Lipoprotein Cholestero | TRANS | T | 0.71 | 0.0177 | 0.0024 | 1.65E-13 |
| rs676210 | High-Density Lipoprotein Cholestero | TRANS | A | 0.24 | 0.0617 | 0.0024 | 4.71E-143 |
| rs562338 | High-Density Lipoprotein Cholestero | TRANS | A | 0.19 | 0.0178 | 0.0025 | 1.85E-12 |
| rs2943641 | High-Density Lipoprotein Cholestero | TRANS | T | 0.35 | 0.0391 | 0.0021 | 2.52E-77 |
| rs11553746 | High-Density Lipoprotein Cholestero | TRANS | T | 0.33 | 0.0158 | 0.0021 | 5.70E-14 |
| rs4850047 | High-Density Lipoprotein Cholestero | TRANS | T | 0.15 | 0.0201 | 0.0033 | 1.03E-09 |
| rs36020289 | High-Density Lipoprotein Cholestero | TRANS | C | 0.02 | 0.0462 | 0.0077 | 1.98E-09 |
| rs2867125 | High-Density Lipoprotein Cholestero | TRANS | T | 0.17 | 0.0173 | 0.0026 | 3.25E-11 |
| rs12990465 | High-Density Lipoprotein Cholestero | TRANS | T | 0.26 | 0.0263 | 0.0030 | 1.86E-18 |
| rs1132274 | High-Density Lipoprotein Cholestero | TRANS | A | 0.16 | -0.0159 | 0.0027 | 3.89E-09 |
| rs2268086 | High-Density Lipoprotein Cholestero | TRANS | A | 0.38 | 0.0144 | 0.0024 | 3.41E-09 |
| rs1415771 | High-Density Lipoprotein Cholestero | TRANS | A | 0.46 | -0.0129 | 0.0020 | 1.90E-10 |
| rs1800961 | High-Density Lipoprotein Cholestero | TRANS | T | 0.03 | -0.1421 | 0.0057 | 5.06E-137 |
| rs3827066 | High-Density Lipoprotein Cholestero | TRANS | T | 0.16 | 0.0496 | 0.0039 | 4.16E-37 |
| rs8123864 | High-Density Lipoprotein Cholestero | TRANS | T | 0.66 | 0.0477 | 0.0030 | 2.19E-55 |
| rs1211644 | High-Density Lipoprotein Cholestero | TRANS | T | 0.75 | -0.0178 | 0.0030 | 3.16E-09 |
| rs11700063 | High-Density Lipoprotein Cholestero | TRANS | A | 0.26 | 0.0206 | 0.0027 | 3.29E-14 |
| rs4239651 | High-Density Lipoprotein Cholestero | TRANS | T | 0.19 | -0.0210 | 0.0034 | 8.38E-10 |
| rs6025606 | High-Density Lipoprotein Cholestero | TRANS | T | 0.36 | 0.0119 | 0.0020 | 2.93E-09 |
| rs310631 | High-Density Lipoprotein Cholestero | TRANS | A | 0.41 | 0.0133 | 0.0023 | 7.42E-09 |
| rs6062343 | High-Density Lipoprotein Cholestero | TRANS | A | 0.43 | 0.0137 | 0.0020 | 7.57E-12 |
| rs235314 | High-Density Lipoprotein Cholestero | TRANS | T | 0.54 | -0.0150 | 0.0023 | 7.15E-11 |
| rs12482088 | High-Density Lipoprotein Cholestero | TRANS | A | 0.77 | 0.0187 | 0.0030 | 4.97E-10 |
| rs181362 | High-Density Lipoprotein Cholestero | TRANS | T | 0.23 | -0.0303 | 0.0024 | 1.29E-36 |
| rs4823006 | High-Density Lipoprotein Cholestero | TRANS | A | 0.55 | -0.0130 | 0.0020 | 9.08E-11 |
| rs17738540 | High-Density Lipoprotein Cholestero | TRANS | T | 0.22 | -0.0184 | 0.0024 | 1.63E-14 |
| rs738322 | High-Density Lipoprotein Cholestero | TRANS | A | 0.52 | -0.0198 | 0.0020 | 4.24E-23 |
| rs738409 | High-Density Lipoprotein Cholestero | TRANS | C | 0.77 | 0.0155 | 0.0023 | 1.47E-11 |
| rs11712666 | High-Density Lipoprotein Cholestero | TRANS | A | 0.46 | -0.0141 | 0.0023 | 1.01E-09 |
| rs35000036 | High-Density Lipoprotein Cholestero | TRANS | T | 0.38 | -0.0132 | 0.0021 | 3.27E-10 |
| rs2067819 | High-Density Lipoprotein Cholestero | TRANS | A | 0.22 | 0.0211 | 0.0028 | 5.63E-14 |
| rs2292101 | High-Density Lipoprotein Cholestero | TRANS | T | 0.04 | -0.0438 | 0.0065 | 1.84E-11 |
| rs1279840 | High-Density Lipoprotein Cholestero | TRANS | T | 0.24 | 0.0280 | 0.0027 | 3.43E-25 |
| rs3773910 | High-Density Lipoprotein Cholestero | TRANS | C | 0.75 | -0.0169 | 0.0027 | 4.08E-10 |
| rs900399 | High-Density Lipoprotein Cholestero | TRANS | A | 0.61 | -0.0213 | 0.0020 | 1.37E-26 |
| rs7633675 | High-Density Lipoprotein Cholestero | TRANS | T | 0.66 | 0.0133 | 0.0021 | 2.19E-10 |
| rs4234589 | High-Density Lipoprotein Cholestero | TRANS | A | 0.86 | 0.0222 | 0.0034 | 6.21E-11 |
| rs6777217 | High-Density Lipoprotein Cholestero | TRANS | A | 0.46 | -0.0129 | 0.0020 | 1.12E-10 |
| rs2305637 | High-Density Lipoprotein Cholestero | TRANS | T | 0.17 | -0.0264 | 0.0029 | 1.06E-19 |
| rs6762477 | High-Density Lipoprotein Cholestero | TRANS | A | 0.58 | 0.0232 | 0.0021 | 2.14E-28 |

| rs13326165 | High-Density Lipoprotein Cholestero | TRANS | A | 0.20 | 0.0213 | 0.0026 | 1.26E-16 |
| --- | --- | --- | --- | --- | --- | --- | --- |
| rs11242 | High-Density Lipoprotein Cholestero | TRANS | T | 0.43 | 0.0150 | 0.0024 | 2.35E-10 |
| rs2602836 | High-Density Lipoprotein Cholestero | TRANS | A | 0.42 | 0.0127 | 0.0020 | 2.04E-10 |
| rs112519623 | High-Density Lipoprotein Cholestero | TRANS | A | 0.01 | -0.0472 | 0.0082 | 8.80E-09 |
| rs13107325 | High-Density Lipoprotein Cholestero | TRANS | T | 0.07 | -0.0777 | 0.0041 | 6.35E-80 |
| rs6855363 | High-Density Lipoprotein Cholestero | TRANS | T | 0.66 | -0.0185 | 0.0024 | 1.28E-14 |
| rs10019888 | High-Density Lipoprotein Cholestero | TRANS | A | 0.83 | 0.0243 | 0.0031 | 4.57E-15 |
| rs293429 | High-Density Lipoprotein Cholestero | TRANS | T | 0.70 | -0.0133 | 0.0022 | 1.49E-09 |
| rs11248051 | High-Density Lipoprotein Cholestero | TRANS | T | 0.10 | -0.0190 | 0.0033 | 8.54E-09 |
| rs10023050 | High-Density Lipoprotein Cholestero | TRANS | A | 0.60 | -0.0152 | 0.0023 | 3.66E-11 |
| rs3822072 | High-Density Lipoprotein Cholestero | TRANS | A | 0.47 | -0.0214 | 0.0023 | 1.36E-20 |
| rs4705986 | High-Density Lipoprotein Cholestero | TRANS | T | 0.94 | -0.0348 | 0.0058 | 1.98E-09 |
| rs390299 | High-Density Lipoprotein Cholestero | TRANS | A | 0.64 | -0.0145 | 0.0021 | 4.56E-12 |
| rs2434612 | High-Density Lipoprotein Cholestero | TRANS | A | 0.79 | 0.0225 | 0.0028 | 8.11E-16 |
| rs7730898 | High-Density Lipoprotein Cholestero | TRANS | A | 0.73 | -0.0183 | 0.0026 | 1.95E-12 |
| rs7735253 | High-Density Lipoprotein Cholestero | TRANS | A | 0.26 | -0.0210 | 0.0026 | 6.46E-16 |
| rs459193 | High-Density Lipoprotein Cholestero | TRANS | A | 0.27 | 0.0265 | 0.0022 | 9.16E-33 |
| rs9686661 | High-Density Lipoprotein Cholestero | TRANS | T | 0.19 | -0.0343 | 0.0025 | 3.45E-42 |
| rs4976033 | High-Density Lipoprotein Cholestero | TRANS | A | 0.58 | 0.0134 | 0.0020 | 1.83E-11 |
| rs10057967 | High-Density Lipoprotein Cholestero | TRANS | T | 0.63 | -0.0205 | 0.0027 | 2.77E-14 |
| rs2754820 | High-Density Lipoprotein Cholestero | TRANS | A | 0.20 | 0.0196 | 0.0033 | 3.14E-09 |
| rs884366 | High-Density Lipoprotein Cholestero | TRANS | A | 0.31 | -0.0143 | 0.0022 | 8.14E-11 |
| rs3756772 | High-Density Lipoprotein Cholestero | TRANS | T | 0.42 | 0.0128 | 0.0020 | 1.56E-10 |
| rs2745353 | High-Density Lipoprotein Cholestero | TRANS | T | 0.52 | -0.0202 | 0.0020 | 6.49E-24 |
| rs6925103 | High-Density Lipoprotein Cholestero | TRANS | T | 0.51 | -0.0121 | 0.0020 | 1.26E-09 |
| rs643381 | High-Density Lipoprotein Cholestero | TRANS | A | 0.51 | 0.0208 | 0.0020 | 2.52E-25 |
| rs41272114 | High-Density Lipoprotein Cholestero | TRANS | T | 0.03 | 0.0663 | 0.0061 | 3.35E-27 |
| rs1652507 | High-Density Lipoprotein Cholestero | TRANS | T | 0.83 | 0.0460 | 0.0031 | 9.57E-51 |
| rs1265099 | High-Density Lipoprotein Cholestero | TRANS | A | 0.59 | 0.0170 | 0.0021 | 1.23E-16 |
| rs184070214 | High-Density Lipoprotein Cholestero | TRANS | A | 0.02 | -0.0445 | 0.0074 | 1.48E-09 |
| rs9332739 | High-Density Lipoprotein Cholestero | TRANS | C | 0.04 | -0.0290 | 0.0048 | 1.84E-09 |
| rs3135006 | High-Density Lipoprotein Cholestero | TRANS | T | 0.24 | -0.0195 | 0.0027 | 3.03E-13 |
| rs2894342 | High-Density Lipoprotein Cholestero | TRANS | A | 0.23 | 0.0146 | 0.0024 | 1.31E-09 |
| rs1759645 | High-Density Lipoprotein Cholestero | TRANS | T | 0.84 | 0.0239 | 0.0033 | 3.27E-13 |
| rs16885998 | High-Density Lipoprotein Cholestero | TRANS | T | 0.05 | 0.0506 | 0.0062 | 2.98E-16 |
| rs11755393 | High-Density Lipoprotein Cholestero | TRANS | A | 0.64 | 0.0304 | 0.0022 | 5.92E-45 |
| rs41270076 | High-Density Lipoprotein Cholestero | TRANS | T | 0.03 | 0.0386 | 0.0062 | 6.24E-10 |
| rs4711698 | High-Density Lipoprotein Cholestero | TRANS | T | 0.74 | 0.0180 | 0.0027 | 2.78E-11 |
| rs2274517 | High-Density Lipoprotein Cholestero | TRANS | T | 0.54 | 0.0156 | 0.0023 | 1.36E-11 |
| rs6905288 | High-Density Lipoprotein Cholestero | TRANS | A | 0.58 | -0.0295 | 0.0020 | 5.52E-49 |
| rs35349911 | High-Density Lipoprotein Cholestero | TRANS | T | 0.57 | -0.0138 | 0.0020 | 1.03E-11 |
| rs881858 | High-Density Lipoprotein Cholestero | TRANS | A | 0.69 | -0.0138 | 0.0021 | 9.49E-11 |
| rs1997243 | High-Density Lipoprotein Cholestero | TRANS | A | 0.85 | -0.0222 | 0.0028 | 2.19E-15 |
| rs11556924 | High-Density Lipoprotein Cholestero | TRANS | T | 0.35 | 0.0126 | 0.0021 | 1.97E-09 |
| rs972283 | High-Density Lipoprotein Cholestero | TRANS | A | 0.46 | 0.0276 | 0.0020 | 2.94E-43 |
| rs3735080 | High-Density Lipoprotein Cholestero | TRANS | T | 0.23 | -0.0137 | 0.0023 | 2.65E-09 |
| rs7787577 | High-Density Lipoprotein Cholestero | TRANS | A | 0.91 | 0.0314 | 0.0046 | 8.34E-12 |
| rs10282707 | High-Density Lipoprotein Cholestero | TRANS | T | 0.42 | -0.0258 | 0.0022 | 7.65E-32 |
| rs1534696 | High-Density Lipoprotein Cholestero | TRANS | A | 0.56 | 0.0185 | 0.0025 | 1.30E-13 |
| rs2726070 | High-Density Lipoprotein Cholestero | TRANS | A | 0.45 | -0.0172 | 0.0027 | 1.97E-10 |
| rs4917014 | High-Density Lipoprotein Cholestero | TRANS | T | 0.69 | -0.0138 | 0.0021 | 4.99E-11 |
| rs2303361 | High-Density Lipoprotein Cholestero | TRANS | T | 0.78 | -0.0238 | 0.0024 | 4.10E-23 |
| rs1178979 | High-Density Lipoprotein Cholestero | TRANS | T | 0.81 | -0.0335 | 0.0025 | 6.22E-41 |
| rs2957447 | High-Density Lipoprotein Cholestero | TRANS | A | 0.45 | 0.0138 | 0.0024 | 8.94E-09 |
| rs9657541 | High-Density Lipoprotein Cholestero | TRANS | T | 0.21 | -0.0246 | 0.0029 | 3.56E-17 |
| rs2293889 | High-Density Lipoprotein Cholestero | TRANS | T | 0.40 | -0.0304 | 0.0020 | 2.66E-52 |
| rs4871137 | High-Density Lipoprotein Cholestero | TRANS | T | 0.65 | -0.0213 | 0.0022 | 4.41E-22 |
| rs17405319 | High-Density Lipoprotein Cholestero | TRANS | T | 0.17 | -0.0252 | 0.0031 | 9.05E-16 |

| rs2954026 | High-Density Lipoprotein Cholestero | TRANS | T | 0.29 | -0.0493 | 0.0022 | 1.29E-108 |
| --- | --- | --- | --- | --- | --- | --- | --- |
| rs1801177 | High-Density Lipoprotein Cholestero | TRANS | A | 0.02 | -0.1200 | 0.0086 | 2.28E-44 |
| rs264 | High-Density Lipoprotein Cholestero | TRANS | A | 0.14 | 0.0325 | 0.0033 | 6.10E-23 |
| rs268 | High-Density Lipoprotein Cholestero | TRANS | A | 0.98 | 0.2379 | 0.0076 | 5.47E-213 |
| rs13702 | High-Density Lipoprotein Cholestero | TRANS | T | 0.71 | -0.0589 | 0.0050 | 4.41E-32 |
| rs17091872 | High-Density Lipoprotein Cholestero | TRANS | A | 0.18 | -0.0559 | 0.0044 | 2.57E-36 |
| rs2410622 | High-Density Lipoprotein Cholestero | TRANS | T | 0.16 | -0.0264 | 0.0041 | 1.43E-10 |
| rs6983170 | High-Density Lipoprotein Cholestero | TRANS | T | 0.97 | 0.0933 | 0.0105 | 5.62E-19 |
| rs6651485 | High-Density Lipoprotein Cholestero | TRANS | A | 0.76 | 0.0539 | 0.0043 | 1.90E-36 |
| rs2083637 | High-Density Lipoprotein Cholestero | TRANS | A | 0.74 | -0.0484 | 0.0050 | 3.50E-22 |
| rs7837677 | High-Density Lipoprotein Cholestero | TRANS | T | 0.35 | -0.0336 | 0.0041 | 3.83E-16 |
| rs10106652 | High-Density Lipoprotein Cholestero | TRANS | A | 0.27 | 0.0306 | 0.0047 | 4.58E-11 |
| rs34859606 | High-Density Lipoprotein Cholestero | TRANS | C | 0.81 | -0.0194 | 0.0034 | 8.95E-09 |
| rs6586892 | High-Density Lipoprotein Cholestero | TRANS | A | 0.62 | 0.0360 | 0.0037 | 5.51E-22 |
| rs6983999 | High-Density Lipoprotein Cholestero | TRANS | A | 0.49 | 0.0239 | 0.0030 | 2.34E-15 |
| rs4512408 | High-Density Lipoprotein Cholestero | TRANS | T | 0.89 | 0.0255 | 0.0037 | 5.52E-12 |
| rs11774381 | High-Density Lipoprotein Cholestero | TRANS | T | 0.28 | -0.0278 | 0.0027 | 5.13E-24 |
| rs4841132 | High-Density Lipoprotein Cholestero | TRANS | A | 0.09 | -0.1080 | 0.0034 | 1.94E-215 |
| rs13292026 | High-Density Lipoprotein Cholestero | TRANS | A | 0.03 | -0.0437 | 0.0075 | 4.88E-09 |
| rs2230808 | High-Density Lipoprotein Cholestero | TRANS | T | 0.25 | -0.0236 | 0.0024 | 3.85E-23 |
| rs76881554 | High-Density Lipoprotein Cholestero | TRANS | A | 0.00 | -0.1641 | 0.0248 | 3.38E-11 |
| rs2066714 | High-Density Lipoprotein Cholestero | TRANS | T | 0.86 | -0.0514 | 0.0029 | 3.49E-69 |
| rs3905000 | High-Density Lipoprotein Cholestero | TRANS | A | 0.14 | -0.0547 | 0.0035 | 6.83E-56 |
| rs10120087 | High-Density Lipoprotein Cholestero | TRANS | A | 0.10 | 0.0444 | 0.0042 | 1.54E-26 |
| rs1800978 | High-Density Lipoprotein Cholestero | TRANS | C | 0.87 | 0.0574 | 0.0038 | 3.71E-52 |
| rs13284054 | High-Density Lipoprotein Cholestero | TRANS | T | 0.88 | 0.0541 | 0.0050 | 1.47E-27 |
| rs1800977 | High-Density Lipoprotein Cholestero | TRANS | A | 0.36 | 0.0256 | 0.0025 | 3.16E-24 |
| rs10733608 | High-Density Lipoprotein Cholestero | TRANS | T | 0.48 | 0.0139 | 0.0024 | 6.35E-09 |
| rs635634 | High-Density Lipoprotein Cholestero | TRANS | T | 0.19 | 0.0145 | 0.0025 | 6.64E-09 |
| rs581080 | High-Density Lipoprotein Cholestero | TRANS | C | 0.80 | 0.0370 | 0.0025 | 1.53E-49 |
| rs1730859 | Low-Density Lipoprotein Cholesterol | TRANS | A | 0.67 | -0.0190 | 0.0026 | 2.61E-13 |
| rs12740374 | Low-Density Lipoprotein Cholesterol | TRANS | T | 0.22 | -0.1598 | 0.0024 | 0.00E+00 |
| rs4745 | Low-Density Lipoprotein Cholesterol | TRANS | A | 0.50 | 0.0124 | 0.0020 | 5.65E-10 |
| rs2992753 | Low-Density Lipoprotein Cholesterol | TRANS | A | 0.63 | -0.0124 | 0.0021 | 3.90E-09 |
| rs867772 | Low-Density Lipoprotein Cholesterol | TRANS | A | 0.30 | -0.0258 | 0.0029 | 5.82E-19 |
| rs558971 | Low-Density Lipoprotein Cholesterol | TRANS | A | 0.47 | -0.0363 | 0.0024 | 1.19E-51 |
| rs35172831 | Low-Density Lipoprotein Cholesterol | TRANS | T | 0.55 | 0.0191 | 0.0028 | 8.12E-12 |
| rs12748152 | Low-Density Lipoprotein Cholesterol | TRANS | T | 0.07 | 0.0260 | 0.0038 | 7.62E-12 |
| rs17111483 | Low-Density Lipoprotein Cholesterol | TRANS | T | 0.89 | 0.0403 | 0.0049 | 1.91E-16 |
| rs11206510 | Low-Density Lipoprotein Cholesterol | TRANS | T | 0.83 | 0.0684 | 0.0031 | 2.68E-111 |
| rs2479409 | Low-Density Lipoprotein Cholesterol | TRANS | A | 0.65 | -0.0225 | 0.0023 | 1.06E-22 |
| rs11583680 | Low-Density Lipoprotein Cholesterol | TRANS | T | 0.13 | 0.0272 | 0.0039 | 1.86E-12 |
| rs10888896 | Low-Density Lipoprotein Cholesterol | TRANS | C | 0.74 | 0.0289 | 0.0031 | 1.03E-20 |
| rs693668 | Low-Density Lipoprotein Cholesterol | TRANS | A | 0.64 | 0.0286 | 0.0036 | 4.46E-15 |
| rs562556 | Low-Density Lipoprotein Cholesterol | TRANS | A | 0.83 | 0.1298 | 0.0079 | 4.91E-61 |
| rs61739739 | Low-Density Lipoprotein Cholesterol | TRANS | T | 0.01 | 0.0800 | 0.0113 | 1.68E-12 |
| rs1165222 | Low-Density Lipoprotein Cholesterol | TRANS | A | 0.85 | -0.1352 | 0.0080 | 1.69E-63 |
| rs1475701 | Low-Density Lipoprotein Cholesterol | TRANS | T | 0.96 | -0.0676 | 0.0064 | 1.84E-26 |
| rs7551981 | Low-Density Lipoprotein Cholesterol | TRANS | T | 0.61 | 0.0296 | 0.0028 | 7.20E-27 |
| rs10489488 | Low-Density Lipoprotein Cholesterol | TRANS | A | 0.01 | -0.0934 | 0.0111 | 5.29E-17 |
| rs12742537 | Low-Density Lipoprotein Cholesterol | TRANS | A | 0.42 | 0.0153 | 0.0020 | 2.48E-14 |
| rs10874746 | Low-Density Lipoprotein Cholesterol | TRANS | T | 0.35 | -0.0154 | 0.0021 | 2.25E-13 |
| rs2792751 | Low-Density Lipoprotein Cholesterol | TRANS | T | 0.28 | 0.0262 | 0.0022 | 1.08E-32 |
| rs1891110 | Low-Density Lipoprotein Cholesterol | TRANS | A | 0.55 | 0.0212 | 0.0020 | 3.01E-26 |
| rs7080366 | Low-Density Lipoprotein Cholesterol | TRANS | T | 0.47 | 0.0194 | 0.0025 | 8.54E-15 |
| rs41274050 | Low-Density Lipoprotein Cholesterol | TRANS | T | 0.01 | 0.0774 | 0.0112 | 4.83E-12 |
| rs2068888 | Low-Density Lipoprotein Cholesterol | TRANS | A | 0.46 | -0.0171 | 0.0020 | 1.12E-17 |
| rs2274224 | Low-Density Lipoprotein Cholesterol | TRANS | C | 0.44 | -0.0142 | 0.0024 | 2.97E-09 |

| rs11603023 | Low-Density Lipoprotein Cholesterol | TRANS | T | 0.43 | 0.0126 | 0.0020 | 3.03E-10 |
| --- | --- | --- | --- | --- | --- | --- | --- |
| rs7941030 | Low-Density Lipoprotein Cholesterol | TRANS | T | 0.62 | -0.0138 | 0.0020 | 5.69E-12 |
| rs10893500 | Low-Density Lipoprotein Cholesterol | TRANS | T | 0.86 | -0.0427 | 0.0034 | 3.98E-36 |
| rs4752805 | Low-Density Lipoprotein Cholesterol | TRANS | A | 0.75 | -0.0148 | 0.0024 | 6.98E-10 |
| rs174449 | Low-Density Lipoprotein Cholesterol | TRANS | A | 0.64 | 0.0268 | 0.0025 | 1.07E-26 |
| rs2521567 | Low-Density Lipoprotein Cholesterol | TRANS | A | 0.42 | -0.0149 | 0.0024 | 5.94E-10 |
| rs3816492 | Low-Density Lipoprotein Cholesterol | TRANS | T | 0.23 | -0.0167 | 0.0024 | 3.36E-12 |
| rs3184504 | Low-Density Lipoprotein Cholesterol | TRANS | T | 0.46 | -0.0258 | 0.0021 | 1.35E-34 |
| rs1169288 | Low-Density Lipoprotein Cholesterol | TRANS | A | 0.67 | -0.0346 | 0.0022 | 1.15E-55 |
| rs10773003 | Low-Density Lipoprotein Cholesterol | TRANS | A | 0.09 | 0.0237 | 0.0040 | 3.07E-09 |
| rs1521516 | Low-Density Lipoprotein Cholesterol | TRANS | T | 0.35 | -0.0161 | 0.0025 | 1.20E-10 |
| rs61754230 | Low-Density Lipoprotein Cholesterol | TRANS | T | 0.02 | 0.0515 | 0.0077 | 2.26E-11 |
| rs4773173 | Low-Density Lipoprotein Cholesterol | TRANS | A | 0.65 | 0.0164 | 0.0025 | 5.39E-11 |
| rs11571836 | Low-Density Lipoprotein Cholesterol | TRANS | A | 0.79 | 0.0213 | 0.0034 | 4.12E-10 |
| rs3742318 | Low-Density Lipoprotein Cholesterol | TRANS | T | 0.80 | 0.0201 | 0.0025 | 9.20E-16 |
| rs9646133 | Low-Density Lipoprotein Cholesterol | TRANS | T | 0.32 | -0.0195 | 0.0021 | 2.01E-20 |
| rs13379043 | Low-Density Lipoprotein Cholesterol | TRANS | T | 0.70 | 0.0144 | 0.0022 | 5.46E-11 |
| rs28929474 | Low-Density Lipoprotein Cholesterol | TRANS | T | 0.02 | 0.0713 | 0.0080 | 5.02E-19 |
| rs3812945 | Low-Density Lipoprotein Cholesterol | TRANS | T | 0.55 | -0.0150 | 0.0024 | 4.11E-10 |
| rs35259348 | Low-Density Lipoprotein Cholesterol | TRANS | C | 0.25 | 0.0197 | 0.0028 | 7.70E-13 |
| rs7197453 | Low-Density Lipoprotein Cholesterol | TRANS | C | 0.35 | 0.0251 | 0.0035 | 1.47E-12 |
| rs217181 | Low-Density Lipoprotein Cholesterol | TRANS | T | 0.21 | -0.0494 | 0.0034 | 1.34E-48 |
| rs9302635 | Low-Density Lipoprotein Cholesterol | TRANS | T | 0.82 | 0.0550 | 0.0041 | 1.90E-41 |
| rs28555129 | Low-Density Lipoprotein Cholesterol | TRANS | A | 0.33 | 0.0134 | 0.0022 | 1.12E-09 |
| rs6502640 | Low-Density Lipoprotein Cholesterol | TRANS | A | 0.20 | 0.0195 | 0.0033 | 3.61E-09 |
| rs704 | Low-Density Lipoprotein Cholesterol | TRANS | A | 0.48 | 0.0201 | 0.0020 | 2.22E-23 |
| rs12601110 | Low-Density Lipoprotein Cholesterol | TRANS | A | 0.92 | 0.0272 | 0.0046 | 4.91E-09 |
| rs1487971 | Low-Density Lipoprotein Cholesterol | TRANS | T | 0.39 | -0.0157 | 0.0024 | 8.28E-11 |
| rs11080150 | Low-Density Lipoprotein Cholesterol | TRANS | A | 0.69 | 0.0148 | 0.0022 | 2.33E-11 |
| rs72836561 | Low-Density Lipoprotein Cholesterol | TRANS | T | 0.03 | -0.0373 | 0.0059 | 2.51E-10 |
| rs4968318 | Low-Density Lipoprotein Cholesterol | TRANS | A | 0.61 | 0.0220 | 0.0020 | 1.69E-27 |
| rs118004742 | Low-Density Lipoprotein Cholesterol | TRANS | T | 0.95 | 0.0291 | 0.0049 | 2.24E-09 |
| rs8069974 | Low-Density Lipoprotein Cholesterol | TRANS | C | 0.36 | 0.0148 | 0.0025 | 3.40E-09 |
| rs12939848 | Low-Density Lipoprotein Cholesterol | TRANS | T | 0.38 | -0.0140 | 0.0024 | 5.64E-09 |
| rs12602912 | Low-Density Lipoprotein Cholesterol | TRANS | T | 0.21 | 0.0156 | 0.0024 | 7.20E-11 |
| rs77542162 | Low-Density Lipoprotein Cholesterol | TRANS | A | 0.98 | -0.1767 | 0.0091 | 1.91E-84 |
| rs4968839 | Low-Density Lipoprotein Cholesterol | TRANS | T | 0.69 | 0.0387 | 0.0027 | 5.75E-47 |
| rs72852601 | Low-Density Lipoprotein Cholesterol | TRANS | T | 0.02 | 0.0481 | 0.0081 | 2.54E-09 |
| rs2886232 | Low-Density Lipoprotein Cholesterol | TRANS | T | 0.11 | 0.0409 | 0.0049 | 6.60E-17 |
| rs314253 | Low-Density Lipoprotein Cholesterol | TRANS | T | 0.65 | 0.0203 | 0.0021 | 4.59E-22 |
| rs4485425 | Low-Density Lipoprotein Cholesterol | TRANS | A | 0.31 | -0.0189 | 0.0023 | 2.17E-16 |
| rs4129767 | Low-Density Lipoprotein Cholesterol | TRANS | A | 0.49 | 0.0157 | 0.0020 | 5.05E-15 |
| rs871841 | Low-Density Lipoprotein Cholesterol | TRANS | T | 0.49 | -0.0146 | 0.0020 | 2.53E-13 |
| rs77960347 | Low-Density Lipoprotein Cholesterol | TRANS | A | 0.99 | -0.0797 | 0.0093 | 1.07E-17 |
| rs7241918 | Low-Density Lipoprotein Cholesterol | TRANS | T | 0.84 | 0.0191 | 0.0028 | 8.99E-12 |
| rs1982074 | Low-Density Lipoprotein Cholesterol | TRANS | A | 0.82 | 0.0229 | 0.0026 | 3.25E-18 |
| rs892010 | Low-Density Lipoprotein Cholesterol | TRANS | C | 0.93 | 0.0395 | 0.0054 | 2.28E-13 |
| rs10417443 | Low-Density Lipoprotein Cholesterol | TRANS | C | 0.46 | 0.0325 | 0.0028 | 1.54E-31 |
| rs1122608 | Low-Density Lipoprotein Cholesterol | TRANS | T | 0.24 | -0.0479 | 0.0029 | 9.63E-63 |
| rs4300767 | Low-Density Lipoprotein Cholesterol | TRANS | A | 0.89 | -0.0947 | 0.0056 | 3.77E-63 |
| rs6511721 | Low-Density Lipoprotein Cholesterol | TRANS | A | 0.51 | -0.0371 | 0.0032 | 1.07E-31 |
| rs73015030 | Low-Density Lipoprotein Cholesterol | TRANS | A | 0.03 | -0.0788 | 0.0086 | 4.84E-20 |
| rs3745677 | Low-Density Lipoprotein Cholesterol | TRANS | A | 0.06 | 0.0774 | 0.0084 | 4.03E-20 |
| rs11669576 | Low-Density Lipoprotein Cholesterol | TRANS | A | 0.05 | 0.0636 | 0.0066 | 3.61E-22 |
| rs45508991 | Low-Density Lipoprotein Cholesterol | TRANS | T | 0.01 | 0.1064 | 0.0146 | 3.51E-13 |
| rs5927 | Low-Density Lipoprotein Cholesterol | TRANS | A | 0.25 | -0.0299 | 0.0038 | 7.92E-15 |
| rs2569538 | Low-Density Lipoprotein Cholesterol | TRANS | A | 0.09 | -0.0606 | 0.0066 | 2.09E-20 |
| rs892115 | Low-Density Lipoprotein Cholesterol | TRANS | T | 0.69 | -0.0232 | 0.0037 | 2.33E-10 |

| rs6511727 | Low-Density Lipoprotein Cholesterol | TRANS | T | 0.39 | 0.0202 | 0.0025 | 1.83E-15 |
| --- | --- | --- | --- | --- | --- | --- | --- |
| rs4804579 | Low-Density Lipoprotein Cholesterol | TRANS | T | 0.84 | 0.0244 | 0.0039 | 2.82E-10 |
| rs58542926 | Low-Density Lipoprotein Cholesterol | TRANS | T | 0.07 | -0.0978 | 0.0038 | 7.50E-146 |
| rs941408 | Low-Density Lipoprotein Cholesterol | TRANS | T | 0.29 | 0.0163 | 0.0026 | 3.66E-10 |
| rs150090162 | Low-Density Lipoprotein Cholesterol | TRANS | A | 1.00 | 0.1614 | 0.0194 | 1.02E-16 |
| rs8103315 | Low-Density Lipoprotein Cholesterol | TRANS | A | 0.12 | 0.0277 | 0.0041 | 2.02E-11 |
| rs35106910 | Low-Density Lipoprotein Cholesterol | TRANS | A | 0.95 | 0.0317 | 0.0047 | 1.31E-11 |
| rs1135062 | Low-Density Lipoprotein Cholesterol | TRANS | A | 0.70 | -0.0169 | 0.0023 | 4.48E-13 |
| rs3852856 | Low-Density Lipoprotein Cholesterol | TRANS | A | 0.20 | 0.0612 | 0.0047 | 5.90E-38 |
| rs12610605 | Low-Density Lipoprotein Cholesterol | TRANS | A | 0.16 | 0.1262 | 0.0046 | 4.86E-164 |
| rs8104483 | Low-Density Lipoprotein Cholesterol | TRANS | T | 0.71 | -0.0874 | 0.0038 | 3.37E-116 |
| rs6859 | Low-Density Lipoprotein Cholesterol | TRANS | A | 0.41 | 0.0783 | 0.0032 | 8.68E-132 |
| rs11669338 | Low-Density Lipoprotein Cholesterol | TRANS | T | 0.91 | 0.0501 | 0.0062 | 7.96E-16 |
| rs3852861 | Low-Density Lipoprotein Cholesterol | TRANS | T | 0.41 | -0.0563 | 0.0040 | 1.14E-45 |
| rs187706273 | Low-Density Lipoprotein Cholesterol | TRANS | A | 0.01 | -0.1081 | 0.0164 | 4.45E-11 |
| rs157580 | Low-Density Lipoprotein Cholesterol | TRANS | A | 0.62 | 0.0754 | 0.0033 | 2.18E-113 |
| rs157582 | Low-Density Lipoprotein Cholesterol | TRANS | T | 0.21 | -0.0324 | 0.0050 | 7.31E-11 |
| rs115881343 | Low-Density Lipoprotein Cholesterol | TRANS | T | 0.03 | 0.1945 | 0.0095 | 6.81E-94 |
| rs10119 | Low-Density Lipoprotein Cholesterol | TRANS | A | 0.28 | 0.0668 | 0.0043 | 1.93E-53 |
| rs405509 | Low-Density Lipoprotein Cholesterol | TRANS | T | 0.48 | 0.1727 | 0.0045 | 0.00E+00 |
| rs769450 | Low-Density Lipoprotein Cholesterol | TRANS | A | 0.41 | 0.0605 | 0.0047 | 2.86E-37 |
| rs769452 | Low-Density Lipoprotein Cholesterol | TRANS | T | 1.00 | -0.1493 | 0.0198 | 4.34E-14 |
| rs439401 | Low-Density Lipoprotein Cholesterol | TRANS | T | 0.37 | 0.0292 | 0.0030 | 4.57E-22 |
| rs59325138 | Low-Density Lipoprotein Cholesterol | TRANS | T | 0.40 | 0.0982 | 0.0042 | 1.03E-119 |
| rs732841 | Low-Density Lipoprotein Cholesterol | TRANS | A | 0.95 | 0.0413 | 0.0057 | 5.97E-13 |
| rs17651629 | Low-Density Lipoprotein Cholesterol | TRANS | T | 0.12 | -0.0317 | 0.0037 | 1.28E-17 |
| rs492602 | Low-Density Lipoprotein Cholesterol | TRANS | A | 0.53 | -0.0279 | 0.0021 | 4.28E-40 |
| rs641738 | Low-Density Lipoprotein Cholesterol | TRANS | T | 0.43 | 0.0141 | 0.0023 | 1.03E-09 |
| rs35350976 | Low-Density Lipoprotein Cholesterol | TRANS | A | 0.82 | -0.0168 | 0.0029 | 7.20E-09 |
| rs10185855 | Low-Density Lipoprotein Cholesterol | TRANS | A | 0.62 | 0.0138 | 0.0024 | 8.94E-09 |
| rs10490626 | Low-Density Lipoprotein Cholesterol | TRANS | A | 0.07 | -0.0447 | 0.0039 | 2.35E-30 |
| rs1808458 | Low-Density Lipoprotein Cholesterol | TRANS | T | 0.07 | 0.0310 | 0.0049 | 2.65E-10 |
| rs6706968 | Low-Density Lipoprotein Cholesterol | TRANS | A | 0.43 | -0.0228 | 0.0028 | 3.86E-16 |
| rs2198562 | Low-Density Lipoprotein Cholesterol | TRANS | C | 0.95 | 0.0353 | 0.0056 | 2.91E-10 |
| rs2287623 | Low-Density Lipoprotein Cholesterol | TRANS | A | 0.59 | -0.0181 | 0.0020 | 1.44E-19 |
| rs6435161 | Low-Density Lipoprotein Cholesterol | TRANS | T | 0.71 | 0.0236 | 0.0025 | 4.34E-21 |
| rs1473886 | Low-Density Lipoprotein Cholesterol | TRANS | T | 0.47 | -0.0146 | 0.0023 | 2.06E-10 |
| rs1048013 | Low-Density Lipoprotein Cholesterol | TRANS | T | 0.54 | 0.0123 | 0.0020 | 8.80E-10 |
| rs12710745 | Low-Density Lipoprotein Cholesterol | TRANS | A | 0.60 | 0.0200 | 0.0026 | 1.03E-14 |
| rs6547409 | Low-Density Lipoprotein Cholesterol | TRANS | T | 0.05 | -0.0953 | 0.0057 | 8.94E-62 |
| rs1801702 | Low-Density Lipoprotein Cholesterol | TRANS | C | 0.98 | 0.0969 | 0.0067 | 1.46E-47 |
| rs1042023 | Low-Density Lipoprotein Cholesterol | TRANS | C | 0.01 | 0.0864 | 0.0111 | 5.52E-15 |
| rs12713843 | Low-Density Lipoprotein Cholesterol | TRANS | T | 0.00 | -0.1881 | 0.0158 | 8.19E-33 |
| rs12713844 | Low-Density Lipoprotein Cholesterol | TRANS | C | 0.99 | 0.0741 | 0.0107 | 4.97E-12 |
| rs679899 | Low-Density Lipoprotein Cholesterol | TRANS | A | 0.47 | -0.0295 | 0.0023 | 6.98E-37 |
| rs515135 | Low-Density Lipoprotein Cholesterol | TRANS | T | 0.19 | -0.0882 | 0.0029 | 1.38E-202 |
| rs62122515 | Low-Density Lipoprotein Cholesterol | TRANS | A | 0.33 | 0.0258 | 0.0035 | 8.19E-14 |
| rs4635554 | Low-Density Lipoprotein Cholesterol | TRANS | T | 0.66 | -0.0221 | 0.0025 | 2.85E-18 |
| rs887829 | Low-Density Lipoprotein Cholesterol | TRANS | T | 0.33 | -0.0205 | 0.0021 | 1.65E-22 |
| rs1260327 | Low-Density Lipoprotein Cholesterol | TRANS | A | 0.53 | 0.0148 | 0.0022 | 2.10E-11 |
| rs814295 | Low-Density Lipoprotein Cholesterol | TRANS | A | 0.84 | 0.0305 | 0.0034 | 1.20E-19 |
| rs11556157 | Low-Density Lipoprotein Cholesterol | TRANS | A | 0.75 | -0.0202 | 0.0023 | 1.97E-18 |
| rs72796748 | Low-Density Lipoprotein Cholesterol | TRANS | T | 0.05 | 0.0588 | 0.0065 | 2.61E-19 |
| rs4077440 | Low-Density Lipoprotein Cholesterol | TRANS | T | 0.44 | 0.1167 | 0.0050 | 1.07E-122 |
| rs6718187 | Low-Density Lipoprotein Cholesterol | TRANS | A | 0.51 | -0.0697 | 0.0050 | 1.98E-43 |
| rs4148218 | Low-Density Lipoprotein Cholesterol | TRANS | A | 0.19 | -0.0598 | 0.0034 | 7.70E-70 |
| rs11125936 | Low-Density Lipoprotein Cholesterol | TRANS | T | 0.90 | 0.0245 | 0.0033 | 1.14E-13 |
| rs2143544 | Low-Density Lipoprotein Cholesterol | TRANS | T | 0.40 | -0.0165 | 0.0028 | 5.46E-09 |

| rs2745865 | Low-Density Lipoprotein Cholesterol | TRANS | T | 0.15 | 0.0387 | 0.0036 | 2.12E-26 |
| --- | --- | --- | --- | --- | --- | --- | --- |
| rs6058302 | Low-Density Lipoprotein Cholesterol | TRANS | T | 0.14 | -0.0271 | 0.0034 | 1.82E-15 |
| rs6016373 | Low-Density Lipoprotein Cholesterol | TRANS | A | 0.61 | 0.0227 | 0.0020 | 9.19E-30 |
| rs926663 | Low-Density Lipoprotein Cholesterol | TRANS | A | 0.59 | 0.0144 | 0.0025 | 8.27E-09 |
| rs6072328 | Low-Density Lipoprotein Cholesterol | TRANS | T | 0.62 | -0.0264 | 0.0027 | 1.73E-22 |
| rs6062343 | Low-Density Lipoprotein Cholesterol | TRANS | A | 0.43 | -0.0146 | 0.0020 | 2.88E-13 |
| rs2833487 | Low-Density Lipoprotein Cholesterol | TRANS | A | 0.95 | -0.0350 | 0.0056 | 4.34E-10 |
| rs2183573 | Low-Density Lipoprotein Cholesterol | TRANS | A | 0.41 | -0.0144 | 0.0023 | 4.04E-10 |
| rs138777 | Low-Density Lipoprotein Cholesterol | TRANS | A | 0.37 | 0.0125 | 0.0021 | 2.35E-09 |
| rs738409 | Low-Density Lipoprotein Cholesterol | TRANS | C | 0.77 | 0.0148 | 0.0024 | 6.76E-10 |
| rs13268 | Low-Density Lipoprotein Cholesterol | TRANS | A | 0.98 | 0.0420 | 0.0067 | 3.59E-10 |
| rs7616006 | Low-Density Lipoprotein Cholesterol | TRANS | A | 0.56 | 0.0222 | 0.0024 | 2.27E-20 |
| rs1979848 | Low-Density Lipoprotein Cholesterol | TRANS | A | 0.91 | 0.0257 | 0.0043 | 2.28E-09 |
| rs10513551 | Low-Density Lipoprotein Cholesterol | TRANS | T | 0.53 | -0.0154 | 0.0024 | 1.40E-10 |
| rs7640978 | Low-Density Lipoprotein Cholesterol | TRANS | T | 0.09 | -0.0321 | 0.0035 | 4.70E-20 |
| rs2251219 | Low-Density Lipoprotein Cholesterol | TRANS | T | 0.61 | -0.0137 | 0.0021 | 7.58E-11 |
| rs13315871 | Low-Density Lipoprotein Cholesterol | TRANS | A | 0.09 | -0.0323 | 0.0036 | 3.22E-19 |
| rs3816873 | Low-Density Lipoprotein Cholesterol | TRANS | T | 0.74 | 0.0135 | 0.0023 | 4.14E-09 |
| rs13107325 | Low-Density Lipoprotein Cholesterol | TRANS | T | 0.07 | -0.0292 | 0.0041 | 9.93E-13 |
| rs3748034 | Low-Density Lipoprotein Cholesterol | TRANS | T | 0.14 | 0.0179 | 0.0030 | 2.42E-09 |
| rs4530754 | Low-Density Lipoprotein Cholesterol | TRANS | A | 0.55 | 0.0161 | 0.0021 | 1.76E-14 |
| rs10065787 | Low-Density Lipoprotein Cholesterol | TRANS | T | 0.45 | -0.0189 | 0.0032 | 2.18E-09 |
| rs2522056 | Low-Density Lipoprotein Cholesterol | TRANS | A | 0.22 | -0.0182 | 0.0024 | 1.22E-13 |
| rs4704825 | Low-Density Lipoprotein Cholesterol | TRANS | A | 0.36 | -0.0279 | 0.0027 | 5.07E-25 |
| rs870992 | Low-Density Lipoprotein Cholesterol | TRANS | A | 0.91 | -0.0268 | 0.0042 | 1.76E-10 |
| rs10062361 | Low-Density Lipoprotein Cholesterol | TRANS | T | 0.23 | 0.0245 | 0.0033 | 1.90E-13 |
| rs3846662 | Low-Density Lipoprotein Cholesterol | TRANS | A | 0.54 | -0.0565 | 0.0023 | 2.13E-131 |
| rs11955819 | Low-Density Lipoprotein Cholesterol | TRANS | A | 0.03 | -0.0583 | 0.0081 | 4.85E-13 |
| rs17789218 | Low-Density Lipoprotein Cholesterol | TRANS | T | 0.76 | 0.0220 | 0.0027 | 4.03E-16 |
| rs9390698 | Low-Density Lipoprotein Cholesterol | TRANS | A | 0.43 | 0.0129 | 0.0020 | 1.27E-10 |
| rs3798236 | Low-Density Lipoprotein Cholesterol | TRANS | T | 0.66 | 0.0184 | 0.0025 | 1.85E-13 |
| rs9376090 | Low-Density Lipoprotein Cholesterol | TRANS | T | 0.76 | 0.0275 | 0.0023 | 5.62E-33 |
| rs1044418 | Low-Density Lipoprotein Cholesterol | TRANS | T | 0.16 | 0.0180 | 0.0028 | 1.19E-10 |
| rs12208357 | Low-Density Lipoprotein Cholesterol | TRANS | T | 0.07 | 0.0616 | 0.0040 | 2.67E-52 |
| rs34130495 | Low-Density Lipoprotein Cholesterol | TRANS | A | 0.02 | 0.0478 | 0.0065 | 2.50E-13 |
| rs62440901 | Low-Density Lipoprotein Cholesterol | TRANS | T | 0.15 | 0.0358 | 0.0038 | 1.11E-20 |
| rs3798220 | Low-Density Lipoprotein Cholesterol | TRANS | T | 0.98 | -0.1363 | 0.0080 | 1.25E-64 |
| rs10455872 | Low-Density Lipoprotein Cholesterol | TRANS | A | 0.93 | -0.0876 | 0.0057 | 4.64E-54 |
| rs12175867 | Low-Density Lipoprotein Cholesterol | TRANS | T | 0.76 | 0.0255 | 0.0033 | 1.81E-14 |
| rs1652507 | Low-Density Lipoprotein Cholesterol | TRANS | T | 0.83 | 0.0270 | 0.0030 | 1.16E-19 |
| rs2235215 | Low-Density Lipoprotein Cholesterol | TRANS | T | 0.67 | 0.0246 | 0.0028 | 1.75E-18 |
| rs1800562 | Low-Density Lipoprotein Cholesterol | TRANS | A | 0.06 | -0.0492 | 0.0044 | 6.60E-29 |
| rs129128 | Low-Density Lipoprotein Cholesterol | TRANS | T | 0.86 | 0.0208 | 0.0035 | 3.40E-09 |
| rs2249741 | Low-Density Lipoprotein Cholesterol | TRANS | A | 0.46 | -0.0203 | 0.0027 | 7.84E-14 |
| rs13192471 | Low-Density Lipoprotein Cholesterol | TRANS | T | 0.85 | -0.0336 | 0.0028 | 5.76E-33 |
| rs3800406 | Low-Density Lipoprotein Cholesterol | TRANS | A | 0.89 | 0.0278 | 0.0039 | 1.10E-12 |
| rs1129187 | Low-Density Lipoprotein Cholesterol | TRANS | T | 0.44 | -0.0123 | 0.0020 | 8.54E-10 |
| rs2239619 | Low-Density Lipoprotein Cholesterol | TRANS | A | 0.62 | 0.0159 | 0.0021 | 4.04E-14 |
| rs10263252 | Low-Density Lipoprotein Cholesterol | TRANS | A | 0.21 | -0.0230 | 0.0033 | 6.27E-12 |
| rs1997243 | Low-Density Lipoprotein Cholesterol | TRANS | A | 0.85 | -0.0164 | 0.0028 | 6.65E-09 |
| rs2282889 | Low-Density Lipoprotein Cholesterol | TRANS | A | 0.41 | -0.0164 | 0.0028 | 5.02E-09 |
| rs12670798 | Low-Density Lipoprotein Cholesterol | TRANS | T | 0.76 | -0.0295 | 0.0024 | 1.39E-34 |
| rs144787122 | Low-Density Lipoprotein Cholesterol | TRANS | A | 1.00 | -0.1042 | 0.0177 | 3.95E-09 |
| rs4722551 | Low-Density Lipoprotein Cholesterol | TRANS | T | 0.84 | -0.0386 | 0.0028 | 3.18E-42 |
| rs2391211 | Low-Density Lipoprotein Cholesterol | TRANS | T | 0.20 | 0.0231 | 0.0035 | 7.66E-11 |
| rs4302748 | Low-Density Lipoprotein Cholesterol | TRANS | A | 0.19 | 0.0152 | 0.0025 | 1.16E-09 |
| rs35803101 | Low-Density Lipoprotein Cholesterol | TRANS | A | 0.00 | -0.1387 | 0.0173 | 1.09E-15 |
| rs10260606 | Low-Density Lipoprotein Cholesterol | TRANS | C | 0.19 | 0.0369 | 0.0031 | 1.16E-32 |

| rs1014283 | Low-Density Lipoprotein Cholesterol | TRANS | A | 0.18 | -0.0186 | 0.0031 | 1.98E-09 |
| --- | --- | --- | --- | --- | --- | --- | --- |
| rs2737245 | Low-Density Lipoprotein Cholesterol | TRANS | T | 0.26 | -0.0179 | 0.0030 | 2.65E-09 |
| rs2954029 | Low-Density Lipoprotein Cholesterol | TRANS | A | 0.55 | 0.0242 | 0.0028 | 4.12E-18 |
| rs4870941 | Low-Density Lipoprotein Cholesterol | TRANS | C | 0.23 | 0.0439 | 0.0037 | 3.30E-33 |
| rs2954038 | Low-Density Lipoprotein Cholesterol | TRANS | A | 0.71 | -0.0368 | 0.0031 | 8.52E-32 |
| rs4921914 | Low-Density Lipoprotein Cholesterol | TRANS | T | 0.76 | -0.0181 | 0.0024 | 4.86E-14 |
| rs9298506 | Low-Density Lipoprotein Cholesterol | TRANS | A | 0.80 | -0.0217 | 0.0027 | 8.43E-16 |
| rs2081687 | Low-Density Lipoprotein Cholesterol | TRANS | T | 0.34 | 0.0268 | 0.0021 | 2.48E-37 |
| rs330093 | Low-Density Lipoprotein Cholesterol | TRANS | C | 0.77 | 0.0295 | 0.0032 | 1.98E-20 |
| rs11774381 | Low-Density Lipoprotein Cholesterol | TRANS | T | 0.28 | -0.0286 | 0.0028 | 1.95E-24 |
| rs11782386 | Low-Density Lipoprotein Cholesterol | TRANS | T | 0.11 | -0.0246 | 0.0034 | 8.55E-13 |
| rs3905000 | Low-Density Lipoprotein Cholesterol | TRANS | A | 0.14 | -0.0179 | 0.0029 | 6.73E-10 |
| rs635634 | Low-Density Lipoprotein Cholesterol | TRANS | T | 0.19 | 0.0750 | 0.0026 | 1.50E-182 |
| rs3812594 | Low-Density Lipoprotein Cholesterol | TRANS | A | 0.25 | -0.0139 | 0.0023 | 1.60E-09 |
| rs67710536 | Low-Density Lipoprotein Cholesterol | TRANS | A | 0.90 | -0.0267 | 0.0034 | 3.74E-15 |
| rs10757272 | Low-Density Lipoprotein Cholesterol | TRANS | T | 0.49 | -0.0195 | 0.0031 | 2.91E-10 |
| rs3780181 | Low-Density Lipoprotein Cholesterol | TRANS | A | 0.93 | 0.0328 | 0.0039 | 4.11E-17 |
| rs12740374 | Total Cholesterol | TRANS | T | 0.22 | -0.1341 | 0.0024 | 0.00E+00 |
| rs4745 | Total Cholesterol | TRANS | A | 0.50 | 0.0116 | 0.0020 | 6.64E-09 |
| rs17008917 | Total Cholesterol | TRANS | A | 0.32 | -0.0199 | 0.0029 | 6.81E-12 |
| rs558971 | Total Cholesterol | TRANS | A | 0.47 | -0.0387 | 0.0024 | 2.21E-58 |
| rs10797566 | Total Cholesterol | TRANS | T | 0.42 | 0.0165 | 0.0027 | 1.02E-09 |
| rs1077514 | Total Cholesterol | TRANS | T | 0.84 | 0.0254 | 0.0027 | 5.73E-21 |
| rs932372 | Total Cholesterol | TRANS | A | 0.54 | 0.0199 | 0.0025 | 1.61E-15 |
| rs11206508 | Total Cholesterol | TRANS | A | 0.14 | 0.0204 | 0.0034 | 1.52E-09 |
| rs11588151 | Total Cholesterol | TRANS | A | 0.80 | 0.0374 | 0.0036 | 1.24E-24 |
| rs2479409 | Total Cholesterol | TRANS | A | 0.66 | -0.0247 | 0.0022 | 1.10E-29 |
| rs10888896 | Total Cholesterol | TRANS | C | 0.74 | 0.0249 | 0.0030 | 6.14E-17 |
| rs630431 | Total Cholesterol | TRANS | A | 0.70 | 0.0306 | 0.0027 | 3.52E-29 |
| rs505151 | Total Cholesterol | TRANS | A | 0.95 | -0.0798 | 0.0049 | 1.36E-58 |
| rs4926670 | Total Cholesterol | TRANS | T | 0.10 | -0.0569 | 0.0042 | 3.74E-42 |
| rs10489488 | Total Cholesterol | TRANS | A | 0.01 | -0.0930 | 0.0110 | 3.42E-17 |
| rs1998013 | Total Cholesterol | TRANS | T | 0.01 | -0.1677 | 0.0176 | 1.31E-21 |
| rs12039115 | Total Cholesterol | TRANS | T | 0.12 | -0.0685 | 0.0037 | 2.00E-78 |
| rs11207993 | Total Cholesterol | TRANS | T | 0.17 | -0.0323 | 0.0043 | 7.95E-14 |
| rs12130333 | Total Cholesterol | TRANS | T | 0.19 | -0.0455 | 0.0033 | 8.33E-44 |
| rs2391199 | Total Cholesterol | TRANS | T | 0.10 | -0.0232 | 0.0033 | 2.07E-12 |
| rs2862954 | Total Cholesterol | TRANS | T | 0.56 | -0.0138 | 0.0020 | 6.23E-12 |
| rs2792751 | Total Cholesterol | TRANS | T | 0.28 | 0.0239 | 0.0022 | 2.17E-27 |
| rs12572599 | Total Cholesterol | TRANS | T | 0.78 | 0.0183 | 0.0028 | 7.01E-11 |
| rs3736946 | Total Cholesterol | TRANS | A | 0.89 | 0.0246 | 0.0031 | 2.76E-15 |
| rs11595267 | Total Cholesterol | TRANS | T | 0.73 | -0.0177 | 0.0030 | 3.50E-09 |
| rs7903137 | Total Cholesterol | TRANS | T | 0.43 | 0.0259 | 0.0027 | 8.71E-22 |
| rs970548 | Total Cholesterol | TRANS | A | 0.76 | -0.0179 | 0.0023 | 8.14E-15 |
| rs17134533 | Total Cholesterol | TRANS | A | 0.14 | -0.0202 | 0.0032 | 2.75E-10 |
| rs41274050 | Total Cholesterol | TRANS | T | 0.01 | 0.0930 | 0.0110 | 2.75E-17 |
| rs2068888 | Total Cholesterol | TRANS | A | 0.46 | -0.0210 | 0.0020 | 6.82E-26 |
| rs2274224 | Total Cholesterol | TRANS | C | 0.44 | -0.0171 | 0.0024 | 9.06E-13 |
| rs10466533 | Total Cholesterol | TRANS | A | 0.92 | -0.0400 | 0.0063 | 2.53E-10 |
| rs6589566 | Total Cholesterol | TRANS | A | 0.92 | -0.0856 | 0.0049 | 2.23E-67 |
| rs3135506 | Total Cholesterol | TRANS | C | 0.06 | 0.0778 | 0.0054 | 4.75E-47 |
| rs5104 | Total Cholesterol | TRANS | T | 0.86 | -0.0283 | 0.0032 | 5.82E-19 |
| rs12270837 | Total Cholesterol | TRANS | A | 0.94 | 0.0402 | 0.0049 | 3.14E-16 |
| rs11603023 | Total Cholesterol | TRANS | T | 0.43 | 0.0135 | 0.0020 | 1.29E-11 |
| rs10750215 | Total Cholesterol | TRANS | T | 0.39 | 0.0240 | 0.0024 | 1.45E-23 |
| rs7952602 | Total Cholesterol | TRANS | C | 0.14 | 0.0356 | 0.0034 | 1.08E-25 |
| rs16928809 | Total Cholesterol | TRANS | A | 0.09 | -0.0211 | 0.0035 | 1.66E-09 |
| rs4752805 | Total Cholesterol | TRANS | A | 0.75 | -0.0239 | 0.0023 | 2.74E-25 |

| rs2521567 | Total Cholesterol | TRANS | A | 0.42 | -0.0167 | 0.0024 | 3.45E-12 |
| --- | --- | --- | --- | --- | --- | --- | --- |
| rs2305535 | Total Cholesterol | TRANS | A | 0.24 | -0.0155 | 0.0023 | 1.68E-11 |
| rs499974 | Total Cholesterol | TRANS | A | 0.18 | -0.0154 | 0.0026 | 3.22E-09 |
| rs4134379 | Total Cholesterol | TRANS | A | 0.83 | 0.0267 | 0.0036 | 1.19E-13 |
| rs7298565 | Total Cholesterol | TRANS | A | 0.53 | 0.0161 | 0.0020 | 6.94E-16 |
| rs3184504 | Total Cholesterol | TRANS | T | 0.46 | -0.0291 | 0.0020 | 4.45E-48 |
| rs1169288 | Total Cholesterol | TRANS | A | 0.67 | -0.0359 | 0.0021 | 2.09E-65 |
| rs10773003 | Total Cholesterol | TRANS | A | 0.09 | 0.0369 | 0.0040 | 2.63E-20 |
| rs838880 | Total Cholesterol | TRANS | T | 0.65 | -0.0144 | 0.0021 | 7.49E-12 |
| rs10846744 | Total Cholesterol | TRANS | C | 0.16 | 0.0201 | 0.0034 | 3.47E-09 |
| rs12320328 | Total Cholesterol | TRANS | A | 0.91 | 0.0269 | 0.0045 | 2.27E-09 |
| rs1521516 | Total Cholesterol | TRANS | T | 0.36 | -0.0161 | 0.0025 | 1.26E-10 |
| rs7305599 | Total Cholesterol | TRANS | A | 0.47 | 0.0113 | 0.0019 | 2.62E-09 |
| rs4773173 | Total Cholesterol | TRANS | A | 0.65 | 0.0158 | 0.0024 | 4.61E-11 |
| rs17532301 | Total Cholesterol | TRANS | A | 0.07 | -0.0294 | 0.0046 | 1.65E-10 |
| rs8017377 | Total Cholesterol | TRANS | A | 0.44 | 0.0187 | 0.0020 | 8.82E-21 |
| rs7157785 | Total Cholesterol | TRANS | T | 0.17 | 0.0175 | 0.0027 | 9.47E-11 |
| rs9646133 | Total Cholesterol | TRANS | T | 0.32 | -0.0149 | 0.0021 | 1.35E-12 |
| rs28929474 | Total Cholesterol | TRANS | T | 0.02 | 0.0688 | 0.0078 | 1.15E-18 |
| rs4622454 | Total Cholesterol | TRANS | T | 0.36 | 0.0153 | 0.0026 | 2.39E-09 |
| rs28594460 | Total Cholesterol | TRANS | A | 0.84 | -0.0597 | 0.0037 | 3.16E-57 |
| rs11855284 | Total Cholesterol | TRANS | T | 0.82 | -0.0410 | 0.0037 | 1.40E-28 |
| rs13329672 | Total Cholesterol | TRANS | T | 0.26 | 0.0246 | 0.0040 | 5.47E-10 |
| rs12912415 | Total Cholesterol | TRANS | A | 0.85 | 0.0249 | 0.0038 | 7.28E-11 |
| rs17765311 | Total Cholesterol | TRANS | A | 0.62 | -0.0174 | 0.0025 | 3.19E-12 |
| rs1127796 | Total Cholesterol | TRANS | T | 0.47 | -0.0144 | 0.0023 | 4.86E-10 |
| rs2469202 | Total Cholesterol | TRANS | A | 0.81 | 0.0148 | 0.0025 | 3.37E-09 |
| rs9938550 | Total Cholesterol | TRANS | A | 0.39 | 0.0139 | 0.0021 | 3.62E-11 |
| rs72786786 | Total Cholesterol | TRANS | A | 0.30 | 0.0415 | 0.0030 | 3.42E-43 |
| rs7203984 | Total Cholesterol | TRANS | A | 0.79 | 0.0377 | 0.0032 | 2.77E-32 |
| rs289719 | Total Cholesterol | TRANS | T | 0.31 | 0.0182 | 0.0027 | 1.08E-11 |
| rs8060686 | Total Cholesterol | TRANS | T | 0.81 | -0.0210 | 0.0026 | 7.07E-16 |
| rs35259348 | Total Cholesterol | TRANS | C | 0.25 | 0.0169 | 0.0028 | 7.36E-10 |
| rs7197453 | Total Cholesterol | TRANS | C | 0.35 | 0.0244 | 0.0036 | 6.85E-12 |
| rs217181 | Total Cholesterol | TRANS | T | 0.21 | -0.0507 | 0.0033 | 5.36E-54 |
| rs9302635 | Total Cholesterol | TRANS | T | 0.82 | 0.0543 | 0.0041 | 2.15E-40 |
| rs6502640 | Total Cholesterol | TRANS | A | 0.20 | 0.0210 | 0.0033 | 2.14E-10 |
| rs170041 | Total Cholesterol | TRANS | T | 0.27 | 0.0167 | 0.0026 | 1.28E-10 |
| rs704 | Total Cholesterol | TRANS | A | 0.48 | 0.0118 | 0.0020 | 5.21E-09 |
| rs16963468 | Total Cholesterol | TRANS | T | 0.08 | -0.0285 | 0.0042 | 2.04E-11 |
| rs1487971 | Total Cholesterol | TRANS | T | 0.39 | -0.0178 | 0.0024 | 1.39E-13 |
| rs2517957 | Total Cholesterol | TRANS | A | 0.66 | 0.0174 | 0.0024 | 4.73E-13 |
| rs3809868 | Total Cholesterol | TRANS | A | 0.53 | -0.0170 | 0.0023 | 1.30E-13 |
| rs12939848 | Total Cholesterol | TRANS | T | 0.38 | -0.0146 | 0.0024 | 1.19E-09 |
| rs12602912 | Total Cholesterol | TRANS | T | 0.21 | 0.0171 | 0.0024 | 1.15E-12 |
| rs77542162 | Total Cholesterol | TRANS | A | 0.98 | -0.1364 | 0.0089 | 1.80E-52 |
| rs4968839 | Total Cholesterol | TRANS | T | 0.69 | 0.0303 | 0.0026 | 2.38E-31 |
| rs2886232 | Total Cholesterol | TRANS | T | 0.11 | 0.0321 | 0.0048 | 3.07E-11 |
| rs314253 | Total Cholesterol | TRANS | T | 0.65 | 0.0176 | 0.0020 | 3.22E-18 |
| rs17203281 | Total Cholesterol | TRANS | A | 0.32 | -0.0181 | 0.0030 | 2.22E-09 |
| rs9901673 | Total Cholesterol | TRANS | A | 0.16 | -0.0178 | 0.0026 | 8.67E-12 |
| rs4129767 | Total Cholesterol | TRANS | A | 0.49 | 0.0173 | 0.0019 | 7.74E-20 |
| rs871841 | Total Cholesterol | TRANS | T | 0.49 | -0.0150 | 0.0020 | 6.93E-14 |
| rs77960347 | Total Cholesterol | TRANS | A | 0.99 | -0.1804 | 0.0091 | 3.09E-87 |
| rs117623631 | Total Cholesterol | TRANS | T | 0.00 | 0.2665 | 0.0251 | 3.00E-26 |
| rs9955201 | Total Cholesterol | TRANS | A | 0.05 | 0.0426 | 0.0060 | 1.64E-12 |
| rs7240405 | Total Cholesterol | TRANS | A | 0.16 | -0.0387 | 0.0026 | 1.90E-48 |
| rs11660468 | Total Cholesterol | TRANS | T | 0.40 | 0.0187 | 0.0025 | 2.04E-14 |

| rs12968116 | Total Cholesterol | TRANS | T | 0.11 | 0.0183 | 0.0031 | 3.40E-09 |
| --- | --- | --- | --- | --- | --- | --- | --- |
| rs3786725 | Total Cholesterol | TRANS | A | 0.29 | -0.0248 | 0.0038 | 4.02E-11 |
| rs73015007 | Total Cholesterol | TRANS | A | 0.24 | 0.0512 | 0.0051 | 6.69E-24 |
| rs61194703 | Total Cholesterol | TRANS | A | 0.88 | 0.1693 | 0.0051 | 5.00E-240 |
| rs12710260 | Total Cholesterol | TRANS | C | 0.46 | 0.0301 | 0.0031 | 1.35E-22 |
| rs11669576 | Total Cholesterol | TRANS | A | 0.05 | 0.0773 | 0.0050 | 5.03E-53 |
| rs45508991 | Total Cholesterol | TRANS | T | 0.01 | 0.0833 | 0.0142 | 4.18E-09 |
| rs5927 | Total Cholesterol | TRANS | A | 0.25 | -0.0456 | 0.0034 | 6.06E-41 |
| rs440677 | Total Cholesterol | TRANS | A | 0.60 | -0.0134 | 0.0021 | 1.83E-10 |
| rs737337 | Total Cholesterol | TRANS | T | 0.90 | 0.0297 | 0.0035 | 3.86E-17 |
| rs7252293 | Total Cholesterol | TRANS | T | 0.17 | -0.0294 | 0.0039 | 5.13E-14 |
| rs157582 | Total Cholesterol | TRANS | T | 0.21 | 0.0689 | 0.0043 | 5.37E-58 |
| rs8106922 | Total Cholesterol | TRANS | A | 0.59 | -0.0390 | 0.0046 | 1.12E-17 |
| rs115881343 | Total Cholesterol | TRANS | T | 0.02 | 0.1384 | 0.0091 | 2.26E-52 |
| rs1160985 | Total Cholesterol | TRANS | T | 0.45 | -0.1167 | 0.0069 | 1.54E-63 |
| rs405509 | Total Cholesterol | TRANS | T | 0.48 | 0.0622 | 0.0047 | 5.60E-40 |
| rs769450 | Total Cholesterol | TRANS | A | 0.41 | 0.1239 | 0.0072 | 3.36E-67 |
| rs439401 | Total Cholesterol | TRANS | T | 0.37 | -0.0270 | 0.0023 | 7.70E-31 |
| rs59325138 | Total Cholesterol | TRANS | T | 0.40 | 0.0259 | 0.0039 | 3.37E-11 |
| rs8111069 | Total Cholesterol | TRANS | A | 0.67 | -0.0173 | 0.0028 | 8.45E-10 |
| rs732841 | Total Cholesterol | TRANS | A | 0.95 | 0.0403 | 0.0056 | 7.83E-13 |
| rs17651629 | Total Cholesterol | TRANS | T | 0.12 | -0.0311 | 0.0036 | 7.93E-18 |
| rs4802307 | Total Cholesterol | TRANS | T | 0.30 | -0.0158 | 0.0025 | 2.85E-10 |
| rs28362459 | Total Cholesterol | TRANS | A | 0.89 | -0.0226 | 0.0038 | 2.62E-09 |
| rs10490626 | Total Cholesterol | TRANS | A | 0.07 | -0.0410 | 0.0038 | 4.23E-27 |
| rs17050272 | Total Cholesterol | TRANS | A | 0.43 | -0.0198 | 0.0027 | 2.50E-13 |
| rs16831235 | Total Cholesterol | TRANS | A | 0.15 | 0.0161 | 0.0027 | 2.48E-09 |
| rs13019116 | Total Cholesterol | TRANS | A | 0.41 | -0.0188 | 0.0027 | 3.81E-12 |
| rs2111485 | Total Cholesterol | TRANS | A | 0.40 | 0.0141 | 0.0024 | 4.11E-09 |
| rs2287623 | Total Cholesterol | TRANS | A | 0.59 | -0.0191 | 0.0020 | 1.51E-21 |
| rs1035140 | Total Cholesterol | TRANS | A | 0.54 | 0.0138 | 0.0023 | 1.87E-09 |
| rs7589059 | Total Cholesterol | TRANS | T | 0.73 | -0.0165 | 0.0028 | 2.50E-09 |
| rs6435161 | Total Cholesterol | TRANS | T | 0.71 | 0.0277 | 0.0025 | 1.75E-29 |
| rs1473886 | Total Cholesterol | TRANS | T | 0.47 | -0.0243 | 0.0023 | 4.24E-26 |
| rs7585590 | Total Cholesterol | TRANS | T | 0.39 | -0.0151 | 0.0024 | 3.57E-10 |
| rs6547409 | Total Cholesterol | TRANS | T | 0.05 | -0.0832 | 0.0061 | 5.40E-43 |
| rs10184054 | Total Cholesterol | TRANS | C | 0.76 | -0.0359 | 0.0046 | 9.86E-15 |
| rs1042034 | Total Cholesterol | TRANS | T | 0.76 | 0.0512 | 0.0037 | 8.28E-43 |
| rs1801702 | Total Cholesterol | TRANS | C | 0.98 | 0.0941 | 0.0064 | 3.03E-48 |
| rs12713844 | Total Cholesterol | TRANS | C | 0.99 | 0.0616 | 0.0105 | 3.95E-09 |
| rs1800478 | Total Cholesterol | TRANS | A | 0.01 | -0.1220 | 0.0175 | 3.57E-12 |
| rs512535 | Total Cholesterol | TRANS | T | 0.49 | 0.0289 | 0.0050 | 9.47E-09 |
| rs515135 | Total Cholesterol | TRANS | T | 0.19 | -0.0803 | 0.0035 | 1.28E-113 |
| rs1367113 | Total Cholesterol | TRANS | A | 0.33 | 0.0692 | 0.0049 | 1.11E-45 |
| rs1429972 | Total Cholesterol | TRANS | T | 0.47 | -0.0486 | 0.0052 | 4.25E-21 |
| rs887829 | Total Cholesterol | TRANS | T | 0.33 | -0.0197 | 0.0021 | 2.07E-20 |
| rs11563251 | Total Cholesterol | TRANS | T | 0.11 | 0.0247 | 0.0039 | 1.45E-10 |
| rs6717803 | Total Cholesterol | TRANS | A | 0.95 | 0.0594 | 0.0063 | 4.54E-21 |
| rs780090 | Total Cholesterol | TRANS | T | 0.09 | 0.0329 | 0.0050 | 3.98E-11 |
| rs814295 | Total Cholesterol | TRANS | A | 0.84 | 0.0534 | 0.0033 | 1.54E-58 |
| rs7604798 | Total Cholesterol | TRANS | T | 0.51 | -0.0346 | 0.0030 | 2.29E-30 |
| rs12104449 | Total Cholesterol | TRANS | A | 0.88 | -0.0255 | 0.0035 | 4.84E-13 |
| rs6547829 | Total Cholesterol | TRANS | T | 0.09 | 0.0249 | 0.0036 | 4.24E-12 |
| rs4850047 | Total Cholesterol | TRANS | T | 0.15 | 0.0200 | 0.0033 | 1.36E-09 |
| rs10208987 | Total Cholesterol | TRANS | T | 0.92 | 0.0417 | 0.0044 | 2.91E-21 |
| rs6752551 | Total Cholesterol | TRANS | A | 0.45 | -0.0330 | 0.0027 | 1.11E-33 |
| rs4148218 | Total Cholesterol | TRANS | A | 0.19 | -0.0522 | 0.0033 | 5.20E-57 |
| rs11125936 | Total Cholesterol | TRANS | T | 0.90 | 0.0214 | 0.0033 | 9.18E-11 |

| rs847812 | Total Cholesterol | TRANS | C | 0.40 | -0.0147 | 0.0024 | 1.02E-09 |
| --- | --- | --- | --- | --- | --- | --- | --- |
| rs14234 | Total Cholesterol | TRANS | A | 0.58 | -0.0125 | 0.0020 | 3.85E-10 |
| rs2745865 | Total Cholesterol | TRANS | T | 0.15 | 0.0242 | 0.0035 | 4.72E-12 |
| rs11700250 | Total Cholesterol | TRANS | T | 0.15 | -0.0334 | 0.0037 | 1.88E-19 |
| rs4142393 | Total Cholesterol | TRANS | T | 0.48 | 0.0172 | 0.0024 | 8.59E-13 |
| rs2183573 | Total Cholesterol | TRANS | A | 0.41 | -0.0165 | 0.0023 | 7.31E-13 |
| rs2298428 | Total Cholesterol | TRANS | T | 0.20 | -0.0229 | 0.0025 | 5.22E-20 |
| rs4253772 | Total Cholesterol | TRANS | T | 0.10 | 0.0205 | 0.0033 | 5.23E-10 |
| rs1961957 | Total Cholesterol | TRANS | A | 0.40 | -0.0163 | 0.0027 | 1.71E-09 |
| rs7616006 | Total Cholesterol | TRANS | A | 0.56 | 0.0238 | 0.0024 | 7.28E-24 |
| rs11708067 | Total Cholesterol | TRANS | A | 0.78 | 0.0151 | 0.0024 | 2.81E-10 |
| rs2290159 | Total Cholesterol | TRANS | C | 0.21 | -0.0157 | 0.0027 | 3.77E-09 |
| rs1979848 | Total Cholesterol | TRANS | A | 0.91 | 0.0251 | 0.0042 | 2.21E-09 |
| rs4683438 | Total Cholesterol | TRANS | T | 0.35 | -0.0199 | 0.0025 | 1.73E-15 |
| rs4680585 | Total Cholesterol | TRANS | A | 0.47 | 0.0191 | 0.0026 | 2.05E-13 |
| rs7640978 | Total Cholesterol | TRANS | T | 0.09 | -0.0314 | 0.0034 | 2.59E-20 |
| rs13315871 | Total Cholesterol | TRANS | A | 0.09 | -0.0292 | 0.0035 | 7.28E-17 |
| rs2035816 | Total Cholesterol | TRANS | A | 0.92 | 0.0250 | 0.0043 | 6.47E-09 |
| rs13107325 | Total Cholesterol | TRANS | T | 0.07 | -0.0361 | 0.0040 | 1.94E-19 |
| rs6054 | Total Cholesterol | TRANS | T | 0.00 | 0.1303 | 0.0176 | 1.33E-13 |
| rs16844364 | Total Cholesterol | TRANS | A | 0.22 | 0.0255 | 0.0030 | 2.31E-17 |
| rs16844401 | Total Cholesterol | TRANS | A | 0.07 | 0.0240 | 0.0040 | 1.93E-09 |
| rs2603188 | Total Cholesterol | TRANS | C | 0.86 | 0.0184 | 0.0029 | 2.48E-10 |
| rs293429 | Total Cholesterol | TRANS | T | 0.70 | -0.0175 | 0.0022 | 2.31E-15 |
| rs61361928 | Total Cholesterol | TRANS | T | 1.00 | 0.1057 | 0.0148 | 9.12E-13 |
| rs35844368 | Total Cholesterol | TRANS | T | 0.22 | 0.0252 | 0.0032 | 3.43E-15 |
| rs4530754 | Total Cholesterol | TRANS | A | 0.55 | 0.0151 | 0.0020 | 4.37E-14 |
| rs4704820 | Total Cholesterol | TRANS | T | 0.81 | 0.0191 | 0.0033 | 5.65E-09 |
| rs6882076 | Total Cholesterol | TRANS | T | 0.37 | -0.0440 | 0.0020 | 3.61E-102 |
| rs870992 | Total Cholesterol | TRANS | A | 0.91 | -0.0252 | 0.0042 | 1.98E-09 |
| rs7703051 | Total Cholesterol | TRANS | A | 0.39 | 0.0599 | 0.0020 | 8.78E-197 |
| rs202046887 | Total Cholesterol | TRANS | T | 0.00 | -0.5371 | 0.0354 | 5.86E-52 |
| rs1881116 | Total Cholesterol | TRANS | T | 0.30 | -0.0194 | 0.0030 | 8.42E-11 |
| rs1997243 | Total Cholesterol | TRANS | A | 0.85 | -0.0294 | 0.0029 | 2.03E-24 |
| rs10282707 | Total Cholesterol | TRANS | T | 0.42 | -0.0132 | 0.0022 | 1.85E-09 |
| rs2282894 | Total Cholesterol | TRANS | T | 0.58 | 0.0172 | 0.0027 | 1.87E-10 |
| rs12670798 | Total Cholesterol | TRANS | T | 0.76 | -0.0294 | 0.0024 | 2.19E-34 |
| rs144787122 | Total Cholesterol | TRANS | A | 1.00 | -0.1223 | 0.0173 | 1.59E-12 |
| rs4722551 | Total Cholesterol | TRANS | T | 0.84 | -0.0230 | 0.0027 | 1.81E-17 |
| rs35803101 | Total Cholesterol | TRANS | A | 0.00 | -0.1288 | 0.0169 | 2.75E-14 |
| rs10260606 | Total Cholesterol | TRANS | C | 0.19 | 0.0344 | 0.0031 | 1.41E-28 |
| rs217378 | Total Cholesterol | TRANS | A | 0.43 | 0.0142 | 0.0023 | 7.55E-10 |
| rs3812316 | Total Cholesterol | TRANS | C | 0.88 | 0.0191 | 0.0030 | 1.93E-10 |
| rs2721965 | Total Cholesterol | TRANS | A | 0.68 | 0.0209 | 0.0028 | 9.09E-14 |
| rs17730649 | Total Cholesterol | TRANS | A | 0.41 | 0.0258 | 0.0027 | 2.56E-21 |
| rs2980876 | Total Cholesterol | TRANS | T | 0.68 | -0.0892 | 0.0036 | 4.12E-135 |
| rs7846135 | Total Cholesterol | TRANS | A | 0.26 | 0.0488 | 0.0036 | 5.20E-43 |
| rs75998532 | Total Cholesterol | TRANS | T | 0.97 | 0.0774 | 0.0080 | 3.87E-22 |
| rs6557781 | Total Cholesterol | TRANS | T | 0.15 | -0.0201 | 0.0033 | 1.12E-09 |
| rs9298506 | Total Cholesterol | TRANS | A | 0.80 | -0.0228 | 0.0027 | 3.55E-17 |
| rs2081687 | Total Cholesterol | TRANS | T | 0.34 | 0.0333 | 0.0021 | 1.15E-56 |
| rs2875967 | Total Cholesterol | TRANS | A | 0.42 | 0.0163 | 0.0021 | 9.26E-15 |
| rs7013120 | Total Cholesterol | TRANS | A | 0.28 | -0.0146 | 0.0025 | 5.23E-09 |
| rs7002551 | Total Cholesterol | TRANS | T | 0.77 | 0.0310 | 0.0030 | 7.57E-25 |
| rs11774381 | Total Cholesterol | TRANS | T | 0.28 | -0.0309 | 0.0029 | 3.15E-27 |
| rs78933755 | Total Cholesterol | TRANS | A | 0.89 | 0.0330 | 0.0044 | 9.97E-14 |
| rs2230808 | Total Cholesterol | TRANS | T | 0.25 | -0.0191 | 0.0023 | 1.26E-16 |
| rs4149311 | Total Cholesterol | TRANS | T | 0.12 | 0.0449 | 0.0036 | 3.08E-35 |

| rs11790326 | Total Cholesterol | TRANS | T | 0.79 | 0.0420 | 0.0035 | 2.35E-33 |
| --- | --- | --- | --- | --- | --- | --- | --- |
| rs11789603 | Total Cholesterol | TRANS | T | 0.10 | 0.0706 | 0.0049 | 9.40E-47 |
| rs34171271 | Total Cholesterol | TRANS | T | 0.89 | 0.0473 | 0.0045 | 4.50E-26 |
| rs1800977 | Total Cholesterol | TRANS | A | 0.36 | 0.0173 | 0.0025 | 5.92E-12 |
| rs7866925 | Total Cholesterol | TRANS | T | 0.24 | 0.0177 | 0.0028 | 2.87E-10 |
| rs635634 | Total Cholesterol | TRANS | T | 0.19 | 0.0710 | 0.0025 | 3.57E-177 |
| rs581080 | Total Cholesterol | TRANS | C | 0.80 | 0.0182 | 0.0025 | 3.56E-13 |
| rs3927680 | Total Cholesterol | TRANS | A | 0.55 | -0.0120 | 0.0020 | 2.02E-09 |
| rs16937390 | Total Cholesterol | TRANS | T | 0.92 | -0.0382 | 0.0058 | 4.37E-11 |
| rs7859362 | Total Cholesterol | TRANS | T | 0.48 | 0.0162 | 0.0026 | 5.25E-10 |
| rs3780181 | Total Cholesterol | TRANS | A | 0.93 | 0.0293 | 0.0038 | 1.26E-14 |
| rs12740374 | Triglyceride | TRANS | T | 0.22 | -0.0153 | 0.0024 | 1.83E-10 |
| rs12043350 | Triglyceride | TRANS | T | 0.31 | 0.0153 | 0.0025 | 9.37E-10 |
| rs1011731 | Triglyceride | TRANS | A | 0.57 | -0.0132 | 0.0020 | 4.12E-11 |
| rs78444298 | Triglyceride | TRANS | A | 0.02 | -0.0522 | 0.0088 | 3.00E-09 |
| rs2821231 | Triglyceride | TRANS | T | 0.48 | 0.0159 | 0.0027 | 3.90E-09 |
| rs765751 | Triglyceride | TRANS | T | 0.37 | -0.0195 | 0.0023 | 2.30E-17 |
| rs10489615 | Triglyceride | TRANS | A | 0.42 | 0.0392 | 0.0020 | 3.17E-85 |
| rs2273967 | Triglyceride | TRANS | T | 0.24 | -0.0175 | 0.0027 | 8.84E-11 |
| rs1077514 | Triglyceride | TRANS | T | 0.84 | 0.0206 | 0.0027 | 2.36E-14 |
| rs16826069 | Triglyceride | TRANS | A | 0.79 | -0.0237 | 0.0024 | 5.39E-23 |
| rs2055491 | Triglyceride | TRANS | T | 0.51 | -0.0118 | 0.0020 | 3.64E-09 |
| rs10889353 | Triglyceride | TRANS | A | 0.67 | 0.0752 | 0.0021 | 2.01E-280 |
| rs2613503 | Triglyceride | TRANS | A | 0.82 | 0.0202 | 0.0030 | 1.49E-11 |
| rs2792751 | Triglyceride | TRANS | T | 0.28 | -0.0177 | 0.0022 | 7.50E-16 |
| rs11195943 | Triglyceride | TRANS | T | 0.10 | -0.0229 | 0.0036 | 2.05E-10 |
| rs10886863 | Triglyceride | TRANS | T | 0.03 | -0.0582 | 0.0099 | 4.26E-09 |
| rs41274050 | Triglyceride | TRANS | T | 0.01 | 0.0939 | 0.0111 | 2.70E-17 |
| rs17134592 | Triglyceride | TRANS | C | 0.86 | 0.0206 | 0.0033 | 4.32E-10 |
| rs7923609 | Triglyceride | TRANS | A | 0.52 | 0.0296 | 0.0031 | 1.20E-21 |
| rs2298117 | Triglyceride | TRANS | T | 0.46 | 0.0122 | 0.0020 | 1.02E-09 |
| rs7901016 | Triglyceride | TRANS | T | 0.94 | -0.0396 | 0.0043 | 3.55E-20 |
| rs10748579 | Triglyceride | TRANS | A | 0.42 | -0.0178 | 0.0020 | 1.92E-18 |
| rs7081888 | Triglyceride | TRANS | T | 0.92 | -0.0329 | 0.0049 | 1.69E-11 |
| rs2068888 | Triglyceride | TRANS | A | 0.46 | -0.0368 | 0.0021 | 5.79E-71 |
| rs7940646 | Triglyceride | TRANS | T | 0.29 | -0.0152 | 0.0022 | 4.41E-12 |
| rs4938289 | Triglyceride | TRANS | T | 0.06 | 0.0475 | 0.0062 | 1.29E-14 |
| rs12799766 | Triglyceride | TRANS | A | 0.22 | 0.0420 | 0.0047 | 3.70E-19 |
| rs74360954 | Triglyceride | TRANS | T | 0.05 | -0.1130 | 0.0082 | 1.52E-43 |
| rs2000571 | Triglyceride | TRANS | A | 0.21 | 0.0633 | 0.0059 | 1.51E-26 |
| rs180357 | Triglyceride | TRANS | T | 0.70 | -0.0493 | 0.0049 | 4.16E-24 |
| rs4938307 | Triglyceride | TRANS | A | 0.12 | -0.0732 | 0.0067 | 4.66E-28 |
| rs61730763 | Triglyceride | TRANS | A | 0.00 | 0.1900 | 0.0232 | 2.85E-16 |
| rs17120029 | Triglyceride | TRANS | T | 0.07 | 0.1421 | 0.0090 | 1.20E-56 |
| rs11604424 | Triglyceride | TRANS | T | 0.78 | -0.0662 | 0.0061 | 1.52E-27 |
| rs619054 | Triglyceride | TRANS | A | 0.24 | -0.0567 | 0.0045 | 1.14E-36 |
| rs143292359 | Triglyceride | TRANS | A | 0.00 | 0.2415 | 0.0399 | 1.46E-09 |
| rs662799 | Triglyceride | TRANS | A | 0.93 | -0.1059 | 0.0076 | 1.18E-43 |
| rs9804646 | Triglyceride | TRANS | T | 0.09 | -0.0598 | 0.0060 | 2.84E-23 |
| rs5104 | Triglyceride | TRANS | T | 0.86 | -0.0354 | 0.0042 | 1.61E-17 |
| rs11216157 | Triglyceride | TRANS | A | 0.87 | 0.0549 | 0.0066 | 1.24E-16 |
| rs888246 | Triglyceride | TRANS | T | 0.10 | 0.0711 | 0.0064 | 9.50E-29 |
| rs2075292 | Triglyceride | TRANS | T | 0.87 | -0.0627 | 0.0047 | 8.39E-40 |
| rs11216168 | Triglyceride | TRANS | A | 0.14 | -0.0694 | 0.0064 | 5.32E-27 |
| rs2000615 | Triglyceride | TRANS | T | 0.88 | 0.0572 | 0.0061 | 3.97E-21 |
| rs490262 | Triglyceride | TRANS | A | 0.20 | -0.0186 | 0.0025 | 2.24E-13 |
| rs10832027 | Triglyceride | TRANS | A | 0.69 | 0.0161 | 0.0024 | 1.76E-11 |
| rs546383 | Triglyceride | TRANS | T | 0.41 | -0.0133 | 0.0023 | 7.57E-09 |

| rs925946 | Triglyceride | TRANS | T | 0.30 | 0.0164 | 0.0022 | 7.62E-14 |
| --- | --- | --- | --- | --- | --- | --- | --- |
| rs326214 | Triglyceride | TRANS | A | 0.66 | 0.0222 | 0.0021 | 4.09E-26 |
| rs2727271 | Triglyceride | TRANS | A | 0.87 | -0.0646 | 0.0034 | 1.84E-78 |
| rs174587 | Triglyceride | TRANS | T | 0.21 | 0.0481 | 0.0032 | 6.87E-50 |
| rs35169799 | Triglyceride | TRANS | T | 0.06 | 0.0415 | 0.0041 | 4.77E-24 |
| rs4014195 | Triglyceride | TRANS | C | 0.65 | -0.0147 | 0.0021 | 2.62E-12 |
| rs2229738 | Triglyceride | TRANS | T | 0.08 | 0.0243 | 0.0038 | 1.63E-10 |
| rs11237471 | Triglyceride | TRANS | T | 0.17 | -0.0217 | 0.0034 | 1.67E-10 |
| rs2075260 | Triglyceride | TRANS | A | 0.81 | 0.0147 | 0.0025 | 4.63E-09 |
| rs3742004 | Triglyceride | TRANS | A | 0.79 | 0.0161 | 0.0028 | 9.77E-09 |
| rs940904 | Triglyceride | TRANS | A | 0.74 | 0.0153 | 0.0026 | 4.18E-09 |
| rs11057408 | Triglyceride | TRANS | T | 0.35 | -0.0222 | 0.0024 | 2.68E-20 |
| rs10846744 | Triglyceride | TRANS | C | 0.16 | 0.0272 | 0.0034 | 1.32E-15 |
| rs7134375 | Triglyceride | TRANS | A | 0.42 | -0.0143 | 0.0020 | 7.43E-13 |
| rs4149056 | Triglyceride | TRANS | T | 0.85 | -0.0320 | 0.0028 | 3.29E-30 |
| rs718314 | Triglyceride | TRANS | A | 0.74 | -0.0192 | 0.0025 | 1.41E-14 |
| rs7979398 | Triglyceride | TRANS | T | 0.46 | 0.0149 | 0.0023 | 9.30E-11 |
| rs11613352 | Triglyceride | TRANS | T | 0.21 | -0.0247 | 0.0027 | 5.84E-20 |
| rs7400722 | Triglyceride | TRANS | A | 0.41 | 0.0155 | 0.0022 | 1.85E-12 |
| rs2298058 | Triglyceride | TRANS | T | 0.32 | 0.0243 | 0.0029 | 5.37E-17 |
| rs7157785 | Triglyceride | TRANS | T | 0.17 | 0.0201 | 0.0028 | 7.06E-13 |
| rs10152471 | Triglyceride | TRANS | A | 0.41 | -0.0158 | 0.0027 | 4.87E-09 |
| rs11634257 | Triglyceride | TRANS | A | 0.27 | 0.0175 | 0.0029 | 1.51E-09 |
| rs17747633 | Triglyceride | TRANS | A | 0.59 | -0.0145 | 0.0021 | 4.65E-12 |
| rs16949992 | Triglyceride | TRANS | C | 0.96 | -0.0745 | 0.0061 | 5.71E-34 |
| rs11858955 | Triglyceride | TRANS | A | 0.01 | -0.1145 | 0.0158 | 3.78E-13 |
| rs493258 | Triglyceride | TRANS | T | 0.49 | 0.0171 | 0.0020 | 1.41E-17 |
| rs12913346 | Triglyceride | TRANS | A | 0.14 | 0.0238 | 0.0036 | 4.09E-11 |
| rs17184382 | Triglyceride | TRANS | A | 0.61 | 0.0174 | 0.0024 | 3.94E-13 |
| rs2415168 | Triglyceride | TRANS | A | 0.74 | 0.0154 | 0.0026 | 3.21E-09 |
| rs11075253 | Triglyceride | TRANS | A | 0.29 | -0.0269 | 0.0034 | 2.56E-15 |
| rs2032915 | Triglyceride | TRANS | T | 0.38 | -0.0173 | 0.0023 | 5.43E-14 |
| rs9939609 | Triglyceride | TRANS | A | 0.40 | 0.0190 | 0.0020 | 1.99E-21 |
| rs1800775 | Triglyceride | TRANS | A | 0.50 | -0.0229 | 0.0021 | 3.40E-27 |
| rs7203984 | Triglyceride | TRANS | A | 0.79 | -0.0220 | 0.0032 | 5.18E-12 |
| rs9940315 | Triglyceride | TRANS | A | 0.43 | -0.0188 | 0.0026 | 4.74E-13 |
| rs2000999 | Triglyceride | TRANS | A | 0.20 | 0.0203 | 0.0026 | 7.25E-15 |
| rs12443634 | Triglyceride | TRANS | A | 0.28 | 0.0184 | 0.0030 | 9.35E-10 |
| rs1053328 | Triglyceride | TRANS | T | 0.36 | 0.0138 | 0.0024 | 8.80E-09 |
| rs143076454 | Triglyceride | TRANS | A | 0.02 | 0.0481 | 0.0083 | 6.83E-09 |
| rs897453 | Triglyceride | TRANS | T | 0.43 | -0.0142 | 0.0020 | 1.08E-12 |
| rs1563631 | Triglyceride | TRANS | T | 0.29 | -0.0171 | 0.0022 | 6.68E-15 |
| rs3110454 | Triglyceride | TRANS | T | 0.42 | -0.0166 | 0.0027 | 7.38E-10 |
| rs2306590 | Triglyceride | TRANS | A | 0.39 | -0.0131 | 0.0021 | 5.02E-10 |
| rs2079005 | Triglyceride | TRANS | T | 0.77 | -0.0219 | 0.0028 | 6.24E-15 |
| rs1662750 | Triglyceride | TRANS | A | 0.45 | -0.0176 | 0.0026 | 1.35E-11 |
| rs2074108 | Triglyceride | TRANS | T | 0.41 | -0.0147 | 0.0024 | 1.15E-09 |
| rs11871606 | Triglyceride | TRANS | A | 0.50 | 0.0163 | 0.0020 | 3.42E-16 |
| rs8075803 | Triglyceride | TRANS | T | 0.50 | -0.0190 | 0.0023 | 1.34E-16 |
| rs12602912 | Triglyceride | TRANS | T | 0.21 | 0.0229 | 0.0024 | 1.20E-21 |
| rs3853818 | Triglyceride | TRANS | T | 0.44 | 0.0158 | 0.0023 | 6.46E-12 |
| rs2125345 | Triglyceride | TRANS | T | 0.69 | 0.0132 | 0.0022 | 1.76E-09 |
| rs2292642 | Triglyceride | TRANS | T | 0.61 | -0.0207 | 0.0020 | 4.27E-25 |
| rs1652343 | Triglyceride | TRANS | T | 0.51 | 0.0156 | 0.0026 | 1.98E-09 |
| rs1862644 | Triglyceride | TRANS | A | 0.48 | -0.0168 | 0.0028 | 2.19E-09 |
| rs117877390 | Triglyceride | TRANS | T | 0.03 | -0.1029 | 0.0108 | 1.73E-21 |
| rs10401969 | Triglyceride | TRANS | T | 0.92 | 0.0946 | 0.0036 | 5.20E-150 |
| rs145702982 | Triglyceride | TRANS | A | 0.99 | 0.1193 | 0.0205 | 6.20E-09 |

| rs8182584 | Triglyceride | TRANS | T | 0.40 | 0.0166 | 0.0021 | 2.74E-15 |
| --- | --- | --- | --- | --- | --- | --- | --- |
| rs1688030 | Triglyceride | TRANS | T | 0.06 | -0.0334 | 0.0046 | 5.52E-13 |
| rs2018519 | Triglyceride | TRANS | T | 0.83 | -0.0209 | 0.0032 | 8.86E-11 |
| rs28399653 | Triglyceride | TRANS | A | 0.03 | 0.0405 | 0.0059 | 5.48E-12 |
| rs4803760 | Triglyceride | TRANS | T | 0.19 | 0.0208 | 0.0031 | 1.34E-11 |
| rs157582 | Triglyceride | TRANS | T | 0.21 | 0.0421 | 0.0042 | 7.54E-24 |
| rs439401 | Triglyceride | TRANS | T | 0.37 | -0.0798 | 0.0025 | 1.15E-229 |
| rs59325138 | Triglyceride | TRANS | T | 0.40 | -0.0400 | 0.0034 | 1.15E-31 |
| rs7259004 | Triglyceride | TRANS | C | 0.10 | 0.0637 | 0.0045 | 2.26E-45 |
| rs2287922 | Triglyceride | TRANS | A | 0.49 | 0.0177 | 0.0021 | 3.97E-17 |
| rs17178414 | Triglyceride | TRANS | T | 0.34 | 0.0188 | 0.0029 | 1.00E-10 |
| rs1132990 | Triglyceride | TRANS | A | 0.81 | -0.0182 | 0.0031 | 4.21E-09 |
| rs1799816 | Triglyceride | TRANS | T | 0.01 | -0.0681 | 0.0110 | 6.44E-10 |
| rs7248104 | Triglyceride | TRANS | A | 0.41 | -0.0191 | 0.0020 | 1.55E-21 |
| rs116843064 | Triglyceride | TRANS | A | 0.02 | -0.2647 | 0.0072 | 3.79E-295 |
| rs140744493 | Triglyceride | TRANS | T | 0.00 | -0.1210 | 0.0189 | 1.55E-10 |
| rs13396091 | Triglyceride | TRANS | A | 0.44 | 0.0138 | 0.0023 | 1.98E-09 |
| rs13389219 | Triglyceride | TRANS | T | 0.40 | -0.0352 | 0.0021 | 2.09E-62 |
| rs16849863 | Triglyceride | TRANS | T | 0.98 | 0.0561 | 0.0079 | 1.60E-12 |
| rs3769823 | Triglyceride | TRANS | A | 0.30 | -0.0138 | 0.0022 | 3.23E-10 |
| rs6435161 | Triglyceride | TRANS | T | 0.71 | 0.0145 | 0.0024 | 1.39E-09 |
| rs1473886 | Triglyceride | TRANS | T | 0.47 | -0.0171 | 0.0023 | 1.20E-13 |
| rs1801701 | Triglyceride | TRANS | T | 0.09 | -0.0302 | 0.0036 | 2.22E-17 |
| rs676210 | Triglyceride | TRANS | A | 0.24 | -0.0765 | 0.0025 | 3.74E-211 |
| rs541041 | Triglyceride | TRANS | A | 0.81 | 0.0232 | 0.0026 | 1.24E-18 |
| rs1344642 | Triglyceride | TRANS | A | 0.44 | -0.0131 | 0.0020 | 5.14E-11 |
| rs2943650 | Triglyceride | TRANS | T | 0.62 | 0.0424 | 0.0029 | 2.88E-48 |
| rs3208747 | Triglyceride | TRANS | T | 1.00 | -0.1500 | 0.0218 | 6.25E-12 |
| rs1049817 | Triglyceride | TRANS | A | 0.60 | 0.0459 | 0.0031 | 5.18E-49 |
| rs11689803 | Triglyceride | TRANS | A | 0.27 | -0.0214 | 0.0035 | 1.02E-09 |
| rs11891554 | Triglyceride | TRANS | A | 0.04 | -0.0956 | 0.0057 | 7.15E-63 |
| rs79593977 | Triglyceride | TRANS | A | 0.01 | 0.2690 | 0.0290 | 1.98E-20 |
| rs780090 | Triglyceride | TRANS | T | 0.09 | 0.0477 | 0.0048 | 1.89E-23 |
| rs147073127 | Triglyceride | TRANS | A | 1.00 | -0.2135 | 0.0194 | 2.71E-28 |
| rs814295 | Triglyceride | TRANS | A | 0.84 | 0.0772 | 0.0038 | 9.28E-90 |
| rs1919128 | Triglyceride | TRANS | A | 0.74 | -0.0457 | 0.0032 | 1.35E-47 |
| rs115289288 | Triglyceride | TRANS | T | 0.02 | -0.2216 | 0.0190 | 1.43E-31 |
| rs4245791 | Triglyceride | TRANS | T | 0.70 | -0.0170 | 0.0022 | 1.10E-14 |
| rs1861410 | Triglyceride | TRANS | T | 0.55 | -0.0156 | 0.0026 | 1.97E-09 |
| rs2723062 | Triglyceride | TRANS | A | 0.36 | -0.0230 | 0.0027 | 1.64E-17 |
| rs2049019 | Triglyceride | TRANS | A | 0.69 | -0.0138 | 0.0024 | 9.49E-09 |
| rs6029143 | Triglyceride | TRANS | T | 0.06 | -0.0351 | 0.0049 | 8.60E-13 |
| rs6016381 | Triglyceride | TRANS | T | 0.63 | 0.0179 | 0.0023 | 8.44E-15 |
| rs1997833 | Triglyceride | TRANS | T | 0.73 | -0.0158 | 0.0026 | 1.19E-09 |
| rs3827066 | Triglyceride | TRANS | T | 0.15 | -0.0428 | 0.0039 | 4.29E-28 |
| rs8123864 | Triglyceride | TRANS | T | 0.66 | -0.0436 | 0.0030 | 2.04E-46 |
| rs1211644 | Triglyceride | TRANS | T | 0.75 | 0.0237 | 0.0030 | 2.84E-15 |
| rs2426428 | Triglyceride | TRANS | T | 0.09 | -0.0308 | 0.0046 | 2.28E-11 |
| rs6025606 | Triglyceride | TRANS | T | 0.36 | -0.0130 | 0.0021 | 7.37E-10 |
| rs41302559 | Triglyceride | TRANS | A | 0.00 | -0.1447 | 0.0205 | 1.83E-12 |
| rs114139997 | Triglyceride | TRANS | A | 0.00 | -0.3646 | 0.0385 | 2.85E-21 |
| rs200559406 | Triglyceride | TRANS | A | 0.00 | 0.2821 | 0.0362 | 6.63E-15 |
| rs35665085 | Triglyceride | TRANS | A | 0.05 | 0.0317 | 0.0045 | 1.87E-12 |
| rs738322 | Triglyceride | TRANS | A | 0.52 | 0.0210 | 0.0020 | 9.23E-26 |
| rs5757161 | Triglyceride | TRANS | A | 0.36 | 0.0160 | 0.0025 | 1.60E-10 |
| rs738409 | Triglyceride | TRANS | C | 0.77 | 0.0166 | 0.0024 | 4.02E-12 |
| rs1801282 | Triglyceride | TRANS | C | 0.88 | 0.0339 | 0.0032 | 3.66E-26 |
| rs17819328 | Triglyceride | TRANS | T | 0.58 | -0.0252 | 0.0024 | 3.30E-26 |

| rs7621025 | Triglyceride | TRANS | T | 0.22 | -0.0212 | 0.0034 | 4.14E-10 |
| --- | --- | --- | --- | --- | --- | --- | --- |
| rs4683438 | Triglyceride | TRANS | T | 0.35 | -0.0172 | 0.0024 | 8.14E-13 |
| rs382534 | Triglyceride | TRANS | T | 0.25 | 0.0152 | 0.0026 | 4.53E-09 |
| rs9822326 | Triglyceride | TRANS | A | 0.61 | 0.0159 | 0.0024 | 3.75E-11 |
| rs10513687 | Triglyceride | TRANS | T | 0.14 | 0.0215 | 0.0033 | 6.65E-11 |
| rs17600346 | Triglyceride | TRANS | T | 0.96 | -0.0464 | 0.0070 | 3.34E-11 |
| rs13326165 | Triglyceride | TRANS | A | 0.20 | -0.0169 | 0.0025 | 1.38E-11 |
| rs1126673 | Triglyceride | TRANS | T | 0.71 | 0.0178 | 0.0022 | 5.63E-16 |
| rs13107325 | Triglyceride | TRANS | T | 0.07 | 0.0327 | 0.0041 | 1.69E-15 |
| rs41278045 | Triglyceride | TRANS | A | 1.00 | -0.1991 | 0.0287 | 3.97E-12 |
| rs6054 | Triglyceride | TRANS | T | 0.00 | 0.1234 | 0.0180 | 7.19E-12 |
| rs6855363 | Triglyceride | TRANS | T | 0.66 | 0.0165 | 0.0024 | 5.94E-12 |
| rs9884830 | Triglyceride | TRANS | T | 0.17 | 0.0190 | 0.0030 | 2.40E-10 |
| rs3748034 | Triglyceride | TRANS | T | 0.14 | 0.0280 | 0.0031 | 2.94E-19 |
| rs16844401 | Triglyceride | TRANS | A | 0.07 | 0.0324 | 0.0042 | 1.01E-14 |
| rs6831256 | Triglyceride | TRANS | A | 0.56 | -0.0141 | 0.0021 | 1.71E-11 |
| rs1037814 | Triglyceride | TRANS | T | 0.40 | -0.0245 | 0.0024 | 2.77E-24 |
| rs10029254 | Triglyceride | TRANS | T | 0.22 | 0.0210 | 0.0028 | 1.11E-13 |
| rs13133548 | Triglyceride | TRANS | A | 0.48 | 0.0118 | 0.0020 | 3.27E-09 |
| rs6599389 | Triglyceride | TRANS | A | 0.08 | 0.0221 | 0.0038 | 6.88E-09 |
| rs11248060 | Triglyceride | TRANS | T | 0.12 | 0.0224 | 0.0031 | 6.14E-13 |
| rs1045241 | Triglyceride | TRANS | T | 0.27 | -0.0172 | 0.0026 | 3.71E-11 |
| rs26008 | Triglyceride | TRANS | T | 0.07 | 0.0241 | 0.0038 | 2.28E-10 |
| rs4705986 | Triglyceride | TRANS | T | 0.94 | 0.0340 | 0.0058 | 4.41E-09 |
| rs4704820 | Triglyceride | TRANS | T | 0.81 | 0.0217 | 0.0032 | 1.03E-11 |
| rs6882076 | Triglyceride | TRANS | T | 0.37 | -0.0417 | 0.0022 | 6.74E-83 |
| rs1650527 | Triglyceride | TRANS | T | 0.23 | 0.0251 | 0.0031 | 5.09E-16 |
| rs4311394 | Triglyceride | TRANS | A | 0.74 | -0.0203 | 0.0022 | 3.44E-20 |
| rs459193 | Triglyceride | TRANS | A | 0.27 | -0.0354 | 0.0023 | 3.67E-52 |
| rs2448428 | Triglyceride | TRANS | T | 0.41 | 0.0151 | 0.0024 | 4.29E-10 |
| rs9686661 | Triglyceride | TRANS | T | 0.19 | 0.0439 | 0.0025 | 2.29E-67 |
| rs4976033 | Triglyceride | TRANS | A | 0.58 | -0.0160 | 0.0020 | 1.25E-15 |
| rs2745353 | Triglyceride | TRANS | T | 0.52 | 0.0185 | 0.0020 | 1.92E-20 |
| rs9388768 | Triglyceride | TRANS | A | 0.66 | 0.0128 | 0.0021 | 1.16E-09 |
| rs643381 | Triglyceride | TRANS | A | 0.51 | -0.0236 | 0.0020 | 3.17E-32 |
| rs12208357 | Triglyceride | TRANS | T | 0.07 | 0.0298 | 0.0040 | 1.12E-13 |
| rs2665357 | Triglyceride | TRANS | A | 0.49 | -0.0184 | 0.0023 | 1.88E-15 |
| rs645718 | Triglyceride | TRANS | A | 0.05 | -0.0337 | 0.0053 | 2.13E-10 |
| rs2524060 | Triglyceride | TRANS | A | 0.24 | -0.0285 | 0.0031 | 3.31E-20 |
| rs2442719 | Triglyceride | TRANS | T | 0.55 | -0.0242 | 0.0021 | 7.45E-32 |
| rs17207867 | Triglyceride | TRANS | T | 0.09 | 0.0266 | 0.0037 | 5.04E-13 |
| rs9271366 | Triglyceride | TRANS | A | 0.85 | 0.0279 | 0.0031 | 7.40E-19 |
| rs9273368 | Triglyceride | TRANS | A | 0.27 | -0.0179 | 0.0031 | 9.28E-09 |
| rs11752643 | Triglyceride | TRANS | T | 0.03 | 0.0663 | 0.0063 | 3.69E-26 |
| rs2395655 | Triglyceride | TRANS | A | 0.61 | 0.0165 | 0.0023 | 6.58E-13 |
| rs6458349 | Triglyceride | TRANS | A | 0.73 | 0.0383 | 0.0030 | 4.13E-37 |
| rs78807370 | Triglyceride | TRANS | A | 0.15 | -0.0433 | 0.0037 | 3.92E-31 |
| rs881858 | Triglyceride | TRANS | A | 0.69 | 0.0204 | 0.0022 | 1.85E-20 |
| rs4715316 | Triglyceride | TRANS | T | 0.64 | -0.0155 | 0.0027 | 8.51E-09 |
| rs287621 | Triglyceride | TRANS | T | 0.28 | 0.0223 | 0.0025 | 4.70E-19 |
| rs3735080 | Triglyceride | TRANS | T | 0.23 | 0.0160 | 0.0024 | 2.62E-11 |
| rs16884656 | Triglyceride | TRANS | T | 0.88 | -0.0242 | 0.0042 | 8.31E-09 |
| rs12699758 | Triglyceride | TRANS | A | 0.25 | 0.0159 | 0.0027 | 3.64E-09 |
| rs4410790 | Triglyceride | TRANS | T | 0.39 | -0.0121 | 0.0020 | 1.41E-09 |
| rs10235225 | Triglyceride | TRANS | A | 0.60 | 0.0148 | 0.0024 | 1.33E-09 |
| rs4722551 | Triglyceride | TRANS | T | 0.84 | 0.0192 | 0.0028 | 1.14E-11 |
| rs1534696 | Triglyceride | TRANS | A | 0.56 | -0.0191 | 0.0024 | 2.90E-15 |
| rs2070971 | Triglyceride | TRANS | T | 0.15 | 0.0234 | 0.0034 | 7.19E-12 |

| rs3757838 | Triglyceride | TRANS | A | 0.93 | 0.0299 | 0.0045 | 3.76E-11 |
| --- | --- | --- | --- | --- | --- | --- | --- |
| rs1178979 | Triglyceride | TRANS | T | 0.81 | 0.0439 | 0.0038 | 1.11E-30 |
| rs799158 | Triglyceride | TRANS | T | 0.04 | 0.0756 | 0.0080 | 4.02E-21 |
| rs3812316 | Triglyceride | TRANS | C | 0.88 | 0.0872 | 0.0045 | 4.26E-82 |
| rs11776767 | Triglyceride | TRANS | C | 0.37 | 0.0236 | 0.0022 | 1.34E-27 |
| rs2686187 | Triglyceride | TRANS | A | 0.42 | -0.0142 | 0.0024 | 3.71E-09 |
| rs3947 | Triglyceride | TRANS | A | 0.25 | 0.0194 | 0.0025 | 7.14E-15 |
| rs17730649 | Triglyceride | TRANS | A | 0.41 | 0.0215 | 0.0026 | 3.80E-16 |
| rs6982502 | Triglyceride | TRANS | T | 0.52 | -0.0278 | 0.0048 | 5.11E-09 |
| rs2980876 | Triglyceride | TRANS | T | 0.68 | -0.0911 | 0.0050 | 1.40E-73 |
| rs8180991 | Triglyceride | TRANS | C | 0.77 | -0.0345 | 0.0034 | 2.58E-24 |
| rs4871624 | Triglyceride | TRANS | T | 0.73 | -0.0203 | 0.0026 | 1.05E-14 |
| rs1495741 | Triglyceride | TRANS | A | 0.76 | -0.0364 | 0.0023 | 3.41E-56 |
| rs1801177 | Triglyceride | TRANS | A | 0.02 | 0.0947 | 0.0089 | 1.13E-26 |
| rs264 | Triglyceride | TRANS | A | 0.14 | -0.0280 | 0.0034 | 7.93E-17 |
| rs268 | Triglyceride | TRANS | A | 0.98 | -0.2325 | 0.0077 | 5.48E-202 |
| rs301 | Triglyceride | TRANS | T | 0.74 | 0.0619 | 0.0049 | 2.83E-36 |
| rs312 | Triglyceride | TRANS | C | 0.12 | 0.0476 | 0.0064 | 1.40E-13 |
| rs326 | Triglyceride | TRANS | A | 0.70 | 0.0779 | 0.0047 | 6.65E-63 |
| rs12545984 | Triglyceride | TRANS | T | 0.16 | 0.0435 | 0.0059 | 2.08E-13 |
| rs17091905 | Triglyceride | TRANS | A | 0.13 | -0.0416 | 0.0056 | 1.50E-13 |
| rs10105418 | Triglyceride | TRANS | A | 0.03 | 0.0818 | 0.0086 | 1.89E-21 |
| rs4637851 | Triglyceride | TRANS | A | 0.66 | -0.0291 | 0.0030 | 8.54E-22 |
| rs13256965 | Triglyceride | TRANS | A | 0.61 | 0.0170 | 0.0025 | 7.53E-12 |
| rs3736147 | Triglyceride | TRANS | A | 0.33 | 0.0179 | 0.0028 | 1.66E-10 |
| rs1982768 | Triglyceride | TRANS | A | 0.85 | -0.0220 | 0.0037 | 3.01E-09 |
| rs2081687 | Triglyceride | TRANS | T | 0.34 | 0.0191 | 0.0021 | 9.50E-20 |
| rs4738141 | Triglyceride | TRANS | A | 0.74 | -0.0169 | 0.0028 | 1.59E-09 |
| rs4240624 | Triglyceride | TRANS | A | 0.92 | -0.0342 | 0.0040 | 1.23E-17 |
| rs1800978 | Triglyceride | TRANS | C | 0.87 | 0.0270 | 0.0037 | 2.95E-13 |
| rs3927680 | Triglyceride | TRANS | A | 0.55 | -0.0160 | 0.0020 | 1.25E-15 |
| rs4120895 | Triglyceride | TRANS | T | 0.62 | -0.0178 | 0.0027 | 4.34E-11 |
| rs11264477 | Hemoglobin A1c | EA | C | 0.24 | 0.0286 | 0.0040 | 2.44E-11 |
| rs12601 | Hemoglobin A1c | EA | A | 0.44 | 0.0218 | 0.0040 | 2.86E-08 |
| rs895636 | Hemoglobin A1c | EA | T | 0.17 | 0.0583 | 0.0070 | 1.46E-16 |
| rs16856247 | Hemoglobin A1c | EA | C | 0.02 | -0.0233 | 0.0040 | 9.07E-09 |
| rs7356034 | Hemoglobin A1c | EA | A | 0.28 | -0.0513 | 0.0090 | 4.84E-09 |
| rs76922886 | Hemoglobin A1c | EA | A | 0.31 | 0.0573 | 0.0070 | 1.96E-14 |
| rs9368222 | Hemoglobin A1c | EA | A | 0.28 | 0.0237 | 0.0040 | 8.92E-10 |
| rs9399137 | Hemoglobin A1c | EA | T | 0.26 | 0.0705 | 0.0100 | 8.50E-15 |
| rs2908289 | Hemoglobin A1c | EA | A | 0.18 | 0.0392 | 0.0050 | 4.17E-17 |
| rs4737009 | Hemoglobin A1c | EA | A | 0.25 | 0.0247 | 0.0040 | 1.11E-10 |
| rs11558471 | Hemoglobin A1c | EA | A | 0.29 | 0.0208 | 0.0040 | 1.35E-08 |
| rs10965243 | Hemoglobin A1c | EA | G | 0.09 | -0.0587 | 0.0070 | 1.09E-15 |
| rs7045859 | Hemoglobin A1c | EA | G | 0.40 | -0.0534 | 0.0080 | 8.19E-11 |
| rs12221133 | Hemoglobin A1c | EA | A | 0.26 | 0.0459 | 0.0070 | 1.10E-11 |
| rs12219514 | Hemoglobin A1c | EA | G | 0.44 | -0.0694 | 0.0100 | 1.57E-11 |
| rs7901695 | Hemoglobin A1c | EA | C | 0.34 | 0.1169 | 0.0170 | 2.93E-12 |
| rs3782123 | Hemoglobin A1c | EA | A | 0.28 | -0.0419 | 0.0070 | 7.21E-10 |
| rs2237892 | Hemoglobin A1c | EA | T | 0.06 | -0.0780 | 0.0070 | 2.91E-29 |
| rs174584 | Hemoglobin A1c | EA | A | 0.37 | -0.0398 | 0.0070 | 9.67E-09 |
| rs10830963 | Hemoglobin A1c | EA | C | 0.29 | -0.0222 | 0.0040 | 7.04E-09 |
| rs4886869 | Hemoglobin A1c | EA | G | 0.41 | 0.0402 | 0.0070 | 9.54E-09 |
| rs9933309 | Hemoglobin A1c | EA | C | 0.29 | 0.0705 | 0.0100 | 1.10E-08 |
| rs8068430 | Hemoglobin A1c | EA | C | 0.20 | 0.0497 | 0.0080 | 2.40E-09 |
| rs1046875 | Hemoglobin A1c | EA | A | 0.35 | 0.0227 | 0.0040 | 4.14E-10 |
| rs11086054 | Hemoglobin A1c | EA | A | 0.29 | 0.0237 | 0.0040 | 2.37E-09 |
| rs57601949 | Hemoglobin A1c | EA | T | 0.12 | 0.0421 | 0.0070 | 4.51E-09 |

| rs2301142 | Hemoglobin A1c | EA | G | 0.16 | -0.0408 | 0.0060 | 9.92E-13 |
| --- | --- | --- | --- | --- | --- | --- | --- |
| rs4846565 | Fasting Isulin(BMI adjusted) | EUP | G | 0.33 | 0.0129 | 0.0020 | 1.76E-09 |
| rs780094 | Fasting Isulin(BMI adjusted) | EUP | T | 0.41 | -0.0192 | 0.0030 | 3.36E-12 |
| rs10195252 | Fasting Isulin(BMI adjusted) | EUP | T | 0.44 | 0.0178 | 0.0020 | 1.26E-16 |
| rs2943645 | Fasting Isulin(BMI adjusted) | EUP | T | 0.38 | 0.0188 | 0.0020 | 2.26E-19 |
| rs3822072 | Fasting Isulin(BMI adjusted) | EUP | A | 0.48 | 0.0119 | 0.0020 | 1.80E-08 |
| rs974801 | Fasting Isulin(BMI adjusted) | EUP | G | 0.41 | 0.0139 | 0.0020 | 3.27E-11 |
| rs6822892 | Fasting Isulin(BMI adjusted) | EUP | A | ##### | 0.0139 | 0.0020 | 2.58E-10 |
| rs4865796 | Fasting Isulin(BMI adjusted) | EUP | A | 0.30 | 0.0159 | 0.0020 | 2.16E-12 |
| rs459193 | Fasting Isulin(BMI adjusted) | EUP | G | 0.29 | 0.0149 | 0.0020 | 1.15E-10 |
| rs6912327 | Fasting Isulin(BMI adjusted) | EUP | T | 0.24 | 0.0169 | 0.0030 | 2.26E-08 |
| rs35747 | Fasting Isulin(BMI adjusted) | EUP | A | 0.17 | 0.0218 | 0.0040 | 8.85E-10 |
| rs731839 | Fasting Isulin(BMI adjusted) | EUP | G | 0.33 | 0.0149 | 0.0020 | 5.13E-12 |
| rs602662 | Vitamin B12 level | EUR | G | 0.44 | -0.0700 | 0.0100 | 1.83E-15 |
| rs601338 | Vitamin B12 level | EUR | A | 0.45 | 0.0600 | 0.0100 | 6.92E-15 |
| rs492602 | Vitamin B12 level | EUR | G | 0.44 | 0.0600 | 0.0100 | 1.30E-14 |
| rs1801222 | Vitamin B12 level | EUR | A | 0.28 | -0.0500 | 0.0100 | 2.87E-09 |
| rs526934 | Vitamin B12 level | EUR | G | 0.27 | -0.0500 | 0.0100 | 2.25E-10 |
| rs9473558 | Vitamin B12 level | EUR | T | 0.35 | -0.0400 | 0.0100 | 4.05E-08 |
| rs9473555 | Vitamin B12 level | EUR | C | 0.35 | -0.0400 | 0.0100 | 4.91E-08 |
| rs10100245 | Body Fat Percentage | TRANS | A | 0.55 | 0.0190 | 0.0025 | 1.93E-14 |
| rs1013293 | Body Fat Percentage | TRANS | A | 0.42 | -0.0181 | 0.0025 | 3.24E-13 |
| rs10172196 | Body Fat Percentage | TRANS | A | 0.34 | 0.0169 | 0.0027 | 2.45E-10 |
| rs10259620 | Body Fat Percentage | TRANS | G | 0.78 | -0.0206 | 0.0030 | 6.24E-12 |
| rs10278040 | Body Fat Percentage | TRANS | A | 0.08 | -0.0312 | 0.0051 | 1.31E-09 |
| rs1038088 | Body Fat Percentage | TRANS | G | 0.51 | 0.0156 | 0.0025 | 2.53E-10 |
| rs10423928 | Body Fat Percentage | TRANS | A | 0.21 | -0.0299 | 0.0031 | 7.47E-22 |
| rs1046080 | Body Fat Percentage | TRANS | A | 0.77 | 0.0238 | 0.0027 | 2.94E-18 |
| rs10496731 | Body Fat Percentage | TRANS | G | 0.51 | -0.0157 | 0.0026 | 7.82E-10 |
| rs10499014 | Body Fat Percentage | TRANS | G | 0.27 | -0.0177 | 0.0028 | 1.61E-10 |
| rs10510025 | Body Fat Percentage | TRANS | T | 0.26 | 0.0182 | 0.0029 | 1.77E-10 |
| rs10514641 | Body Fat Percentage | TRANS | G | 0.33 | 0.0166 | 0.0027 | 4.45E-10 |
| rs1056441 | Body Fat Percentage | TRANS | C | 0.72 | 0.0179 | 0.0026 | 8.69E-12 |
| rs10732335 | Body Fat Percentage | TRANS | C | 0.53 | -0.0277 | 0.0025 | 4.84E-29 |
| rs10756798 | Body Fat Percentage | TRANS | T | 0.64 | -0.0203 | 0.0026 | 2.65E-15 |
| rs10758031 | Body Fat Percentage | TRANS | C | 0.79 | -0.0165 | 0.0028 | 4.50E-09 |
| rs10766077 | Body Fat Percentage | TRANS | A | 0.35 | -0.0161 | 0.0026 | 2.99E-10 |
| rs10774018 | Body Fat Percentage | TRANS | C | 0.25 | 0.0168 | 0.0030 | 1.81E-08 |
| rs10791109 | Body Fat Percentage | TRANS | G | 0.50 | 0.0159 | 0.0025 | 9.55E-11 |
| rs10803762 | Body Fat Percentage | TRANS | A | 0.68 | 0.0157 | 0.0026 | 2.24E-09 |
| rs10830566 | Body Fat Percentage | TRANS | G | 0.31 | -0.0154 | 0.0027 | 2.04E-08 |
| rs10848835 | Body Fat Percentage | TRANS | C | 0.10 | -0.0289 | 0.0041 | 3.09E-12 |
| rs10854853 | Body Fat Percentage | TRANS | T | 0.44 | 0.0137 | 0.0025 | 3.01E-08 |
| rs10938397 | Body Fat Percentage | TRANS | G | 0.42 | 0.0256 | 0.0025 | 5.59E-25 |
| rs10947510 | Body Fat Percentage | TRANS | C | 0.47 | -0.0144 | 0.0025 | 4.69E-09 |
| rs10947793 | Body Fat Percentage | TRANS | G | 0.35 | -0.0154 | 0.0025 | 1.38E-09 |
| rs10954772 | Body Fat Percentage | TRANS | C | 0.70 | -0.0176 | 0.0026 | 2.82E-11 |
| rs10999460 | Body Fat Percentage | TRANS | T | 0.24 | 0.0221 | 0.0028 | 2.01E-15 |
| rs11012732 | Body Fat Percentage | TRANS | G | 0.35 | 0.0237 | 0.0026 | 1.04E-19 |
| rs11030016 | Body Fat Percentage | TRANS | T | 0.72 | 0.0158 | 0.0028 | 1.93E-08 |
| rs11042030 | Body Fat Percentage | TRANS | C | 0.32 | -0.0182 | 0.0028 | 4.24E-11 |
| rs11113445 | Body Fat Percentage | TRANS | G | 0.42 | 0.0186 | 0.0025 | 1.34E-13 |
| rs11119364 | Body Fat Percentage | TRANS | T | 0.16 | 0.0210 | 0.0032 | 4.35E-11 |
| rs11150745 | Body Fat Percentage | TRANS | G | 0.32 | -0.0170 | 0.0026 | 1.22E-10 |
| rs11258438 | Body Fat Percentage | TRANS | A | 0.57 | 0.0135 | 0.0025 | 4.58E-08 |
| rs113019802 | Body Fat Percentage | TRANS | A | 0.20 | -0.0187 | 0.0029 | 1.98E-10 |
| rs113230003 | Body Fat Percentage | TRANS | A | 0.24 | -0.0197 | 0.0028 | 2.01E-12 |
| rs114052060 | Body Fat Percentage | TRANS | G | 0.04 | -0.0385 | 0.0053 | 5.10E-13 |

| rs114295766 | Body Fat Percentage | TRANS | T | 0.10 | -0.0268 | 0.0048 | 2.68E-08 |
| --- | --- | --- | --- | --- | --- | --- | --- |
| rs11594905 | Body Fat Percentage | TRANS | A | 0.13 | 0.0201 | 0.0036 | 1.99E-08 |
| rs11603783 | Body Fat Percentage | TRANS | C | 0.27 | 0.0167 | 0.0029 | 4.66E-09 |
| rs116298073 | Body Fat Percentage | TRANS | T | 0.05 | -0.0329 | 0.0051 | 1.48E-10 |
| rs116399833 | Body Fat Percentage | TRANS | A | 0.24 | 0.0167 | 0.0029 | 1.15E-08 |
| rs11642015 | Body Fat Percentage | TRANS | T | 0.43 | 0.0521 | 0.0025 | 3.10E-96 |
| rs116792274 | Body Fat Percentage | TRANS | T | 0.03 | -0.0349 | 0.0063 | 3.25E-08 |
| rs11685627 | Body Fat Percentage | TRANS | A | 0.50 | -0.0136 | 0.0025 | 3.50E-08 |
| rs11695700 | Body Fat Percentage | TRANS | T | 0.43 | 0.0139 | 0.0025 | 2.35E-08 |
| rs117068593 | Body Fat Percentage | TRANS | T | 0.19 | -0.0190 | 0.0031 | 1.36E-09 |
| rs11742930 | Body Fat Percentage | TRANS | T | 0.60 | 0.0143 | 0.0025 | 7.75E-09 |
| rs117632017 | Body Fat Percentage | TRANS | A | 0.03 | 0.0363 | 0.0063 | 1.04E-08 |
| rs11781222 | Body Fat Percentage | TRANS | C | 0.13 | -0.0207 | 0.0036 | 5.91E-09 |
| rs11782341 | Body Fat Percentage | TRANS | G | 0.18 | 0.0196 | 0.0031 | 4.58E-10 |
| rs11786089 | Body Fat Percentage | TRANS | G | 0.46 | 0.0155 | 0.0025 | 3.42E-10 |
| rs11790018 | Body Fat Percentage | TRANS | G | 0.38 | -0.0141 | 0.0025 | 3.22E-08 |
| rs11810577 | Body Fat Percentage | TRANS | T | 0.18 | 0.0181 | 0.0031 | 8.75E-09 |
| rs1182143 | Body Fat Percentage | TRANS | C | 0.29 | -0.0162 | 0.0027 | 1.90E-09 |
| rs11852419 | Body Fat Percentage | TRANS | T | 0.27 | 0.0165 | 0.0028 | 3.45E-09 |
| rs11856579 | Body Fat Percentage | TRANS | A | 0.22 | -0.0166 | 0.0028 | 2.14E-09 |
| rs11857221 | Body Fat Percentage | TRANS | A | 0.37 | 0.0142 | 0.0025 | 1.59E-08 |
| rs11866219 | Body Fat Percentage | TRANS | C | 0.56 | -0.0221 | 0.0025 | 7.41E-19 |
| rs11873305 | Body Fat Percentage | TRANS | C | 0.03 | -0.0588 | 0.0063 | 1.76E-20 |
| rs11943456 | Body Fat Percentage | TRANS | C | 0.46 | 0.0170 | 0.0025 | 5.74E-12 |
| rs12037698 | Body Fat Percentage | TRANS | A | 0.12 | -0.0222 | 0.0035 | 2.86E-10 |
| rs12072739 | Body Fat Percentage | TRANS | G | 0.22 | 0.0183 | 0.0029 | 5.43E-10 |
| rs12124126 | Body Fat Percentage | TRANS | G | 0.32 | -0.0149 | 0.0026 | 1.12E-08 |
| rs12127506 | Body Fat Percentage | TRANS | A | 0.35 | -0.0148 | 0.0026 | 1.96E-08 |
| rs1218822 | Body Fat Percentage | TRANS | A | 0.68 | 0.0155 | 0.0026 | 2.50E-09 |
| rs12218858 | Body Fat Percentage | TRANS | T | 0.55 | 0.0146 | 0.0025 | 3.90E-09 |
| rs12339822 | Body Fat Percentage | TRANS | G | 0.54 | 0.0185 | 0.0025 | 5.97E-14 |
| rs12367809 | Body Fat Percentage | TRANS | T | 0.34 | 0.0250 | 0.0025 | 1.13E-22 |
| rs12370302 | Body Fat Percentage | TRANS | C | 0.11 | 0.0225 | 0.0038 | 2.13E-09 |
| rs12477088 | Body Fat Percentage | TRANS | C | 0.40 | -0.0161 | 0.0025 | 1.26E-10 |
| rs12477385 | Body Fat Percentage | TRANS | T | 0.22 | -0.0172 | 0.0029 | 4.11E-09 |
| rs12550674 | Body Fat Percentage | TRANS | C | 0.71 | -0.0152 | 0.0026 | 6.54E-09 |
| rs12568522 | Body Fat Percentage | TRANS | A | 0.32 | -0.0149 | 0.0027 | 2.51E-08 |
| rs12616638 | Body Fat Percentage | TRANS | G | 0.44 | -0.0137 | 0.0025 | 3.74E-08 |
| rs12619178 | Body Fat Percentage | TRANS | T | 0.39 | -0.0161 | 0.0025 | 1.37E-10 |
| rs12622267 | Body Fat Percentage | TRANS | G | 0.39 | -0.0165 | 0.0025 | 9.35E-11 |
| rs1262557 | Body Fat Percentage | TRANS | T | 0.48 | 0.0168 | 0.0025 | 7.66E-12 |
| rs12679106 | Body Fat Percentage | TRANS | T | 0.70 | -0.0225 | 0.0027 | 9.33E-17 |
| rs12702528 | Body Fat Percentage | TRANS | T | 0.46 | -0.0144 | 0.0025 | 5.42E-09 |
| rs12890931 | Body Fat Percentage | TRANS | G | 0.36 | 0.0158 | 0.0026 | 6.14E-10 |
| rs12901071 | Body Fat Percentage | TRANS | G | 0.34 | 0.0160 | 0.0026 | 8.66E-10 |
| rs12923476 | Body Fat Percentage | TRANS | A | 0.26 | -0.0203 | 0.0028 | 5.66E-13 |
| rs1296328 | Body Fat Percentage | TRANS | C | 0.55 | -0.0162 | 0.0025 | 5.40E-11 |
| rs13062093 | Body Fat Percentage | TRANS | G | 0.36 | 0.0170 | 0.0026 | 2.74E-11 |
| rs13069856 | Body Fat Percentage | TRANS | C | 0.06 | -0.0277 | 0.0049 | 2.18E-08 |
| rs13109830 | Body Fat Percentage | TRANS | G | 0.48 | -0.0147 | 0.0025 | 2.60E-09 |
| rs13135092 | Body Fat Percentage | TRANS | G | 0.09 | 0.0476 | 0.0044 | 1.10E-26 |
| rs13174863 | Body Fat Percentage | TRANS | G | 0.17 | 0.0206 | 0.0035 | 2.80E-09 |
| rs1322842 | Body Fat Percentage | TRANS | G | 0.65 | -0.0142 | 0.0025 | 1.69E-08 |
| rs1322998 | Body Fat Percentage | TRANS | A | 0.42 | 0.0139 | 0.0025 | 2.07E-08 |
| rs1324088 | Body Fat Percentage | TRANS | A | 0.11 | 0.0195 | 0.0035 | 3.34E-08 |
| rs1335055 | Body Fat Percentage | TRANS | A | 0.68 | -0.0147 | 0.0026 | 1.29E-08 |
| rs136309 | Body Fat Percentage | TRANS | A | 0.19 | 0.0173 | 0.0030 | 1.27E-08 |
| rs141403828 | Body Fat Percentage | TRANS | T | 0.02 | -0.0607 | 0.0110 | 3.23E-08 |

| rs1414506 | Body Fat Percentage | TRANS | T | 0.62 | 0.0154 | 0.0026 | 2.63E-09 |
| --- | --- | --- | --- | --- | --- | --- | --- |
| rs1415475 | Body Fat Percentage | TRANS | C | 0.06 | -0.0334 | 0.0051 | 4.00E-11 |
| rs1441264 | Body Fat Percentage | TRANS | A | 0.63 | 0.0164 | 0.0025 | 4.91E-11 |
| rs145350287 | Body Fat Percentage | TRANS | A | 0.03 | -0.0461 | 0.0063 | 2.56E-13 |
| rs1460940 | Body Fat Percentage | TRANS | A | 0.82 | 0.0269 | 0.0031 | 2.81E-18 |
| rs147730268 | Body Fat Percentage | TRANS | T | 0.07 | -0.0397 | 0.0043 | 1.93E-20 |
| rs1478804 | Body Fat Percentage | TRANS | A | 0.78 | 0.0165 | 0.0030 | 4.74E-08 |
| rs1486921 | Body Fat Percentage | TRANS | T | 0.29 | -0.0149 | 0.0027 | 3.17E-08 |
| rs1491592 | Body Fat Percentage | TRANS | T | 0.55 | 0.0143 | 0.0025 | 7.40E-09 |
| rs1503526 | Body Fat Percentage | TRANS | C | 0.51 | 0.0161 | 0.0025 | 6.20E-11 |
| rs1535274 | Body Fat Percentage | TRANS | C | 0.56 | 0.0171 | 0.0025 | 1.00E-11 |
| rs1538742 | Body Fat Percentage | TRANS | A | 0.59 | -0.0255 | 0.0025 | 9.23E-25 |
| rs1568489 | Body Fat Percentage | TRANS | G | 0.56 | 0.0152 | 0.0025 | 1.02E-09 |
| rs157845 | Body Fat Percentage | TRANS | C | 0.71 | -0.0167 | 0.0028 | 3.45E-09 |
| rs1609906 | Body Fat Percentage | TRANS | A | 0.55 | -0.0204 | 0.0025 | 9.13E-17 |
| rs1653892 | Body Fat Percentage | TRANS | C | 0.71 | -0.0171 | 0.0027 | 1.66E-10 |
| rs16934748 | Body Fat Percentage | TRANS | C | 0.15 | 0.0190 | 0.0034 | 3.11E-08 |
| rs16940859 | Body Fat Percentage | TRANS | G | 0.16 | -0.0179 | 0.0031 | 1.13E-08 |
| rs16951304 | Body Fat Percentage | TRANS | C | 0.22 | -0.0268 | 0.0030 | 9.49E-19 |
| rs1701820 | Body Fat Percentage | TRANS | A | 0.51 | -0.0147 | 0.0025 | 2.49E-09 |
| rs17024393 | Body Fat Percentage | TRANS | C | 0.03 | 0.0598 | 0.0078 | 1.17E-14 |
| rs17115183 | Body Fat Percentage | TRANS | T | 0.41 | 0.0149 | 0.0025 | 2.59E-09 |
| rs17265513 | Body Fat Percentage | TRANS | C | 0.19 | 0.0185 | 0.0031 | 1.85E-09 |
| rs17296856 | Body Fat Percentage | TRANS | C | 0.26 | -0.0270 | 0.0027 | 5.13E-23 |
| rs1731260 | Body Fat Percentage | TRANS | T | 0.50 | 0.0151 | 0.0025 | 8.19E-10 |
| rs17704028 | Body Fat Percentage | TRANS | T | 0.13 | -0.0190 | 0.0035 | 4.29E-08 |
| rs17770336 | Body Fat Percentage | TRANS | T | 0.30 | 0.0207 | 0.0026 | 2.81E-15 |
| rs1783541 | Body Fat Percentage | TRANS | T | 0.19 | 0.0166 | 0.0030 | 2.29E-08 |
| rs1861410 | Body Fat Percentage | TRANS | T | 0.57 | -0.0183 | 0.0025 | 1.39E-13 |
| rs1881505 | Body Fat Percentage | TRANS | C | 0.95 | -0.0294 | 0.0053 | 2.22E-08 |
| rs1891215 | Body Fat Percentage | TRANS | C | 0.43 | 0.0139 | 0.0025 | 1.61E-08 |
| rs1893659 | Body Fat Percentage | TRANS | A | 0.57 | -0.0200 | 0.0025 | 4.42E-16 |
| rs1928185 | Body Fat Percentage | TRANS | C | 0.17 | 0.0305 | 0.0033 | 1.35E-20 |
| rs1928496 | Body Fat Percentage | TRANS | T | 0.73 | 0.0177 | 0.0028 | 3.11E-10 |
| rs2002023 | Body Fat Percentage | TRANS | T | 0.44 | 0.0155 | 0.0025 | 5.42E-10 |
| rs2038646 | Body Fat Percentage | TRANS | G | 0.59 | 0.0186 | 0.0025 | 1.24E-13 |
| rs2042864 | Body Fat Percentage | TRANS | C | 0.37 | 0.0163 | 0.0025 | 6.65E-11 |
| rs2043016 | Body Fat Percentage | TRANS | T | 0.32 | 0.0161 | 0.0025 | 2.11E-10 |
| rs2052607 | Body Fat Percentage | TRANS | A | 0.35 | -0.0196 | 0.0026 | 3.95E-14 |
| rs208015 | Body Fat Percentage | TRANS | C | 0.91 | -0.0336 | 0.0049 | 6.39E-12 |
| rs2112347 | Body Fat Percentage | TRANS | G | 0.37 | -0.0228 | 0.0026 | 6.07E-19 |
| rs2124499 | Body Fat Percentage | TRANS | C | 0.37 | -0.0169 | 0.0025 | 3.54E-11 |
| rs2172131 | Body Fat Percentage | TRANS | C | 0.53 | -0.0168 | 0.0025 | 1.52E-11 |
| rs217672 | Body Fat Percentage | TRANS | C | 0.24 | 0.0162 | 0.0028 | 4.30E-09 |
| rs2192527 | Body Fat Percentage | TRANS | G | 0.47 | 0.0182 | 0.0025 | 1.66E-13 |
| rs2239647 | Body Fat Percentage | TRANS | C | 0.54 | -0.0166 | 0.0025 | 2.05E-11 |
| rs2242449 | Body Fat Percentage | TRANS | T | 0.42 | 0.0144 | 0.0025 | 7.22E-09 |
| rs2243928 | Body Fat Percentage | TRANS | G | 0.65 | -0.0177 | 0.0026 | 6.02E-12 |
| rs2281819 | Body Fat Percentage | TRANS | A | 0.27 | -0.0178 | 0.0029 | 1.15E-09 |
| rs2289379 | Body Fat Percentage | TRANS | T | 0.43 | -0.0165 | 0.0025 | 4.82E-11 |
| rs2306937 | Body Fat Percentage | TRANS | T | 0.23 | -0.0191 | 0.0031 | 3.94E-10 |
| rs2318543 | Body Fat Percentage | TRANS | G | 0.79 | -0.0180 | 0.0030 | 1.62E-09 |
| rs2370982 | Body Fat Percentage | TRANS | T | 0.20 | 0.0260 | 0.0030 | 2.95E-18 |
| rs2371767 | Body Fat Percentage | TRANS | C | 0.26 | 0.0178 | 0.0028 | 1.14E-10 |
| rs2398861 | Body Fat Percentage | TRANS | G | 0.31 | 0.0163 | 0.0028 | 7.31E-09 |
| rs241459 | Body Fat Percentage | TRANS | C | 0.70 | -0.0199 | 0.0026 | 3.86E-14 |
| rs2436772 | Body Fat Percentage | TRANS | A | 0.19 | -0.0195 | 0.0030 | 1.11E-10 |
| rs2439823 | Body Fat Percentage | TRANS | G | 0.55 | 0.0189 | 0.0025 | 1.82E-14 |

| rs2455821 | Body Fat Percentage | TRANS | A | 0.25 | 0.0161 | 0.0028 | 5.50E-09 |
| --- | --- | --- | --- | --- | --- | --- | --- |
| rs245775 | Body Fat Percentage | TRANS | G | 0.72 | 0.0194 | 0.0028 | 1.97E-12 |
| rs2477534 | Body Fat Percentage | TRANS | G | 0.63 | -0.0142 | 0.0026 | 3.22E-08 |
| rs249612 | Body Fat Percentage | TRANS | T | 0.74 | 0.0152 | 0.0027 | 2.59E-08 |
| rs254024 | Body Fat Percentage | TRANS | T | 0.42 | 0.0144 | 0.0025 | 5.80E-09 |
| rs2606228 | Body Fat Percentage | TRANS | C | 0.68 | -0.0160 | 0.0026 | 4.87E-10 |
| rs2660241 | Body Fat Percentage | TRANS | C | 0.39 | 0.0153 | 0.0026 | 2.24E-09 |
| rs2665856 | Body Fat Percentage | TRANS | A | 0.69 | -0.0146 | 0.0026 | 2.79E-08 |
| rs2678204 | Body Fat Percentage | TRANS | G | 0.31 | 0.0211 | 0.0026 | 3.68E-16 |
| rs2798297 | Body Fat Percentage | TRANS | A | 0.35 | 0.0151 | 0.0026 | 3.44E-09 |
| rs2802774 | Body Fat Percentage | TRANS | A | 0.51 | 0.0144 | 0.0025 | 4.90E-09 |
| rs28434748 | Body Fat Percentage | TRANS | A | 0.76 | -0.0172 | 0.0031 | 4.31E-08 |
| rs28483178 | Body Fat Percentage | TRANS | G | 0.29 | 0.0240 | 0.0027 | 2.36E-19 |
| rs2855818 | Body Fat Percentage | TRANS | A | 0.24 | 0.0237 | 0.0029 | 2.21E-16 |
| rs28726372 | Body Fat Percentage | TRANS | C | 0.31 | 0.0158 | 0.0027 | 3.04E-09 |
| rs28893270 | Body Fat Percentage | TRANS | A | 0.42 | -0.0152 | 0.0025 | 1.13E-09 |
| rs2914231 | Body Fat Percentage | TRANS | C | 0.24 | -0.0217 | 0.0029 | 8.30E-14 |
| rs2943650 | Body Fat Percentage | TRANS | T | 0.63 | -0.0206 | 0.0026 | 1.22E-15 |
| rs2954033 | Body Fat Percentage | TRANS | G | 0.68 | 0.0157 | 0.0027 | 4.84E-09 |
| rs2980240 | Body Fat Percentage | TRANS | C | 0.80 | 0.0233 | 0.0032 | 3.20E-13 |
| rs2984618 | Body Fat Percentage | TRANS | T | 0.39 | 0.0148 | 0.0025 | 2.70E-09 |
| rs301806 | Body Fat Percentage | TRANS | T | 0.53 | 0.0158 | 0.0025 | 2.20E-10 |
| rs314279 | Body Fat Percentage | TRANS | A | 0.88 | -0.0221 | 0.0038 | 8.46E-09 |
| rs33503 | Body Fat Percentage | TRANS | A | 0.80 | -0.0174 | 0.0031 | 2.04E-08 |
| rs34220678 | Body Fat Percentage | TRANS | A | 0.09 | 0.0228 | 0.0038 | 2.39E-09 |
| rs34308737 | Body Fat Percentage | TRANS | G | 0.10 | 0.0235 | 0.0042 | 2.21E-08 |
| rs34356467 | Body Fat Percentage | TRANS | T | 0.18 | 0.0174 | 0.0032 | 3.96E-08 |
| rs34417222 | Body Fat Percentage | TRANS | T | 0.27 | -0.0168 | 0.0027 | 2.23E-10 |
| rs34483452 | Body Fat Percentage | TRANS | A | 0.16 | 0.0311 | 0.0036 | 4.54E-18 |
| rs34580448 | Body Fat Percentage | TRANS | C | 0.05 | -0.0448 | 0.0062 | 4.32E-13 |
| rs34769775 | Body Fat Percentage | TRANS | T | 0.28 | -0.0178 | 0.0027 | 3.12E-11 |
| rs34811474 | Body Fat Percentage | TRANS | A | 0.22 | -0.0173 | 0.0029 | 3.05E-09 |
| rs34898535 | Body Fat Percentage | TRANS | T | 0.39 | -0.0214 | 0.0025 | 3.34E-17 |
| rs34966008 | Body Fat Percentage | TRANS | T | 0.40 | -0.0169 | 0.0025 | 1.39E-11 |
| rs35060985 | Body Fat Percentage | TRANS | A | 0.35 | 0.0227 | 0.0026 | 7.84E-18 |
| rs35128308 | Body Fat Percentage | TRANS | C | 0.49 | 0.0149 | 0.0025 | 1.47E-09 |
| rs35154152 | Body Fat Percentage | TRANS | C | 0.13 | -0.0228 | 0.0040 | 1.05E-08 |
| rs35276127 | Body Fat Percentage | TRANS | G | 0.17 | -0.0166 | 0.0030 | 4.53E-08 |
| rs35557355 | Body Fat Percentage | TRANS | T | 0.41 | 0.0141 | 0.0025 | 2.43E-08 |
| rs35644221 | Body Fat Percentage | TRANS | A | 0.33 | -0.0167 | 0.0027 | 4.53E-10 |
| rs35761930 | Body Fat Percentage | TRANS | T | 0.22 | 0.0186 | 0.0032 | 4.62E-09 |
| rs35882248 | Body Fat Percentage | TRANS | T | 0.35 | 0.0216 | 0.0026 | 3.12E-16 |
| rs36090025 | Body Fat Percentage | TRANS | C | 0.32 | -0.0147 | 0.0027 | 4.62E-08 |
| rs3736896 | Body Fat Percentage | TRANS | C | 0.19 | 0.0318 | 0.0030 | 1.78E-25 |
| rs3764002 | Body Fat Percentage | TRANS | T | 0.26 | -0.0231 | 0.0028 | 1.36E-16 |
| rs3766823 | Body Fat Percentage | TRANS | A | 0.16 | 0.0212 | 0.0033 | 8.17E-11 |
| rs3796658 | Body Fat Percentage | TRANS | A | 0.49 | -0.0177 | 0.0025 | 7.05E-13 |
| rs3803286 | Body Fat Percentage | TRANS | G | 0.68 | -0.0178 | 0.0026 | 8.42E-12 |
| rs3810291 | Body Fat Percentage | TRANS | A | 0.66 | 0.0177 | 0.0026 | 1.87E-11 |
| rs3817428 | Body Fat Percentage | TRANS | G | 0.24 | -0.0218 | 0.0028 | 4.28E-15 |
| rs3826226 | Body Fat Percentage | TRANS | A | 0.42 | 0.0139 | 0.0025 | 4.77E-08 |
| rs3847072 | Body Fat Percentage | TRANS | A | 0.23 | -0.0156 | 0.0029 | 4.33E-08 |
| rs40071 | Body Fat Percentage | TRANS | C | 0.19 | -0.0212 | 0.0032 | 3.74E-11 |
| rs41310284 | Body Fat Percentage | TRANS | A | 0.10 | -0.0256 | 0.0041 | 3.20E-10 |
| rs4148866 | Body Fat Percentage | TRANS | T | 0.42 | 0.0139 | 0.0025 | 2.48E-08 |
| rs4238585 | Body Fat Percentage | TRANS | T | 0.87 | 0.0211 | 0.0037 | 8.05E-09 |
| rs4284389 | Body Fat Percentage | TRANS | T | 0.48 | 0.0157 | 0.0025 | 1.83E-10 |
| rs4402589 | Body Fat Percentage | TRANS | G | 0.52 | 0.0196 | 0.0025 | 2.38E-15 |

| rs4450871 | Body Fat Percentage | TRANS | G | 0.46 | 0.0144 | 0.0025 | 5.73E-09 |
| --- | --- | --- | --- | --- | --- | --- | --- |
| rs4477562 | Body Fat Percentage | TRANS | T | 0.11 | 0.0218 | 0.0037 | 2.69E-09 |
| rs4482463 | Body Fat Percentage | TRANS | A | 0.92 | -0.0261 | 0.0046 | 1.77E-08 |
| rs4502882 | Body Fat Percentage | TRANS | T | 0.67 | -0.0153 | 0.0026 | 3.30E-09 |
| rs4503172 | Body Fat Percentage | TRANS | T | 0.64 | -0.0147 | 0.0025 | 5.63E-09 |
| rs4547132 | Body Fat Percentage | TRANS | T | 0.32 | -0.0177 | 0.0028 | 2.09E-10 |
| rs4547574 | Body Fat Percentage | TRANS | T | 0.23 | -0.0164 | 0.0029 | 2.40E-08 |
| rs4549080 | Body Fat Percentage | TRANS | T | 0.34 | 0.0173 | 0.0026 | 2.32E-11 |
| rs4549685 | Body Fat Percentage | TRANS | T | 0.34 | -0.0176 | 0.0026 | 1.57E-11 |
| rs4619804 | Body Fat Percentage | TRANS | C | 0.70 | 0.0183 | 0.0028 | 5.80E-11 |
| rs4642249 | Body Fat Percentage | TRANS | G | 0.89 | -0.0240 | 0.0040 | 1.19E-09 |
| rs4657796 | Body Fat Percentage | TRANS | C | 1.00 | -0.0161 | 0.0025 | 2.06E-10 |
| rs4690324 | Body Fat Percentage | TRANS | A | 0.83 | -0.0191 | 0.0032 | 2.64E-09 |
| rs4709745 | Body Fat Percentage | TRANS | C | 0.30 | 0.0147 | 0.0027 | 3.53E-08 |
| rs4718964 | Body Fat Percentage | TRANS | T | 0.41 | 0.0173 | 0.0025 | 4.07E-12 |
| rs4722398 | Body Fat Percentage | TRANS | T | 0.13 | 0.0199 | 0.0036 | 3.03E-08 |
| rs4727630 | Body Fat Percentage | TRANS | G | 0.58 | 0.0139 | 0.0025 | 2.21E-08 |
| rs4752182 | Body Fat Percentage | TRANS | G | 0.56 | -0.0137 | 0.0025 | 3.19E-08 |
| rs4759318 | Body Fat Percentage | TRANS | T | 0.36 | 0.0160 | 0.0026 | 3.52E-10 |
| rs477895 | Body Fat Percentage | TRANS | T | 0.83 | 0.0184 | 0.0033 | 3.13E-08 |
| rs4785955 | Body Fat Percentage | TRANS | T | 0.22 | 0.0168 | 0.0030 | 2.14E-08 |
| rs479018 | Body Fat Percentage | TRANS | A | 0.33 | -0.0193 | 0.0026 | 1.15E-13 |
| rs4790841 | Body Fat Percentage | TRANS | T | 0.15 | -0.0310 | 0.0034 | 7.52E-20 |
| rs4821764 | Body Fat Percentage | TRANS | A | 0.58 | -0.0214 | 0.0025 | 9.42E-18 |
| rs4864201 | Body Fat Percentage | TRANS | C | 0.64 | -0.0149 | 0.0026 | 8.56E-09 |
| rs4876611 | Body Fat Percentage | TRANS | G | 0.76 | 0.0229 | 0.0027 | 4.34E-17 |
| rs4976033 | Body Fat Percentage | TRANS | G | 0.41 | -0.0167 | 0.0025 | 2.72E-11 |
| rs538656 | Body Fat Percentage | TRANS | T | 0.24 | 0.0304 | 0.0029 | 1.13E-25 |
| rs539515 | Body Fat Percentage | TRANS | C | 0.18 | 0.0362 | 0.0030 | 7.41E-33 |
| rs55637757 | Body Fat Percentage | TRANS | T | 0.12 | -0.0225 | 0.0036 | 4.84E-10 |
| rs55714539 | Body Fat Percentage | TRANS | C | 0.35 | 0.0250 | 0.0026 | 4.20E-22 |
| rs56226325 | Body Fat Percentage | TRANS | T | 0.17 | -0.0222 | 0.0034 | 7.50E-11 |
| rs56288810 | Body Fat Percentage | TRANS | G | 0.22 | 0.0179 | 0.0030 | 2.49E-09 |
| rs56399737 | Body Fat Percentage | TRANS | T | 0.48 | -0.0146 | 0.0025 | 3.46E-09 |
| rs56803094 | Body Fat Percentage | TRANS | G | 0.20 | -0.0181 | 0.0029 | 6.32E-10 |
| rs57292329 | Body Fat Percentage | TRANS | A | 0.05 | 0.0286 | 0.0050 | 1.40E-08 |
| rs57866767 | Body Fat Percentage | TRANS | C | 0.46 | -0.0213 | 0.0025 | 8.24E-18 |
| rs58360798 | Body Fat Percentage | TRANS | C | 0.19 | 0.0178 | 0.0031 | 1.34E-08 |
| rs586461 | Body Fat Percentage | TRANS | G | 0.25 | -0.0172 | 0.0029 | 1.91E-09 |
| rs591939 | Body Fat Percentage | TRANS | G | 0.24 | 0.0173 | 0.0028 | 1.03E-09 |
| rs597309 | Body Fat Percentage | TRANS | G | 0.16 | -0.0180 | 0.0033 | 3.11E-08 |
| rs6021948 | Body Fat Percentage | TRANS | A | 0.30 | -0.0166 | 0.0026 | 2.78E-10 |
| rs61778068 | Body Fat Percentage | TRANS | T | 0.03 | -0.0346 | 0.0061 | 1.24E-08 |
| rs61888762 | Body Fat Percentage | TRANS | G | 0.29 | 0.0268 | 0.0026 | 1.92E-24 |
| rs61975142 | Body Fat Percentage | TRANS | A | 0.19 | -0.0215 | 0.0033 | 6.34E-11 |
| rs62037365 | Body Fat Percentage | TRANS | G | 0.33 | 0.0310 | 0.0025 | 3.37E-35 |
| rs62104477 | Body Fat Percentage | TRANS | T | 0.28 | 0.0193 | 0.0026 | 1.66E-13 |
| rs62106258 | Body Fat Percentage | TRANS | C | 0.04 | -0.0654 | 0.0057 | 3.10E-30 |
| rs62261725 | Body Fat Percentage | TRANS | G | 0.35 | -0.0200 | 0.0026 | 2.49E-14 |
| rs62277722 | Body Fat Percentage | TRANS | A | 0.05 | 0.0296 | 0.0051 | 4.67E-09 |
| rs62285233 | Body Fat Percentage | TRANS | A | 0.11 | -0.0234 | 0.0041 | 7.35E-09 |
| rs62414900 | Body Fat Percentage | TRANS | T | 0.04 | -0.0337 | 0.0056 | 1.36E-09 |
| rs62477684 | Body Fat Percentage | TRANS | T | 0.44 | -0.0183 | 0.0025 | 1.81E-13 |
| rs6446187 | Body Fat Percentage | TRANS | A | 0.49 | 0.0240 | 0.0025 | 1.89E-22 |
| rs6545714 | Body Fat Percentage | TRANS | A | 0.62 | -0.0201 | 0.0025 | 1.15E-15 |
| rs6561937 | Body Fat Percentage | TRANS | A | 0.80 | -0.0174 | 0.0028 | 9.19E-10 |
| rs6575340 | Body Fat Percentage | TRANS | A | 0.62 | 0.0182 | 0.0026 | 1.13E-12 |
| rs6686901 | Body Fat Percentage | TRANS | T | 0.53 | -0.0168 | 0.0025 | 1.06E-11 |

| rs6688826 | Body Fat Percentage | TRANS | C | 0.30 | 0.0155 | 0.0027 | 7.73E-09 |
| --- | --- | --- | --- | --- | --- | --- | --- |
| rs6717858 | Body Fat Percentage | TRANS | C | 0.44 | 0.0256 | 0.0025 | 1.31E-24 |
| rs6739303 | Body Fat Percentage | TRANS | T | 0.83 | 0.0340 | 0.0033 | 2.27E-25 |
| rs67807996 | Body Fat Percentage | TRANS | A | 0.36 | 0.0224 | 0.0025 | 3.34E-19 |
| rs6792984 | Body Fat Percentage | TRANS | T | 0.31 | 0.0148 | 0.0027 | 2.75E-08 |
| rs68169458 | Body Fat Percentage | TRANS | C | 0.30 | 0.0165 | 0.0027 | 8.71E-10 |
| rs6847975 | Body Fat Percentage | TRANS | A | 0.37 | 0.0172 | 0.0026 | 2.25E-11 |
| rs6857 | Body Fat Percentage | TRANS | T | 0.17 | -0.0230 | 0.0033 | 1.68E-12 |
| rs6861649 | Body Fat Percentage | TRANS | C | 0.63 | 0.0174 | 0.0025 | 4.66E-12 |
| rs6875585 | Body Fat Percentage | TRANS | C | 0.66 | 0.0148 | 0.0026 | 1.47E-08 |
| rs6888037 | Body Fat Percentage | TRANS | T | 0.79 | 0.0167 | 0.0028 | 4.71E-09 |
| rs6893495 | Body Fat Percentage | TRANS | T | 0.21 | 0.0182 | 0.0030 | 1.23E-09 |
| rs6927268 | Body Fat Percentage | TRANS | G | 0.24 | -0.0187 | 0.0030 | 7.91E-10 |
| rs6948959 | Body Fat Percentage | TRANS | A | 0.75 | -0.0163 | 0.0028 | 7.31E-09 |
| rs6977416 | Body Fat Percentage | TRANS | A | 0.30 | -0.0144 | 0.0026 | 3.82E-08 |
| rs7020 | Body Fat Percentage | TRANS | A | 0.42 | 0.0177 | 0.0025 | 7.63E-13 |
| rs7027304 | Body Fat Percentage | TRANS | T | 0.65 | 0.0142 | 0.0026 | 3.73E-08 |
| rs704061 | Body Fat Percentage | TRANS | C | 0.44 | 0.0194 | 0.0025 | 4.10E-15 |
| rs7046483 | Body Fat Percentage | TRANS | A | 0.61 | -0.0146 | 0.0025 | 6.15E-09 |
| rs7094644 | Body Fat Percentage | TRANS | A | 0.69 | 0.0146 | 0.0026 | 2.46E-08 |
| rs7117842 | Body Fat Percentage | TRANS | C | 0.37 | 0.0146 | 0.0025 | 9.73E-09 |
| rs7124681 | Body Fat Percentage | TRANS | A | 0.42 | 0.0310 | 0.0025 | 2.28E-35 |
| rs7133378 | Body Fat Percentage | TRANS | A | 0.35 | 0.0252 | 0.0026 | 1.44E-21 |
| rs7135617 | Body Fat Percentage | TRANS | G | 0.40 | 0.0158 | 0.0025 | 2.10E-10 |
| rs713586 | Body Fat Percentage | TRANS | C | 0.47 | 0.0319 | 0.0025 | 1.32E-38 |
| rs7139583 | Body Fat Percentage | TRANS | C | 0.27 | -0.0196 | 0.0027 | 4.71E-13 |
| rs7159203 | Body Fat Percentage | TRANS | T | 0.05 | -0.0254 | 0.0047 | 4.99E-08 |
| rs7164727 | Body Fat Percentage | TRANS | T | 0.70 | 0.0197 | 0.0026 | 4.35E-14 |
| rs71658797 | Body Fat Percentage | TRANS | A | 0.09 | 0.0286 | 0.0037 | 1.71E-14 |
| rs7191938 | Body Fat Percentage | TRANS | G | 0.74 | 0.0155 | 0.0027 | 8.79E-09 |
| rs7200589 | Body Fat Percentage | TRANS | A | 0.24 | -0.0174 | 0.0028 | 3.00E-10 |
| rs7206608 | Body Fat Percentage | TRANS | G | 0.31 | 0.0150 | 0.0026 | 1.15E-08 |
| rs7238896 | Body Fat Percentage | TRANS | G | 0.14 | 0.0200 | 0.0035 | 1.52E-08 |
| rs72755233 | Body Fat Percentage | TRANS | A | 0.10 | -0.0212 | 0.0039 | 4.62E-08 |
| rs72820274 | Body Fat Percentage | TRANS | A | 0.47 | 0.0137 | 0.0025 | 3.50E-08 |
| rs72917533 | Body Fat Percentage | TRANS | C | 0.16 | -0.0177 | 0.0032 | 2.00E-08 |
| rs72959041 | Body Fat Percentage | TRANS | A | 0.05 | -0.0343 | 0.0056 | 8.44E-10 |
| rs72976986 | Body Fat Percentage | TRANS | A | 0.18 | -0.0237 | 0.0031 | 3.53E-14 |
| rs72995085 | Body Fat Percentage | TRANS | C | 0.20 | -0.0192 | 0.0032 | 2.24E-09 |
| rs73021485 | Body Fat Percentage | TRANS | T | 0.13 | -0.0221 | 0.0038 | 6.00E-09 |
| rs73041988 | Body Fat Percentage | TRANS | G | 0.16 | -0.0246 | 0.0033 | 9.14E-14 |
| rs73213501 | Body Fat Percentage | TRANS | C | 0.21 | -0.0188 | 0.0033 | 8.80E-09 |
| rs73236524 | Body Fat Percentage | TRANS | A | 0.16 | 0.0203 | 0.0036 | 2.11E-08 |
| rs7412 | Body Fat Percentage | TRANS | T | 0.06 | 0.0274 | 0.0045 | 1.13E-09 |
| rs7442885 | Body Fat Percentage | TRANS | G | 0.24 | -0.0194 | 0.0030 | 1.32E-10 |
| rs7498044 | Body Fat Percentage | TRANS | A | 0.21 | -0.0171 | 0.0030 | 9.67E-09 |
| rs7498798 | Body Fat Percentage | TRANS | A | 0.63 | -0.0148 | 0.0026 | 8.69E-09 |
| rs7503604 | Body Fat Percentage | TRANS | A | 0.49 | -0.0136 | 0.0025 | 3.19E-08 |
| rs7519259 | Body Fat Percentage | TRANS | A | 0.53 | 0.0142 | 0.0025 | 8.27E-09 |
| rs7570258 | Body Fat Percentage | TRANS | C | 0.48 | 0.0136 | 0.0025 | 3.30E-08 |
| rs7571753 | Body Fat Percentage | TRANS | C | 0.42 | -0.0142 | 0.0025 | 2.02E-08 |
| rs75846784 | Body Fat Percentage | TRANS | G | 0.05 | -0.0255 | 0.0046 | 2.22E-08 |
| rs75854315 | Body Fat Percentage | TRANS | A | 0.05 | 0.0289 | 0.0050 | 5.99E-09 |
| rs7591494 | Body Fat Percentage | TRANS | A | 0.26 | 0.0169 | 0.0028 | 1.47E-09 |
| rs7601895 | Body Fat Percentage | TRANS | G | 0.28 | -0.0153 | 0.0027 | 8.23E-09 |
| rs76040172 | Body Fat Percentage | TRANS | A | 0.05 | -0.0389 | 0.0054 | 7.13E-13 |
| rs76095247 | Body Fat Percentage | TRANS | C | 0.24 | -0.0161 | 0.0029 | 2.48E-08 |
| rs7613261 | Body Fat Percentage | TRANS | T | 0.20 | 0.0215 | 0.0030 | 1.54E-12 |

| rs7630228 | Body Fat Percentage | TRANS | C | 0.45 | -0.0148 | 0.0025 | 2.65E-09 |
| --- | --- | --- | --- | --- | --- | --- | --- |
| rs7649970 | Body Fat Percentage | TRANS | T | 0.12 | 0.0395 | 0.0038 | 1.18E-25 |
| rs76824303 | Body Fat Percentage | TRANS | C | 0.11 | -0.0228 | 0.0041 | 3.98E-08 |
| rs7692075 | Body Fat Percentage | TRANS | G | 0.14 | 0.0196 | 0.0034 | 9.45E-09 |
| rs7700107 | Body Fat Percentage | TRANS | C | 0.10 | 0.0196 | 0.0036 | 3.94E-08 |
| rs7774 | Body Fat Percentage | TRANS | A | 0.36 | 0.0149 | 0.0027 | 2.02E-08 |
| rs78265103 | Body Fat Percentage | TRANS | T | 0.06 | -0.0370 | 0.0056 | 3.50E-11 |
| rs78801969 | Body Fat Percentage | TRANS | T | 0.16 | -0.0208 | 0.0035 | 3.66E-09 |
| rs7925100 | Body Fat Percentage | TRANS | A | 0.35 | 0.0154 | 0.0025 | 8.62E-10 |
| rs79478789 | Body Fat Percentage | TRANS | C | 0.16 | 0.0190 | 0.0034 | 1.59E-08 |
| rs7953 | Body Fat Percentage | TRANS | A | 0.51 | 0.0139 | 0.0025 | 1.56E-08 |
| rs7959140 | Body Fat Percentage | TRANS | T | 0.77 | 0.0189 | 0.0029 | 1.41E-10 |
| rs7982447 | Body Fat Percentage | TRANS | C | 0.20 | 0.0211 | 0.0030 | 3.52E-12 |
| rs79869125 | Body Fat Percentage | TRANS | T | 0.11 | -0.0225 | 0.0040 | 1.82E-08 |
| rs79921461 | Body Fat Percentage | TRANS | C | 0.14 | -0.0192 | 0.0035 | 3.13E-08 |
| rs80135947 | Body Fat Percentage | TRANS | C | 0.22 | 0.0307 | 0.0031 | 3.58E-23 |
| rs8042404 | Body Fat Percentage | TRANS | A | 0.29 | 0.0158 | 0.0028 | 1.17E-08 |
| rs8087074 | Body Fat Percentage | TRANS | T | 0.29 | 0.0173 | 0.0028 | 6.00E-10 |
| rs809955 | Body Fat Percentage | TRANS | A | 0.34 | -0.0175 | 0.0025 | 7.66E-12 |
| rs8112818 | Body Fat Percentage | TRANS | G | 0.42 | -0.0175 | 0.0025 | 2.72E-12 |
| rs815163 | Body Fat Percentage | TRANS | C | 0.57 | -0.0140 | 0.0025 | 1.74E-08 |
| rs815611 | Body Fat Percentage | TRANS | A | 0.54 | -0.0152 | 0.0025 | 7.14E-10 |
| rs825501 | Body Fat Percentage | TRANS | C | 0.61 | -0.0158 | 0.0026 | 5.72E-10 |
| rs878206 | Body Fat Percentage | TRANS | G | 0.54 | 0.0136 | 0.0025 | 3.72E-08 |
| rs879620 | Body Fat Percentage | TRANS | T | 0.61 | 0.0218 | 0.0025 | 6.80E-18 |
| rs9320823 | Body Fat Percentage | TRANS | C | 0.59 | 0.0228 | 0.0025 | 8.81E-20 |
| rs9358657 | Body Fat Percentage | TRANS | A | 0.75 | -0.0150 | 0.0027 | 3.87E-08 |
| rs9358912 | Body Fat Percentage | TRANS | T | 0.30 | -0.0320 | 0.0028 | 6.17E-31 |
| rs9394307 | Body Fat Percentage | TRANS | T | 0.34 | 0.0171 | 0.0026 | 5.58E-11 |
| rs9425633 | Body Fat Percentage | TRANS | C | 0.47 | 0.0143 | 0.0025 | 5.39E-09 |
| rs9471333 | Body Fat Percentage | TRANS | T | 0.57 | -0.0218 | 0.0025 | 9.25E-19 |
| rs9522279 | Body Fat Percentage | TRANS | T | 0.43 | 0.0179 | 0.0025 | 5.58E-13 |
| rs9551991 | Body Fat Percentage | TRANS | C | 0.15 | 0.0214 | 0.0035 | 1.02E-09 |
| rs9659073 | Body Fat Percentage | TRANS | A | 0.49 | 0.0152 | 0.0025 | 6.47E-10 |
| rs972283 | Body Fat Percentage | TRANS | G | 0.56 | -0.0190 | 0.0025 | 1.04E-14 |
| rs9747171 | Body Fat Percentage | TRANS | G | 0.60 | 0.0139 | 0.0025 | 3.19E-08 |
| rs9788550 | Body Fat Percentage | TRANS | C | 0.26 | -0.0191 | 0.0028 | 2.13E-11 |
| rs9803921 | Body Fat Percentage | TRANS | A | 0.36 | -0.0140 | 0.0025 | 3.43E-08 |
| rs9865173 | Body Fat Percentage | TRANS | A | 0.72 | -0.0166 | 0.0027 | 6.42E-10 |
| rs9902386 | Body Fat Percentage | TRANS | G | 0.70 | -0.0165 | 0.0026 | 3.73E-10 |
| rs9906944 | Body Fat Percentage | TRANS | T | 0.30 | -0.0180 | 0.0026 | 3.58E-12 |
| rs9948863 | Body Fat Percentage | TRANS | G | 0.48 | 0.0134 | 0.0025 | 4.95E-08 |
| rs10811661 | Fasting Glucose | EUP | T | 0.82 | 0.0238 | 0.0028 | 5.65E-18 |
| rs4869272 | Fasting Glucose | EUP | T | 0.69 | 0.0177 | 0.0022 | 1.02E-15 |
| rs11619319 | Fasting Glucose | EUP | G | 0.23 | 0.0195 | 0.0024 | 1.33E-15 |
| rs983309 | Fasting Glucose | EUP | T | 0.12 | 0.0256 | 0.0033 | 6.29E-15 |
| rs6943153 | Fasting Glucose | EUP | T | 0.34 | 0.0154 | 0.0022 | 1.63E-12 |
| rs11603334 | Fasting Glucose | EUP | G | 0.83 | 0.0192 | 0.0028 | 1.12E-11 |
| rs6113722 | Fasting Glucose | EUP | G | 0.96 | 0.0353 | 0.0053 | 2.49E-11 |
| rs16913693 | Fasting Glucose | EUP | T | 0.97 | 0.0434 | 0.0066 | 3.51E-11 |
| rs3829109 | Fasting Glucose | EUP | G | 0.71 | 0.0172 | 0.0027 | 1.13E-10 |
| rs3783347 | Fasting Glucose | EUP | G | 0.79 | 0.0168 | 0.0026 | 1.32E-10 |
| rs2302593 | Fasting Glucose | EUP | C | 0.50 | 0.0144 | 0.0023 | 9.26E-10 |
| rs9368222 | Fasting Glucose | EUP | A | 0.28 | 0.0143 | 0.0023 | 1.00E-09 |
| rs10747083 | Fasting Glucose | EUP | A | 0.66 | 0.0133 | 0.0023 | 7.57E-09 |
| rs6072275 | Fasting Glucose | EUP | A | 0.16 | 0.0159 | 0.0028 | 1.66E-08 |
| rs7651090 | Fasting Glucose | EUP | G | 0.31 | 0.0128 | 0.0023 | 1.75E-08 |
| rs576674 | Fasting Glucose | EUP | G | 0.15 | 0.0167 | 0.0030 | 2.26E-08 |

| rs11715915 | Fasting Glucose | EUP | C | 0.68 | 0.0120 | 0.0022 | 4.90E-08 |
| --- | --- | --- | --- | --- | --- | --- | --- |
| rs17762454 | Fasting Glucose | EUP | T | 0.26 | 0.0121 | 0.0023 | 1.88E-07 |
| rs7708285 | Fasting Glucose | EUP | G | 0.27 | 0.0114 | 0.0025 | 4.89E-06 |
| rs2657879 | Fasting Glucose | EUP | G | 0.18 | 0.0124 | 0.0027 | 5.69E-06 |
| rs340874 | Fasting Glucose | EUP | C | 0.52 | 0.0135 | 0.0022 | 4.08E-10 |
| rs780094 | Fasting Glucose | EUP | C | 0.61 | 0.0274 | 0.0021 | 2.58E-37 |
| rs560887 | Fasting Glucose | EUP | C | 0.70 | 0.0711 | 0.0025 | 3.93E-19 |
| rs11708067 | Fasting Glucose | EUP | A | 0.79 | 0.0230 | 0.0026 | 1.30E-18 |
| rs11920090 | Fasting Glucose | EUP | T | 0.86 | 0.0264 | 0.0031 | 8.56E-18 |
| rs2191349 | Fasting Glucose | EUP | T | 0.53 | 0.0292 | 0.0021 | 2.71E-20 |
| rs11558471 | Fasting Glucose | EUP | A | 0.68 | 0.0289 | 0.0023 | 7.80E-37 |
| rs7034200 | Fasting Glucose | EUP | A | 0.51 | 0.0134 | 0.0023 | 3.90E-09 |
| rs10814916 | Fasting Glucose | EUP | C | 0.51 | 0.0158 | 0.0022 | 2.26E-13 |
| rs11195502 | Fasting Glucose | EUP | C | 0.91 | 0.0324 | 0.0037 | 1.97E-18 |
| rs10885122 | Fasting Glucose | EUP | G | 0.88 | 0.0274 | 0.0033 | 6.32E-17 |
| rs4506565 | Fasting Glucose | EUP | T | 0.30 | 0.0206 | 0.0023 | 3.95E-19 |
| rs7903146 | Fasting Glucose | EUP | T | 0.28 | 0.0220 | 0.0024 | 2.71E-20 |
| rs11607883 | Fasting Glucose | EUP | G | 0.48 | 0.0213 | 0.0021 | 6.32E-24 |
| rs11605924 | Fasting Glucose | EUP | A | 0.49 | 0.0202 | 0.0023 | 3.93E-19 |
| rs7944584 | Fasting Glucose | EUP | T | 0.73 | 0.0233 | 0.0024 | 4.82E-22 |
| rs174550 | Fasting Glucose | EUP | T | 0.66 | 0.0189 | 0.0022 | 1.34E-17 |
| rs174576 | Fasting Glucose | EUP | C | 0.65 | 0.0197 | 0.0022 | 1.18E-18 |
| rs10830963 | Fasting Glucose | EUP | G | 0.29 | 0.0779 | 0.0025 | 1.88E-07 |
| rs4502156 | Fasting Glucose | EUP | T | 0.55 | 0.0224 | 0.0021 | 1.38E-25 |
| rs10882272 | Vitamin A1 level | Caucasia | C | 0.38 | -0.0875 | 0.0149 | 7.00E-15 |
| rs1667255 | Vitamin A1 level | Caucasia | C | 0.38 | 0.0902 | 0.0153 | 6.00E-14 |
| rs729876 | Periodontitis | EUP | T | 0.79 | 0.2151 | 0.0390 | 9.77E-09 |
| rs16870060 | Periodontitis | EUP | T | 0.09 | 0.3075 | 0.0523 | 3.69E-09 |
| rs4284742 | Periodontitis | EUP | A | 0.24 | 0.2231 | 0.0387 | 2.06E-08 |
| rs2738058 | Periodontitis | EUP | T | 0.43 | 0.2469 | 0.0399 | 6.78E-10 |
| rs1059513 | Serum IgE | American | C | 0.11 | -0.1608 | 0.0229 | 2.00E-12 |
| rs20541 | Serum IgE | American | A | 0.21 | 0.1144 | 0.0131 | 3.00E-18 |
| rs2251746 | Serum IgE | American | C | 0.30 | 0.1725 | 0.0163 | 5.00E-26 |
| rs17106184 | Type2 Diabetes Mellitus | TRANS | G | 0.91 | 0.0953 | 0.0160 | 4.10E-09 |
| rs2867125 | Type2 Diabetes Mellitus | TRANS | C | 0.07 | 0.0611 | 0.0100 | 2.18E-09 |
| rs780094 | Type2 Diabetes Mellitus | TRANS | C | 0.78 | 0.0658 | 0.0100 | 1.00E-10 |
| rs10203174 | Type2 Diabetes Mellitus | TRANS | C | 0.44 | 0.1196 | 0.0170 | 4.30E-12 |
| rs243088 | Type2 Diabetes Mellitus | TRANS | T | 0.79 | 0.0592 | 0.0090 | 6.14E-11 |
| rs11123406 | Type2 Diabetes Mellitus | TRANS | T | 0.58 | 0.0421 | 0.0070 | 8.57E-09 |
| rs13389219 | Type2 Diabetes Mellitus | TRANS | C | 0.36 | 0.0797 | 0.0100 | 2.81E-15 |
| rs2943640 | Type2 Diabetes Mellitus | TRANS | C | 0.23 | 0.0917 | 0.0100 | 1.16E-19 |
| rs1801282 | Type2 Diabetes Mellitus | TRANS | C | 0.48 | 0.1178 | 0.0150 | 6.08E-15 |
| rs1496653 | Type2 Diabetes Mellitus | TRANS | A | 0.83 | 0.0898 | 0.0110 | 1.97E-17 |
| rs17676309 | Type2 Diabetes Mellitus | TRANS | C | 0.55 | 0.0751 | 0.0110 | 4.60E-12 |
| rs11708067 | Type2 Diabetes Mellitus | TRANS | A | 0.15 | 0.1035 | 0.0120 | 1.01E-18 |
| rs4402960 | Type2 Diabetes Mellitus | TRANS | T | 0.27 | 0.1178 | 0.0090 | 1.84E-38 |
| rs6808574 | Type2 Diabetes Mellitus | TRANS | C | 0.57 | 0.0677 | 0.0120 | 5.80E-09 |
| rs4458523 | Type2 Diabetes Mellitus | TRANS | G | 0.73 | 0.0908 | 0.0100 | 7.54E-20 |
| rs2706785 | Type2 Diabetes Mellitus | TRANS | G | 0.78 | 0.1187 | 0.0210 | 2.40E-08 |
| rs6813195 | Type2 Diabetes Mellitus | TRANS | C | 0.85 | 0.0770 | 0.0090 | 4.10E-14 |
| rs702634 | Type2 Diabetes Mellitus | TRANS | A | 0.90 | 0.0583 | 0.0120 | 6.90E-09 |
| rs459193 | Type2 Diabetes Mellitus | TRANS | G | 0.17 | 0.0695 | 0.0110 | 5.60E-11 |
| rs6878122 | Type2 Diabetes Mellitus | TRANS | G | 0.56 | 0.0807 | 0.0120 | 1.78E-11 |
| rs329122 | Type2 Diabetes Mellitus | TRANS | A | 0.94 | 0.0431 | 0.0070 | 3.02E-09 |
| rs9505118 | Type2 Diabetes Mellitus | TRANS | A | 0.88 | 0.0583 | 0.0100 | 1.40E-09 |
| rs7756992 | Type2 Diabetes Mellitus | TRANS | G | 0.29 | 0.1389 | 0.0100 | 7.23E-44 |
| rs3130501 | Type2 Diabetes Mellitus | TRANS | G | 0.95 | 0.0677 | 0.0120 | 4.20E-09 |
| rs622217 | Type2 Diabetes Mellitus | TRANS | T | 0.12 | 0.0545 | 0.0090 | 2.47E-10 |

| rs17168486 | Type2 Diabetes Mellitus | TRANS | T | 0.83 | 0.0825 | 0.0110 | 9.01E-14 |
| --- | --- | --- | --- | --- | --- | --- | --- |
| rs864745 | Type2 Diabetes Mellitus | TRANS | T | 0.45 | 0.0908 | 0.0100 | 2.77E-20 |
| rs9648716 | Type2 Diabetes Mellitus | TRANS | T | 0.05 | 0.0620 | 0.0100 | 1.16E-09 |
| rs12681990 | Type2 Diabetes Mellitus | TRANS | C | 0.83 | 0.0516 | 0.0090 | 3.07E-09 |
| rs516946 | Type2 Diabetes Mellitus | TRANS | C | 0.29 | 0.0788 | 0.0120 | 1.42E-11 |
| rs7845219 | Type2 Diabetes Mellitus | TRANS | T | 0.64 | 0.0564 | 0.0090 | 2.85E-10 |
| rs3802177 | Type2 Diabetes Mellitus | TRANS | G | 0.78 | 0.1240 | 0.0110 | 1.45E-30 |
| rs10758593 | Type2 Diabetes Mellitus | TRANS | A | 0.43 | 0.0649 | 0.0100 | 1.03E-10 |
| rs2383208 | Type2 Diabetes Mellitus | TRANS | A | 0.72 | 0.1740 | 0.0120 | 1.28E-50 |
| rs17791513 | Type2 Diabetes Mellitus | TRANS | A | 0.30 | 0.1089 | 0.0170 | 1.14E-10 |
| rs11257655 | Type2 Diabetes Mellitus | TRANS | T | 0.63 | 0.0843 | 0.0120 | 2.95E-13 |
| rs1802295 | Type2 Diabetes Mellitus | TRANS | A | 0.58 | 0.0677 | 0.0120 | 2.10E-08 |
| rs12571751 | Type2 Diabetes Mellitus | TRANS | A | 0.71 | 0.0779 | 0.0090 | 3.29E-18 |
| rs1111875 | Type2 Diabetes Mellitus | TRANS | C | 0.16 | 0.0998 | 0.0100 | 1.85E-24 |
| rs7903146 | Type2 Diabetes Mellitus | TRANS | T | 0.77 | 0.3045 | 0.0110 | 8.99E-174 |
| rs2237896 | Type2 Diabetes Mellitus | TRANS | G | 0.35 | 0.2175 | 0.0200 | 9.79E-28 |
| rs5215 | Type2 Diabetes Mellitus | TRANS | C | 0.16 | 0.0788 | 0.0090 | 2.44E-18 |
| rs1552224 | Type2 Diabetes Mellitus | TRANS | A | 0.52 | 0.0926 | 0.0140 | 1.92E-11 |
| rs10842994 | Type2 Diabetes Mellitus | TRANS | C | 0.61 | 0.0880 | 0.0130 | 8.04E-12 |
| rs2261181 | Type2 Diabetes Mellitus | TRANS | T | 0.71 | 0.1026 | 0.0150 | 1.60E-12 |
| rs10507349 | Type2 Diabetes Mellitus | TRANS | G | 0.33 | 0.0526 | 0.0090 | 9.69E-10 |
| rs576674 | Type2 Diabetes Mellitus | TRANS | G | 0.69 | 0.0723 | 0.0100 | 9.27E-13 |
| rs1359790 | Type2 Diabetes Mellitus | TRANS | G | 0.70 | 0.0649 | 0.0110 | 6.89E-10 |
| rs7985179 | Type2 Diabetes Mellitus | TRANS | T | 0.90 | 0.0695 | 0.0120 | 4.16E-09 |
| rs4502156 | Type2 Diabetes Mellitus | TRANS | T | 0.28 | 0.0554 | 0.0100 | 2.44E-08 |
| rs7177055 | Type2 Diabetes Mellitus | TRANS | A | 0.59 | 0.0807 | 0.0100 | 4.34E-15 |
| rs9940149 | Type2 Diabetes Mellitus | TRANS | G | 0.47 | 0.0526 | 0.0090 | 1.09E-09 |
| rs9936385 | Type2 Diabetes Mellitus | TRANS | C | 0.32 | 0.1115 | 0.0100 | 7.46E-30 |
| rs7202877 | Type2 Diabetes Mellitus | TRANS | T | 0.72 | 0.0871 | 0.0160 | 2.76E-08 |
| rs2925979 | Type2 Diabetes Mellitus | TRANS | T | 0.48 | 0.0459 | 0.0080 | 2.41E-08 |
| rs11651755 | Type2 Diabetes Mellitus | TRANS | C | 0.58 | 0.0935 | 0.0130 | 4.12E-13 |
| rs12970134 | Type2 Diabetes Mellitus | TRANS | A | 0.15 | 0.0770 | 0.0110 | 5.19E-13 |
| rs10401969 | Type2 Diabetes Mellitus | TRANS | C | 0.41 | 0.0971 | 0.0170 | 1.68E-08 |
| rs4812829 | Type2 Diabetes Mellitus | TRANS | A | 0.83 | 0.0723 | 0.0120 | 1.87E-09 |

RA: risk allele, EAF: effect allele frequency

|  | | | | |
| --- | --- | --- | --- | --- |
| **Chr:Pos** | **Closest Gene** | **F** | **Sample size** | **Variance Explained** |
| 7:44198411 | GCK | 43.26 | 131010 | 3.30E-04 |
| 8:9239197 | PPP1R3B | 35.64 | 131010 | 2.72E-04 |
| 5:96280573 | ERAP2 | 33.19 | 131010 | 2.53E-04 |
| 3:124565088 | ADCY5 | 44.84 | 131010 | 3.42E-04 |
| 3:124577141 | ADCY5 | 31.69 | 131010 | 2.42E-04 |
| 10:114798892 | TCF7L2 | 48.89 | 131010 | 3.73E-04 |
| 19:50872024 | GIPR | 67.38 | 131010 | 5.14E-04 |
| 1:246444082 | SMYD3 | 8.69 | 10995 | 7.90E-04 |
| 5:100184647 | ST8SIA4 | 44.95 | 10995 | 4.07E-03 |
| 7:73566677 | LAT2 | 87.86 | 10995 | 7.93E-03 |
| 17:43456728 | ARHGAP27 | 39.57 | 10995 | 3.59E-03 |
| 17:73341284 | GRB2 | 50.67 | 10995 | 4.59E-03 |
| 2:114085785 | PAX8, LOC100130100 | 220.82 | 446118 | 4.95E-04 |
| 2:58871658 | VRK2, LINC01122 | 79.52 | 446118 | 1.78E-04 |
| 18:53059748 | TCF4 | 60.27 | 446118 | 1.35E-04 |
| 1:34731984 | CSMD2, C1orf94, GJB | 59.63 | 446118 | 1.34E-04 |
| 16:53800629 | FTO | 54.79 | 446118 | 1.23E-04 |
| 4:102896591 | BANK1 | 50.09 | 446118 | 1.12E-04 |
| 16:56120461 | GNAO1 | 49.9 | 446118 | 1.12E-04 |
| 5:102321905 | PAM, PPIP5K2, GIN1, | 49.88 | 446118 | 1.12E-04 |
| 3:135838598 | PPP2R3A, PCCB, STA | 45.87 | 446118 | 1.03E-04 |
| 7:114126432 | FOXP2 | 46.75 | 446118 | 1.05E-04 |
| 6:28584775 | ZBED9, SCAND3, LO | 44.94 | 446118 | 1.01E-04 |
| 16:6550400 | RBFOX1 | 44.35 | 446118 | 9.94E-05 |
| 7:2106928 | MAD1L1 | 43.59 | 446118 | 9.77E-05 |
| 8:14279446 | SGCZ | 43.21 | 446118 | 9.68E-05 |
| 9:37100525 | EBLN3, ZCCHC7 | 42.8 | 446118 | 9.59E-05 |
| 17:21313223 | KCNJ12 | 41.63 | 446118 | 9.33E-05 |
| 11:43800474 | HSD17B12 | 41.84 | 446118 | 9.38E-05 |
| 5:1428883 | SLC6A3 | 41.25 | 446118 | 9.25E-05 |
| 11:28829882 | METT5D1, OR2BH1P | 40.75 | 446118 | 9.13E-05 |
| 10:125016501 | BUB3, LOC100131719 | 40.66 | 446118 | 9.11E-05 |
| 8:9149746 | PPP1R3B, LOC100129 | 40.38 | 446118 | 9.05E-05 |
| 15:47989799 | SEMA6D | 39.27 | 446118 | 8.80E-05 |
| 5:3126584 | LINC01377, LOC1001 | 39.92 | 446118 | 8.95E-05 |
| 6:54937974 | FAM83B | 38.8 | 446118 | 8.70E-05 |
| 12:117951150 | KSR2 | 38.32 | 446118 | 8.59E-05 |
| 2:166944004 | SCN1A, GALNT3, TT | 38.36 | 446118 | 8.60E-05 |
| 17:44083402 | MAPT, STH, KANSL1 | 37.01 | 446118 | 8.29E-05 |
| 11:122830251 | BSX, C11orf63 | 36.6 | 446118 | 8.20E-05 |
| 2:147612734 | PABPCP2, LOC10013 | 37.27 | 446118 | 8.35E-05 |
| 1:66476437 | PDE4B | 36.28 | 446118 | 8.13E-05 |
| 14:29816155 | MIR548AI, LOC10012 | 36.66 | 446118 | 8.22E-05 |
| 11:113408518 | DRD2 | 37.16 | 446118 | 8.33E-05 |
| 1:57864304 | DAB1 | 35.43 | 446118 | 7.94E-05 |
| 3:55879269 | ERC2 | 36.38 | 446118 | 8.15E-05 |
| 11:48162453 | PTPRJ, OR4X2, OR4B | 35.45 | 446118 | 7.95E-05 |
| 4:18327896 | LCORL, LOC645174 | 34.67 | 446118 | 7.77E-05 |
| 10:21830580 | MLLT10, DNAJC1, S | K34.69 | 446118 | 7.78E-05 |
| 17:11227352 | SHISA6 | 35.35 | 446118 | 7.92E-05 |
| 1:98527951 | DPYD | 34.1 | 446118 | 7.64E-05 |
| 3:137031237 | IL20RB, NPM1P17 | 33.81 | 446118 | 7.58E-05 |
| 14:26954078 | NOVA1 | 34.72 | 446118 | 7.78E-05 |
| 9:140497072 | ARRDC1, MRPL41, E | 34.28 | 446118 | 7.68E-05 |
| 14:78495761 | ADCK1, FRDAP | 33.51 | 446118 | 7.51E-05 |
| 19:9942262 | FBXL12, UBL5, PIN1 | 33.58 | 446118 | 7.53E-05 |

| 20:43538733 | YWHAB, PABPC1L | 33.62 | 446118 | 7.54E-05 |
| --- | --- | --- | --- | --- |
| 6:43160375 | CUL9, MEA1, PPP2R5 | 34.75 | 446118 | 7.79E-05 |
| 2:40382712 | SLC8A1 | 32.22 | 446118 | 7.22E-05 |
| 5:176751059 | LMAN2, FGFR4, SLC | 32.6 | 446118 | 7.31E-05 |
| 2:9185564 | MBOAT2 | 32.59 | 446118 | 7.30E-05 |
| 17:8134275 | PER1, PFAS, SMAD5- | 33.11 | 446118 | 7.42E-05 |
| 11:101520886 | TRPC6 | 32.91 | 446118 | 7.38E-05 |
| 11:118358027 | KMT2A, ARCN1, IFT | 31.98 | 446118 | 7.17E-05 |
| 3:70470834 | FOXP1, LOC1001281 | 31.94 | 446118 | 7.16E-05 |
| 14:60233841 | RTN1 | 33.19 | 446118 | 7.44E-05 |
| 4:82254908 | PRKG2 | 32.27 | 446118 | 7.23E-05 |
| 7:132610266 | CHCHD3 | 31.72 | 446118 | 7.11E-05 |
| 4:92533225 | CCSER1 | 31.87 | 446118 | 7.14E-05 |
| 3:107564459 | BBX, LOC285205 | 32.23 | 446118 | 7.22E-05 |
| 6:93162639 | LOC100129847, LOC1 | 32.08 | 446118 | 7.19E-05 |
| 11:80685181 | LOC729790, LOC6461 | 31.19 | 446118 | 6.99E-05 |
| 11:116576415 | BUD13 | 31.61 | 446118 | 7.09E-05 |
| 14:65554638 | MAX, FNTB, GPX2, C | 30.75 | 446118 | 6.89E-05 |
| 5:137654218 | CDC25C, EGR1, ETF1 | 31.38 | 446118 | 7.03E-05 |
| 5:135615615 | TRPC7, SMAD5, SMA | 30.75 | 446118 | 6.89E-05 |
| 11:88297740 | GRM5 | 31.46 | 446118 | 7.05E-05 |
| 17:50571227 | CA10, LOC339209 | 31.58 | 446118 | 7.08E-05 |
| 12:110007939 | MMAB, MVK, KCTD | 30.81 | 446118 | 6.91E-05 |
| 6:89790201 | PNRC1 | 30.66 | 446118 | 6.87E-05 |
| 11:61581764 | FADS1, FADS3, RAB | 31.08 | 446118 | 6.97E-05 |
| 2:210377845 | MAP2 | 30.78 | 446118 | 6.90E-05 |
| 2:59358659 | LINC01122, LOC1019 | 31.01 | 446118 | 6.95E-05 |
| 2:139195328 | SPOPL | 30.79 | 446118 | 6.90E-05 |
| 16:23909538 | PRKCB | 31 | 446118 | 6.95E-05 |
| 2:157040773 | NR4A2, GPD2 | 29.93 | 446118 | 6.71E-05 |
| 12:38764559 | ALG10B, CPNE8 | 30.25 | 446118 | 6.78E-05 |
| 6:33464363 | ZBTB9, KIFC1, PHF1, | 30.13 | 446118 | 6.75E-05 |
| 10:64618340 | EGR2, ADO | 29.61 | 446118 | 6.64E-05 |
| 10:103128332 | BTRC, FGF8, NFKB2, | 29.69 | 446118 | 6.65E-05 |
| 4:72828262 | GC | 1497.35 | 79366 | 1.85E-02 |
| 11:70845097 | NADSYN1/ DHCR7 | 267.77 | 79366 | 3.36E-03 |
| 11:14871454 | CYP2R1 | 198.55 | 79366 | 2.50E-03 |
| 20:52165769 | CYP24A1 | 92.73 | 79366 | 1.17E-03 |
| 12:94882660 | AMDHD1 | 59.71 | 79366 | 7.52E-04 |
| 14:38625936 | SEC23A | 34.36 | 79366 | 4.33E-04 |
| 11:116154127 | BUD13/ZNF259/APO | 16 | 5006 | 3.19E-03 |
| 19:15851431 | CYP4F2 | 9 | 5006 | 1.80E-03 |
| 12:123873006 | SCARB1 | 9 | 5006 | 1.80E-03 |
| 1：35591626 | Intergenic | 36 | 335394 | 1.07E-04 |
| 1：154548521 | Utr3:CHRNB2 | 53.54 | 335394 | 1.60E-04 |
| 1：175993820 | Intron:RFWD2 | 33.73 | 335394 | 1.01E-04 |
| 2：60718347 | Intron:BCL11A | 31.81 | 335394 | 9.48E-05 |
| 2：62710608 | Intergenic | 30.25 | 335394 | 9.02E-05 |
| 2：148372720 | Intergenic | 51.84 | 335394 | 1.55E-04 |
| 3：16872929 | Intergenic | 38.34 | 335394 | 1.14E-04 |
| 3：48935583 | Intron:SLC25A20 | 49.54 | 335394 | 1.48E-04 |
| 3：50421081 | Intron:CACNA2D2 | 32.87 | 335394 | 9.80E-05 |
| 3：136224697 | Intron:STAG1 | 33.06 | 335394 | 9.86E-05 |
| 3：158083918 | Intron:RSRC1 | 41.71 | 335394 | 1.24E-04 |
| 4：3006043 | Nonsynonymous:GRK | 33.18 | 335394 | 9.89E-05 |
| 4：67086288 | Intergenic | 40.58 | 335394 | 1.21E-04 |
| 4：67980830 | Intergenic | 31.84 | 335394 | 9.49E-05 |
| 6：26184102 | Nonsynonymous:HIST | 41.05 | 335394 | 1.22E-04 |

| 7：32333642 | Intron:PDE1C | 92.16 | 335394 | 2.75E-04 |
| --- | --- | --- | --- | --- |
| 7：50339609 | Intergenic | 32.11 | 335394 | 9.57E-05 |
| 8：27418040 | Intergenic | 33.42 | 335394 | 9.96E-05 |
| 8：27442127 | Intergenic | 45.13 | 335394 | 1.35E-04 |
| 8：42442018 | Intergenic | 30.47 | 335394 | 9.08E-05 |
| 8：42578059 | Intron:CHRNB3 | 139.89 | 335394 | 4.17E-04 |
| 8：64604218 | Intergenic | 42.49 | 335394 | 1.27E-04 |
| 9：136468701 | Intergenic | 84.26 | 335394 | 2.51E-04 |
| 9：136502369 | Intron:DBH | 102.6 | 335394 | 3.06E-04 |
| 11：16377044 | Intron:SOX6 | 46.87 | 335394 | 1.40E-04 |
| 11：43667625 | Intergenic | 29.16 | 335394 | 8.69E-05 |
| 11：46737412 | Intergenic | 36.99 | 335394 | 1.10E-04 |
| 11：113436072 | Intergenic | 45.7 | 335394 | 1.36E-04 |
| 14：104184737 | Intron:ZFYVE21 | 34.18 | 335394 | 1.02E-04 |
| 15：57141231 | Intergenic | 35.6 | 335394 | 1.06E-04 |
| 15：59155050 | Intergenic | 40.41 | 335394 | 1.20E-04 |
| 15：78243579 | Intergenic | 34.26 | 335394 | 1.02E-04 |
| 15：78812098 | Intron:HYKK | 38.03 | 335394 | 1.13E-04 |
| 15：78814046 | Intron:HYKK | 1296 | 335394 | 3.85E-03 |
| 15：78836288 | Intron:PSMA4 | 49.56 | 335394 | 1.48E-04 |
| 15：78849779 | Intergenic | 149.59 | 335394 | 4.46E-04 |
| 15：78906177 | Intron:CHRNA3 | 72.25 | 335394 | 2.15E-04 |
| 15：78908565 | Intron:CHRNA3 | 263.52 | 335394 | 7.85E-04 |
| 15：78911181 | Synonymous:CHRNA3 | 40.96 | 335394 | 1.22E-04 |
| 15：79065171 | Intron:ADAMTS7 | 44.62 | 335394 | 1.33E-04 |
| 15：89943601 | Intergenic | 36 | 335394 | 1.07E-04 |
| 16：52093549 | Intron:C16orf97 | 41.99 | 335394 | 1.25E-04 |
| 16：69552215 | Intergenic | 31.17 | 335394 | 9.29E-05 |
| 16：89756473 | Intron:CDK10 | 39.94 | 335394 | 1.19E-04 |
| 18：53251725 | Intron:TCF4 | 31.77 | 335394 | 9.47E-05 |
| 18：62125063 | Intergenic | 37.45 | 335394 | 1.12E-04 |
| 19：4044424 | Utr3:ZBTB7A | 41.04 | 335394 | 1.22E-04 |
| 19：41338847 | Intergenic | 118.78 | 335394 | 3.54E-04 |
| 19：41353107 | Intron:CYP2A6 | 437.65 | 335394 | 1.30E-03 |
| 19：41354496 | Intron:CYP2A6 | 64.33 | 335394 | 1.92E-04 |
| 19：41371480 | Intergenic | 153.18 | 335394 | 4.57E-04 |
| 20：11863500 | Intergenic | 41.47 | 335394 | 1.24E-04 |
| 20：31054702 | Intron:NOL4L | 45.7 | 335394 | 1.36E-04 |
| 20：61986949 | Intron:CHRNA4 | 114.62 | 335394 | 3.42E-04 |
| 21：40520783 | Intergenic | 30.03 | 335394 | 8.95E-05 |
| 2：23953454 | Intergenic | 35.57 | 547219 | 6.50E-05 |
| 3：49210732 | Intron:KLHDC8B | 49 | 547219 | 8.95E-05 |
| 6：396321 | Intron:IRF4 | 32.78 | 547219 | 5.99E-05 |
| 6：35058117 | Utr3:ANKS1A | 30.78 | 547219 | 5.62E-05 |
| 7：32314690 | Intron:PDE1C | 33.06 | 547219 | 6.04E-05 |
| 8：27426077 | Intergenic | 49.86 | 547219 | 9.11E-05 |
| 8：42602668 | Intergenic | 31.02 | 547219 | 5.67E-05 |
| 9：127917257 | Intron:PPP6C | 41.5 | 547219 | 7.58E-05 |
| 9：136461851 | Intergenic | 38.86 | 547219 | 7.10E-05 |
| 9：136467344 | Intergenic | 155.66 | 547219 | 2.84E-04 |
| 9：136473572 | Intergenic | 45.13 | 547219 | 8.25E-05 |
| 9：136509275 | Intron:DBH | 33.73 | 547219 | 6.16E-05 |
| 11：16372431 | Intron:SOX6 | 40.79 | 547219 | 7.45E-05 |
| 15：47647755 | Intron:SEMA6D | 36.91 | 547219 | 6.74E-05 |
| 15：76629609 | Intron:ISL2 | 32.4 | 547219 | 5.92E-05 |
| 15：78883813 | Intron:CHRNA5 | 50.31 | 547219 | 9.19E-05 |
| 19：41342842 | Intergenic | 51.34 | 547219 | 9.38E-05 |
| 19：41353107 | Intron:CYP2A6 | 218.13 | 547219 | 3.98E-04 |

| 19：41371480 | Intergenic | 51.1 | 547219 | 9.34E-05 |
| --- | --- | --- | --- | --- |
| 19：41375030 | Intergenic | 43.42 | 547219 | 7.93E-05 |
| 20：61984317 | Intron:CHRNA4 | 100 | 547219 | 1.83E-04 |
| 20：61986950 | Intron:CHRNA4 | 36 | 547219 | 6.58E-05 |
| 20：62018289 | Intergenic | 47.65 | 547219 | 8.71E-05 |
| 22：41854446 | Intergenic | 44.95 | 547219 | 8.21E-05 |
| 1：7791461 | Intron:CAMTA1 | 44.44 | 1232091 | 3.61E-05 |
| 1：8484823 | Intron:LOC102724552 | 47.93 | 1232091 | 3.89E-05 |
| 1：18436657 | Intron:IGSF21 | 52.28 | 1232091 | 4.24E-05 |
| 1：32195819 | Intron:ADGRB2 | 34.13 | 1232091 | 2.77E-05 |
| 1：33795572 | Intron:PHC2 | 34.24 | 1232091 | 2.78E-05 |
| 1：38757237 | Intergenic | 42.25 | 1232091 | 3.43E-05 |
| 1：41776623 | Intergenic | 32.53 | 1232091 | 2.64E-05 |
| 1：44011737 | Intron:PTPRF | 128.74 | 1232091 | 1.04E-04 |
| 1：46496709 | Synonymous:MAST2 | 31.04 | 1232091 | 2.52E-05 |
| 1：50603995 | Intron:ELAVL4 | 70.14 | 1232091 | 5.69E-05 |
| 1：50861071 | Intergenic | 32.97 | 1232091 | 2.68E-05 |
| 1：58815243 | Intergenic | 31.81 | 1232091 | 2.58E-05 |
| 1：66469643 | Intron:PDE4B | 61.56 | 1232091 | 5.00E-05 |
| 1：71490122 | Intron:PTGER3 | 41.53 | 1232091 | 3.37E-05 |
| 1：72752073 | Intergenic | 74.59 | 1232091 | 6.05E-05 |
| 1：72900406 | Intergenic | 34.55 | 1232091 | 2.80E-05 |
| 1：73824909 | Intergenic | 81 | 1232091 | 6.57E-05 |
| 1：74991596 | Intron:FPGT-TNNI3K| | 55.1 | 1232091 | 4.47E-05 |
| 1：76689019 | Intron:ST6GALNAC3 | 31.87 | 1232091 | 2.59E-05 |
| 1：87913176 | Intergenic | 35.54 | 1232091 | 2.88E-05 |
| 1：91189731 | Intergenic | 76.9 | 1232091 | 6.24E-05 |
| 1：96414335 | Intergenic | 36.46 | 1232091 | 2.96E-05 |
| 1：154206358 | Intron:UBAP2L | 39.14 | 1232091 | 3.18E-05 |
| 1：155034632 | Intron:ADAM15|DCST | 40.38 | 1232091 | 3.28E-05 |
| 1：174104743 | Intron:LOC102724601 | 44.27 | 1232091 | 3.59E-05 |
| 1：179783167 | Nonsynonymous:FAM | 40.41 | 1232091 | 3.28E-05 |
| 1：190957480 | Intergenic | 30.06 | 1232091 | 2.44E-05 |
| 1：210302043 | Intron:SYT14 | 47.21 | 1232091 | 3.83E-05 |
| 1：236872829 | Intron:ACTN2 | 40.76 | 1232091 | 3.31E-05 |
| 1：237852083 | Intron:RYR2 | 43.95 | 1232091 | 3.57E-05 |
| 2：264621 | Intron:SH3YL1 | 32.96 | 1232091 | 2.67E-05 |
| 2：417167 | Intergenic | 57.51 | 1232091 | 4.67E-05 |
| 2：624205 | Intergenic | 86.38 | 1232091 | 7.01E-05 |
| 2：22067213 | Intergenic | 31.97 | 1232091 | 2.59E-05 |
| 2：22582968 | Intergenic | 44.79 | 1232091 | 3.63E-05 |
| 2：29513404 | Intron:ALK | 34.92 | 1232091 | 2.83E-05 |
| 2：32808804 | Intron:BIRC6 | 46.87 | 1232091 | 3.80E-05 |
| 2：44250149 | Intergenic | 36.93 | 1232091 | 3.00E-05 |
| 2：45159091 | Intron:LINC01833 | 119.31 | 1232091 | 9.68E-05 |
| 2：50735943 | Intron:NRXN1 | 34.57 | 1232091 | 2.81E-05 |
| 2：51341259 | Intron:LOC730100 | 43.76 | 1232091 | 3.55E-05 |
| 2：58171220 | Intron:VRK2 | 42.25 | 1232091 | 3.43E-05 |
| 2：59022210 | Intron:LINC01122 | 30.62 | 1232091 | 2.48E-05 |
| 2：59315828 | Intergenic | 31.53 | 1232091 | 2.56E-05 |
| 2：59819545 | Intergenic | 38.82 | 1232091 | 3.15E-05 |
| 2：60024857 | Intergenic | 61.56 | 1232091 | 5.00E-05 |
| 2：60139524 | Intergenic | 34.63 | 1232091 | 2.81E-05 |
| 2：60477052 | Intergenic | 66.39 | 1232091 | 5.39E-05 |
| 2：63416606 | Intron:WDPCP | 39.57 | 1232091 | 3.21E-05 |
| 2：80748807 | Intron:CTNNA2 | 31.53 | 1232091 | 2.56E-05 |
| 2：80999398 | Intergenic | 35.08 | 1232091 | 2.85E-05 |
| 2：83247997 | Intergenic | 30.67 | 1232091 | 2.49E-05 |

| 2：98275354 | Nonsynonymous:ACT | R29.77 | 1232091 | 2.42E-05 |
| --- | --- | --- | --- | --- |
| 2：100672408 | Intron:AFF3 | 40 | 1232091 | 3.25E-05 |
| 2：104126983 | Intergenic | 136.71 | 1232091 | 1.11E-04 |
| 2：104432494 | Intergenic | 30.54 | 1232091 | 2.48E-05 |
| 2：113240183 | Intron:TTL | 46.53 | 1232091 | 3.78E-05 |
| 2：113246436 | Intron:TTL | 32.11 | 1232091 | 2.61E-05 |
| 2：137571174 | Intron:THSD7B | 41.82 | 1232091 | 3.39E-05 |
| 2：145412271 | Intergenic | 63.43 | 1232091 | 5.15E-05 |
| 2：146140132 | Intergenic | 170 | 1232091 | 1.38E-04 |
| 2：146283610 | Intergenic | 65.15 | 1232091 | 5.29E-05 |
| 2：147825689 | Intergenic | 32.4 | 1232091 | 2.63E-05 |
| 2：155682556 | Intron:KCNJ3 | 62.78 | 1232091 | 5.09E-05 |
| 2：161816880 | Intergenic | 30.82 | 1232091 | 2.50E-05 |
| 2：162101261 | Exon:LINC01806 | 46.34 | 1232091 | 3.76E-05 |
| 2：162802993 | Intron:SLC4A10 | 86.63 | 1232091 | 7.03E-05 |
| 2：164862639 | Intergenic | 33.16 | 1232091 | 2.69E-05 |
| 2：166250244 | Intergenic | 31.87 | 1232091 | 2.59E-05 |
| 2：172521827 | Intergenic | 31.28 | 1232091 | 2.54E-05 |
| 2：182027603 | Intron:LINC01934 | 47.09 | 1232091 | 3.82E-05 |
| 2：199523122 | Intergenic | 38.44 | 1232091 | 3.12E-05 |
| 2：200936399 | Intergenic | 29.83 | 1232091 | 2.42E-05 |
| 2：202843875 | Intergenic | 32.11 | 1232091 | 2.61E-05 |
| 2：225365635 | Intron:CUL3 | 44.44 | 1232091 | 3.61E-05 |
| 2：226332033 | Intron:NYAP2 | 56.25 | 1232091 | 4.57E-05 |
| 3：2365026 | Intron:CNTN4 | 30.25 | 1232091 | 2.46E-05 |
| 3：16851202 | Intergenic | 43.76 | 1232091 | 3.55E-05 |
| 3：25725501 | Intergenic | 39.98 | 1232091 | 3.24E-05 |
| 3：47800216 | Intron:SMARCC1 | 34.73 | 1232091 | 2.82E-05 |
| 3：48731487 | Utr3:IP6K2 | 52.93 | 1232091 | 4.30E-05 |
| 3：50192760 | Intron:SEMA3F|SEMA | 57.65 | 1232091 | 4.68E-05 |
| 3：52886605 | Intron:STIMATE|TME | 39.69 | 1232091 | 3.22E-05 |
| 3：53766212 | Intron:CACNA1D | 41.26 | 1232091 | 3.35E-05 |
| 3：55988394 | Intron:ERC2 | 33.85 | 1232091 | 2.75E-05 |
| 3：59434420 | Intergenic | 31.53 | 1232091 | 2.56E-05 |
| 3：59966156 | Intron:FHIT | 29.83 | 1232091 | 2.42E-05 |
| 3：60459291 | Intron:FHIT | 32.4 | 1232091 | 2.63E-05 |
| 3：64234307 | Intergenic | 32.84 | 1232091 | 2.67E-05 |
| 3：70890288 | Intergenic | 45.08 | 1232091 | 3.66E-05 |
| 3：71064431 | Intron:FOXP1 | 61.56 | 1232091 | 5.00E-05 |
| 3：71483084 | Intron:FOXP1 | 36 | 1232091 | 2.92E-05 |
| 3：74954560 | Intergenic | 45.26 | 1232091 | 3.67E-05 |
| 3：77176032 | Intron:ROBO2 | 31.97 | 1232091 | 2.59E-05 |
| 3：81325861 | Intergenic | 36.78 | 1232091 | 2.98E-05 |
| 3：83241365 | Intergenic | 48.58 | 1232091 | 3.94E-05 |
| 3：85460131 | Intron:CADM2 | 88.79 | 1232091 | 7.21E-05 |
| 3：85475292 | Intron:CADM2 | 50.51 | 1232091 | 4.10E-05 |
| 3：85902536 | Intron:CADM2 | 51.63 | 1232091 | 4.19E-05 |
| 3：107997514 | Intergenic | 29.83 | 1232091 | 2.42E-05 |
| 3：114147927 | Intron:ZBTB20 | 36 | 1232091 | 2.92E-05 |
| 3：117822149 | Intergenic | 56.02 | 1232091 | 4.55E-05 |
| 3：118302515 | Intron:LOC105374060 | 36 | 1232091 | 2.92E-05 |
| 3：131945722 | Intergenic | 29.41 | 1232091 | 2.39E-05 |
| 3：147106319 | Utr3:ZIC4 | 31.5 | 1232091 | 2.56E-05 |
| 3：147719648 | Intergenic | 41.53 | 1232091 | 3.37E-05 |
| 3：149543102 | Intron:RNF13 | 30.25 | 1232091 | 2.46E-05 |
| 3：157393770 | Intergenic | 30.86 | 1232091 | 2.50E-05 |
| 3：158284861 | Intron:LOC100996447 | 39.3 | 1232091 | 3.19E-05 |
| 3：159048333 | Intron:IQCJ-SCHIP1|S | 35.08 | 1232091 | 2.85E-05 |

| 3：161761866 | Intergenic | 31.77 | 1232091 | 2.58E-05 |
| --- | --- | --- | --- | --- |
| 3：173072584 | Intergenic | 30.25 | 1232091 | 2.46E-05 |
| 3：173353739 | Intron:NLGN1 | 32 | 1232091 | 2.60E-05 |
| 3：175718927 | Intergenic | 31.59 | 1232091 | 2.56E-05 |
| 3：181409057 | Intron:SOX2-OT | 42.25 | 1232091 | 3.43E-05 |
| 4：15458598 | Intergenic | 28.99 | 1232091 | 2.35E-05 |
| 4：28027176 | Intergenic | 30.62 | 1232091 | 2.48E-05 |
| 4：28246049 | Intergenic | 52.68 | 1232091 | 4.28E-05 |
| 4：28822284 | Intergenic | 34.03 | 1232091 | 2.76E-05 |
| 4：29082156 | Intergenic | 36.83 | 1232091 | 2.99E-05 |
| 4：31184484 | Intron:LINC02497 | 55.97 | 1232091 | 4.54E-05 |
| 4：35501032 | Intergenic | 53.58 | 1232091 | 4.35E-05 |
| 4：35514712 | Intergenic | 46.53 | 1232091 | 3.78E-05 |
| 4：68017710 | Intergenic | 58.93 | 1232091 | 4.78E-05 |
| 4：94052854 | Intron:GRID2 | 34.63 | 1232091 | 2.81E-05 |
| 4：94550450 | Intron:GRID2 | 34.31 | 1232091 | 2.78E-05 |
| 4：112467612 | Intergenic | 41.66 | 1232091 | 3.38E-05 |
| 4：136406155 | Intergenic | 30.94 | 1232091 | 2.51E-05 |
| 4：137474783 | Intergenic | 34.63 | 1232091 | 2.81E-05 |
| 4：140886963 | Intron:MAML3 | 67.6 | 1232091 | 5.49E-05 |
| 4：143510148 | Intron:INPP4B | 31.59 | 1232091 | 2.56E-05 |
| 4：147797913 | Intron:TTC29 | 89.52 | 1232091 | 7.27E-05 |
| 4：147985231 | Intergenic | 37.35 | 1232091 | 3.03E-05 |
| 4：176875795 | Intron:GPM6A | 29.57 | 1232091 | 2.40E-05 |
| 5：30842054 | Intergenic | 35.08 | 1232091 | 2.85E-05 |
| 5：43190647 | Intergenic | 43.37 | 1232091 | 3.52E-05 |
| 5：50821338 | Intergenic | 37.87 | 1232091 | 3.07E-05 |
| 5：60121271 | Intron:ELOVL7 | 50.95 | 1232091 | 4.14E-05 |
| 5：79308315 | Intron:THBS4 | 38.82 | 1232091 | 3.15E-05 |
| 5：80263865 | Intron:RASGRF2 | 37.45 | 1232091 | 3.04E-05 |
| 5：87685500 | Intron:TMEM161B-AS | 107.04 | 1232091 | 8.69E-05 |
| 5：88873832 | Intergenic | 44.94 | 1232091 | 3.65E-05 |
| 5：91366274 | Intergenic | 38.92 | 1232091 | 3.16E-05 |
| 5：94198290 | Intron:MCTP1 | 36.43 | 1232091 | 2.96E-05 |
| 5：103929588 | Intergenic | 57.58 | 1232091 | 4.67E-05 |
| 5：106455988 | Intergenic | 49.54 | 1232091 | 4.02E-05 |
| 5：106825618 | Intron:EFNA5 | 47.87 | 1232091 | 3.89E-05 |
| 5：107365642 | Intron:FBXL17 | 37.4 | 1232091 | 3.04E-05 |
| 5：133865452 | Intron:JADE2 | 39.79 | 1232091 | 3.23E-05 |
| 5：154839646 | Intergenic | 38.85 | 1232091 | 3.15E-05 |
| 5：157707571 | Intergenic | 37.8 | 1232091 | 3.07E-05 |
| 5：161018271 | Intergenic | 32.4 | 1232091 | 2.63E-05 |
| 5：165096435 | Intergenic | 65.86 | 1232091 | 5.35E-05 |
| 5：165427280 | Intergenic | 33.73 | 1232091 | 2.74E-05 |
| 5：166063680 | Intergenic | 46.46 | 1232091 | 3.77E-05 |
| 5：166778503 | Intron:TENM2 | 55.1 | 1232091 | 4.47E-05 |
| 5：166989513 | Intron:TENM2 | 70.06 | 1232091 | 5.69E-05 |
| 5：167614971 | Intron:TENM2 | 73.02 | 1232091 | 5.93E-05 |
| 5：170299916 | Intron:RANBP17 | 38.26 | 1232091 | 3.10E-05 |
| 5：173288534 | Intergenic | 29.83 | 1232091 | 2.42E-05 |
| 6：26171250 | Utr3:HIST1H2BD | 51.18 | 1232091 | 4.15E-05 |
| 6：26571629 | Intron:LOC105374988 | 33.28 | 1232091 | 2.70E-05 |
| 6：28129789 | Intron:ZNF192P1 | 50.63 | 1232091 | 4.11E-05 |
| 6：29796685 | Intron:HLA-G | 36.93 | 1232091 | 3.00E-05 |
| 6：41901763 | Intergenic | 46.62 | 1232091 | 3.78E-05 |
| 6：52895230 | Intron:ICK | 35.59 | 1232091 | 2.89E-05 |
| 6：67521222 | Intergenic | 59.6 | 1232091 | 4.84E-05 |
| 6：67540984 | Intergenic | 48.04 | 1232091 | 3.90E-05 |

| 6：69470709 | Intron:ADGRB3 | 38.95 | 1232091 | 3.16E-05 |
| --- | --- | --- | --- | --- |
| 6：92226609 | Intergenic | 29.83 | 1232091 | 2.42E-05 |
| 6：94175279 | Intergenic | 30.25 | 1232091 | 2.46E-05 |
| 6：97702876 | Intron:MIR548H3|MM | 39.69 | 1232091 | 3.22E-05 |
| 6：98636905 | Intergenic | 79.08 | 1232091 | 6.42E-05 |
| 6：100347745 | Intergenic | 51.53 | 1232091 | 4.18E-05 |
| 6：101329173 | Utr5:ASCC3 | 54.17 | 1232091 | 4.40E-05 |
| 6：108994161 | Intron:FOXO3 | 35.18 | 1232091 | 2.86E-05 |
| 6：111658371 | Intron:REV3L | 123.68 | 1232091 | 1.00E-04 |
| 6：156431856 | Intergenic | 31.08 | 1232091 | 2.52E-05 |
| 6：157738258 | Intron:TMEM242 | 29.41 | 1232091 | 2.39E-05 |
| 6：158882320 | Intron:TULP4 | 35.79 | 1232091 | 2.90E-05 |
| 6：163807748 | Intergenic | 34.36 | 1232091 | 2.79E-05 |
| 7：1703675 | Intergenic | 59.92 | 1232091 | 4.86E-05 |
| 7：1870794 | Intron:MAD1L1 | 87.35 | 1232091 | 7.09E-05 |
| 7：3329967 | Intergenic | 34.15 | 1232091 | 2.77E-05 |
| 7：3503207 | Intron:SDK1 | 89.87 | 1232091 | 7.29E-05 |
| 7：69432311 | Intron:AUTS2 | 30.07 | 1232091 | 2.44E-05 |
| 7：69484366 | Intron:AUTS2 | 62.82 | 1232091 | 5.10E-05 |
| 7：77771983 | Intron:MAGI2 | 37.79 | 1232091 | 3.07E-05 |
| 7：78139581 | Intron:MAGI2 | 30.25 | 1232091 | 2.46E-05 |
| 7：88442568 | Intron:ZNF804B | 30.45 | 1232091 | 2.47E-05 |
| 7：91281409 | Intergenic | 30.25 | 1232091 | 2.46E-05 |
| 7：96629103 | Intron:DLX6-AS1 | 47.46 | 1232091 | 3.85E-05 |
| 7：97707069 | Intergenic | 40.76 | 1232091 | 3.31E-05 |
| 7：99071478 | Intron:ZNF789 | 59.95 | 1232091 | 4.87E-05 |
| 7：111100585 | Intron:IMMP2L | 45.3 | 1232091 | 3.68E-05 |
| 7：114940159 | Intergenic | 75.11 | 1232091 | 6.10E-05 |
| 7：115077394 | Intergenic | 42.36 | 1232091 | 3.44E-05 |
| 7：117523709 | Intergenic | 96.83 | 1232091 | 7.86E-05 |
| 7：121954709 | Intergenic | 30.67 | 1232091 | 2.49E-05 |
| 7：132593831 | Intron:CHCHD3 | 29.47 | 1232091 | 2.39E-05 |
| 7：133589846 | Intron:EXOC4 | 52.84 | 1232091 | 4.29E-05 |
| 7：133840652 | Intron:LRGUK | 34.18 | 1232091 | 2.77E-05 |
| 8：10143164 | Intron:MSRA | 29.88 | 1232091 | 2.43E-05 |
| 8：10763655 | Intron:XKR6 | 45.82 | 1232091 | 3.72E-05 |
| 8：27344719 | Intergenic | 55.39 | 1232091 | 4.50E-05 |
| 8：27425349 | Intergenic | 181.09 | 1232091 | 1.47E-04 |
| 8：27426077 | Intergenic | 36 | 1232091 | 2.92E-05 |
| 8：52569449 | Intron:PXDNL | 30.64 | 1232091 | 2.49E-05 |
| 8：59814666 | Intron:TOX | 64 | 1232091 | 5.19E-05 |
| 8：64912021 | Intron:LOC102724623 | 53.97 | 1232091 | 4.38E-05 |
| 8：91096366 | Intergenic | 55.97 | 1232091 | 4.54E-05 |
| 8：92733282 | Intergenic | 53.97 | 1232091 | 4.38E-05 |
| 8：93048104 | Intron:RUNX1T1 | 61.45 | 1232091 | 4.99E-05 |
| 8：93190014 | Intergenic | 62.17 | 1232091 | 5.05E-05 |
| 8：115374642 | Intergenic | 31.59 | 1232091 | 2.56E-05 |
| 9：3025368 | Intergenic | 51.73 | 1232091 | 4.20E-05 |
| 9：3398679 | Intron:RFX3 | 29.93 | 1232091 | 2.43E-05 |
| 9：8282399 | Intergenic | 30.9 | 1232091 | 2.51E-05 |
| 9：10981069 | Intergenic | 36 | 1232091 | 2.92E-05 |
| 9：11161799 | Intergenic | 49 | 1232091 | 3.98E-05 |
| 9：20676454 | Intron:FOCAD | 31.28 | 1232091 | 2.54E-05 |
| 9：23831658 | Intron:ELAVL2 | 49.97 | 1232091 | 4.06E-05 |
| 9：29747488 | Intergenic | 32.72 | 1232091 | 2.66E-05 |
| 9：81354129 | Intergenic | 44.94 | 1232091 | 3.65E-05 |
| 9：81444104 | Intergenic | 32.87 | 1232091 | 2.67E-05 |
| 9：86707289 | Intron:LOC101927575 | 34.18 | 1232091 | 2.77E-05 |

| 9：120519111 | Intergenic | 29.83 | 1232091 | 2.42E-05 |
| --- | --- | --- | --- | --- |
| 9：122061948 | Intron:BRINP1 | 35.08 | 1232091 | 2.85E-05 |
| 9：128073097 | Intron:GAPVD1 | 35.08 | 1232091 | 2.85E-05 |
| 9：134334588 | Nonsynonymous:PRRC | 36.29 | 1232091 | 2.95E-05 |
| 9：134907263 | Intron:MED27 | 35.15 | 1232091 | 2.85E-05 |
| 9：137989785 | Utr3:OLFM1 | 36.32 | 1232091 | 2.95E-05 |
| 10：8803551 | Intergenic | 31.98 | 1232091 | 2.60E-05 |
| 10：10043159 | Intergenic | 35.54 | 1232091 | 2.88E-05 |
| 10：11133823 | Intron:CELF2|CELF2- | 31.1 | 1232091 | 2.52E-05 |
| 10：13533053 | Intron:BEND7 | 31.1 | 1232091 | 2.52E-05 |
| 10：22032942 | Intergenic | 61.73 | 1232091 | 5.01E-05 |
| 10：36634124 | Intergenic | 31.1 | 1232091 | 2.52E-05 |
| 10：56698174 | Intron:PCDH15 | 35.05 | 1232091 | 2.84E-05 |
| 10：63674885 | Intron:ARID5B | 86.86 | 1232091 | 7.05E-05 |
| 10：103186838 | Intron:BTRC | 41.39 | 1232091 | 3.36E-05 |
| 10：103960351 | Intergenic | 83.42 | 1232091 | 6.77E-05 |
| 10：104120522 | Intron:GBF1 | 30.25 | 1232091 | 2.46E-05 |
| 10：104403310 | Intergenic | 41.26 | 1232091 | 3.35E-05 |
| 10：104640052 | Intron:AS3MT|BORCS | 91.31 | 1232091 | 7.41E-05 |
| 10：104852121 | Intron:NT5C2 | 49.44 | 1232091 | 4.01E-05 |
| 10：106078937 | Intron:ITPRIP | 31.53 | 1232091 | 2.56E-05 |
| 10：106460460 | Intron:SORCS3 | 36 | 1232091 | 2.92E-05 |
| 10：106929313 | Intron:SORCS3 | 37.98 | 1232091 | 3.08E-05 |
| 10：115378364 | Intron:NRAP | 44.6 | 1232091 | 3.62E-05 |
| 10：118678712 | Intron:SHTN1 | 39.69 | 1232091 | 3.22E-05 |
| 10：125680419 | Intergenic | 47.46 | 1232091 | 3.85E-05 |
| 11：27679916 | Nonsynonymous:BDN | 78.83 | 1232091 | 6.40E-05 |
| 11：28652996 | Intergenic | 67.75 | 1232091 | 5.50E-05 |
| 11：32956492 | Nonsynonymous:QSER | 31.4 | 1232091 | 2.55E-05 |
| 11：41436297 | Intron:LRRC4C | 36.46 | 1232091 | 2.96E-05 |
| 11：42442826 | Intergenic | 31.04 | 1232091 | 2.52E-05 |
| 11：46078656 | Intron:PHF21A | 37.3 | 1232091 | 3.03E-05 |
| 11：46393574 | Intron:DGKZ | 32.89 | 1232091 | 2.67E-05 |
| 11：64133552 | Intron:RPS6KA4 | 47.4 | 1232091 | 3.85E-05 |
| 11：64485193 | Intron:NRXN2 | 30.86 | 1232091 | 2.50E-05 |
| 11：65561468 | Intron:OVOL1 | 29.41 | 1232091 | 2.39E-05 |
| 11：73309393 | Intergenic | 38.88 | 1232091 | 3.16E-05 |
| 11：85980958 | Intron:EED | 40.96 | 1232091 | 3.32E-05 |
| 11：92289734 | Intron:FAT3 | 32.4 | 1232091 | 2.63E-05 |
| 11：111979741 | Intergenic | 43.68 | 1232091 | 3.55E-05 |
| 11：112912811 | Intron:NCAM1 | 211.37 | 1232091 | 1.72E-04 |
| 11：112984491 | Intron:NCAM1 | 46.95 | 1232091 | 3.81E-05 |
| 11：113660576 | Intergenic | 41.26 | 1232091 | 3.35E-05 |
| 11：113994505 | Intron:ZBTB16 | 45.7 | 1232091 | 3.71E-05 |
| 11：121530888 | Intergenic | 45.82 | 1232091 | 3.72E-05 |
| 11：121634334 | Intergenic | 32.6 | 1232091 | 2.65E-05 |
| 11：122023187 | Intron:MIR100HG | 31.1 | 1232091 | 2.52E-05 |
| 11：131966264 | Intron:NTM | 41.53 | 1232091 | 3.37E-05 |
| 11：132098205 | Intron:NTM | 38.62 | 1232091 | 3.13E-05 |
| 12：16748721 | Intron:LMO3 | 36.46 | 1232091 | 2.96E-05 |
| 12：49952394 | Utr3:MCRS1 | 35.71 | 1232091 | 2.90E-05 |
| 12：56474480 | Intron:ERBB3 | 33.38 | 1232091 | 2.71E-05 |
| 12：69655167 | Intron:CPSF6 | 36.86 | 1232091 | 2.99E-05 |
| 12：75263193 | Intergenic | 29.68 | 1232091 | 2.41E-05 |
| 12：112618346 | Intron:HECTD4 | 39.89 | 1232091 | 3.24E-05 |
| 12：121083279 | Intron:CABP1 | 41.26 | 1232091 | 3.35E-05 |
| 12：133380790 | Intron:GOLGA3 | 36.27 | 1232091 | 2.94E-05 |
| 13：38172867 | Utr5:POSTN | 30.67 | 1232091 | 2.49E-05 |

| 13：38359676 | Intron:TRPC4 | 32.4 | 1232091 | 2.63E-05 |
| --- | --- | --- | --- | --- |
| 13：55834929 | Intergenic | 42.88 | 1232091 | 3.48E-05 |
| 13：55931424 | Intergenic | 30.94 | 1232091 | 2.51E-05 |
| 13：59265043 | Intergenic | 44.79 | 1232091 | 3.63E-05 |
| 13：59339281 | Intergenic | 34.63 | 1232091 | 2.81E-05 |
| 13：59454139 | Intergenic | 38.17 | 1232091 | 3.10E-05 |
| 13：59871584 | Intergenic | 30.72 | 1232091 | 2.49E-05 |
| 13：60705792 | Intron:DIAPH3 | 38.82 | 1232091 | 3.15E-05 |
| 13：66949370 | Intron:PCDH9 | 50.13 | 1232091 | 4.07E-05 |
| 13：80191873 | Intergenic | 38.34 | 1232091 | 3.11E-05 |
| 13：81191176 | Intergenic | 39.18 | 1232091 | 3.18E-05 |
| 13：96823724 | Intron:HS6ST3 | 34.62 | 1232091 | 2.81E-05 |
| 13：100546450 | Intron:CLYBL|LOC10 | 56.01 | 1232091 | 4.55E-05 |
| 13：100648356 | Exon:LINC00554 | 59.44 | 1232091 | 4.82E-05 |
| 13：101252635 | Intergenic | 35.54 | 1232091 | 2.88E-05 |
| 14：28346502 | Intergenic | 44.79 | 1232091 | 3.63E-05 |
| 14：29316842 | Intergenic | 39.06 | 1232091 | 3.17E-05 |
| 14：58653514 | Intergenic | 29.83 | 1232091 | 2.42E-05 |
| 14：77529375 | Intron:LINC02288 | 37.21 | 1232091 | 3.02E-05 |
| 14：79563654 | Intron:NRXN3 | 38.26 | 1232091 | 3.10E-05 |
| 14：80102233 | Intron:NRXN3 | 34.68 | 1232091 | 2.81E-05 |
| 14：98362355 | Intergenic | 38.72 | 1232091 | 3.14E-05 |
| 14：98597552 | Intergenic | 47.93 | 1232091 | 3.89E-05 |
| 14：99693843 | Intron:BCL11B | 29.93 | 1232091 | 2.43E-05 |
| 15：36399479 | Intergenic | 29.41 | 1232091 | 2.39E-05 |
| 15：47685010 | Intron:SEMA6D | 83.69 | 1232091 | 6.79E-05 |
| 15：47935843 | Intron:SEMA6D | 49.54 | 1232091 | 4.02E-05 |
| 15：63898709 | Intergenic | 35.03 | 1232091 | 2.84E-05 |
| 15：67476952 | Intron:SMAD3 | 34.81 | 1232091 | 2.83E-05 |
| 15：74048768 | Intergenic | 30.25 | 1232091 | 2.46E-05 |
| 15：78025464 | Intron:LINGO1 | 37.4 | 1232091 | 3.04E-05 |
| 15：83893243 | Intergenic | 56.01 | 1232091 | 4.55E-05 |
| 15：96858409 | Intron:NR2F2-AS1 | 36.8 | 1232091 | 2.99E-05 |
| 15：99204101 | Intron:IGF1R | 37.45 | 1232091 | 3.04E-05 |
| 16：720986 | Nonsynonymous:RHO | 64.53 | 1232091 | 5.24E-05 |
| 16：5811367 | Intergenic | 42.72 | 1232091 | 3.47E-05 |
| 16：10173748 | Intron:GRIN2A | 45.7 | 1232091 | 3.71E-05 |
| 16：17570220 | Intergenic | 48.13 | 1232091 | 3.91E-05 |
| 16：18050926 | Intergenic | 47.46 | 1232091 | 3.85E-05 |
| 16：24727064 | Intron:TNRC6A | 60.96 | 1232091 | 4.95E-05 |
| 16：25351633 | Intergenic | 61.81 | 1232091 | 5.02E-05 |
| 16：49766772 | Intron:ZNF423 | 41.62 | 1232091 | 3.38E-05 |
| 16：49891964 | Intergenic | 41.58 | 1232091 | 3.37E-05 |
| 16：50945156 | Intergenic | 43.12 | 1232091 | 3.50E-05 |
| 16：69576894 | Intergenic | 51.73 | 1232091 | 4.20E-05 |
| 16：72230694 | Intergenic | 28.99 | 1232091 | 2.35E-05 |
| 16：72574550 | Intron:LINC01572 | 40.7 | 1232091 | 3.30E-05 |
| 16：72950468 | Intron:ZFHX3 | 34.81 | 1232091 | 2.83E-05 |
| 16：75620118 | Intergenic | 33.77 | 1232091 | 2.74E-05 |
| 16：75766873 | Intergenic | 34.88 | 1232091 | 2.83E-05 |
| 16：87443734 | Utr3:ZCCHC14 | 32.4 | 1232091 | 2.63E-05 |
| 16：89608702 | Intron:SPG7 | 36 | 1232091 | 2.92E-05 |
| 17：1995177 | Intron:SMG6 | 44.79 | 1232091 | 3.63E-05 |
| 17：7795972 | Intron:CHD3 | 31.53 | 1232091 | 2.56E-05 |
| 17：7803118 | Intron:CHD3 | 41.9 | 1232091 | 3.40E-05 |
| 17：16235462 | Intergenic | 31.36 | 1232091 | 2.55E-05 |
| 17：27323322 | Intron:SEZ6 | 52.35 | 1232091 | 4.25E-05 |
| 17：30685935 | Intron:ZNF207 | 33.73 | 1232091 | 2.74E-05 |

| 17：37824339 | Intron:PNMT | 29.64 | 1232091 | 2.41E-05 |
| --- | --- | --- | --- | --- |
| 17：44121579 | Intron:KANSL1 | 32.49 | 1232091 | 2.64E-05 |
| 17：44793283 | Intron:NSF | 52.7 | 1232091 | 4.28E-05 |
| 17：50193197 | Intron:CA10 | 52.44 | 1232091 | 4.26E-05 |
| 17：50243397 | Intergenic | 39.06 | 1232091 | 3.17E-05 |
| 17：77790135 | Intergenic | 34.57 | 1232091 | 2.81E-05 |
| 18：5872472 | Intergenic | 32.72 | 1232091 | 2.66E-05 |
| 18：25253297 | Intergenic | 38.03 | 1232091 | 3.09E-05 |
| 18：31696075 | Intron:NOL4 | 42.75 | 1232091 | 3.47E-05 |
| 18：36921851 | Intron:MIR924HG | 30.64 | 1232091 | 2.49E-05 |
| 18：39297254 | Intergenic | 45.79 | 1232091 | 3.72E-05 |
| 18：41314171 | Intergenic | 30.67 | 1232091 | 2.49E-05 |
| 18：42658643 | Intergenic | 48.6 | 1232091 | 3.94E-05 |
| 18：49874515 | Intron:DCC | 32.65 | 1232091 | 2.65E-05 |
| 18：49967811 | Intron:DCC | 60.96 | 1232091 | 4.95E-05 |
| 18：50863861 | Intron:DCC | 46.34 | 1232091 | 3.76E-05 |
| 18：53661743 | Intergenic | 46.24 | 1232091 | 3.75E-05 |
| 18：72535282 | Intron:ZNF407 | 34.77 | 1232091 | 2.82E-05 |
| 18：77574374 | Intergenic | 41.44 | 1232091 | 3.36E-05 |
| 19：4474725 | Intron:HDGFL2 | 34.19 | 1232091 | 2.78E-05 |
| 19：4965064 | Intergenic | 30.05 | 1232091 | 2.44E-05 |
| 19：18460956 | Intron:PGPEP1 | 42.47 | 1232091 | 3.45E-05 |
| 19：18566395 | Intron:ELL | 34.24 | 1232091 | 2.78E-05 |
| 19：51129745 | Intron:SYT3 | 35.3 | 1232091 | 2.86E-05 |
| 19：55879872 | Synonymous:IL11 | 29.83 | 1232091 | 2.42E-05 |
| 20：25195509 | Nonsynonymous:ENTP | 51.24 | 1232091 | 4.16E-05 |
| 20：29946968 | Intergenic | 45.56 | 1232091 | 3.70E-05 |
| 20：31175258 | Upstream:NOL4L-DT | 48.48 | 1232091 | 3.93E-05 |
| 20：42015801 | Intergenic | 30.25 | 1232091 | 2.46E-05 |
| 20：54462393 | Intergenic | 33.56 | 1232091 | 2.72E-05 |
| 20：61984317 | Intron:CHRNA4 | 36 | 1232091 | 2.92E-05 |
| 20：62406886 | Intron:ZBTB46 | 37.4 | 1232091 | 3.04E-05 |
| 21：40588819 | Intron:BRWD1 | 61.56 | 1232091 | 5.00E-05 |
| 22：38397797 | Intron:POLR2F | 32.53 | 1232091 | 2.64E-05 |
| 22：42698430 | Intergenic | 40.27 | 1232091 | 3.27E-05 |
| 22：46442288 | Intergenic | 35.54 | 1232091 | 2.88E-05 |
| 19:45411941 | APOE | 42.93 | 377234 | 1.14E-04 |
| 7:133447651 | EXOC4 | 38.32 | 377234 | 1.02E-04 |
| 6:32628084 | HLA-DQB1 | 31.64 | 377234 | 8.39E-05 |
| 7:50237784 | C7orf72/SPATA48 | 32.65 | 377234 | 8.66E-05 |
| 6:30327952 | RPP21 | 33.71 | 377234 | 8.94E-05 |
| 6:28002388 | ZNF165 | 30.44 | 377234 | 8.07E-05 |
| 3:85056521 | CADM2 | 37.05 | 377234 | 9.82E-05 |
| 9:37044388 | PAX5 | 44.44 | 377234 | 1.18E-04 |
| 7:128410012 | CALU | 29.34 | 377234 | 7.78E-05 |
| 3:85015779 | CADM2 | 60.42 | 261055 | 2.31E-04 |
| 6:108927842 | FOXO3 | 30.05 | 261055 | 1.15E-04 |
| 7:133506955 | EXOC4 | 35.28 | 261055 | 1.35E-04 |
| 10:126715436 | CTBP2 | 41.96 | 261055 | 1.61E-04 |
| 7:35020843 | DPY19L1 | 40.64 | 261055 | 1.56E-04 |
| 3:85400801 | CADM2 | 95.26 | 350492 | 2.72E-04 |
| 17:19799698 | AKAP10 | 43.27 | 350492 | 1.23E-04 |
| 6:26233387 | HIST1H1D | 30.75 | 350492 | 8.77E-05 |
| 5:60489247 | CTC-436P18.1 | 35.77 | 350492 | 1.02E-04 |
| 14:71826547 | SIPA1L1 | 37.85 | 350492 | 1.08E-04 |
| 19:19513570 | GATAD2A | 29.64 | 350492 | 8.46E-05 |
| 13:32191408 | RXFP2 | 124.28 | 757601 | 1.64E-04 |
| 14:50735947 | L2HGDH | 109.4 | 757601 | 1.44E-04 |

| 9:128498594 | PBX3 | 95.71 | 757601 | 1.26E-04 |
| --- | --- | --- | --- | --- |
| 19:5066330 | KDM4B | 91.66 | 757601 | 1.21E-04 |
| 18:777282 | YES1 | 90.05 | 757601 | 1.19E-04 |
| 11:46884713 | LRP4 | 89.24 | 757601 | 1.18E-04 |
| 5:1279790 | TERT | 88.94 | 757601 | 1.17E-04 |
| 14:103859962 | MARK3 | 88.55 | 757601 | 1.17E-04 |
| 4:138464842 | RP11-714L20.1 | 88.03 | 757601 | 1.16E-04 |
| 8:38130025 | WHSC1L1 | 87.05 | 757601 | 1.15E-04 |
| 17:18185510 | TOP3A | 85.28 | 757601 | 1.13E-04 |
| 13:22298923 | FGF9 | 84.38 | 757601 | 1.11E-04 |
| 4:18008232 | LCORL | 84.44 | 757601 | 1.11E-04 |
| 12:90567026 | ATP2B1* | 84.08 | 757601 | 1.11E-04 |
| 12:12888438 | APOLD1 | 82.55 | 757601 | 1.09E-04 |
| 1:43856410 | SZT2 | 82.08 | 757601 | 1.08E-04 |
| 7:130432469 | KLF14 | 79.9 | 757601 | 1.05E-04 |
| 8:10268736 | MSRA | 75.26 | 757601 | 9.93E-05 |
| 4:2694773 | FAM193A | 74.65 | 757601 | 9.85E-05 |
| 10:134459388 | INPP5A | 74.03 | 757601 | 9.77E-05 |
| 4:157678511 | PDGFC | 71.81 | 757601 | 9.48E-05 |
| 18:54578482 | WDR7 | 71.12 | 757601 | 9.39E-05 |
| 6:20686996 | CDKAL1 | 71.04 | 757601 | 9.38E-05 |
| 20:50108980 | NFATC2 | 70.55 | 757601 | 9.31E-05 |
| 15:86295286 | RP11-158M2.4 | 70.42 | 757601 | 9.29E-05 |
| 11:63744609 | AP000721.4 | 69.07 | 757601 | 9.12E-05 |
| 11:51539339 | TRIM48 | 68.81 | 757601 | 9.08E-05 |
| 5:96174186 | CTD-2260A17.2 | 68.58 | 757601 | 9.05E-05 |
| 18:48799991 | MEX3C | 67.91 | 757601 | 8.96E-05 |
| 12:58003922 | ARHGEF25 | 67.02 | 757601 | 8.85E-05 |
| 6:126228512 | NCOA7 | 65.77 | 757601 | 8.68E-05 |
| 5:68007803 | SLC30A5 | 65.16 | 757601 | 8.60E-05 |
| 3:30405936 | TGFBR2 | 64.73 | 757601 | 8.54E-05 |
| 13:73826901 | KLF5 | 64.75 | 757601 | 8.55E-05 |
| 15:85680532 | PDE8A | 64.1 | 757601 | 8.46E-05 |
| 12:102837863 | IGF1 | 62.87 | 757601 | 8.30E-05 |
| 12:79685226 | SYT1 | 62.69 | 757601 | 8.27E-05 |
| 1:249155909 | AL672294.1 | 62.38 | 757601 | 8.23E-05 |
| 17:56876627 | PPM1E | 61.42 | 757601 | 8.11E-05 |
| 18:60845884 | BCL2 | 61.44 | 757601 | 8.11E-05 |
| 2:43716933 | THADA | 61.24 | 757601 | 8.08E-05 |
| 1:180859368 | XPR1 | 61.21 | 757601 | 8.08E-05 |
| 1:184585182 | C1orf21 | 60.47 | 757601 | 7.98E-05 |
| 2:66782467 | MEIS1 | 60.29 | 757601 | 7.96E-05 |
| 5:140086677 | ZMAT2 | 60.28 | 757601 | 7.96E-05 |
| 15:100145224 | MEF2A | 59.03 | 757601 | 7.79E-05 |
| 9:9350706 | PTPRD | 57.56 | 757601 | 7.60E-05 |
| 6:1613686 | FOXC1 | 56.87 | 757601 | 7.51E-05 |
| 22:50727921 | PLXNB2 | 57.18 | 757601 | 7.55E-05 |
| 6:131311909 | EPB41L2 | 56.76 | 757601 | 7.49E-05 |
| 13:47189928 | LRCH1 | 56.17 | 757601 | 7.41E-05 |
| 10:133773019 | PPP2R2D | 56.28 | 757601 | 7.43E-05 |
| 12:105871914 | C12orf75 | 54.71 | 757601 | 7.22E-05 |
| 7:30933453 | AQP1 | 54.7 | 757601 | 7.22E-05 |
| 8:81393697 | Y_RNA | 54.65 | 757601 | 7.21E-05 |
| 1:27284913 | C1orf172 | 54.19 | 757601 | 7.15E-05 |
| 13:51489186 | RNASEH2B | 53.94 | 757601 | 7.12E-05 |
| 14:72462381 | RGS6 | 53.28 | 757601 | 7.03E-05 |
| 14:75074316 | LTBP2 | 53.12 | 757601 | 7.01E-05 |
| 3:41107173 | CTNNB1 | 52.51 | 757601 | 6.93E-05 |

| 5:55868097 | AC022431.2 | 51.85 | 757601 | 6.84E-05 |
| --- | --- | --- | --- | --- |
| 11:30355707 | ARL14EP | 51.74 | 757601 | 6.83E-05 |
| 7:77572461 | PHTF2 | 51.59 | 757601 | 6.81E-05 |
| 8:9730663 | TNKS | 51.33 | 757601 | 6.78E-05 |
| 9:95201540 | CENPP | 51.11 | 757601 | 6.75E-05 |
| 15:76276150 | NRG4 | 51.06 | 757601 | 6.74E-05 |
| 2:242344695 | FARP2 | 50.78 | 757601 | 6.70E-05 |
| 8:143312933 | TSNARE1 | 50.55 | 757601 | 6.67E-05 |
| 4:95938386 | BMPR1B | 50.17 | 757601 | 6.62E-05 |
| 8:129483956 | RP11-89M16.1 | 50.05 | 757601 | 6.61E-05 |
| 10:74751579 | PLA2G12B | 49.73 | 757601 | 6.56E-05 |
| 2:18975439 | NT5C1B | 49.1 | 757601 | 6.48E-05 |
| 9:130309028 | FAM129B | 48.62 | 757601 | 6.42E-05 |
| 2:112769721 | MERTK | 48.63 | 757601 | 6.42E-05 |
| 12:1059556 | RAD52 | 48.33 | 757601 | 6.38E-05 |
| 1:22577371 | WNT4 | 48.39 | 757601 | 6.39E-05 |
| 5:127352807 | CTC-228N24.3 | 47.57 | 757601 | 6.28E-05 |
| 10:48411796 | GDF2 | 47.48 | 757601 | 6.27E-05 |
| 2:60096560 | RP11-444A22.1 | 47.45 | 757601 | 6.26E-05 |
| 6:140383733 | CITED2 | 47.05 | 757601 | 6.21E-05 |
| 3:20073193 | KAT2B | 46.04 | 757601 | 6.08E-05 |
| 7:75097488 | POM121C | 45.76 | 757601 | 6.04E-05 |
| 2:187816321 | ZSWIM2 | 45.44 | 757601 | 6.00E-05 |
| 3:132780356 | TMEM108 | 45.41 | 757601 | 5.99E-05 |
| 1:94051350 | BCAR3 | 45.16 | 757601 | 5.96E-05 |
| 10:118523933 | HSPA12A | 45.16 | 757601 | 5.96E-05 |
| 17:58950791 | BCAS3 | 45.22 | 757601 | 5.97E-05 |
| 17:30032420 | RP11-805L22.1 | 45 | 757601 | 5.94E-05 |
| 11:68023742 | C11orf24 | 44.64 | 757601 | 5.89E-05 |
| 2:122000745 | TFCP2L1 | 44.75 | 757601 | 5.91E-05 |
| 4:46595623 | GABRA2 | 44.43 | 757601 | 5.86E-05 |
| 3:135949737 | PCCB | 44.21 | 757601 | 5.84E-05 |
| 2:161368213 | RBMS1 | 43.63 | 757601 | 5.76E-05 |
| 1:94730954 | ARHGAP29 | 42.79 | 757601 | 5.65E-05 |
| 2:230629138 | TRIP12 | 42.74 | 757601 | 5.64E-05 |
| 1:197297417 | CRB1 | 42.75 | 757601 | 5.64E-05 |
| 2:213188795 | ERBB4 | 42.42 | 757601 | 5.60E-05 |
| 7:28142088 | JAZF1 | 42.22 | 757601 | 5.57E-05 |
| 16:85318302 | LINC00311 | 41.54 | 757601 | 5.48E-05 |
| 1:209970355 | IRF6 | 41.61 | 757601 | 5.49E-05 |
| 17:79367409 | RP11-1055B8.6 | 41.76 | 757601 | 5.51E-05 |
| 9:4334791 | GLIS3 | 41.35 | 757601 | 5.46E-05 |
| 13:42738672 | DGKH | 40.9 | 757601 | 5.40E-05 |
| 3:158316726 | MLF1 | 40.8 | 757601 | 5.39E-05 |
| 1:203109801 | ADORA1 | 40.78 | 757601 | 5.38E-05 |
| 1:156129796 | SEMA4A | 40.54 | 757601 | 5.35E-05 |
| 19:50935809 | SPIB | 40.2 | 757601 | 5.31E-05 |
| 7:150050111 | RARRES2 | 39.86 | 757601 | 5.26E-05 |
| 3:49381898 | ARIH2 | 39.69 | 757601 | 5.24E-05 |
| 14:30122409 | PRKD1 | 39.39 | 757601 | 5.20E-05 |
| 2:185033065 | ZNF804A | 38.2 | 757601 | 5.04E-05 |
| 12:24210599 | SOX5 | 37.92 | 757601 | 5.00E-05 |
| 3:74710462 | CNTN3 | 37.37 | 757601 | 4.93E-05 |
| 9:131940019 | IER5L | 37.2 | 757601 | 4.91E-05 |
| 2:55279681 | RTN4 | 37.07 | 757601 | 4.89E-05 |
| 7:140238048 | DENND2A | 37.02 | 757601 | 4.89E-05 |
| 11:8774923 | ST5 | 36.56 | 757601 | 4.83E-05 |
| 5:65662133 | SREK1 | 35.95 | 757601 | 4.75E-05 |

| 5:33411769 | TARS | 35.79 | 757601 | 4.72E-05 |
| --- | --- | --- | --- | --- |
| 16:6889675 | RBFOX1 | 35.78 | 757601 | 4.72E-05 |
| 13:79808655 | RBM26 | 35.33 | 757601 | 4.66E-05 |
| 11:34068037 | CAPRIN1 | 34.43 | 757601 | 4.54E-05 |
| 2:138421227 | THSD7B | 34.24 | 757601 | 4.52E-05 |
| 1:2187085 | SKI | 34.18 | 757601 | 4.51E-05 |
| 7:56122058 | CCT6A | 33.29 | 757601 | 4.39E-05 |
| 6:119113317 | MCM9 | 32.99 | 757601 | 4.35E-05 |
| 2:50429861 | NRXN1 | 32.86 | 757601 | 4.34E-05 |
| 3:114461208 | ZBTB20 | 32.45 | 757601 | 4.28E-05 |
| 10:5804865 | FAM208B | 30.96 | 757601 | 4.09E-05 |
| 8:60535824 | TOX | 30.46 | 757601 | 4.02E-05 |
| 11:9762274 | SWAP70 | 159.06 | 757601 | 2.10E-04 |
| 4:102435265 | BANK1 | 111.77 | 757601 | 1.48E-04 |
| 7:1195692 | ZFAND2A | 102.43 | 757601 | 1.35E-04 |
| 5:61553881 | KIF2A | 91.41 | 757601 | 1.21E-04 |
| 18:51851616 | STARD6 | 89.83 | 757601 | 1.19E-04 |
| 7:96461649 | SHFM1 | 89.94 | 757601 | 1.19E-04 |
| 16:66914492 | PDP2 | 89.94 | 757601 | 1.19E-04 |
| 4:103769304 | UBE2D3 | 89.37 | 757601 | 1.18E-04 |
| 1:78450517 | DNAJB4 | 88.27 | 757601 | 1.16E-04 |
| 1:218549354 | TGFB2 | 86.29 | 757601 | 1.14E-04 |
| 4:26785356 | STIM2 | 85.18 | 757601 | 1.12E-04 |
| 12:8832203 | RP11-20D14.4 | 84.5 | 757601 | 1.12E-04 |
| 6:43809802 | VEGFA | 76.59 | 757601 | 1.01E-04 |
| 11:22515533 | RP11-34N19.1 | 75.96 | 757601 | 1.00E-04 |
| 2:219651349 | CYP27A1 | 74.24 | 757601 | 9.80E-05 |
| 12:124820705 | NCOR2 | 73.02 | 757601 | 9.64E-05 |
| 10:102075479 | PKD2L1 | 72.25 | 757601 | 9.54E-05 |
| 3:194299967 | TMEM44 | 71.33 | 757601 | 9.41E-05 |
| 12:57098040 | NACA | 70.69 | 757601 | 9.33E-05 |
| 18:10879503 | PIEZO2 | 70.75 | 757601 | 9.34E-05 |
| 19:32590773 | AC011518.1 | 66.64 | 757601 | 8.80E-05 |
| 6:34244132 | NUDT3 | 66.86 | 757601 | 8.82E-05 |
| 5:3706050 | IRX1 | 65.98 | 757601 | 8.71E-05 |
| 2:158499902 | ACVR1C | 65.58 | 757601 | 8.66E-05 |
| 11:70005641 | ANO1 | 64.96 | 757601 | 8.57E-05 |
| 6:139835689 | CITED2 | 64.32 | 757601 | 8.49E-05 |
| 8:141858620 | PTK2 | 64.32 | 757601 | 8.49E-05 |
| 3:101268080 | TRMT10C | 63.79 | 757601 | 8.42E-05 |
| 1:46541679 | PIK3R3 | 59.75 | 757601 | 7.89E-05 |
| 7:134215259 | AKR1B10 | 58.08 | 757601 | 7.67E-05 |
| 12:120813921 | RPS27P25 | 56.79 | 757601 | 7.50E-05 |
| 20:11886643 | BTBD3 | 56.99 | 757601 | 7.52E-05 |
| 17:19204432 | EPN2 | 56.59 | 757601 | 7.47E-05 |
| 5:132397351 | HSPA4 | 56.2 | 757601 | 7.42E-05 |
| 13:50564490 | TRIM13 | 56.15 | 757601 | 7.41E-05 |
| 16:30111904 | RP11-455F5.3 | 56.16 | 757601 | 7.41E-05 |
| 14:60700903 | PPM1A | 55.87 | 757601 | 7.37E-05 |
| 5:66291370 | MAST4 | 55.81 | 757601 | 7.37E-05 |
| 10:13523937 | BEND7 | 55.11 | 757601 | 7.27E-05 |
| 2:127183454 | GYPC | 53.42 | 757601 | 7.05E-05 |
| 2:144146311 | ARHGAP15 | 52.87 | 757601 | 6.98E-05 |
| 13:97988689 | MBNL2 | 52.92 | 757601 | 6.98E-05 |
| 1:119541452 | RP4-712E4.1 | 52.23 | 757601 | 6.89E-05 |
| 2:146272860 | ZEB2 | 51.75 | 757601 | 6.83E-05 |
| 14:23313633 | MMP14 | 51.98 | 757601 | 6.86E-05 |
| 4:2165493 | POLN | 51.87 | 757601 | 6.85E-05 |

| 2:98357163 | ZAP70 | 51.88 | 757601 | 6.85E-05 |
| --- | --- | --- | --- | --- |
| 16:69131281 | HAS3 | 51.11 | 757601 | 6.75E-05 |
| 5:57095011 | ACTBL2 | 51.39 | 757601 | 6.78E-05 |
| 7:156311745 | LINC01006 | 51.01 | 757601 | 6.73E-05 |
| 1:51527684 | MIR4421 | 50.2 | 757601 | 6.63E-05 |
| 16:706067 | WDR90 | 48.29 | 757601 | 6.37E-05 |
| 8:144981488 | PLEC | 47.56 | 757601 | 6.28E-05 |
| 3:197070959 | DLG1 | 46.61 | 757601 | 6.15E-05 |
| 3:23163749 | UBE2E2 | 46.26 | 757601 | 6.11E-05 |
| 1:29549216 | MECR | 45.59 | 757601 | 6.02E-05 |
| 15:92702020 | SLCO3A1 | 45.52 | 757601 | 6.01E-05 |
| 18:20158965 | CTAGE1 | 45.04 | 757601 | 5.94E-05 |
| 17:8078765 | TMEM107 | 45.17 | 757601 | 5.96E-05 |
| 6:154418759 | OPRM1 | 44.42 | 757601 | 5.86E-05 |
| 2:34679626 | AC073218.1 | 44.59 | 757601 | 5.89E-05 |
| 12:96717095 | CDK17 | 43.77 | 757601 | 5.78E-05 |
| 9:109670016 | ZNF462 | 42.75 | 757601 | 5.64E-05 |
| 15:85162551 | ZSCAN2 | 41.36 | 757601 | 5.46E-05 |
| 11:4673788 | OR51E1 | 41.2 | 757601 | 5.44E-05 |
| 2:166250129 | SCN2A | 40.01 | 757601 | 5.28E-05 |
| 8:22428708 | SORBS3 | 39.66 | 757601 | 5.23E-05 |
| 3:46861939 | PRSS50 | 39.39 | 757601 | 5.20E-05 |
| 15:68454523 | PIAS1 | 39.42 | 757601 | 5.20E-05 |
| 4:95324968 | PDLIM5 | 38.88 | 757601 | 5.13E-05 |
| 14:35110857 | SNX6 | 38.9 | 757601 | 5.13E-05 |
| 1:145713305 | CD160 | 38.93 | 757601 | 5.14E-05 |
| 13:58316637 | PCDH17 | 38.93 | 757601 | 5.14E-05 |
| 11:6289118 | CCKBR | 38.75 | 757601 | 5.11E-05 |
| 10:126712781 | CTBP2 | 38.44 | 757601 | 5.07E-05 |
| 11:74374950 | POLD3 | 38.51 | 757601 | 5.08E-05 |
| 12:5417856 | RP11-1038A11.3 | 37.82 | 757601 | 4.99E-05 |
| 17:29161503 | ATAD5 | 37.53 | 757601 | 4.95E-05 |
| 12:27321112 | STK38L | 37.52 | 757601 | 4.95E-05 |
| 2:38094149 | LINC00211 | 36.79 | 757601 | 4.86E-05 |
| 21:37692507 | MORC3 | 36.3 | 757601 | 4.79E-05 |
| 7:74107374 | GTF2I | 36.3 | 757601 | 4.79E-05 |
| 7:4669949 | FOXK1 | 35.82 | 757601 | 4.73E-05 |
| 16:19149996 | CTD-2349B8.1 | 35.67 | 757601 | 4.71E-05 |
| 3:7489993 | GRM7 | 35.06 | 757601 | 4.63E-05 |
| 2:3636478 | COLEC11 | 34.43 | 757601 | 4.54E-05 |
| 4:57943153 | IGFBP7 | 34.06 | 757601 | 4.50E-05 |
| 1:117015118 | RP4-655J12.4 | 33.9 | 757601 | 4.47E-05 |
| 11:101100768 | PGR | 34.1 | 757601 | 4.50E-05 |
| 7:69769369 | AUTS2 | 33.32 | 757601 | 4.40E-05 |
| 12:15297359 | RERG | 32.45 | 757601 | 4.28E-05 |
| 20:10458688 | SLX4IP | 183.3 | 757601 | 2.42E-04 |
| 5:158367249 | EBF1* | 149.58 | 757601 | 1.97E-04 |
| 16:71654365 | RP11-432I5.2 | 147.95 | 757601 | 1.95E-04 |
| 17:45888374 | OSBPL7 | 134.72 | 757601 | 1.78E-04 |
| 19:45412079 | APOE | 134.17 | 757601 | 1.77E-04 |
| 7:22766645 | IL6 | 109.17 | 757601 | 1.44E-04 |
| 9:101739709 | COL15A1 | 100.34 | 757601 | 1.32E-04 |
| 1:219753509 | SLC30A10 | 97.89 | 757601 | 1.29E-04 |
| 8:92149429 | LRRC69 | 92.61 | 757601 | 1.22E-04 |
| 6:73657714 | KCNQ5 | 90.2 | 757601 | 1.19E-04 |
| 12:48721634 | H1FNT | 89.46 | 757601 | 1.18E-04 |
| 7:89805241 | STEAP2 | 86.43 | 757601 | 1.14E-04 |
| 12:22015022 | ABCC9 | 82.86 | 757601 | 1.09E-04 |

| 20:6657554 | BMP2 | 81.31 | 757601 | 1.07E-04 |
| --- | --- | --- | --- | --- |
| 2:169763148 | G6PC2 | 79.48 | 757601 | 1.05E-04 |
| 12:27962103 | RN7SKP15 | 78.95 | 757601 | 1.04E-04 |
| 6:36648816 | CDKN1A | 76.12 | 757601 | 1.00E-04 |
| 17:42680402 | FZD2 | 75.25 | 757601 | 9.93E-05 |
| 1:28734372 | PHACTR4 | 74.26 | 757601 | 9.80E-05 |
| 14:54107791 | AL163953.3 | 72.73 | 757601 | 9.60E-05 |
| 11:102077200 | YAP1 | 71.32 | 757601 | 9.41E-05 |
| 9:116696625 | ZNF618 | 69.57 | 757601 | 9.18E-05 |
| 17:40919596 | RAMP2 | 67.35 | 757601 | 8.89E-05 |
| 10:19934813 | MALRD1 | 66.32 | 757601 | 8.75E-05 |
| 1:43037556 | CCDC30 | 64.87 | 757601 | 8.56E-05 |
| 19:46180414 | GIPR | 63.26 | 757601 | 8.35E-05 |
| 16:1344291 | UBE2I | 61.16 | 757601 | 8.07E-05 |
| 3:36964583 | TRANK1 | 61.32 | 757601 | 8.09E-05 |
| 17:27195674 | ERAL1 | 60.42 | 757601 | 7.97E-05 |
| 3:37539090 | ITGA9 | 60.44 | 757601 | 7.98E-05 |
| 10:82215288 | TSPAN14 | 59.52 | 757601 | 7.86E-05 |
| 19:670234 | AC004156.3 | 58.9 | 757601 | 7.77E-05 |
| 17:55446364 | MSI2 | 58.92 | 757601 | 7.78E-05 |
| 2:68503044 | AC017083.3 | 58.18 | 757601 | 7.68E-05 |
| 21:35596842 | AP000320.7,AP00031 | 58.13 | 757601 | 7.67E-05 |
| 4:83925895 | LIN54 | 57.64 | 757601 | 7.61E-05 |
| 1:67071356 | SGIP1 | 56.81 | 757601 | 7.50E-05 |
| 3:38767315 | SCN10A | 56.46 | 757601 | 7.45E-05 |
| 6:12295987 | EDN1 | 56.16 | 757601 | 7.41E-05 |
| 1:46027355 | AKR1A1 | 55.86 | 757601 | 7.37E-05 |
| 12:95487226 | FGD6 | 55.48 | 757601 | 7.32E-05 |
| 14:100742658 | YY1 | 55.24 | 757601 | 7.29E-05 |
| 6:135119089 | ALDH8A1 | 55.28 | 757601 | 7.30E-05 |
| 3:99839106 | CMSS1 | 55.2 | 757601 | 7.29E-05 |
| 16:58566304 | CNOT1 | 55.19 | 757601 | 7.28E-05 |
| 2:180739450 | ZNF385B | 55.03 | 757601 | 7.26E-05 |
| 2:152978341 | STAM2 | 54.28 | 757601 | 7.16E-05 |
| 1:180131640 | QSOX1 | 53.5 | 757601 | 7.06E-05 |
| 8:110107161 | TRHR | 53.23 | 757601 | 7.03E-05 |
| 2:172381487 | CYBRD1 | 52.63 | 757601 | 6.95E-05 |
| 14:94465789 | LINC00521 | 52.47 | 757601 | 6.92E-05 |
| 11:92708710 | MTNR1B | 51.83 | 757601 | 6.84E-05 |
| 11:130779068 | SNX19 | 51.55 | 757601 | 6.80E-05 |
| 15:65166309 | AC069368.3 | 51.37 | 757601 | 6.78E-05 |
| 10:10876943 | CELF2 | 50.55 | 757601 | 6.67E-05 |
| 19:4085896 | MAP2K2 | 50.38 | 757601 | 6.65E-05 |
| 8:116959837 | LINC00536 | 49.18 | 757601 | 6.49E-05 |
| 9:118534500 | PAPPA | 48.62 | 757601 | 6.42E-05 |
| 7:46554358 | AC011294.3 | 48.06 | 757601 | 6.34E-05 |
| 4:10093651 | WDR1 | 47.3 | 757601 | 6.24E-05 |
| 9:127044135 | NEK6 | 46.88 | 757601 | 6.19E-05 |
| 17:74686809 | MXRA7 | 46.91 | 757601 | 6.19E-05 |
| 3:44858131 | KIF15 | 45.86 | 757601 | 6.05E-05 |
| 20:33121942 | DYNLRB1 | 45.47 | 757601 | 6.00E-05 |
| 22:30172254 | UQCR10 | 45.11 | 757601 | 5.95E-05 |
| 21:47962811 | DIP2A | 45.04 | 757601 | 5.94E-05 |
| 12:20754154 | PDE3A* | 44.79 | 757601 | 5.91E-05 |
| 5:52135543 | ITGA1 | 44.81 | 757601 | 5.91E-05 |
| 13:79238925 | RNF219 | 44.44 | 757601 | 5.87E-05 |
| 20:36849007 | KIAA1755 | 43.74 | 757601 | 5.77E-05 |
| 9:19057551 | HAUS6 | 43.65 | 757601 | 5.76E-05 |

| 18:12711052 | PSMG2 | 43.99 | 757601 | 5.81E-05 |
| --- | --- | --- | --- | --- |
| 5:43824677 | NNT | 43.44 | 757601 | 5.73E-05 |
| 6:7211818 | RREB1 | 43.61 | 757601 | 5.76E-05 |
| 15:59429160 | MYO1E | 43.3 | 757601 | 5.71E-05 |
| 7:107019947 | COG5 | 43 | 757601 | 5.68E-05 |
| 22:38117943 | TRIOBP,NOL12 | 42.91 | 757601 | 5.66E-05 |
| 4:15356795 | C1QTNF7 | 42.42 | 757601 | 5.60E-05 |
| 17:33313729 | LIG3 | 42.51 | 757601 | 5.61E-05 |
| 3:149524692 | ANKUB1 | 41.88 | 757601 | 5.53E-05 |
| 12:116699675 | MED13L | 41.91 | 757601 | 5.53E-05 |
| 2:189643316 | DIRC1 | 41.86 | 757601 | 5.53E-05 |
| 9:16872323 | BNC2 | 41.71 | 757601 | 5.51E-05 |
| 9:131210410 | RP11-339B21.8 | 41.3 | 757601 | 5.45E-05 |
| 18:60223017 | ZCCHC2 | 40.96 | 757601 | 5.41E-05 |
| 22:28056338 | MN1 | 40.64 | 757601 | 5.36E-05 |
| 5:72654304 | FOXD1 | 39.91 | 757601 | 5.27E-05 |
| 1:86822231 | ODF2L | 39.48 | 757601 | 5.21E-05 |
| 5:119781569 | PRR16 | 39.41 | 757601 | 5.20E-05 |
| 22:50219952 | BRD1 | 39.36 | 757601 | 5.20E-05 |
| 1:33868469 | PHC2 | 38.48 | 757601 | 5.08E-05 |
| 6:122287990 | HSF2 | 37.94 | 757601 | 5.01E-05 |
| 21:40817702 | LCA5L | 37.24 | 757601 | 4.92E-05 |
| 1:86040107 | DDAH1 | 37.04 | 757601 | 4.89E-05 |
| 5:107458637 | FBXL17 | 36.58 | 757601 | 4.83E-05 |
| 6:169016615 | SMOC2* | 35.79 | 757601 | 4.72E-05 |
| 14:55285588 | SAMD4A | 35.35 | 757601 | 4.67E-05 |
| 16:11198835 | CLEC16A | 34.36 | 757601 | 4.54E-05 |
| 15:84572456 | ADAMTSL3 | 33.77 | 757601 | 4.46E-05 |
| 7:41720763 | AC005027.3 | 32.83 | 757601 | 4.33E-05 |
| 2:66104881 | AC074391.1 | 31.7 | 757601 | 4.18E-05 |
| 3:157576791 | SHOX2 | 31.69 | 757601 | 4.18E-05 |
| 8:95969257 | NDUFAF6 | 31.18 | 757601 | 4.12E-05 |
| 11:116097136 | BUD13 | 29.81 | 757601 | 3.93E-05 |
| 2:27596107 | GCKR | 36 | 51450 | 6.99E-04 |
| 2:165252696 | COBLL1 | 25 | 51450 | 4.86E-04 |
| 3:158281469 | CCNL1 | 27.56 | 51450 | 5.35E-04 |
| 7:127647399 | LEP | 52.56 | 51450 | 1.02E-03 |
| 20:36766426 | SLC32A1 | 36 | 51450 | 6.99E-04 |
| 1:182549729 | RGS16 (RNASEL) | 76.17 | 89283 | 8.52E-04 |
| 6:153135339 | VIP | 45.03 | 89283 | 5.04E-04 |
| 2:239317692 | PER2 | 31.43 | 89283 | 3.52E-04 |
| 6:55021561 | HCRTR2 (aka OX2R) | 36.52 | 89283 | 4.09E-04 |
| 17:17398278 | RASD1 | 29.4 | 89283 | 3.29E-04 |
| 1:7836659 | PER3 (VAMP3) | 31.83 | 89283 | 3.56E-04 |
| 13:77577027 | FBXL3 (CLN5) | 33.64 | 89283 | 3.77E-04 |
| 2:198874006 | PLCL1 | 41.87 | 89283 | 4.69E-04 |
| 1:150234657 | APH1A (CA14) | 38.75 | 89283 | 4.34E-04 |
| 7:102436907 | FBXL13 (FAM185A) | 36 | 89283 | 4.03E-04 |
| 18:31675680 | NOL4 | 32.25 | 89283 | 3.61E-04 |
| 16:52684916 | TOX3 | 46.02 | 89283 | 5.15E-04 |
| 1:77726241 | AK5 | 54.28 | 89283 | 6.08E-04 |
| 7:96457119 | DLX5 (SHFM1) | 25.24 | 89283 | 2.83E-04 |
| 12:38726137 | ALG10B | 31.83 | 89283 | 3.56E-04 |
| 1:11778589 | MTHFR | 84.31 | 44147 | 1.91E-03 |
| 10:17181245 | CUBN | 34.55 | 44147 | 7.82E-04 |
| 16:89793345 | DPEP1/FANCA | 41.33 | 44147 | 9.35E-04 |
| 16:89641688 | DPEP1 | 189.26 | 44147 | 4.27E-03 |
| 1:11796321 | MTHFR | 511.41 | 44147 | 1.15E-02 |

| 10:17114152 | CUBN | 41.88 | 44147 | 9.48E-04 |
| --- | --- | --- | --- | --- |
| 12:120967323 | HNF1A | 53.5 | 44147 | 1.21E-03 |
| 1:236885376 | MTR | 36.27 | 44147 | 8.21E-04 |
| 21:43066854 | CBS | 105.21 | 44147 | 2.38E-03 |
| 21:43067294 | CBS | 49 | 44147 | 1.11E-03 |
| 7:90348446 | GTPB10 | 31.84 | 44147 | 7.21E-04 |
| 1:45513003 | MMACHC | 38.62 | 44147 | 8.74E-04 |
| 6:25869143 | SLC17A3 | 35.64 | 44147 | 8.07E-04 |
| 11:89415204 | NOX4 | 91.28 | 44147 | 2.06E-03 |
| 2:210675783 | CPS1 | 116.64 | 44147 | 2.64E-03 |
| 19:48756272 | FUT2 | 36.34 | 44147 | 8.23E-04 |
| 6:49414480 | MUT | 41.14 | 44147 | 9.31E-04 |
| 11:89468459 | NOX4 | 31.64 | 44147 | 7.16E-04 |
| 5:32850785 | NPR3-C5orf23 | 39.22 | 203056 | 1.93E-04 |
| 6:26199158 | HFE | 40.94 | 203056 | 2.02E-04 |
| 6:31724345 | BAT2-BAT5 | 41.86 | 203056 | 2.06E-04 |
| 10:95885930 | PLCE1 | 32.94 | 203056 | 1.62E-04 |
| 11:100098748 | FLJ32810-TMEM133 | 43.04 | 203056 | 2.12E-04 |
| 20:57184512 | GNAS-EDN3 | 57.18 | 203056 | 2.82E-04 |
| 1:11785365 | MTHFR-NPPB | 40.01 | 203056 | 1.97E-04 |
| 10:18747454 | CACNB2(3′) | 38.13 | 203056 | 1.88E-04 |
| 10:63137559 | C10orf107 | 32.72 | 203056 | 1.61E-04 |
| 12:88584717 | ATP2B1 | 59.85 | 203056 | 2.95E-04 |
| 15:72864420 | CYP1A1-ULK3 | 32.68 | 203056 | 1.61E-04 |
| 2:113548804 | IL1F10 | 169 | 5888 | 2.79E-02 |
| 2:113548783 | IL1F10 | 144 | 5888 | 2.39E-02 |
| 2:113551291 | NA | 169 | 5888 | 2.79E-02 |
| 12:123338164 | SBNO1 | 32.4 | 29857 | 1.08E-03 |
| 12:65964567 | HMGA2 | 42.25 | 29857 | 1.41E-03 |
| 10:17041344 | RSU1 | 20.87 | 2076 | 9.96E-03 |
| 7:156563826 | MNX1 | 22.37 | 2076 | 1.07E-02 |
| 8:53128610 | PCMTD1 | 19.67 | 2076 | 9.40E-03 |
| 2:55064109 | FLJ31438 | 19.7 | 2076 | 9.41E-03 |
| 8:5863417 | ANGPT2 | 27.88 | 2076 | 1.33E-02 |
| 1:19039958 | ALDH4A | 27.51 | 2076 | 1.31E-02 |
| 1:229153698 | ARV1 | 20.46 | 2076 | 9.77E-03 |
| 15:26804716 | GOLGA8 | 23.47 | 2076 | 1.12E-02 |
| 12:26899160 | C12orf11 | 20.4 | 2076 | 9.74E-03 |
| 2:226887874 | RS1 | 25 | 37638 | 6.64E-04 |
| 3:52597126 | GNL3 | 56.25 | 37638 | 1.49E-03 |
| 3:188031259 | ADIPOQ | 225 | 37638 | 5.94E-03 |
| 8:126550657 | TRIB1 | 56.25 | 37638 | 1.49E-03 |
| 16:80092291 | CMIP | 100 | 37638 | 2.65E-03 |
| 16:81220789 | CDH13 | 66.02 | 37638 | 1.75E-03 |
| 19:38597963 | PEPD | 36 | 37638 | 9.56E-04 |
| 1:33920586 | PHC2-ZSCAN20 | 40.29 | 382500 | 1.05E-04 |
| 1:47961691 | FOXD2-TRABD2B | 81.86 | 382500 | 2.14E-04 |
| 1:155095107 | EFNA3-EFNA1 | 33.91 | 382500 | 8.87E-05 |
| 2:27598097 | SNX17 | 48.18 | 382500 | 1.26E-04 |
| 2:85754342 | SH2D6-MAT2A/ PAR | 38.15 | 382500 | 9.97E-05 |
| 2:203714973 | ICA1L | 48.34 | 382500 | 1.26E-04 |
| 2:211540507 | CPS1 | 45.19 | 382500 | 1.18E-04 |
| 2:226684886 | MIR548AR- LOC6467 | 94.57 | 382500 | 2.47E-04 |
| 2:227876687 | COL4A4 | 32.84 | 382500 | 8.59E-05 |
| 2:229131286 | SPHKAP-PID1 | 96.38 | 382500 | 2.52E-04 |
| 3:170027407 | PRKCI | 47.74 | 382500 | 1.25E-04 |
| 4:77409795 | SHROOM3 | 36 | 382500 | 9.41E-05 |
| 4:149132756 | NR3C2 | 45.76 | 382500 | 1.20E-04 |

| 4:190729009 | LINC01262- FRG1 | 145.66 | 382500 | 3.81E-04 |
| --- | --- | --- | --- | --- |
| 5:53271420 | ARL15 | 33.38 | 382500 | 8.73E-05 |
| 5:64296471 | CWC27 | 39.06 | 382500 | 1.02E-04 |
| 7:17284577 | AGR3-AHR | 112.11 | 382500 | 2.93E-04 |
| 7:27243238 | HOTTIP | 36.38 | 382500 | 9.51E-05 |
| 7:29805361 | WIPF3-DPY19L2P3 | 49 | 382500 | 1.28E-04 |
| 8:126500031 | TRIB1- LINC00861 | 44.97 | 382500 | 1.18E-04 |
| 10:16932384 | CUBN | 413.83 | 382500 | 1.08E-03 |
| 10:16967417 | CUBN | 44.34 | 382500 | 1.16E-04 |
| 10:16971426 | CUBN | 34.13 | 382500 | 8.92E-05 |
| 10:16992011 | CUBN | 484.54 | 382500 | 1.27E-03 |
| 10:77893686 | LRMDA | 52.39 | 382500 | 1.37E-04 |
| 11:10296836 | SBF2 | 34.98 | 382500 | 9.14E-05 |
| 12:69979517 | CCT2 | 42.98 | 382500 | 1.12E-04 |
| 14:69302399 | ZFP36L1- ACTN1 | 33.23 | 382500 | 8.69E-05 |
| 15:42077961 | MAPKBP1 | 52.35 | 382500 | 1.37E-04 |
| 15:45703824 | SPATA5L1 | 36 | 382500 | 9.41E-05 |
| 15:75027880 | CYP1A2- CYP1A1 | 90.75 | 382500 | 2.37E-04 |
| 17:79419025 | BAHCC1 | 38.15 | 382500 | 9.97E-05 |
| 19:49252151 | FUT1 | 37.92 | 382500 | 9.91E-05 |
| 1:4548453 | NA | 36.67 | 939356 | 3.90E-05 |
| 1:33837334 | ZNF362,A3GALT2,PH | 36.75 | 939356 | 3.91E-05 |
| 1:66407700 | PDE4B | 33.79 | 939356 | 3.60E-05 |
| 1:71491890 | PTGER3,ZRANB2|ZR | 36.75 | 939356 | 3.91E-05 |
| 1:165119792 | NA | 28.89 | 939356 | 3.08E-05 |
| 1:173848808 | KLHL20,CENPL,DAR | 37.35 | 939356 | 3.98E-05 |
| 1:205719532 | SLC45A3,NUCKS1,R | 34.42 | 939356 | 3.66E-05 |
| 2:430975 | NA | 42.25 | 939356 | 4.50E-05 |
| 2:27730940 | GTF3C2,EIF2B4,SNX | 194.14 | 939356 | 2.07E-04 |
| 2:27860551 | GTF3C2,EIF2B4,SNX | 34.42 | 939356 | 3.66E-05 |
| 2:45155276 | LINC01833,SIX3 | 86.72 | 939356 | 9.23E-05 |
| 2:45159091 | LINC01833,SIX3-AS1 | 31.36 | 939356 | 3.34E-05 |
| 2:63581507 | EHBP1,OTX1,WDPCP | 40.64 | 939356 | 4.33E-05 |
| 2:74334462 | TET3 | 34.42 | 939356 | 3.66E-05 |
| 2:98275354 | ZAP70,TMEM131,AC | 44.79 | 939356 | 4.77E-05 |
| 2:144225215 | ARHGAP15 | 52.8 | 939356 | 5.62E-05 |
| 2:147956293 | NA | 34.48 | 939356 | 3.67E-05 |
| 2:225475560 | CUL3 | 40.96 | 939356 | 4.36E-05 |
| 3:70968431 | FOXP1 | 41.44 | 939356 | 4.41E-05 |
| 3:85457240 | CADM2 | 92.16 | 939356 | 9.81E-05 |
| 3:93994255 | PROS1,ARL13B,ARL | 34.42 | 939356 | 3.66E-05 |
| 3:131576287 | CPNE4 | 30.86 | 939356 | 3.29E-05 |
| 3:141124607 | ZBTB38,RASA2 | 35.2 | 939356 | 3.75E-05 |
| 3:141267295 | ZBTB38,RASA2 | 30.99 | 939356 | 3.30E-05 |
| 3:158187811 | SHOX2,RSRC1,UC.13 | 28.44 | 939356 | 3.03E-05 |
| 4:3446091 | RGS12,HGFAC,DOK7 | 31.04 | 939356 | 3.30E-05 |
| 4:39406254 | NA | 31.36 | 939356 | 3.34E-05 |
| 4:39414993 | RFC1,KLB | 298.14 | 939356 | 3.17E-04 |
| 4:42151306 | LINC00682,TMEM33, | 39.62 | 939356 | 4.22E-05 |
| 4:99678691 | NA | 33.32 | 939356 | 3.55E-05 |
| 4:99713350 | TSPAN5,METAP1,LO | 50.19 | 939356 | 5.34E-05 |
| 4:100000136 | ADH5,LOC100507053 | 70.24 | 939356 | 7.48E-05 |
| 4:100239319 | METAP1,ADH1B | 1489.17 | 939356 | 1.58E-03 |
| 4:100254520 | ADH4|LOC100507053 | 71.48 | 939356 | 7.61E-05 |
| 4:100260679 | NA | 116.16 | 939356 | 1.24E-04 |
| 4:100273594 | LOC100507053,ADH1 | 37.62 | 939356 | 4.00E-05 |
| 4:100286085 | ADH1C | 92.64 | 939356 | 9.86E-05 |
| 4:100444363 | ADH7,C4orf17 | 139.8 | 939356 | 1.49E-04 |

| 4:101243023 | EMCN | 55.35 | 939356 | 5.89E-05 |
| --- | --- | --- | --- | --- |
| 4:103188709 | BANK1,SLC39A8,LO | 96.46 | 939356 | 1.03E-04 |
| 4:143648579 | INPP4B | 45.56 | 939356 | 4.85E-05 |
| 4:152968372 | LINC02273 | 32.35 | 939356 | 3.44E-05 |
| 4:171086393 | LINC01612 | 32.87 | 939356 | 3.50E-05 |
| 5:87854395 | TMEM161B,TMEM16 | 44.44 | 939356 | 4.73E-05 |
| 5:144412335 | NA | 30.62 | 939356 | 3.26E-05 |
| 5:155902003 | SGCD | 34.6 | 939356 | 3.68E-05 |
| 5:166803321 | TENM2 | 31.36 | 939356 | 3.34E-05 |
| 7:69783020 | AUTS2 | 36 | 939356 | 3.83E-05 |
| 7:73042443 | BAZ1B,BCL7B,TBL2, | 41.53 | 939356 | 4.42E-05 |
| 7:98977515 | MYH16,ARPC1A,ARP | 37.65 | 939356 | 4.01E-05 |
| 7:103840115 | ORC5 | 29.16 | 939356 | 3.10E-05 |
| 7:153489744 | NA | 77.44 | 939356 | 8.24E-05 |
| 8:20949917 | NA | 29.04 | 939356 | 3.09E-05 |
| 8:64527399 | LOC102724612,LINC | 41.23 | 939356 | 4.39E-05 |
| 8:126500031 | NA | 36.8 | 939356 | 3.92E-05 |
| 9:108755622 | NA | 31.23 | 939356 | 3.32E-05 |
| 9:109345993 | LINC01505 | 42.25 | 939356 | 4.50E-05 |
| 10:110507806 | NA | 30.57 | 939356 | 3.25E-05 |
| 10:125093880 | NA | 33.64 | 939356 | 3.58E-05 |
| 11:8642218 | STK33,TRIM66 | 42.68 | 939356 | 4.54E-05 |
| 11:27643725 | LGR4,LOC105376671 | 31.12 | 939356 | 3.31E-05 |
| 11:47397353 | ACP2,MADD,MYBPC | 52.56 | 939356 | 5.60E-05 |
| 11:113424042 | DRD2 | 41.44 | 939356 | 4.41E-05 |
| 11:113660576 | TMPRSS5,ZW10,USP | 28.44 | 939356 | 3.03E-05 |
| 11:116075001 | NA | 40.96 | 939356 | 4.36E-05 |
| 11:121544285 | NA | 29.88 | 939356 | 3.18E-05 |
| 11:133658168 | LOC646522 | 30.62 | 939356 | 3.26E-05 |
| 12:51895882 | GALNT6,SLC4A8 | 28.44 | 939356 | 3.03E-05 |
| 12:54674235 | CBX5,HNRNPA1|HN | 36.8 | 939356 | 3.92E-05 |
| 12:81601464 | ACSS3,PPFIA2,PPFIA | 36 | 939356 | 3.83E-05 |
| 12:92170791 | NA | 34.42 | 939356 | 3.66E-05 |
| 13:27124360 | WASF3 | 32.56 | 939356 | 3.47E-05 |
| 14:57274519 | OTX2,OTX2-AS1 | 35.2 | 939356 | 3.75E-05 |
| 14:58782779 | ACTR10,PSMA3,PSM | 33.75 | 939356 | 3.59E-05 |
| 14:94844947 | SERPINA10,SERPINA | 46.44 | 939356 | 4.94E-05 |
| 14:104610138 | ASPG,KIF26A | 31.89 | 939356 | 3.39E-05 |
| 15:75027880 | COX5A,PPCDC | 38.88 | 939356 | 4.14E-05 |
| 15:86796012 | AGBL1,AGBL1|AGBL | 32.11 | 939356 | 3.42E-05 |
| 16:20013793 | GPR139 | 38.29 | 939356 | 4.08E-05 |
| 16:24810681 | LINC01567|TNRC6A, | 55.25 | 939356 | 5.88E-05 |
| 16:28754684 | SBK1,EIF3C|EIF3CL, | 55.75 | 939356 | 5.93E-05 |
| 16:29892184 | CDIPT-AS1,SEZ6L2 | 40.64 | 939356 | 4.33E-05 |
| 16:64872590 | NA | 41.23 | 939356 | 4.39E-05 |
| 16:69131281 | HAS3,CHTF8,UTP4,S | 30.8 | 939356 | 3.28E-05 |
| 16:72338507 | LINC01572 | 46.13 | 939356 | 4.91E-05 |
| 16:73912588 | NA | 53.78 | 939356 | 5.72E-05 |
| 17:2209888 | SMG6,LOC101927839 | 31.36 | 939356 | 3.34E-05 |
| 17:7462969 | SENP3|SENP3-EIF4A | 40.11 | 939356 | 4.27E-05 |
| 17:29715500 | NF1,EVI2B,EVI2B|NF | 37.62 | 939356 | 4.00E-05 |
| 17:78524597 | RPTOR | 31.64 | 939356 | 3.37E-05 |
| 18:53052169 | TCF4,TCF4|TCF4-AS1 | 36.8 | 939356 | 3.92E-05 |
| 18:55080437 | NA | 30.62 | 939356 | 3.26E-05 |
| 19:49214274 | FAM83E,SEC1P,NTN | 83.42 | 939356 | 8.88E-05 |
| 20:25035711 | ACSS1,VSX1 | 32.87 | 939356 | 3.50E-05 |
| 22:41946519 | EP300,EP300-AS1,L3 | 28.82 | 939356 | 3.07E-05 |
| 2:103998526 | MRPS9 | 20.3 | 2076 | 9.69E-03 |

| 10:83003759 | NRG3 | 20.97 | 2076 | 1.00E-02 |
| --- | --- | --- | --- | --- |
| 9:116739671 | TNC | 22.95 | 2076 | 1.09E-02 |
| 10:103929295 | PPRC1 | 21.29 | 2076 | 1.02E-02 |
| 11:29933817 | FSHB | 20.13 | 2076 | 9.61E-03 |
| 11:134231086 | LOC8994 | 23.28 | 2076 | 1.11E-02 |
| 5:117598798 | DMXL1 | 23.64 | 2076 | 1.13E-02 |
| 11:31005259 | DCDC1 | 19.88 | 2076 | 9.50E-03 |
| 18:47967754 | MEX3C | 20.15 | 2076 | 9.62E-03 |
| 2:239151212 | TRAF3IP1 | 20.9 | 2076 | 9.98E-03 |
| 12:10153542 | CLEC1A | 19.75 | 2076 | 9.43E-03 |
| 18:47962958 | MEX3C | 20.87 | 2076 | 9.96E-03 |
| 19:37696665 | PDCD5 | 19.98 | 2076 | 9.54E-03 |
| 5:78355394 | ARSB | 28.46 | 2076 | 1.35E-02 |
| 13:108375283 | TNFSF13 | 20.54 | 2076 | 9.81E-03 |
| 10:61197033 | SLC16A9 | 28.6 | 2076 | 1.36E-02 |
| 10:61147703 | CCDC6 | 29.21 | 2076 | 1.39E-02 |
| 10:61137188 | CCDC6 | 111.15 | 2076 | 5.09E-02 |
| 4:22769759 | LOC6437 | 20.43 | 2076 | 9.75E-03 |
| 10:61016188 | CCDC6 | 20.18 | 2076 | 9.63E-03 |
| 5:131752849 | LOC4411 | 21 | 2076 | 1.00E-02 |
| 1:158071277 | FCRL6 | 19.86 | 2076 | 9.49E-03 |
| 11:125488674 | RPUSD4 | 20.12 | 2076 | 9.61E-03 |
| 20:8926063 | PLCB1 | 22.06 | 2076 | 1.05E-02 |
| 7:19788530 | TWISTNB | 24.49 | 2076 | 1.17E-02 |
| 2:45795867 | SRBD1 | 19.57 | 2076 | 9.35E-03 |
| 3:5284556 | ARL8B | 26.21 | 2076 | 1.25E-02 |
| 7:106769006 | PIK3CG | 47.95 | 29255 | 1.64E-03 |
| 4:147472512 | EDNRA | 46.08 | 29255 | 1.57E-03 |
| 8:106016051 | ZFPM2 | 19.71 | 2076 | 9.41E-03 |
| 9:97975154 | SLC35D2 | 20.23 | 2076 | 9.66E-03 |
| 10:59912647 | TFAM | 19.97 | 2076 | 9.54E-03 |
| 16:78024057 | WWOX | 20.56 | 2076 | 9.82E-03 |
| 12:90546484 | BTG1 | 23.3 | 2076 | 1.11E-02 |
| 9:38478919 | ALDH1B1 | 21.27 | 2076 | 1.02E-02 |
| 13:75564535 | UCHL3 | 20.19 | 2076 | 9.64E-03 |
| 13:68851492 | DACH1 | 22 | 2076 | 1.05E-02 |
| 2:27508073 | GCKR | 16 | 91462 | 1.75E-04 |
| 4:88117930 | ABCG2 | 36 | 91462 | 3.93E-04 |
| 7:17244953 | AHR | 100 | 91462 | 1.09E-03 |
| 7:17247482 | AHR | 100 | 91462 | 1.09E-03 |
| 7:73621527 | MLXIPL | 25 | 91462 | 2.73E-04 |
| 7:75986787 | POR | 49 | 91462 | 5.35E-04 |
| 11:27658369 | BDNF | 16 | 91462 | 1.75E-04 |
| 15:74727108 | CYP1A1 | 144 | 91462 | 1.57E-03 |
| 15:74735539 | CYP1A2 | 196 | 91462 | 2.14E-03 |
| 17:30022077 | EFCAB5 | 9 | 91462 | 9.84E-05 |
| 8:122395852 | ZHX2 | 42.25 | 41295 | 1.02E-03 |
| 19:44912383 | APOC1 | 31.04 | 41295 | 7.51E-04 |
| 8:10813762 | PINX1 | 31.04 | 41295 | 7.51E-04 |
| 1:197727642 | DENND1B | 3.17 | 51523 | 6.16E-05 |
| 2:25492467 | DNMT3A | 12.04 | 51523 | 2.34E-04 |
| 2:27742603 | GCKR | 33.1 | 51523 | 6.42E-04 |
| 2:43806918 | THADA | 28.59 | 51523 | 5.55E-04 |
| 2:61224259 | C2orf74, REL | 34.2 | 51523 | 6.63E-04 |
| 2:103054449 | IL18RAP, IL12RL2, IL | 46.56 | 51523 | 9.03E-04 |
| 2:198896895 | PLCL1 | 7.29 | 51523 | 1.41E-04 |
| 2:231116874 | SP140 | 20.54 | 51523 | 3.98E-04 |
| 3:18706858 | NA | 10.65 | 51523 | 2.07E-04 |

| 5:72551134 | TMEM174 | 24.69 | 51523 | 4.79E-04 |
| --- | --- | --- | --- | --- |
| 5:96244549 | ERAP2, LRAP | 8.34 | 51523 | 1.62E-04 |
| 5:141479065 | NDFIP1 | 7.29 | 51523 | 1.41E-04 |
| 5:173279842 | CPEB4 | 16.6 | 51523 | 3.22E-04 |
| 6:3433318 | C6orf85 | 14.08 | 51523 | 2.73E-04 |
| 8:129567181 | NA | 59.22 | 51523 | 1.15E-03 |
| 9:139266496 | CARD9, CARD9,SNA | 72.03 | 51523 | 1.40E-03 |
| 10:6102012 | IL2RA | 16.89 | 51523 | 3.28E-04 |
| 10:59913151 | UBE2D1 | 45.84 | 51523 | 8.89E-04 |
| 11:61557803 | FADS1 | 16.6 | 51523 | 3.22E-04 |
| 11:64097233 | PRDX5, ESRRA | 14.08 | 51523 | 2.73E-04 |
| 13:43052880 | TNFSF11 | 16.87 | 51523 | 3.27E-04 |
| 14:69210199 | ZFP36L1 | 16.63 | 51523 | 3.23E-04 |
| 14:88472595 | GALC, GPR65 | 44.59 | 51523 | 8.65E-04 |
| 15:67442596 | SMAD3 | 30.25 | 51523 | 5.87E-04 |
| 17:32593665 | CCL2, CCL7 | 51.11 | 51523 | 9.91E-04 |
| 19:33757062 | NA | 7.29 | 51523 | 1.41E-04 |
| 19:49214274 | FUT2, RASIP1 | 16.63 | 51523 | 3.23E-04 |
| 20:62349586 | RTEL1, TNFRSF6B, S | 17.1 | 51523 | 3.32E-04 |
| 22:30592487 | MTMR3 | 13.19 | 51523 | 2.56E-04 |
| 22:39659773 | MAP3K7IP1 | 69.11 | 51523 | 1.34E-03 |
| 1:67705958 | IL23R | 255.53 | 51523 | 4.94E-03 |
| 1:114377568 | PTPN22 | 32.88 | 51523 | 6.38E-04 |
| 1:160830268 | CD244, CD244, ITLN1 | 27.62 | 51523 | 5.36E-04 |
| 1:172853460 | TNFSF18, TNFSF4, F | 62.79 | 51523 | 1.22E-03 |
| 1:200877562 | C1orf106, KIF21B | 28.13 | 51523 | 5.46E-04 |
| 2:234184417 | ATG16L1 | 196.13 | 51523 | 3.79E-03 |
| 3:49721532 | MST1,GPX1,BSN | 74.14 | 51523 | 1.44E-03 |
| 5:40410584 | PTGER4 | 153.76 | 51523 | 2.98E-03 |
| 5:131784393 | SLC22A4, SLC22A5,I | 99.04 | 51523 | 1.92E-03 |
| 5:150270420 | IRGM | 79.53 | 51523 | 1.54E-03 |
| 5:158787385 | IL12B | 48.76 | 51523 | 9.46E-04 |
| 6:20728731 | CDKAL1 | 35.91 | 51523 | 6.96E-04 |
| 6:31542308 | MCCD1, LTA, HLA-D | 45.84 | 51523 | 8.89E-04 |
| 6:106435025 | PRDM1 | 23.89 | 51523 | 4.64E-04 |
| 6:167406633 | CCR6 | 51.87 | 51523 | 1.01E-03 |
| 7:50304461 | IKZF1, ZPBP, FIGNL1 | 28.59 | 51523 | 5.55E-04 |
| 8:126537570 | NA | 43.15 | 51523 | 8.37E-04 |
| 9:4981602 | JAK2 | 58.71 | 51523 | 1.14E-03 |
| 9:117552885 | TNFSF15, TNFSF8 | 56.76 | 51523 | 1.10E-03 |
| 10:35535695 | CREM | 39.66 | 51523 | 7.69E-04 |
| 10:64445564 | ZNF365 | 83.16 | 51523 | 1.61E-03 |
| 10:101284237 | NKX2-3 | 90.57 | 51523 | 1.75E-03 |
| 11:76301375 | C11orf30 | 51.87 | 51523 | 1.01E-03 |
| 12:40792300 | MUC19, LRRK2 | 89.34 | 51523 | 1.73E-03 |
| 13:44457925 | C13orf31 | 43.15 | 51523 | 8.37E-04 |
| 16:50756881 | NOD2 | 330.34 | 51523 | 6.37E-03 |
| 17:38040763 | GSMDL, ZPBP2, OR | M34.2 | 51523 | 6.63E-04 |
| 17:40570772 | MLX, STAT3 | 33.1 | 51523 | 6.42E-04 |
| 18:12809340 | PTPN2 | 60.85 | 51523 | 1.18E-03 |
| 19:1124835 | GPX4, SBNO2 | 37.3 | 51523 | 7.23E-04 |
| 21:16812552 | NA | 45.5 | 51523 | 8.82E-04 |
| 21:45615023 | ICOSLG | 58.71 | 51523 | 1.14E-03 |
| 1:156321154 | ZNF644 | 38.44 | 352566 | 1.09E-04 |
| 2:629881 | TMEM18 | 38.44 | 352566 | 1.09E-04 |
| 2:88438050 | FABP1 | 30.25 | 352566 | 8.58E-05 |
| 2:102744854 | IL1R1 | 45.56 | 352566 | 1.29E-04 |
| 2:214033530 | IKZF2 | 39.06 | 352566 | 1.11E-04 |

| 3:49891885 | TRAIP | 39.06 | 352566 | 1.11E-04 |
| --- | --- | --- | --- | --- |
| 5:172191052 | DUSP1 | 31.36 | 352566 | 8.89E-05 |
| 6:32591588 | HLA-DQA1 | 70.56 | 352566 | 2.00E-04 |
| 6:116314634 | FRK | 33.06 | 352566 | 9.38E-05 |
| 6:126851160 | C6orf173 | 39.06 | 352566 | 1.11E-04 |
| 6:130371227 | L3MBTL3 | 42.25 | 352566 | 1.20E-04 |
| 7:22759469 | IL6 | 49 | 352566 | 1.39E-04 |
| 7:36084529 | KIAA1706 | 27.56 | 352566 | 7.82E-05 |
| 8:117007850 | TRPS1 | 45.56 | 352566 | 1.29E-04 |
| 8:126344208 | NSMCE2 | 25 | 352566 | 7.09E-05 |
| 9:136142355 | ABO | 33.06 | 352566 | 9.38E-05 |
| 10:91007360 | LIPA | 36 | 352566 | 1.02E-04 |
| 11:13357183 | ARNTL | 42.25 | 352566 | 1.20E-04 |
| 11:47312892 | MADD | 60.06 | 352566 | 1.70E-04 |
| 11:60021948 | MS4A4A | 30.25 | 352566 | 8.58E-05 |
| 11:72496148 | STARD10 | 30.25 | 352566 | 8.58E-05 |
| 11:95855385 | METAP2 | 49 | 352566 | 1.39E-04 |
| 14:73011885 | RGS6 | 76.56 | 352566 | 2.17E-04 |
| 15:51745277 | DMXL2 | 30.25 | 352566 | 8.58E-05 |
| 16:53803574 | FTO | 72.25 | 352566 | 2.05E-04 |
| 17:16097430 | NCOR1 | 25 | 352566 | 7.09E-05 |
| 17:72699833 | CD300LF, RAB37 | 54.76 | 352566 | 1.55E-04 |
| 18:55080437 | ONECUT2 | 45.56 | 352566 | 1.29E-04 |
| 18:57897803 | MC4R | 36 | 352566 | 1.02E-04 |
| 20:62343956 | ZGPAT | 33.06 | 352566 | 9.38E-05 |
| 21:40465534 | DSCR2 | 115.56 | 352566 | 3.28E-04 |
| 22:39074737 | TOMM22 | 49 | 352566 | 1.39E-04 |
| 1:27180088 | ZDHHC18 | 37.73 | 352566 | 1.07E-04 |
| 3:170705693 | EIF5A2 | 45.56 | 352566 | 1.29E-04 |
| 14:94838142 | SERPINA1, SERPINA | 39.62 | 352566 | 1.12E-04 |
| 15:53728154 | ONECUT1 | 39.06 | 352566 | 1.11E-04 |
| 3:135932359 | MSL2 | 36 | 352566 | 1.02E-04 |
| 7:74122854 | GTF2I | 31.36 | 352566 | 8.89E-05 |
| 8:11664738 | FDFT1 | 52.56 | 352566 | 1.49E-04 |
| 22:41339367 | XPNPEP3 | 30.25 | 352566 | 8.58E-05 |
| 1:10730910 | CASZ1 | 36 | 783978 | 4.59E-05 |
| 1:15912987 | AGMAT | 128.44 | 783978 | 1.64E-04 |
| 1:16556786 | RSG1 | 58.78 | 783978 | 7.50E-05 |
| 1:18809916 | KLHDC7A | 75.11 | 783978 | 9.58E-05 |
| 1:23699340 | ZNF436-AS1 | 121 | 783978 | 1.54E-04 |
| 1:46037394 | AKR1A1 | 45.56 | 783978 | 5.81E-05 |
| 1:46581933 | PIK3R3 | 29.16 | 783978 | 3.72E-05 |
| 1:48002447 | FOXD2 | 36 | 783978 | 4.59E-05 |
| 1:55718708 | MIR4422HG | 39.06 | 783978 | 4.98E-05 |
| 1:56615809 | LINC01755 | 49 | 783978 | 6.25E-05 |
| 1:78023173 | AK5 | 27.56 | 783978 | 3.52E-05 |
| 1:78707493 | MGC27382 | 49 | 783978 | 6.25E-05 |
| 1:82957871 | LINC01362 | 40.11 | 783978 | 5.12E-05 |
| 1:94050911 | BCAR3 | 38.44 | 783978 | 4.90E-05 |
| 1:100808363 | CDC14A | 44.44 | 783978 | 5.67E-05 |
| 1:110012289 | SYPL2 | 56.25 | 783978 | 7.17E-05 |
| 1:113258293 | PPM1J | 116.64 | 783978 | 1.49E-04 |
| 1:150159616 | LOC105371433 | 36 | 783978 | 4.59E-05 |
| 1:150940625 | CERS2 | 144 | 783978 | 1.84E-04 |
| 1:155155731 | TRIM46 | 44.44 | 783978 | 5.67E-05 |
| 1:163738950 | LOC100422212 | 40.11 | 783978 | 5.12E-05 |
| 1:170649277 | PRRX1 | 44.44 | 783978 | 5.67E-05 |
| 1:171455322 | PRRC2C | 40.96 | 783978 | 5.22E-05 |

| 1:172346548 | DNM3 | 40.11 | 783978 | 5.12E-05 |
| --- | --- | --- | --- | --- |
| 1:180905694 | KIAA1614 | 44.44 | 783978 | 5.67E-05 |
| 1:184672098 | EDEM3 | 56.25 | 783978 | 7.17E-05 |
| 1:186769572 | PLA2G4A | 36 | 783978 | 4.59E-05 |
| 1:201016296 | CACNA1S | 84.64 | 783978 | 1.08E-04 |
| 1:205537858 | MFSD4A | 44.44 | 783978 | 5.67E-05 |
| 1:207231751 | PFKFB2 | 40.11 | 783978 | 5.12E-05 |
| 1:208039431 | CD34 | 41.33 | 783978 | 5.27E-05 |
| 1:214744893 | PTPN14 | 27.56 | 783978 | 3.52E-05 |
| 1:220224321 | RNU5F-1 | 44.44 | 783978 | 5.67E-05 |
| 1:221001142 | LINC01352 | 36 | 783978 | 4.59E-05 |
| 1:228532195 | OBSCN | 36 | 783978 | 4.59E-05 |
| 1:243469669 | SDCCAG8 | 64 | 783978 | 8.16E-05 |
| 2:226933 | SH3YL1 | 53.78 | 783978 | 6.86E-05 |
| 2:15782471 | DDX1 | 113.78 | 783978 | 1.45E-04 |
| 2:18676276 | RDH14 | 64 | 783978 | 8.16E-05 |
| 2:27742603 | GCKR | 215.11 | 783978 | 2.74E-04 |
| 2:40680149 | SLC8A1 | 33.06 | 783978 | 4.22E-05 |
| 2:43433257 | ZFP36L2 | 64 | 783978 | 8.16E-05 |
| 2:44390369 | PPM1B | 27.56 | 783978 | 3.52E-05 |
| 2:54885640 | SPTBN1 | 64 | 783978 | 8.16E-05 |
| 2:67874553 | LINC01812 | 36 | 783978 | 4.59E-05 |
| 2:73895765 | ALMS1P1 | 217.56 | 783978 | 2.77E-04 |
| 2:103166325 | SLC9A4 | 27.56 | 783978 | 3.52E-05 |
| 2:113967075 | PSD4 | 69.44 | 783978 | 8.86E-05 |
| 2:121306440 | LINC01101 | 53.78 | 783978 | 6.86E-05 |
| 2:121988884 | TFCP2L1 | 67.24 | 783978 | 8.58E-05 |
| 2:137074132 | CXCR4 | 40.11 | 783978 | 5.12E-05 |
| 2:148759656 | ORC4 | 64 | 783978 | 8.16E-05 |
| 2:152387553 | NEB | 44.44 | 783978 | 5.67E-05 |
| 2:159810691 | TANC1 | 40.11 | 783978 | 5.12E-05 |
| 2:169995581 | LRP2 | 83.27 | 783978 | 1.06E-04 |
| 2:176993583 | HOXD8 | 82.64 | 783978 | 1.05E-04 |
| 2:178121524 | NFE2L2 | 93.44 | 783978 | 1.19E-04 |
| 2:183077254 | PDE1A | 44.44 | 783978 | 5.67E-05 |
| 2:188168567 | CALCRL | 45.56 | 783978 | 5.81E-05 |
| 2:210854476 | UNC80 | 32.11 | 783978 | 4.10E-05 |
| 2:211540507 | CPS1 | 264.06 | 783978 | 3.37E-04 |
| 2:212274447 | ERBB4 | 40.11 | 783978 | 5.12E-05 |
| 2:217665788 | TNP1 | 144 | 783978 | 1.84E-04 |
| 2:219286541 | VIL1 | 27.56 | 783978 | 3.52E-05 |
| 2:220358198 | SPEG | 75.11 | 783978 | 9.58E-05 |
| 2:227287718 | MIR5702 | 36 | 783978 | 4.59E-05 |
| 2:230665303 | TRIP12 | 40.11 | 783978 | 5.12E-05 |
| 2:234257105 | SAG | 36 | 783978 | 4.59E-05 |
| 3:12208671 | SYN2 | 44.44 | 783978 | 5.67E-05 |
| 3:13947504 | WNT7A | 32.11 | 783978 | 4.10E-05 |
| 3:30749965 | TGFBR2 | 32.11 | 783978 | 4.10E-05 |
| 3:38527215 | ACVR2B | 100 | 783978 | 1.28E-04 |
| 3:48443816 | PLXNB1 | 39.06 | 783978 | 4.98E-05 |
| 3:49572140 | DAG1 | 33.06 | 783978 | 4.22E-05 |
| 3:50457737 | CACNA2D2 | 30.25 | 783978 | 3.86E-05 |
| 3:51593113 | RAD54L2 | 28.44 | 783978 | 3.63E-05 |
| 3:53020544 | SFMBT1 | 49 | 783978 | 6.25E-05 |
| 3:63974477 | ATXN7 | 49 | 783978 | 6.25E-05 |
| 3:105455955 | CBLB | 40.11 | 783978 | 5.12E-05 |
| 3:121657593 | SLC15A2 | 75.11 | 783978 | 9.58E-05 |
| 3:125906237 | ALDH1L1-AS2 | 44.44 | 783978 | 5.67E-05 |

| 3:135932494 | MSL2 | 39.06 | 783978 | 4.98E-05 |
| --- | --- | --- | --- | --- |
| 3:136536835 | SLC35G2 | 36 | 783978 | 4.59E-05 |
| 3:141093285 | ZBTB38 | 32.11 | 783978 | 4.10E-05 |
| 3:141750810 | TFDP2 | 205.44 | 783978 | 2.62E-04 |
| 3:168888112 | MECOM | 36 | 783978 | 4.59E-05 |
| 3:170070702 | SKIL | 31.36 | 783978 | 4.00E-05 |
| 3:185354216 | SENP2 | 93.44 | 783978 | 1.19E-04 |
| 3:186432839 | KNG1 | 75.11 | 783978 | 9.58E-05 |
| 3:193811168 | LINC02028 | 30.25 | 783978 | 3.86E-05 |
| 4:3449781 | HGFAC | 42.25 | 783978 | 5.39E-05 |
| 4:10090930 | WDR1 | 36 | 783978 | 4.59E-05 |
| 4:23743962 | PPARGC1A | 41.33 | 783978 | 5.27E-05 |
| 4:52686513 | DCUN1D4 | 33.06 | 783978 | 4.22E-05 |
| 4:77401452 | SHROOM3 | 592.11 | 783978 | 7.55E-04 |
| 4:81182554 | FGF5 | 136.11 | 783978 | 1.74E-04 |
| 4:103698786 | LOC102723704 | 87.11 | 783978 | 1.11E-04 |
| 4:109703549 | ETNPPL | 40.11 | 783978 | 5.12E-05 |
| 4:115498457 | UGT8 | 39.06 | 783978 | 4.98E-05 |
| 5:676962 | TPPP | 64 | 783978 | 8.16E-05 |
| 5:34504277 | RAI14 | 81 | 783978 | 1.03E-04 |
| 5:39378115 | DAB2 | 266.78 | 783978 | 3.40E-04 |
| 5:39950266 | LINC00603 | 81 | 783978 | 1.03E-04 |
| 5:44190757 | FGF10 | 31.36 | 783978 | 4.00E-05 |
| 5:44812566 | MRPS30 | 32.11 | 783978 | 4.10E-05 |
| 5:52787358 | FST | 40.11 | 783978 | 5.12E-05 |
| 5:53298716 | ARL15 | 125.44 | 783978 | 1.60E-04 |
| 5:67750213 | PIK3R1 | 125.44 | 783978 | 1.60E-04 |
| 5:68656327 | AK6 | 36 | 783978 | 4.59E-05 |
| 5:76847499 | WDR41 | 40.11 | 783978 | 5.12E-05 |
| 5:78322650 | DMGDH | 40.11 | 783978 | 5.12E-05 |
| 5:131671662 | SLC22A4 | 30.86 | 783978 | 3.94E-05 |
| 5:132226669 | AFF4 | 52.56 | 783978 | 6.70E-05 |
| 5:148524820 | ABLIM3 | 40.11 | 783978 | 5.12E-05 |
| 5:176813404 | SLC34A1 | 264.06 | 783978 | 3.37E-04 |
| 6:7118990 | RREB1 | 45.56 | 783978 | 5.81E-05 |
| 6:24354045 | DCDC2 | 36 | 783978 | 4.59E-05 |
| 6:31826705 | NEU1 | 49 | 783978 | 6.25E-05 |
| 6:34180297 | HMGA1 | 34.81 | 783978 | 4.44E-05 |
| 6:41690823 | TFEB | 64 | 783978 | 8.16E-05 |
| 6:43287722 | CRIP3 | 58.78 | 783978 | 7.50E-05 |
| 6:43806609 | LINC01512 | 324 | 783978 | 4.13E-04 |
| 6:44765535 | SUPT3H | 27.56 | 783978 | 3.52E-05 |
| 6:51492862 | PKHD1 | 36 | 783978 | 4.59E-05 |
| 6:52630153 | GSTA2 | 44.44 | 783978 | 5.67E-05 |
| 6:55422618 | HMGCLL1 | 36 | 783978 | 4.59E-05 |
| 6:90118764 | RRAGD | 36 | 783978 | 4.59E-05 |
| 6:100894587 | SIM1 | 40.11 | 783978 | 5.12E-05 |
| 6:107172979 | LOC100422737 | 33.64 | 783978 | 4.29E-05 |
| 6:109008158 | FOXO3 | 64 | 783978 | 8.16E-05 |
| 6:130374461 | L3MBTL3 | 45.56 | 783978 | 5.81E-05 |
| 6:131882078 | ARG1 | 60.06 | 783978 | 7.66E-05 |
| 6:133849789 | EYA4 | 69.44 | 783978 | 8.86E-05 |
| 6:154986664 | SCAF8 | 45.56 | 783978 | 5.81E-05 |
| 6:160633107 | SLC22A2 | 289 | 783978 | 3.68E-04 |
| 7:1286567 | UNCX | 225 | 783978 | 2.87E-04 |
| 7:17287106 | AHR | 40.11 | 783978 | 5.12E-05 |
| 7:25775690 | MIR148A | 44.44 | 783978 | 5.67E-05 |
| 7:32930876 | KBTBD2 | 53.78 | 783978 | 6.86E-05 |

| 7:41068572 | LINC01450 | 41.33 | 783978 | 5.27E-05 |
| --- | --- | --- | --- | --- |
| 7:45257483 | RAMP3 | 25 | 783978 | 3.19E-05 |
| 7:46753684 | LOC730338 | 106.78 | 783978 | 1.36E-04 |
| 7:50739610 | GRB10 | 60.84 | 783978 | 7.76E-05 |
| 7:65337902 | VKORC1L1 | 40.11 | 783978 | 5.12E-05 |
| 7:66030612 | GS1-124K5.11 | 44.44 | 783978 | 5.67E-05 |
| 7:75612803 | POR | 58.78 | 783978 | 7.50E-05 |
| 7:77422583 | TMEM60 | 136.11 | 783978 | 1.74E-04 |
| 7:127505755 | SND1 | 30.25 | 783978 | 3.86E-05 |
| 7:128576086 | IRF5 | 100 | 783978 | 1.28E-04 |
| 7:129564134 | UBE2H | 45.56 | 783978 | 5.81E-05 |
| 7:151415536 | PRKAG2 | 289 | 783978 | 3.68E-04 |
| 7:155665959 | SHH | 69.44 | 783978 | 8.86E-05 |
| 7:156252939 | LINC01006 | 93.44 | 783978 | 1.19E-04 |
| 8:8142575 | PRAG1 | 58.78 | 783978 | 7.50E-05 |
| 8:8684953 | MFHAS1 | 69.44 | 783978 | 8.86E-05 |
| 8:9297246 | LOC157273 | 42.25 | 783978 | 5.39E-05 |
| 8:10190040 | MSRA | 30.25 | 783978 | 3.86E-05 |
| 8:10841858 | XKR6 | 25 | 783978 | 3.19E-05 |
| 8:11417493 | BLK | 49 | 783978 | 6.25E-05 |
| 8:22495966 | BIN3 | 40.11 | 783978 | 5.12E-05 |
| 8:23748420 | STC1 | 205.44 | 783978 | 2.62E-04 |
| 8:32435620 | NRG1 | 36 | 783978 | 4.59E-05 |
| 8:61384753 | RAB2A | 36 | 783978 | 4.59E-05 |
| 8:76483239 | HNF4G | 36 | 783978 | 4.59E-05 |
| 8:87332552 | WWP1 | 69.44 | 783978 | 8.86E-05 |
| 8:120886486 | DEPTOR | 30.25 | 783978 | 3.86E-05 |
| 8:126476873 | TRIB1 | 64 | 783978 | 8.16E-05 |
| 9:20554583 | MLLT3 | 27.04 | 783978 | 3.45E-05 |
| 9:33169034 | B4GALT1-AS1 | 25 | 783978 | 3.19E-05 |
| 9:33956791 | UBAP2 | 53.78 | 783978 | 6.86E-05 |
| 9:71432174 | PIP5K1B | 215.11 | 783978 | 2.74E-04 |
| 9:119324929 | ASTN2 | 58.78 | 783978 | 7.50E-05 |
| 9:136932706 | BRD3 | 27.56 | 783978 | 3.52E-05 |
| 9:139100413 | QSOX2 | 33.06 | 783978 | 4.22E-05 |
| 9:140103272 | NDOR1 | 36 | 783978 | 4.59E-05 |
| 10:899071 | LARP4B | 169 | 783978 | 2.16E-04 |
| 10:29781798 | SVIL | 36 | 783978 | 4.59E-05 |
| 10:35150364 | PARD3-AS1 | 40.11 | 783978 | 5.12E-05 |
| 10:51026705 | PARG | 44.44 | 783978 | 5.67E-05 |
| 10:52646093 | A1CF | 85.56 | 783978 | 1.09E-04 |
| 10:63785089 | ARID5B | 44.44 | 783978 | 5.67E-05 |
| 10:69965177 | MYPN | 106.78 | 783978 | 1.36E-04 |
| 10:75016365 | DNAJC9-AS1 | 28.44 | 783978 | 3.63E-05 |
| 10:79252446 | KCNMA1 | 25 | 783978 | 3.19E-05 |
| 10:82209232 | TSPAN14 | 36 | 783978 | 4.59E-05 |
| 10:88880689 | FAM35A | 33.06 | 783978 | 4.22E-05 |
| 10:94839642 | CYP26A1 | 64 | 783978 | 8.16E-05 |
| 10:97278922 | SORBS1 | 30.25 | 783978 | 3.86E-05 |
| 10:104573017 | WBP1L | 42.25 | 783978 | 5.39E-05 |
| 10:105202318 | PDCD11 | 49 | 783978 | 6.25E-05 |
| 10:126446592 | EEF1AKMT2 | 69.44 | 783978 | 8.86E-05 |
| 11:2178330 | INS-IGF2 | 68.06 | 783978 | 8.68E-05 |
| 11:2789062 | KCNQ1 | 105.06 | 783978 | 1.34E-04 |
| 11:5578558 | OR52H1 | 52.56 | 783978 | 6.70E-05 |
| 11:9909127 | SBF2 | 40.11 | 783978 | 5.12E-05 |
| 11:30749090 | DCDC1 | 361 | 783978 | 4.60E-04 |
| 11:31424823 | DNAJC24 | 75.11 | 783978 | 9.58E-05 |

| 11:47427667 | SLC39A13 | 52.56 | 783978 | 6.70E-05 |
| --- | --- | --- | --- | --- |
| 11:48250675 | OR4B1 | 58.78 | 783978 | 7.50E-05 |
| 11:49057603 | TRIM49B | 42.25 | 783978 | 5.39E-05 |
| 11:50468801 | LOC646813 | 30.25 | 783978 | 3.86E-05 |
| 11:51421909 | OR4A5 | 33.64 | 783978 | 4.29E-05 |
| 11:55126517 | OR4A15 | 33.06 | 783978 | 4.22E-05 |
| 11:57409538 | MIR130A | 44.44 | 783978 | 5.67E-05 |
| 11:65552154 | MIR1234 | 121 | 783978 | 1.54E-04 |
| 11:68884755 | TPCN2 | 58.78 | 783978 | 7.50E-05 |
| 11:78023356 | GAB2 | 64 | 783978 | 8.16E-05 |
| 11:119326726 | USP2-AS1 | 44.44 | 783978 | 5.67E-05 |
| 11:121584931 | SORL1 | 44.44 | 783978 | 5.67E-05 |
| 12:364739 | SLC6A13 | 169 | 783978 | 2.16E-04 |
| 12:3392351 | TSPAN9 | 113.78 | 783978 | 1.45E-04 |
| 12:4616642 | C12orf4 | 33.64 | 783978 | 4.29E-05 |
| 12:12209203 | BCL2L14 | 42.25 | 783978 | 5.39E-05 |
| 12:15325031 | RERG | 72.25 | 783978 | 9.21E-05 |
| 12:48740855 | ZNF641 | 69.44 | 783978 | 8.86E-05 |
| 12:51161744 | ATF1 | 30.25 | 783978 | 3.86E-05 |
| 12:57751854 | R3HDM2 | 52.56 | 783978 | 6.70E-05 |
| 12:76271183 | PHLDA1 | 64 | 783978 | 8.16E-05 |
| 12:111696528 | CUX2 | 53.78 | 783978 | 6.86E-05 |
| 12:112553032 | NAA25 | 49 | 783978 | 6.25E-05 |
| 13:42749192 | DGKH | 49 | 783978 | 6.25E-05 |
| 13:50655989 | DLEU2 | 42.25 | 783978 | 5.39E-05 |
| 13:72348768 | DACH1 | 93.44 | 783978 | 1.19E-04 |
| 14:38008215 | MIPOL1 | 36 | 783978 | 4.59E-05 |
| 14:50735947 | L2HGDH | 32.4 | 783978 | 4.13E-05 |
| 14:81870100 | STON2 | 32.11 | 783978 | 4.10E-05 |
| 14:88829975 | SPATA7 | 44.44 | 783978 | 5.67E-05 |
| 14:93102251 | RIN3 | 33.64 | 783978 | 4.29E-05 |
| 14:100752644 | SLC25A29 | 40.11 | 783978 | 5.12E-05 |
| 15:39305443 | C15orf54 | 81 | 783978 | 1.03E-04 |
| 15:41399951 | INO80 | 68.06 | 783978 | 8.68E-05 |
| 15:45683795 | GATM | 802.78 | 783978 | 1.02E-03 |
| 15:53950578 | WDR72 | 169 | 783978 | 2.16E-04 |
| 15:57793765 | CGNL1 | 25 | 783978 | 3.19E-05 |
| 15:62808539 | MGC15885 | 40.11 | 783978 | 5.12E-05 |
| 15:63580155 | APH1B | 69.44 | 783978 | 8.86E-05 |
| 15:66067188 | DENND4A | 30.25 | 783978 | 3.86E-05 |
| 15:67463391 | SMAD3 | 42.25 | 783978 | 5.39E-05 |
| 15:74477239 | STRA6 | 36 | 783978 | 4.59E-05 |
| 15:75027880 | CYP1A1 | 95.06 | 783978 | 1.21E-04 |
| 15:75664570 | SIN3A | 64 | 783978 | 8.16E-05 |
| 15:76298132 | NRG4 | 186.78 | 783978 | 2.38E-04 |
| 15:76802175 | SCAPER | 43.56 | 783978 | 5.56E-05 |
| 15:83722059 | BTBD1 | 36 | 783978 | 4.59E-05 |
| 15:85191274 | WDR73 | 36 | 783978 | 4.59E-05 |
| 15:99276521 | IGF1R | 58.78 | 783978 | 7.50E-05 |
| 16:2003425 | RPL3L | 34.03 | 783978 | 4.34E-05 |
| 16:3747042 | TRAP1 | 39.06 | 783978 | 4.98E-05 |
| 16:16127916 | ABCC1 | 44.44 | 783978 | 5.67E-05 |
| 16:20392332 | PDILT | 600.25 | 783978 | 7.65E-04 |
| 16:28857645 | TUFM | 53.78 | 783978 | 6.86E-05 |
| 16:51735746 | LINC01571 | 100 | 783978 | 1.28E-04 |
| 16:53189672 | CHD9 | 69.44 | 783978 | 8.86E-05 |
| 16:68323115 | SLC7A6 | 45.56 | 783978 | 5.81E-05 |
| 16:69802865 | WWP2 | 49 | 783978 | 6.25E-05 |

| 16:71643669 | MARVELD3 | 27.56 | 783978 | 3.52E-05 |
| --- | --- | --- | --- | --- |
| 16:73024276 | ZFHX3 | 44.44 | 783978 | 5.67E-05 |
| 16:79942679 | LINC01229 | 49 | 783978 | 6.25E-05 |
| 16:89141490 | ACSF3 | 33.06 | 783978 | 4.22E-05 |
| 16:89708003 | CHMP1A | 100 | 783978 | 1.28E-04 |
| 17:2008278 | SMG6 | 49 | 783978 | 6.25E-05 |
| 17:12139964 | MAP2K4 | 42.25 | 783978 | 5.39E-05 |
| 17:17017267 | MPRIP | 32.11 | 783978 | 4.10E-05 |
| 17:17543846 | SMCR2 | 36 | 783978 | 4.59E-05 |
| 17:19428719 | SLC47A1 | 177.78 | 783978 | 2.27E-04 |
| 17:34882998 | MYO19 | 64 | 783978 | 8.16E-05 |
| 17:37670994 | CDK12 | 189.06 | 783978 | 2.41E-04 |
| 17:38181134 | MED24 | 44.44 | 783978 | 5.67E-05 |
| 17:54773238 | C17orf67 | 36 | 783978 | 4.59E-05 |
| 17:56755223 | TEX14 | 36 | 783978 | 4.59E-05 |
| 17:58915261 | BCAS3 | 138.06 | 783978 | 1.76E-04 |
| 17:59456589 | BCAS3 | 297.56 | 783978 | 3.79E-04 |
| 17:65373979 | PITPNC1 | 36 | 783978 | 4.59E-05 |
| 17:66449122 | PRKAR1A | 53.78 | 783978 | 6.86E-05 |
| 18:5585158 | EPB41L3 | 75.11 | 783978 | 9.58E-05 |
| 18:24393213 | AQP4 | 33.64 | 783978 | 4.29E-05 |
| 18:42346956 | SETBP1 | 31.36 | 783978 | 4.00E-05 |
| 18:46463136 | SMAD7 | 53.78 | 783978 | 6.86E-05 |
| 18:59354616 | LINC01544 | 69.44 | 783978 | 8.86E-05 |
| 18:77156537 | NFATC1 | 156.25 | 783978 | 1.99E-04 |
| 19:13053034 | CALR | 36 | 783978 | 4.59E-05 |
| 19:18843752 | CRTC1 | 36 | 783978 | 4.59E-05 |
| 19:33402419 | CEP89 | 215.11 | 783978 | 2.74E-04 |
| 19:36997147 | ZNF260 | 64 | 783978 | 8.16E-05 |
| 19:37649748 | ZNF585A | 56.25 | 783978 | 7.17E-05 |
| 19:38157969 | ZNF781 | 152.11 | 783978 | 1.94E-04 |
| 19:49214470 | MAMSTR | 49 | 783978 | 6.25E-05 |
| 19:50138143 | RRAS | 27.56 | 783978 | 3.52E-05 |
| 20:1333060 | FKBP1A-SDCBP2 | 31.04 | 783978 | 3.96E-05 |
| 20:8303120 | PLCB1 | 36 | 783978 | 4.59E-05 |
| 20:14677788 | MACROD2 | 27.56 | 783978 | 3.52E-05 |
| 20:32985155 | ITCH | 87.11 | 783978 | 1.11E-04 |
| 20:33529766 | GSS | 113.78 | 783978 | 1.45E-04 |
| 20:39970385 | LPIN3 | 49 | 783978 | 6.25E-05 |
| 20:52732362 | CYP24A1 | 162.56 | 783978 | 2.07E-04 |
| 20:56143169 | PCK1 | 56.25 | 783978 | 7.17E-05 |
| 20:57472174 | GNAS | 36 | 783978 | 4.59E-05 |
| 20:60858758 | OSBPL2 | 53.78 | 783978 | 6.86E-05 |
| 20:62152519 | PPDPF | 49 | 783978 | 6.25E-05 |
| 20:62706105 | RGS19 | 49 | 783978 | 6.25E-05 |
| 21:16576783 | NRIP1 | 75.11 | 783978 | 9.58E-05 |
| 21:35356706 | LOC101928126 | 49 | 783978 | 6.25E-05 |
| 21:37818141 | CLDN14 | 45.56 | 783978 | 5.81E-05 |
| 22:30133045 | ZMAT5 | 36 | 783978 | 4.59E-05 |
| 22:36539804 | APOL3 | 31.36 | 783978 | 4.00E-05 |
| 22:38599857 | MAFF | 58.78 | 783978 | 7.50E-05 |
| 22:40884662 | MKL1 | 108.16 | 783978 | 1.38E-04 |
| 22:43112961 | A4GALT | 113.78 | 783978 | 1.45E-04 |
| 11:72110633 | ARAP1 | 325.39 | 27079 | 1.19E-02 |
| 11:47250375 | MADD | 146.64 | 27079 | 5.39E-03 |
| 5:95754654 | PCSK1 | 79.9 | 27079 | 2.94E-03 |
| 10:114748339 | TCF7L2 | 23.13 | 27079 | 8.54E-04 |
| 15:60170447 | VPS13C/C2CD4A/β | 36.56 | 27079 | 1.35E-03 |

| 8:118254914 | SLC30A8 | 32.35 | 27079 | 1.19E-03 |
| --- | --- | --- | --- | --- |
| 11:47269468 | MADD (2nd signal)* | 25.2 | 27079 | 9.30E-04 |
| 15:68896201 | LARP6 | 12.96 | 27079 | 4.78E-04 |
| 17:2209453 | SGSM2 | 18.16 | 27079 | 6.70E-04 |
| 16:19992996 | GPR139 | 36 | 153781 | 2.34E-04 |
| 19:33926013 | PEPD | 33.06 | 153781 | 2.15E-04 |
| 11:92708710 | MTNR1B | 30.25 | 153781 | 1.97E-04 |
| 3:148622968 | CPA3 | 33.06 | 153781 | 2.15E-04 |
| 6:152032917 | ESR1 | 85.56 | 153781 | 5.56E-04 |
| 12:12890626 | APOLD1 | 33.06 | 153781 | 2.15E-04 |
| 17:7171356 | CLDN7 | 56.25 | 153781 | 3.66E-04 |
| 3:123068744 | ADCY5 | 132.25 | 153781 | 8.59E-04 |
| 7:23479013 | IGF2BP3 | 42.25 | 153781 | 2.75E-04 |
| 8:142247979 | SLC45A4 | 30.25 | 153781 | 1.97E-04 |
| 12:26872730 | ITPR2 | 25 | 153781 | 1.63E-04 |
| 15:91427612 | FES | 33.06 | 153781 | 2.15E-04 |
| 17:45968294 | SP6-SP2 | 36 | 153781 | 2.34E-04 |
| 8:41533514 | ANK1-NKX6-3 | 56.25 | 153781 | 3.66E-04 |
| 3:156795468 | CCNL1-LEKR1 | 169 | 153781 | 1.10E-03 |
| 22:29468456 | KREMEN1 | 30.25 | 153781 | 1.97E-04 |
| 12:66351826 | HMGA2 | 115.56 | 153781 | 7.51E-04 |
| 2:46484205 | EPAS1 | 132.25 | 153781 | 8.59E-04 |
| 6:130345835 | L3MBTL3 | 45.56 | 153781 | 2.96E-04 |
| 17:29037339 | SUZ12P1-CRLF3 | 38.21 | 153781 | 2.48E-04 |
| 13:78580283 | RNF219-AS1 | 43.56 | 153781 | 2.83E-04 |
| 9:113945067 | LPAR1 | 25 | 153781 | 1.63E-04 |
| 21:16339172 | NRIP1 | 32.11 | 153781 | 2.09E-04 |
| 3:46941116 | PTH1R | 27.56 | 153781 | 1.79E-04 |
| 13:40662001 | LINC00332 | 33.06 | 153781 | 2.15E-04 |
| 10:124167512 | PLEKHA1 | 27.56 | 153781 | 1.79E-04 |
| 1:22536643 | WNT4-ZBTB40 | 43.56 | 153781 | 2.83E-04 |
| 9:98245026 | PTCH1 | 78.03 | 153781 | 5.07E-04 |
| 20:31275581 | C20orf203 | 36 | 153781 | 2.34E-04 |
| 13:48882363 | RB1 | 36 | 153781 | 2.34E-04 |
| 6:20675792 | CDKAL1 | 121 | 153781 | 7.86E-04 |
| 1:154986091 | ZBTB7B | 60.06 | 153781 | 3.90E-04 |
| 20:39172728 | MAFB | 36 | 153781 | 2.34E-04 |
| 20:10658882 | JAG1 | 30.25 | 153781 | 1.97E-04 |
| 19:8787750 | ACTL9 | 31.36 | 153781 | 2.04E-04 |
| 1:212289976 | near DTL | 30.25 | 153781 | 1.97E-04 |
| 10:94468643 | HHEX-IDE | 49 | 153781 | 3.19E-04 |
| 22:42259524 | SREBF2 | 45.08 | 153781 | 2.93E-04 |
| 7:73056805 | MLXIPL | 53.08 | 153781 | 3.45E-04 |
| 4:145601863 | HHIP | 42.25 | 153781 | 2.75E-04 |
| 7:35295365 | TBX20 | 27.56 | 153781 | 1.79E-04 |
| 8:126508746 | TRIB1 | 30.25 | 153781 | 1.97E-04 |
| 9:125824055 | STRBP | 51.84 | 153781 | 3.37E-04 |
| 10:115789375 | ADRB1 | 76.56 | 153781 | 4.98E-04 |
| 1:161644871 | FCGR2B | 36 | 153781 | 2.34E-04 |
| 11:2130620 | INS-IGF2 | 43.18 | 153781 | 2.81E-04 |
| 15:99193269 | IGF1R | 33.06 | 153781 | 2.15E-04 |
| 10:104913940 | NT5C2 | 42.25 | 153781 | 2.75E-04 |
| 2:23962647 | ATAD2B | 36 | 153781 | 2.34E-04 |
| 5:157886953 | EBF1 | 39.06 | 153781 | 2.54E-04 |
| 6:34165721 | HMGA1 | 29.16 | 153781 | 1.90E-04 |
| 9:123631225 | PHF19 | 33.06 | 153781 | 2.15E-04 |
| 12:102994878 | IGF1 | 29.47 | 153781 | 1.92E-04 |
| 7:2801803 | GNA12 | 36 | 153781 | 2.34E-04 |

| 5:57091783 | 5q11.2 | 25 | 153781 | 1.63E-04 |
| --- | --- | --- | --- | --- |
| 4:17919811 | LCORL | 64 | 153781 | 4.16E-04 |
| 6:26186200 | HIST1H2BE | 36 | 153781 | 2.34E-04 |
| 2:25141538 | ADCY3 | 94.37 | 47541 | 1.98E-03 |
| 1:75003500 | TNNI3K | 43.18 | 47541 | 9.08E-04 |
| 13:54102206 | OLFM4 | 57.76 | 47541 | 1.21E-03 |
| 4:103188709 | SLC39A8 | 25.63 | 47541 | 5.39E-04 |
| 4:45175691 | GNPDA2 | 91.61 | 47541 | 1.92E-03 |
| 16:53800954 | FTO | 71.04 | 47541 | 1.49E-03 |
| 11:27748493 | BDNF | 25 | 47541 | 5.26E-04 |
| 1:72885281 | NEGR1 | 27.27 | 47541 | 5.73E-04 |
| 2:647861 | TMEM18 | 100 | 47541 | 2.10E-03 |
| 1:177889480 | SEC16B | 73.2 | 47541 | 1.54E-03 |
| 18:57829135 | MC4R | 39.06 | 47541 | 8.21E-04 |
| 12:50263148 | FAIM2 | 68.06 | 47541 | 1.43E-03 |
| 1:110082886 | GPR61 | 30.54 | 47541 | 6.42E-04 |
| 16:19979334 | GPR139 | 21.78 | 47541 | 4.58E-04 |
| 6:50803050 | TFAP2B | 47.46 | 47541 | 9.97E-04 |
| 17:5283252 | RABEP1 | 31.89 | 339224 | 9.40E-05 |
| 14:25928179 | STXBP6 | 45.76 | 339224 | 1.35E-04 |
| 2:59305625 | LINC01122 | 45.36 | 339224 | 1.34E-04 |
| 2:25150296 | ADCY3 | 98.07 | 339224 | 2.89E-04 |
| 9:129460914 | LMX1B | 31.5 | 339224 | 9.29E-05 |
| 4:45182527 | GNPDA2 | 168.16 | 339224 | 4.95E-04 |
| 9:28414339 | LINGO2 | 56.93 | 339224 | 1.68E-04 |
| 11:27684517 | BDNF | 118.7 | 339224 | 3.50E-04 |
| 12:122781897 | CLIP1 | 31.16 | 339224 | 9.18E-05 |
| 2:26928811 | KCNK3 | 37.07 | 339224 | 1.09E-04 |
| 1:96924097 | PTBP2 | 49.45 | 339224 | 1.46E-04 |
| 10:104869038 | NT5C2 | 33.77 | 339224 | 9.95E-05 |
| 1:50559820 | ELAVL4 | 32.6 | 339224 | 9.61E-05 |
| 7:75163169 | HIP1 | 37.47 | 339224 | 1.10E-04 |
| 2:63053048 | EHBP1 | 30.78 | 339224 | 9.07E-05 |
| 4:145659064 | HHIP | 31.29 | 339224 | 9.22E-05 |
| 14:30515112 | PRKD1 | 34.31 | 339224 | 1.01E-04 |
| 11:115022404 | CADM1 | 49 | 339224 | 1.44E-04 |
| 1:78446761 | FUBP1 | 40.88 | 339224 | 1.21E-04 |
| 13:54102206 | OLFM4 | 50.5 | 339224 | 1.49E-04 |
| 16:19935389 | GPRC5B | 76.75 | 339224 | 2.26E-04 |
| 1:75002193 | FPGT-TNNI3K | 60.94 | 339224 | 1.80E-04 |
| 14:29736838 | PRKD1 | 39.35 | 339224 | 1.16E-04 |
| 17:78615571 | RPTOR | 34.47 | 339224 | 1.02E-04 |
| 2:632348 | TMEM18 | 225.75 | 339224 | 6.65E-04 |
| 3:85807590 | CADM2 | 57.99 | 339224 | 1.71E-04 |
| 4:103188709 | SLC39A8 | 49.21 | 339224 | 1.45E-04 |
| 6:163033350 | PARK2 | 33.3 | 339224 | 9.82E-05 |
| 6:137675541 | IFNGR1 | 26.81 | 339224 | 7.90E-05 |
| 13:79580919 | MIR548A2 | 29.91 | 339224 | 8.82E-05 |
| 2:164567689 | FIGN | 24.26 | 339224 | 7.15E-05 |
| 3:185824004 | ETV5 | 96.13 | 339224 | 2.83E-04 |
| 2:181550962 | UBE2E3 | 32.97 | 339224 | 9.72E-05 |
| 16:53803574 | FTO | 696.28 | 339224 | 2.05E-03 |
| 3:141275436 | RASA2 | 39.35 | 339224 | 1.16E-04 |
| 8:81375457 | ZBTB10 | 28.12 | 339224 | 8.29E-05 |
| 15:68077168 | MAP2K5 | 70.65 | 339224 | 2.08E-04 |
| 4:77129568 | SCARB2 | 33.33 | 339224 | 9.83E-05 |
| 1:110154688 | GNAT2 | 55.91 | 339224 | 1.65E-04 |
| 10:102395440 | HIF1AN | 42.94 | 339224 | 1.27E-04 |

| 2:208255518 | CREB1 | 28.99 | 339224 | 8.55E-05 |
| --- | --- | --- | --- | --- |
| 8:76806584 | HNF4G | 46.08 | 339224 | 1.36E-04 |
| 19:18454825 | PGPEP1 | 30.72 | 339224 | 9.06E-05 |
| 18:21104888 | C18orf8 | 29.02 | 339224 | 8.55E-05 |
| 9:120378483 | TLR4 | 36.78 | 339224 | 1.08E-04 |
| 6:40348653 | TDRG1 | 33.15 | 339224 | 9.77E-05 |
| 8:85079709 | RALYL | 30.09 | 339224 | 8.87E-05 |
| 6:34563164 | C6orf106 | 39.87 | 339224 | 1.18E-04 |
| 19:45395619 | TOMM40 | 32.87 | 339224 | 9.69E-05 |
| 16:49062590 | CBLN1 | 29.37 | 339224 | 8.66E-05 |
| 5:75015242 | POC5 | 70.89 | 339224 | 2.09E-04 |
| 2:143043285 | LRP1B | 31 | 339224 | 9.14E-05 |
| 2:227092802 | LOC646736 | 20.69 | 339224 | 6.10E-05 |
| 11:43864278 | HSD17B12 | 30.25 | 339224 | 8.92E-05 |
| 6:50845490 | TFAP2B | 124.88 | 339224 | 3.68E-04 |
| 7:76608143 | PMS2L11 | 30.93 | 339224 | 9.12E-05 |
| 19:46202172 | QPCTL | 73.47 | 339224 | 2.17E-04 |
| 3:61236462 | FHIT | 41.62 | 339224 | 1.23E-04 |
| 16:28333411 | SBK1 | 34.98 | 339224 | 1.03E-04 |
| 1:201784287 | NAV1 | 39.57 | 339224 | 1.17E-04 |
| 21:40291740 | ETS2 | 26.27 | 339224 | 7.74E-05 |
| 19:34309532 | KCTD15 | 30.42 | 339224 | 8.97E-05 |
| 1:72751185 | NEGR1 | 116.08 | 339224 | 3.42E-04 |
| 15:51748610 | DMXL2 | 32.23 | 339224 | 9.50E-05 |
| 19:47569003 | ZC3H4 | 61.8 | 339224 | 1.82E-04 |
| 11:47650993 | MTCH2 | 71.43 | 339224 | 2.11E-04 |
| 3:81792112 | GBE1 | 30.57 | 339224 | 9.01E-05 |
| 16:28889486 | ATP2A1 | 99.36 | 339224 | 2.93E-04 |
| 11:8673939 | TRIM66 | 45.45 | 339224 | 1.34E-04 |
| 9:15634326 | C9orf93 | 33.34 | 339224 | 9.83E-05 |
| 16:30015337 | INO80E | 21.87 | 339224 | 6.45E-05 |
| 2:219349752 | USP37 | 25.98 | 339224 | 7.66E-05 |
| 1:177889480 | SEC16B | 152.74 | 339224 | 4.50E-04 |
| 20:51087862 | ZFP64 | 28.85 | 339224 | 8.50E-05 |
| 7:95169514 | ASB4 | 22.49 | 339224 | 6.63E-05 |
| 9:111932342 | EPB41L4B | 31.5 | 339224 | 9.29E-05 |
| 18:57829135 | MC4R | 238.53 | 339224 | 7.03E-04 |
| 1:49589847 | AGBL4 | 53.62 | 339224 | 1.58E-04 |
| 3:25106437 | RARB | 35.61 | 339224 | 1.05E-04 |
| 12:50247468 | BCDIN3D | 103.25 | 339224 | 3.04E-04 |
| 14:79899454 | NRXN3 | 57.47 | 339224 | 1.69E-04 |
| 15:73093991 | LOC100287559 | 29.75 | 339224 | 8.77E-05 |
| 18:40147671 | LOC284260 | 27.99 | 339224 | 8.25E-05 |
| 18:56883319 | GRP | 29.43 | 339224 | 8.68E-05 |
| 16:3627358 | NLRC3 | 36.98 | 339224 | 1.09E-04 |
| 2:213413231 | ERBB4 | 41.87 | 339224 | 1.23E-04 |
| 5:153537893 | GALNT10 | 27.65 | 339224 | 8.15E-05 |
| 10:87410904 | GRID1 | 30.95 | 339224 | 9.12E-05 |
| 10:114758349 | TCF7L2 | 47.37 | 339224 | 1.40E-04 |
| 6:120185665 | LOC285762 | 28.55 | 339224 | 8.41E-05 |
| 6:108977663 | FOXO3 | 32.46 | 339224 | 9.57E-05 |
| 13:66205704 | MIR548X2 | 27.17 | 339224 | 8.01E-05 |
| 7:93197732 | CALCR | 25.26 | 339224 | 7.45E-05 |
| 1:47684677 | TAL1 | 29.02 | 339224 | 8.55E-05 |
| 17:2005136 | SMG6 | 27.98 | 339224 | 8.25E-05 |
| 16:31129895 | KAT8 | 38.36 | 339224 | 1.13E-04 |
| 2:165513091 | GRB14 | 56.25 | 224459 | 2.51E-04 |
| 7:25858614 | AA553656f | 69.44 | 224459 | 3.09E-04 |

| 5:118729286 | TNFAIP8 | 20.46 | 224459 | 9.12E-05 |
| --- | --- | --- | --- | --- |
| 3:129334233 | PLXND1 | 39.06 | 224459 | 1.74E-04 |
| 12:26471364 | ITPR2-SSPN | 88.58 | 224459 | 3.94E-04 |
| 1:170372503 | GORAB | 47.02 | 224459 | 2.09E-04 |
| 9:107735920 | ABCA1 | 74.15 | 224459 | 3.30E-04 |
| 11:63862612 | MACROD1-VEGFB | 129.71 | 224459 | 5.78E-04 |
| 18:60845884 | BCL2 | 16.83 | 224459 | 7.50E-05 |
| 19:18389135 | JUND | 37.35 | 224459 | 1.66E-04 |
| 8:72514228 | MSC | 45.56 | 224459 | 2.03E-04 |
| 6:6738752 | LY86 | 78.45 | 224459 | 3.49E-04 |
| 6:43764551 | VEGFA | 117.36 | 224459 | 5.23E-04 |
| 2:66200648 | MEIS1 | 61.43 | 224459 | 2.74E-04 |
| 15:67033151 | SMAD6 | 36 | 224459 | 1.60E-04 |
| 12:54342684 | HOXC13 | 49 | 224459 | 2.18E-04 |
| 7:26397239 | SNX10 | 9.34 | 224459 | 4.16E-05 |
| 2:188115398 | CALCRL | 38.15 | 224459 | 1.70E-04 |
| 3:156797609 | LEKR1 | 55.18 | 224459 | 2.46E-04 |
| 6:34195011 | HMGA1 | 59.17 | 224459 | 2.64E-04 |
| 3:12489342 | PPARG | 36 | 224459 | 1.60E-04 |
| 6:127452116 | RSPO3 | 121.56 | 224459 | 5.41E-04 |
| 20:34023962 | GDF5 | 21.63 | 224459 | 9.64E-05 |
| 3:52637486 | PBRM1 | 23.99 | 224459 | 1.07E-04 |
| 22:29449477 | ZNRF3-KREMEN1 | 48.23 | 224459 | 2.15E-04 |
| 3:64718258 | ADAMTS9 | 112.11 | 224459 | 4.99E-04 |
| 1:119574587 | TBX15-WARS2 | 36.95 | 224459 | 1.65E-04 |
| 1:219753509 | LYPLAL1 | 105.97 | 224459 | 4.72E-04 |
| 16:81534790 | CMIP | 11.11 | 224459 | 4.95E-05 |
| 4:124066948 | SPATA5-FGF2 | 29.99 | 224459 | 1.34E-04 |
| 4:56482750 | NMU | 6 | 224459 | 2.67E-05 |
| 19:33824946 | CEBPA | 84.83 | 224459 | 3.78E-04 |
| 17:17420199 | PEMT | 12.96 | 224459 | 5.77E-05 |
| 12:124440110 | CCDC92 | 67.82 | 224459 | 3.02E-04 |
| 20:45558831 | EYA2 | 31.82 | 224459 | 1.42E-04 |
| 5:176527577 | FGFR4 | 39.51 | 224459 | 1.76E-04 |
| 1:172352990 | DNM3-PIGC | 56.25 | 224459 | 2.51E-04 |
| 5:173320815 | CPEB4 | 56.25 | 224459 | 2.51E-04 |
| 6:32381736 | BTNL2 | 40.82 | 224459 | 1.82E-04 |
| 7:27223771 | HOXA11 | 41.33 | 224459 | 1.84E-04 |
| 8:23603324 | NKX2-6 | 41.87 | 224459 | 1.86E-04 |
| 10:104487443 | SFXN2 | 16 | 224459 | 7.13E-05 |
| 15:56504598 | RFX7 | 51.02 | 224459 | 2.27E-04 |
| 15:31708263 | KLF13 | 36.56 | 224459 | 1.63E-04 |
| 17:68453345 | KCNJ2 | 20.25 | 224459 | 9.02E-05 |
| 1:154991389 | DCST2 | 39.06 | 224459 | 1.74E-04 |
| 5:55861894 | MAP3K1 | 44.44 | 224459 | 1.98E-04 |
| 20:6623374 | BMP2 | 25.95 | 224459 | 1.16E-04 |
| 4:89713121 | FAM13A | 31.23 | 224459 | 1.39E-04 |
| 3:141140968 | ZBTB38 | 36 | 182904 | 1.97E-04 |
| 7:23502974 | IGF2BP3 | 64 | 182904 | 3.50E-04 |
| 9:125918772 | STRBP | 25 | 182904 | 1.37E-04 |
| 11:111529037 | IL18 | 144 | 5888 | 2.39E-02 |
| 11:111542224 | NA | 121 | 5888 | 2.01E-02 |
| 11:111585594 | BCO2 | 100 | 5888 | 1.67E-02 |
| 11:111590526 | BCO2 | 100 | 5888 | 1.67E-02 |
| 2:316411522 | SRD5A2 | 49 | 5888 | 8.26E-03 |
| 2:31637256 | SRD5A2 | 49 | 5888 | 8.26E-03 |
| 2:32115705 | DPY30 | 64 | 5888 | 1.08E-02 |
| 2:32116591 | DPY30 | 64 | 5888 | 1.08E-02 |

| 2:32122090 | NA | 64 | 5888 | 1.08E-02 |
| --- | --- | --- | --- | --- |
| 2:32132286 | NA | 64 | 5888 | 1.08E-02 |
| 2:32143250 | SPAST | 49 | 5888 | 8.26E-03 |
| 2:32160890 | SPAST | 36 | 5888 | 6.08E-03 |
| 2:32266336 | SLC30A6 | 49 | 5888 | 8.26E-03 |
| 2:32284611 | SLC30A6 | 49 | 5888 | 8.26E-03 |
| 2:32311041 | NLRC4 | 36 | 5888 | 6.08E-03 |
| 2:32342662 | NLRC4 | 49 | 5888 | 8.26E-03 |
| 1:107549245 | NA | 36 | 312571 | 1.15E-04 |
| 1:109817590 | NA | 357.84 | 312571 | 1.14E-03 |
| 1:110082886 | NA | 68.97 | 312571 | 2.21E-04 |
| 1:110163879 | NA | 52.75 | 312571 | 1.69E-04 |
| 1:110470764 | NA | 61.67 | 312571 | 1.97E-04 |
| 1:150940625 | NA | 91.39 | 312571 | 2.92E-04 |
| 1:156700651 | NA | 40.5 | 312571 | 1.30E-04 |
| 1:172346548 | NA | 44.89 | 312571 | 1.44E-04 |
| 1:178515312 | NA | 93.12 | 312571 | 2.98E-04 |
| 1:182157235 | NA | 145 | 312571 | 4.64E-04 |
| 1:205631767 | NA | 38.67 | 312571 | 1.24E-04 |
| 1:219631981 | NA | 71.88 | 312571 | 2.30E-04 |
| 1:220970593 | NA | 90.63 | 312571 | 2.90E-04 |
| 1:230295691 | NA | 496.65 | 312571 | 1.59E-03 |
| 1:230416744 | NA | 65.11 | 312571 | 2.08E-04 |
| 1:234853406 | NA | 33.06 | 312571 | 1.06E-04 |
| 1:23734350 | NA | 41.15 | 312571 | 1.32E-04 |
| 1:26902388 | NA | 49.48 | 312571 | 1.58E-04 |
| 1:27236212 | NA | 156.63 | 312571 | 5.01E-04 |
| 1:28344980 | NA | 41.75 | 312571 | 1.34E-04 |
| 1:40028180 | NA | 277.78 | 312571 | 8.88E-04 |
| 1:63113719 | NA | 40.64 | 312571 | 1.30E-04 |
| 1:93584606 | NA | 105.8 | 312571 | 3.38E-04 |
| 10:101912064 | NA | 74.82 | 312571 | 2.39E-04 |
| 10:113940329 | NA | 158.53 | 312571 | 5.07E-04 |
| 10:114048792 | NA | 63.43 | 312571 | 2.03E-04 |
| 10:115789375 | NA | 61.12 | 312571 | 1.96E-04 |
| 10:17260290 | NA | 34.81 | 312571 | 1.11E-04 |
| 10:46013277 | NA | 113.47 | 312571 | 3.63E-04 |
| 10:8601074 | NA | 36.8 | 312571 | 1.18E-04 |
| 10:94839642 | NA | 105.06 | 312571 | 3.36E-04 |
| 11:109995944 | NA | 56.25 | 312571 | 1.80E-04 |
| 11:116611827 | NA | 40.11 | 312571 | 1.28E-04 |
| 11:116633947 | NA | 108.06 | 312571 | 3.46E-04 |
| 11:116648917 | NA | 1815.86 | 312571 | 5.78E-03 |
| 11:116701354 | NA | 775.09 | 312571 | 2.47E-03 |
| 11:116707044 | NA | 71.64 | 312571 | 2.29E-04 |
[truncated: 372,023 more chars]
